# A Nurr1 agonist derived from the natural ligand DHI induces neuroprotective gene expression

**DOI:** 10.1021/acs.jmedchem.4c03104

**Published:** 2025-02-07

**Authors:** Markus Egner, Romy Busch, Úrsula López-García, Max Lewandowski, Georg Höfner, Thomas Wein, Julian A. Marschner, Daniel Merk

**Affiliations:** 1 Ludwig-Maximilians-Universität München, Department of Pharmacy, 81377 Munich, Germany

**Keywords:** Nuclear receptor related 1, NR4A2, Parkinson’s disease, Alzheimer’s disease, multiple sclerosis

## Abstract

The dopamine metabolite 5,6-dihydroxyindole (DHI) has been discovered as natural Nurr1 ligand with potential biological relevance and is an attractive lead for Nurr1 modulator development but exhibits chemical reactivity and weak potency. We have systematically explored the SAR of 5-chloroindole-6-carboxamide as DHI mimetic scaffold and identified the first high-affinity (K_d_ 0.08 - 0.12 μM) ligands of the DHI binding site of Nurr1. An optimized Nurr1 agonist of this scaffold endowed with favorable physicochemical properties, high selectivity and low toxicity emerges as chemical tool to explore the biological impact of Nurr1 activation via the DHI binding site. Treatment of neuronal cells with this compound mediated enhanced expression of Nurr1-regulated neuroprotective genes like brain-derived neurotrophic factor (BDNF) supporting the great potential of Nurr1 activation in neurodegeneration.

## Introduction

**Chart 1:**
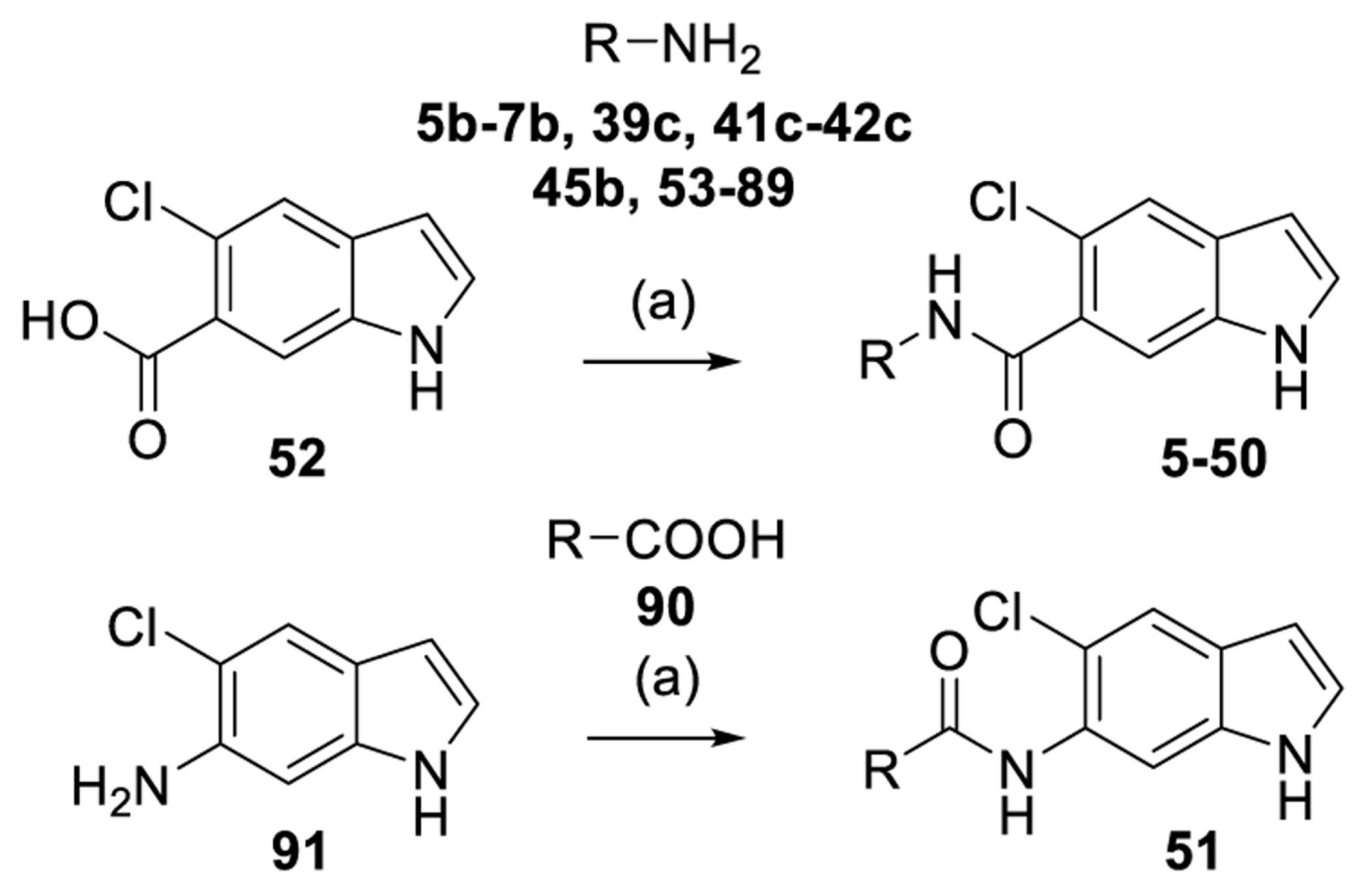
Nurr1 agonists

The ligand-activated transcription factor nuclear receptor related 1 (Nurr1, NR4A2) has neuroprotective properties and is considered as promising target in various degenerative diseases^1^. Nurr1 is required for (dopaminergic) neuron development and maintenance^1–3^ but diminished neural expression levels were observed in Alzheimer’s (AD) and Parkinson’s disease (PD) patients^4^ and rodent models^5–7^. Correspondingly, promoting Nurr1 activity by overexpression^5,6,8,9^ or with the early Nurr1 agonist tool amodiaquine (AQ, **1**, Chart 1)^10,11^ had beneficial effects in preclinical models of neurodegenerative diseases. Nurr1 activators may therefore open new therapeutic avenues in neurodegeneration. In addition, preliminary evidence suggests potential of Nurr1 modulation in age-related macular degeneration^12^, metabolic diseases^13^ and inflammatory arthritis^14^.

Nurr1 is a constitutively active transcriptional inducer also in absence of a bound agonist but its activity can be modulated by ligands in a bidirectional manner^15,16^. To date, unsaturated fatty acids^17^ and prostaglandins^18^ as well as the dopamine metabolite 5,6-dihydroxyindole (DHI, **2**)^19^ have been discovered as endogenous Nurr1 ligands. Among them, prostaglandin A1^18^ and DHI^19^ bind covalently to Cys566 of Nurr1 in partially solvent-exposed pockets behind helix 12 of the Nurr1 LBD. Since instability and reactivity of DHI complicate studies on its role as natural Nurr1 ligand, 5-chloroindole (**3**) has been developed as valuable DHI mimetic^20^, and we have recently designed **4**^21^ by exploiting the exposed binding site of DHI. **4** exhibits significantly enhanced Nurr1 agonist potency (EC_50_ = 3 μM) compared to DHI and has the rare characteristic among synthetic Nurr1 ligands that its binding epitope is known rendering it as an attractive lead for Nurr1 modulator development. Here we explored the structure-activity relationship of **4** as Nurr1 agonist focusing on the amide linker and the solvent-exposed amide substituent. We identified analogues with markedly increased cellular Nurr1 agonist potency and binding affinity and observed beneficial impact on protective gene expression in neuronal cells upon treatment with an optimized DHI descendant.

## Results & Discussion

The DHI descendant **4** was designed^21^ based on the unique binding mode of DHI (pdb ID: 6dda)^19^ and to address the binding pocket for the indole behind helix 12 of the Nurr1 LBD while additionally extending to surface pockets. Therefore, we kept the indole as essential motif fixed and commenced structural optimization of **4** by scanning importance of all elements in the 5-chloro-2-(2-(dimethylamino)ethoxy)aniline substituent (Table 1).

Both replacement of the chloro substituent of **4** by a trifluoromethyl group (**5**) or its removal (**6**) were not tolerated suggesting importance of the substituent in meta position. Introduction of a methylene bridge between the phenyl motif and the amide (**7**) also led to loss of Nurr1 agonism indicating that enhanced flexibility was not favored and that arylamides were preferred. In contrast, deconstruction of the dimethylaminoethoxy substituent of **4** to a methoxy group (**8**) strongly enhanced potency. The alternative regiochemistry of **9** with the methoxy group moved to the meta position was less favored. The further deconstructed chloroaniline **10** lacking the alkoxy group interestingly retained submicromolar Nurr1 agonism and was preferable as the 1,2-aminoalkoxy motif of **4, 5, 6** and **8** might be oxidation sensitive.

Based on these favorable results on simplification of the scaffold of **4**, we next evaluated monosubstituted analogues and scanned the favored position for the chlorine substituent. While the ortho-chloro analogue **11** was slightly less active, para-chloro substitution in **12** provided a marked improvement in potency. The corresponding methyl series (**13**-**15**) revealed a similar SAR with preference for meta (**14**) and para (**15**) substitutions. Fluorine (**16, 17**) was slightly less favored but all meta and para substituted derivatives (**10, 12, 14**-**17**) outmatched the unsubstituted aniline derivative **18**.

Therefore, we probed alternative substituents in the preferred meta and para positions (Table 2). As observed for **5**, replacement of chlorine (**10, 12**) by trifluoromethyl (**19, 20**) was not favored. The bulky and hydrophobic meta and para biphenyls (**21, 22**) as well as a tert-butyl group in meta position (**23**) also disrupted Nurr1 agonism while the latter was tolerated in para position (**24**) suggesting potentially more available space in this direction. Methoxy substituents in meta (**25**) and para (**26**) position outmatched both the chloro (**10, 12**) and methyl (**14, 15**) analogues but like previous derivatives (**4, 5, 6** and **8**), the highly potent paramethoxy aniline **26** may be oxidation sensitive and has potential PAINS character^22,23^ contradicting further evaluation.

Double substitution in meta and para positions with dimethyl (**27**) or dichloro (**28**) despite failing to outmatch the para-chloro (**12**) and para-methyl (**15**) derivatives, retained considerable Nurr1 agonism suggesting that further extension could be possible, and we moved our attention to bicyclic motifs. The indane derivative **29** exhibited substantially enhanced Nurr1 agonism which was also evident from enhanced affinity (K_d_ = 0.08 μM, Figure S1) according to isothermal titration calorimetry (ITC). The homologous tetrahydronaphthalene **30** as well as the naphthalene **31** exhibited similar potency as **27**/**28** supporting the hypothesis that bicyclic motifs would be favored and, hence, we evaluated various bicyclic aromatic systems in this respective position (Table 3).

The 5-indole analogue **32** albeit not providing increased potency revealed favorably enhanced Nurr1 activation efficacy while the 6-indole derivative **33** retained the high potency and moderate efficacy of **29** and **31**. Significant improvement in potency was achieved with quinoline substituents (**34-37**) wherein the 3-quinoline regiochemistry (**37**) was preferred and achieved double-digit nanomolar potency with favorable activation efficacy. Further evaluation of this 3-quinoline scaffold demonstrated that reduction to the tetrahydroquinoline **38** and introduction of chloro (**39, 40**) or methyl (**41, 42**) substituents in 6- or 7-position decreased or disrupted Nurr1 agonism indicating that further substituents or extension would not be productive.

Several bicyclic substituents on the 5-chloroindole-6-carboxamide scaffold either provided enhanced potency (indane **29**, quinolines **34-37**) or higher activation efficacy (indole **32**, quinolines **34-37**) than the lead. Especially the presence of nitrogen in the bicyclic motifs appeared favorable. Based on these observations, we tested fused motifs (**43-45**) for potentially enhanced potency and efficacy. A 1,5-naphthyridin-3-yl substituent (**43**) incorporating two of the favored nitrogen atoms failed to outmatch the quinoline derivatives **34-37** in potency or efficacy. The pyrrolo[2,3-b]pyridin-5-yl derivative **44** fusing the favored quinolin-3-yl (**37**) and indol-5-yl (**32**) motifs as well as its N-methyl analogue **45** exhibited strong efficacy but slightly reduced potency. **44** and **45** were equipotent suggesting that the indole nitrogen did not act as H-bond donor and hence we evaluated the benzofuran-5-yl analogue **46** which revealed both strong potency and efficacy. Similar to the matched pair **32**/**33**, the inverted benzofuran-6-yl derivative **47** exhibited reduced efficacy.

Eventually we explored the impact of modifications in the amide linker on the scaffold’s Nurr1 agonist potency (Table 4). As **37** displayed the most favorable overall Nurr1 agonist profile considering potency and efficacy, we studied further variations on this compound. The secondary amide linker turned out as highly favored as the tertiary N-methyl amide **48** was inactive on Nurr1 and the corresponding secondary amine **49** displayed significantly reduced potency. Shifting the amide linker from the 3- (**37**) to the 4-position of the quinoline (**50**) was also not tolerated. The inverted amide **51** retained strong Nurr1 agonist potency but was still less active than **37**.

**Figure 1:**
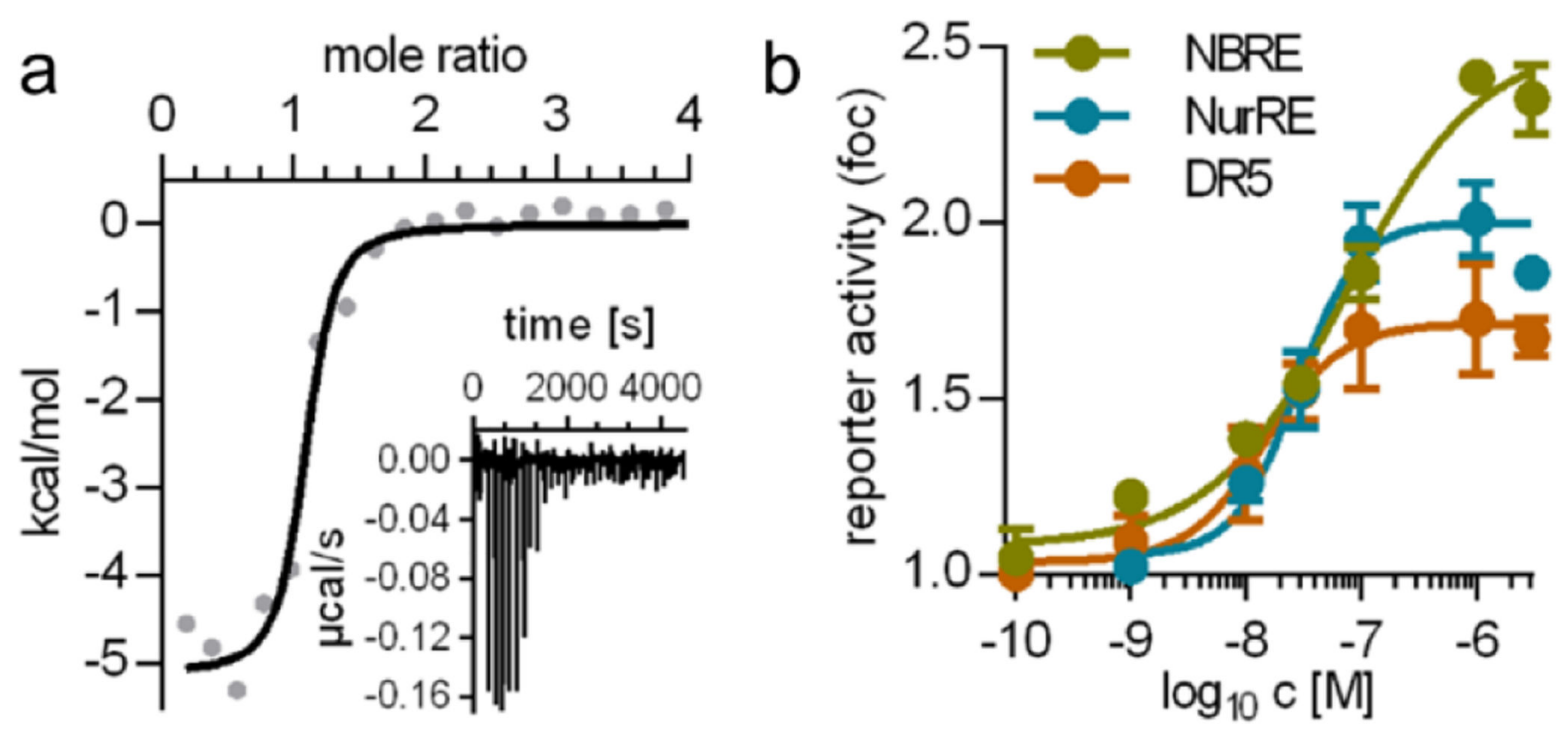
Nurr1 agonism of 37. (a) Isothermal titration calorimetry (ITC) demonstrated high affinity (Kd 0.12 μM) binding of **37** to the recombinant Nurr1 LBD. The fitting of the heat of binding is shown and the isotherm at 25°C is shown as inset. (b) **37** activated full-length human Nurr1 on the response elements for the monomer (NBRE, EC_50_ 0.07±0.02 μM), homodimer (NurRE, EC_50_ 0.027±0.008 μM) and RXR heterodimer (DR5, EC_50_ 0.014±0.006 μM) with consistently low nanomolar potency. Data are the mean±S.E.M., n≥4.

Considering potency, efficacy and the lack of unfavorable chemical motifs, the quinolin-3-yl derivative **37** thus emerged as most favored DHI descendant. Further profiling of **37** as Nurr1 modulator revealed high affinity to the Nurr1 LBD (Kd 0.12 μM, ITC, Figure 1a) and robust activation of full-length Nurr1 on all human response elements (NBRE, NurRE, DR5; Figure 1b).

**Figure 2:**
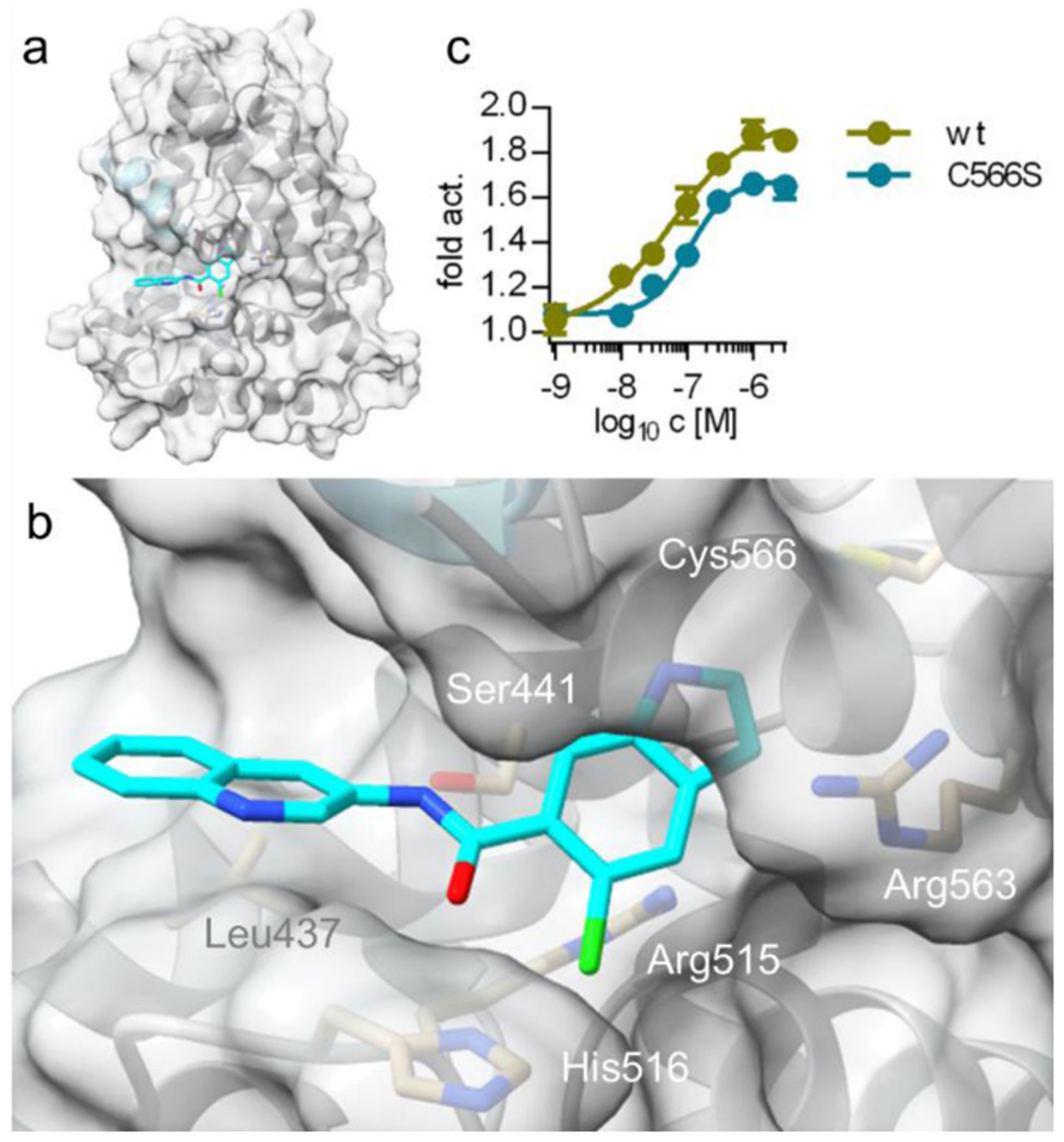
Binding of **37** to the DHI binding site in the Nurr1 LBD. (a) Overview of the Nurr1 LBD (pdb ID 6dda^19^) with **37** (cyan) docked to the DHI binding site. (b) Predicted binding mode of **37**. The chloroindole favorably occupies the DHI binding pocket behind helix 12 lined by Arg515, Arg563 and Cys566. The quinoline motif is bound in a rather hydrophobic surface pocket (Leu437) and the amide engages H-bonds to Ser441 and His516. (c) **37** exhibited slightly reduced potency and efficacy on C566S Nurr1 (EC_50_ 0.11±0.02 μM, 1.7±0.1-fold act.) compared to the wildtype (wt; EC_50_ 0.06±0.02 μM, 1.9±0.1-fold act.) suggesting importance of Cys566 for interaction but no covalent binding. Data are the mean±S.E.M., n≥4.

Based on the known binding site of DHI^19^ in the Nurr1 LBD and the hypothesis that the descendants address the same binding epitope, we modeled and evaluated the interaction of **37** with Nurr1 (Figure 2). The predicted binding mode indicated favorable occupation of the DHI pocket^19^ behind helix 12 by **37**. The 5-chloroindole motif was bound between Arg515 and Arg563 with potential cation-π interactions and faced Cys566. Additionally, **37** extended to a rather hydrophobic (Leu437) surface pocket between the end of helix 12 and the loop connecting helices 9 and 10. The amide nitrogen and carbonyl engaged H-bonds to Ser441 and His516, respectively, aligning with the observation that *N*-methylation to the tertiary amide **48** and reduction to the corresponding amine **49** were not tolerated. The quinoline motif was partly solvent exposed.

DHI forms a covalent adduct with Cys566 of Nurr1 which is enabled by oxidation to a quinone that can accept additional electrons rendering the 2-position of the indole electrophilic^19^. The DHI descendant **37**, in contrast, exhibits likely insufficient electrophilicity to react with Cys566 which was supported by similar agonism of **37** on the C566S Nurr1 mutant^21^ compared to wildtype (Figure 2c). Potency and efficacy of **37** were slightly diminished on C566S Nurr1 (EC_50_ 0.11±0.02 μM, 1.7±0.1-fold act.) indicating that the cysteine residue was relevant for interaction with the ligand, but the moderate potency difference suggested no covalent binding.

**Figure 3:**
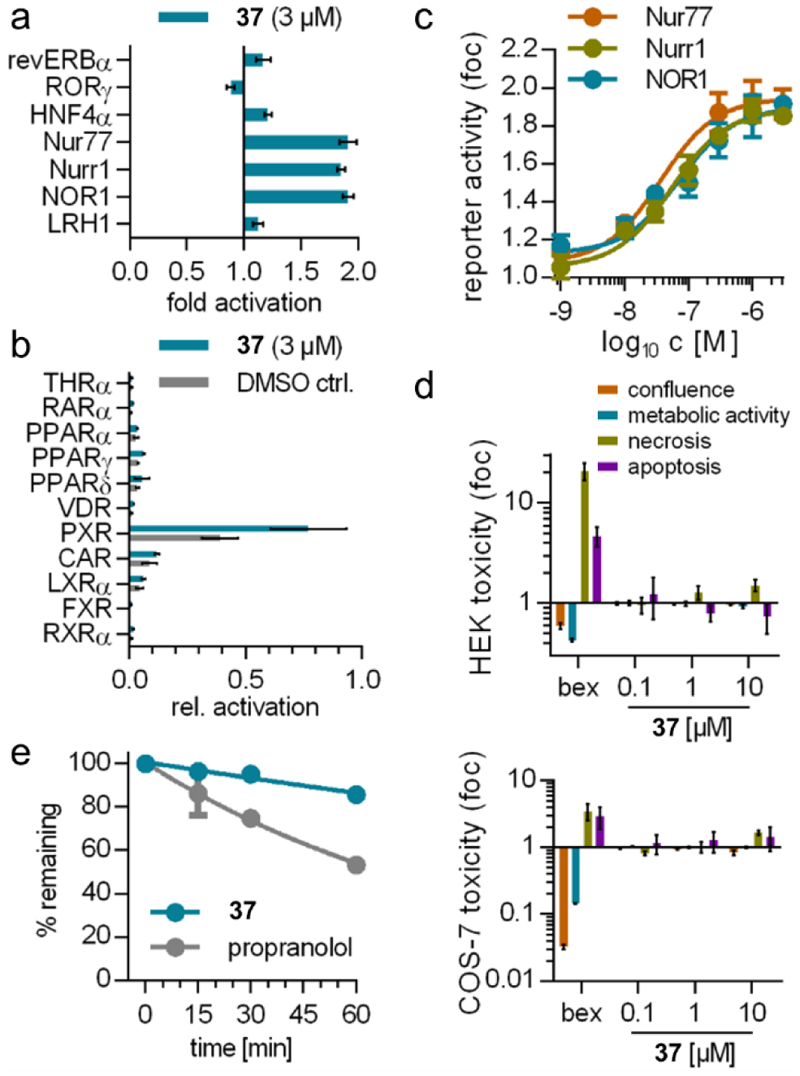
In vitro characterization of 37. (a) Selectivity profiling of **37** on constitutively active nuclear receptors in uniform Gal4-hybrid reporter gene assays. Data are the mean±S.E.M. fold activation vs. DMSO ctrl; n=3. (b) Selectivity profiling of **37** on lipid-activated nuclear receptors in uniform Gal4-hybrid reporter gene assays. Data are the mean±S.E.M. relative activation vs. reference ligands; n=3. Reference ligands are listed in the methods section. (c) Dose-response curves of **37** on the NR4A receptors. Data are the mean±S.E.M., n≥3. (d) Effects of **37** in a multiplex toxicity assay measuring confluence, metabolic activity, necrosis and apoptosis in HEK293T and COS-7 cells. Bexarotene (100 μM) as positive control. Data are the mean±S.E.M., n≥3. (e) Stability of **37** against degradation by rat liver microsomes. Propranolol for comparison. Data are the mean±S.E.M., n=3.

To assess the potential of **37** as Nurr1 agonist and as chemical tool, we characterized its selectivity, toxicity, physicochemical and in vitro pharmacokinetic properties. Profiling of **37** on nuclear receptors outside the NR4A family (Figure 3a,b) revealed moderate PXR activation as only off-target at 3 μM (EC_50_ = 1.5±0.6 μM, Figure S2) corresponding to at least 25-fold selectivity. Within the NR4A family, **37** exhibited similar agonism on all three human receptors (Figure 3c) which is a general issue of most NR4A ligands with few exceptions^1,24,25^. A multiplex toxicity assay monitoring confluence, metabolic activity, apoptosis and necrosis in HEK293T and COS-7 cells revealed no toxic effects of **37** up to 10 μM (Figure 3d). Moreover, **37** exhibited attractive physicochemical properties with an experimentally determined logP of 2.83 (Supporting Information, Figure S3) and 4.4 mg/L (13.8 μM) aqueous solubility. The stability of **37** against degradation by rat liver microsomes was very high (Figure 3e) indicating favorable pharmacokinetics.

**Figure 4:**
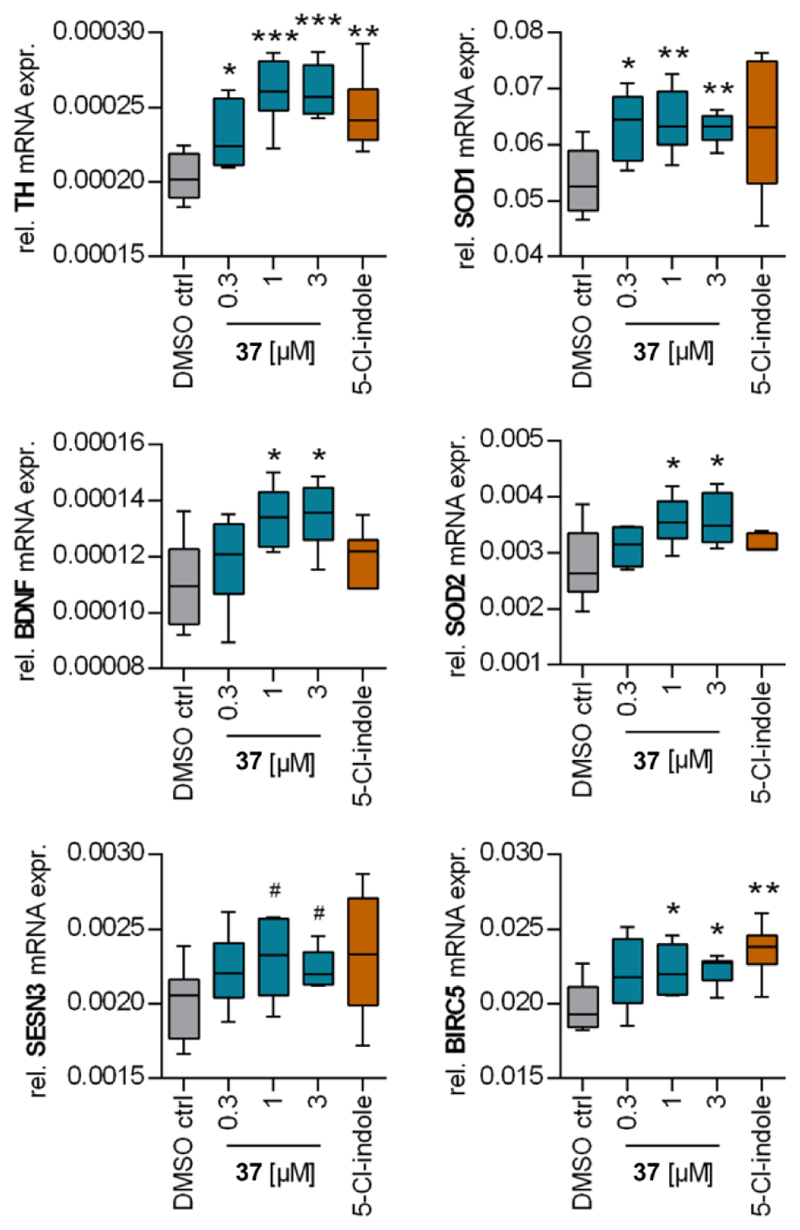
Effects of **37** on Nurr1 regulated gene expression in rat dopaminergic neurons (N27). 5-Chloroindole (**3**, 100 μM) for comparison. TH – tyrosine hydroxylase, SOD1/2 – superoxide dismutase 1/2, BDNF – brain derived neurotrophic factor, SESN3 – sestrin 3, BIRC5 – baculoviral inhibitor of apoptosis repeat-containing 5 (also termed survivin). Boxplots are min.-max.; n=6. ^#^ p<0.1, * p<0.05, ** p<0.01, *** p<0.001 (t-test vs. DMSO control).

To capture cellular effects of **37** and validate cellular target engagement in native setting, we evaluated the compound’s impact on Nurr1 regulated gene expression in rat dopaminergic neurons (N27)^26^. **37** robustly induced tyrosine hydroxylase (TH) and superoxide dismutase 1 (SOD1) and 2 expression suggesting protective activity in dopaminergic neurons (Figure 4). Neuronal cells treated with **37** also displayed significantly enhanced mRNA expression of brain derived neurotrophic factor (BDNF), which supports survival of neurons, promotes neuronal differentiation and plays a key role in synaptic plasticity^27,28^. Further neuroprotective effects of **37** were evident from enhanced expression of sestrin 3 (SESN3) and baculoviral inhibitor of apoptosis repeat-containing 5 (BIRC5). SESN3 can protect from reactive oxygen species (ROS) induced damage and counteract ageing^29^. BIRC5, also termed survivin, is a caspase inhibitor and can protect from apoptotic cell death^30^. It can promote neuronal survival^31^ and there is evidence that survivin can enhance cognitive function in Alzheimer’s disease^32,33^. Altogether, the transcriptional response to treatment with **37** indicated neuroprotective activity of the Nurr1 agonist. These effects of **37** were dose-dependent and appeared to reach a plateau at 1 μM underscoring the compound’s high Nurr1 agonist potency. 5-Chloroindole (**3**, 100 μM) tended to exhibit similar but less consistent and weaker effects.

**Figure 5:**
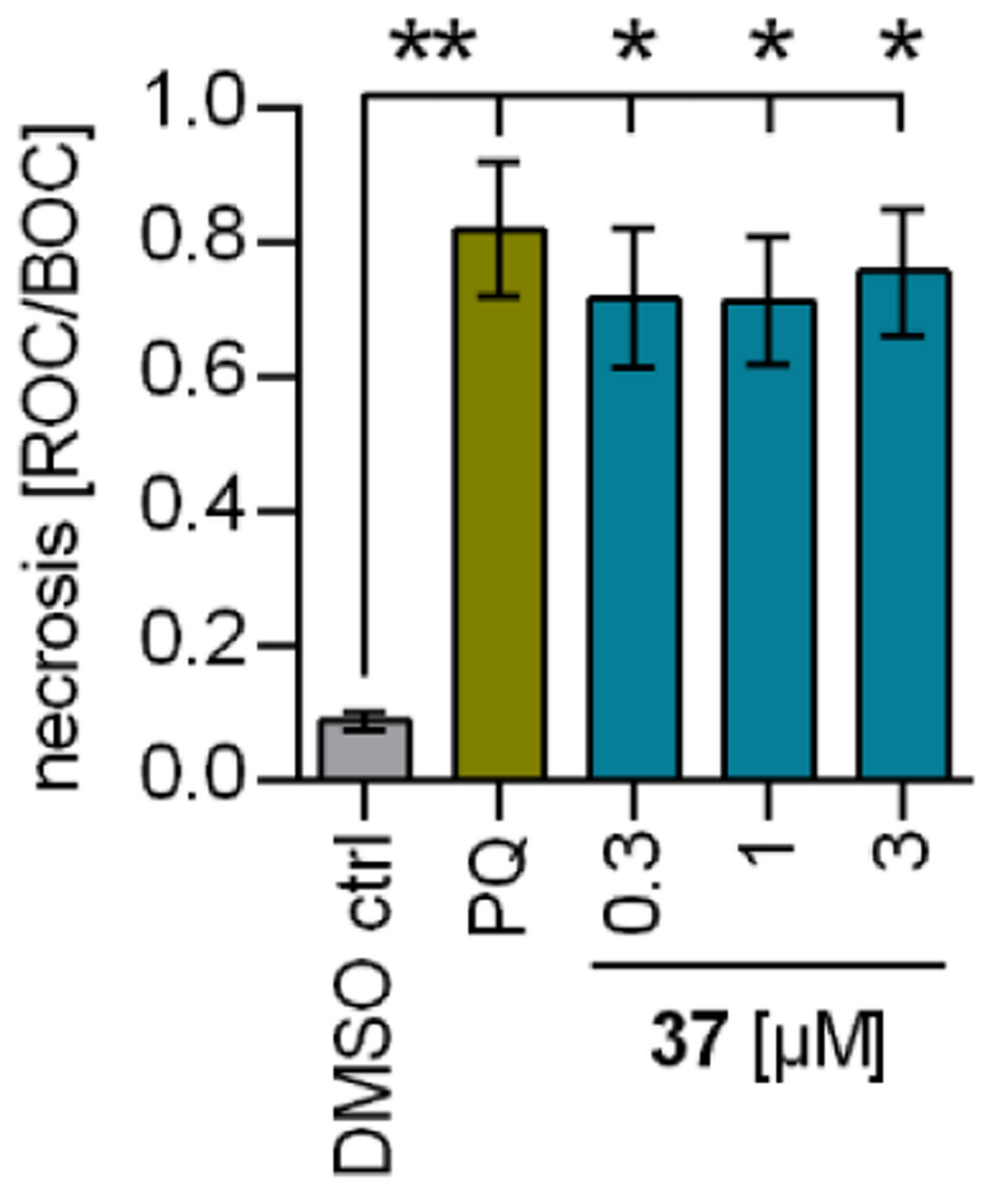
Effects of the Nurr1 agonist **37** on necrosis of rat dopaminergic neurons (N27) induced by the neurotoxin paraquat (PQ, 600 μM). Data are the mean±S.E.M.; n=5; * p<0.05, ** p<0.01 (ANOVA with Tukey’s multiple comparisons test).

Based on the gene expression effects of **37** pointing to protection against oxidative stress and cell death, we evaluated the impact of **37** on the health of neuronal cells treated with the neurotoxin paraquat (PQ). PQ interferes with electron transport and induces oxidative stress and mitochondrial dysfunction. It is used as a pesticide and potentially linked to increased PD risk. PQ can be employed to induce loss of dopaminergic neurons in in vivo PD models^34^ and protective effects of SOD activity and a SOD mimetic have been observed in PQ-induced PD in vivo^35^ suggesting that the gene expression induced by **37** might protect from PQ toxicity. Treatment of rat dopaminergic neurons (N27)^26^ with PQ indeed caused substantial necrosis (Figure 5) and pretreatment with **37** to induce protective gene expression slightly but significantly ameliorated the toxic effects of PQ supporting neuroprotective activity.

## Conclusion

Using natural ligands as templates for drug design has been very fruitful in the field of nuclear receptors as exemplified by glucocorticoids^36^ or the FXR agonist obeticholic acid^37^. The natural Nurr1 ligand DHI (**2**)^19^, therefore, appeared very attractive as lead for Nurr1 agonist discovery. Additionally, **2** is one of very few Nurr1 modulators whose binding epitope in the LBD is known opening access to improved molecular understanding of Nurr1 modulation by ligands. DHI (**2**) and the optimized analogue **3** are weak Nurr1 modulators, however, and not suitable to explore the biological impact of Nurr1 activation via the natural ligand binding site. Therefore, we set out to develop potent Nurr1 agonists acting via the same epitope as the natural ligand as chemical tools. The optimized DHI descendant **37** is several orders of magnitude more potent than the natural ligand and its activity has been validated in several cellular and cell-free settings. It exhibits nanomolar Nurr1 binding affinity, robust target engagement in cellular setting at 1 μM, >25-fold selectivity over nuclear receptors outside the NR4 family, and no cytotoxicity (Table 5) rendering **37** as valuable chemical tool to probe biological effects of Nurr1 modulation via the DHI binding site. In neuronal cells, treatment with **37** mediated induction of several neuroprotective genes underlining the great potential of Nurr1 activation in neurodegeneration.

## Chemistry

**Scheme 1:**
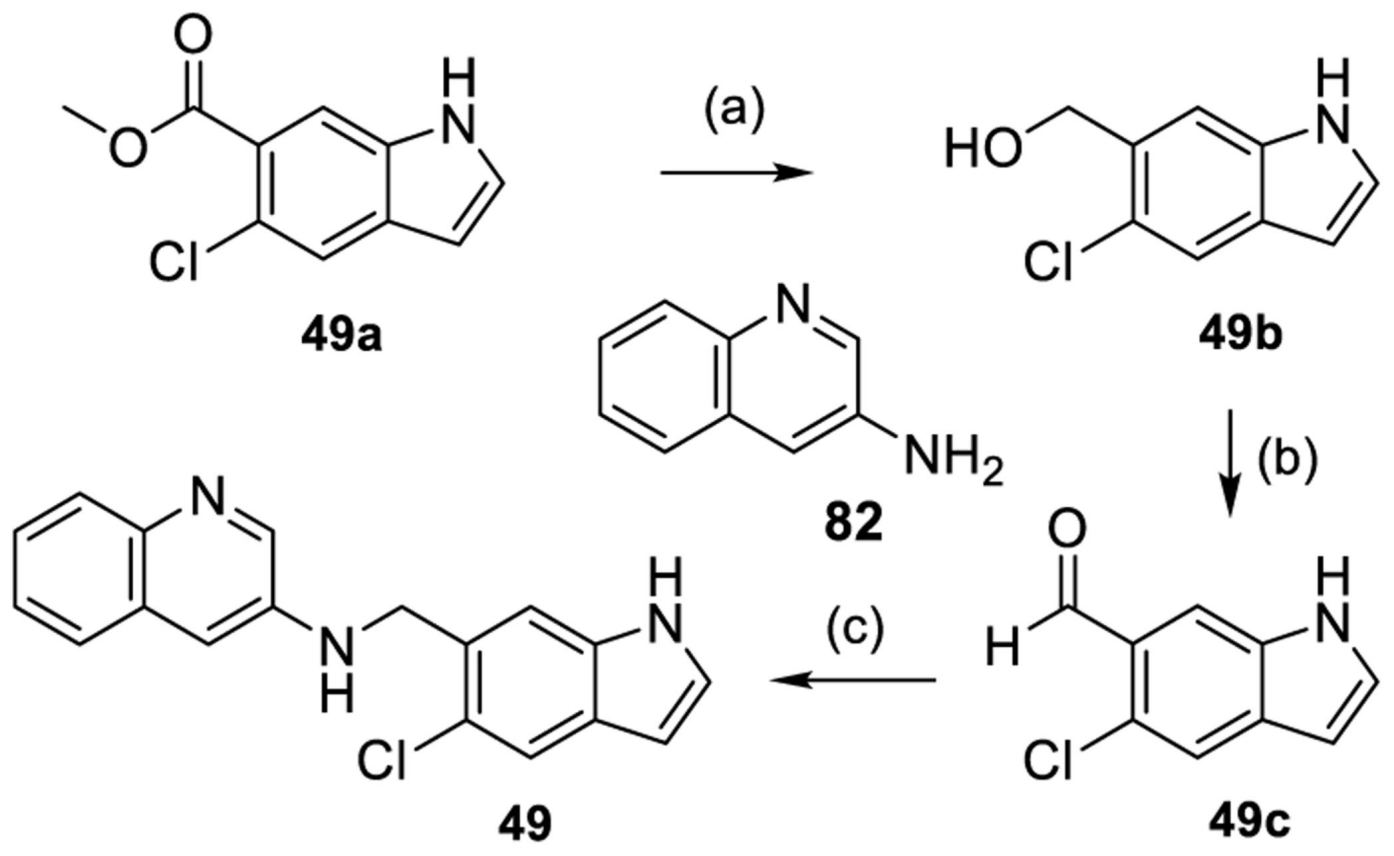
Synthesis of 5-51.^a^ ^a^ Reagents & Conditions: (a) NMI, TCFH, MeCN, DMF, 24 h, rt, 6-97%.

Compounds **5-48** and **50** were prepared according to Scheme 1 by amide coupling of 5-chloroindole-6-carboxylic acid (**52**) with the respective amines **5b-7b, 39c, 41c-42c, 45b, 48a** and **53-89** using *N*-[chloro(dimethylamino)methylene]-*N* methylmethanaminium (TCFH) and 1-methylimidazole (NMI) as coupling reagent^39^. The inverted analogue **51** was obtained similarly by TCFH/NMI-mediated amide coupling of quinoline-3-carboxylic acid (**90**) and 5-chloro-1*H*-indol-6-amine (**91**).

**Scheme 2:**
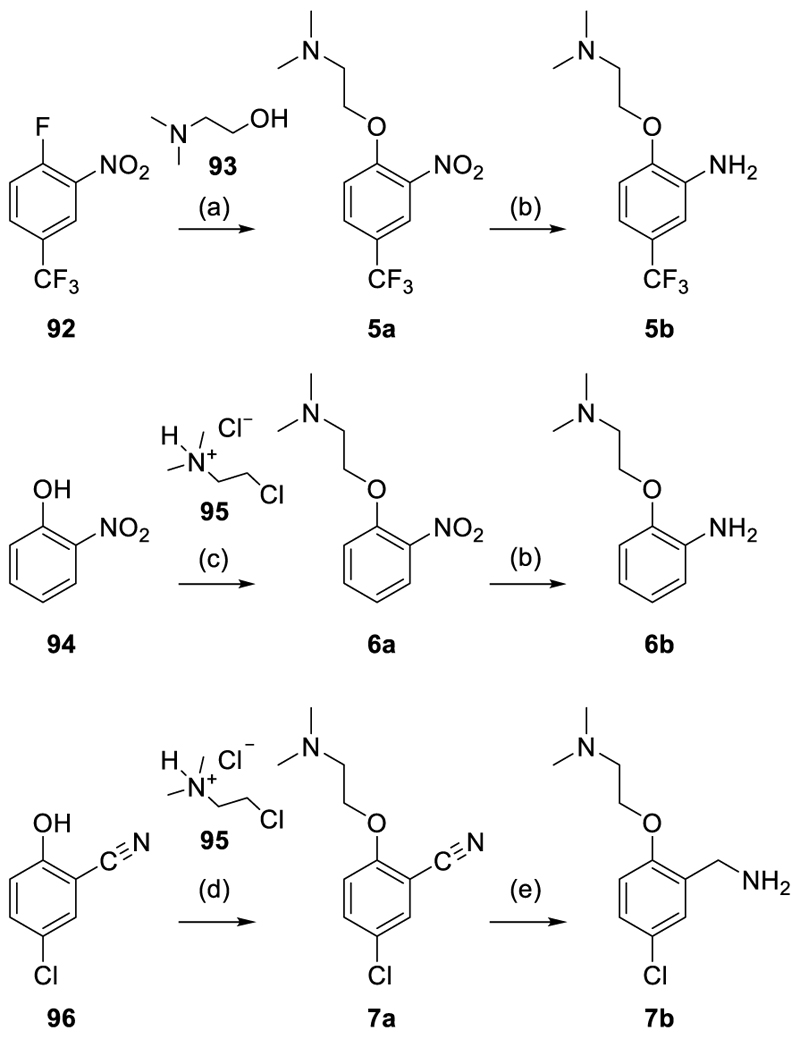
Synthesis of 49.^a^ ^a^ Reagents & Conditions: (a) LiAlH_4_, THF, 0°C, 2 h, 67%; (b) Dess–Martin-periodinane, CH_2_Cl_2_, DMF, 0°C to rt, 1.5 h, 61%; (c) NaBH(OAc)_3_, AcOH, DCM, rt, 2 h, 12%.

The secondary amine analogue **49** was prepared from the methyl ester precursor **49a** of **52** which was prepared as described previously^21^ and then reduced to the corresponding alcohol **49b** using LiAlH_4_. Oxidation of **49b** to the aldehyde **49c** with Dess–Martin periodinane (DMP) and subsequent reductive amination of **49c** with quinoline-3-amine (**82**) then afforded **49** (Scheme 2).

**Scheme 3:**
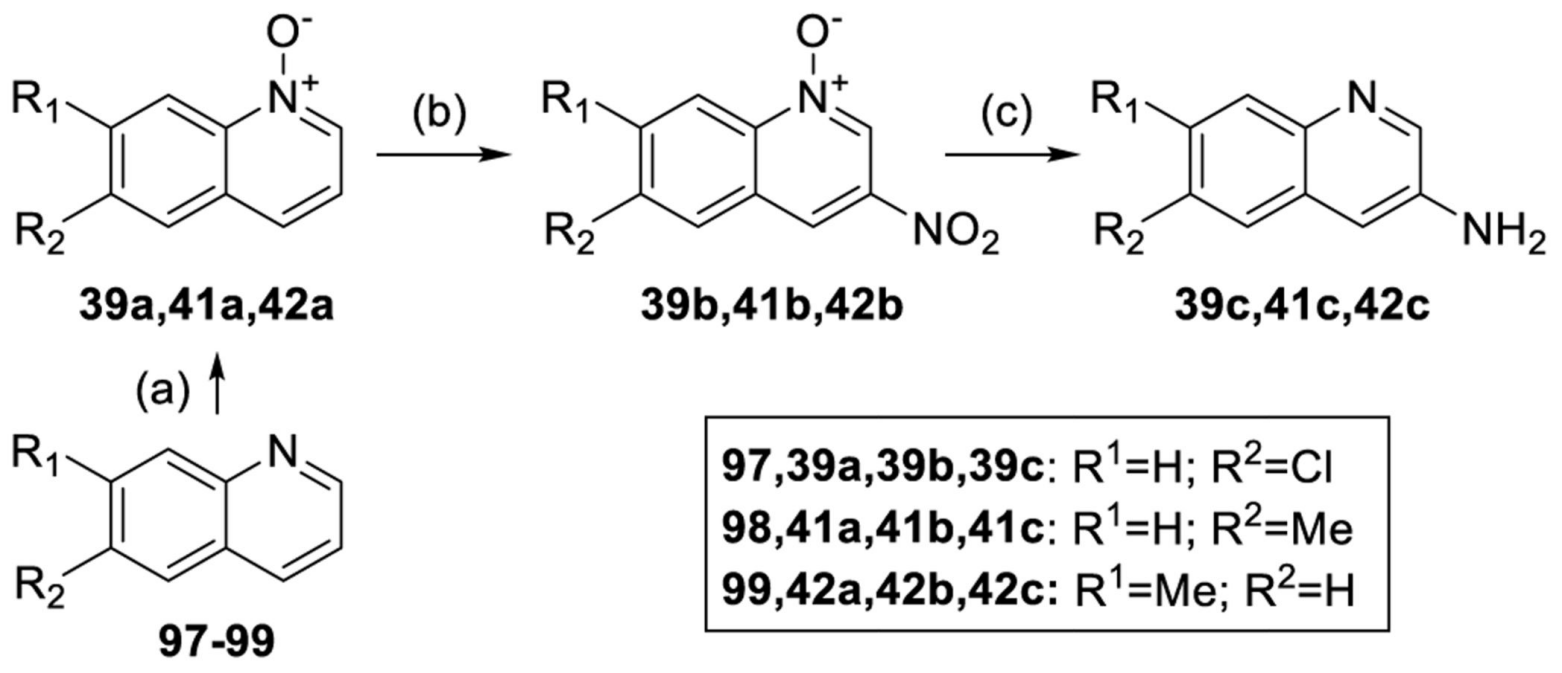
Synthesis of 5b-7b.^a^ ^a^ Reagents & Conditions: (a) Cs_2_CO_3_, DMF, 70°C, 2 h, 54%; (b) H_2_, Pd/C, MeOH, rt, 2-3 h, 99%; (c) KOH, EtOH, DMF, BuOH, reflux, overnight, 56%; (d) Cs_2_CO_3_, KI, THF, reflux, 16 h, 94%; (e) LiAlH_4_, THF, 0°C to rt, 44%.

**Scheme 4:**
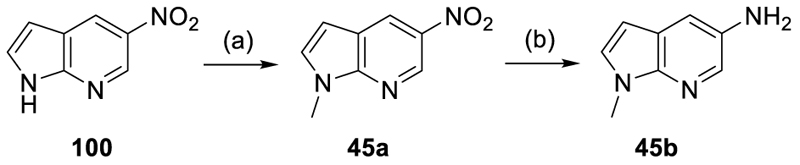
Synthesis of 39c, 41c and 42c.^a^ ^a^ Reagents & Conditions: (a) mCPBA, CH_2_Cl_2_, 0°C to rt, 24 h, 44-85%; (b) *tert*-butyl nitrite, MeCN, 100°C, 24 h, 30-47%; (c) NH_4_Cl, Fe powder, H_2_O, MeOH, 85°C, 2 h, 7-30%.

**Scheme 5:**
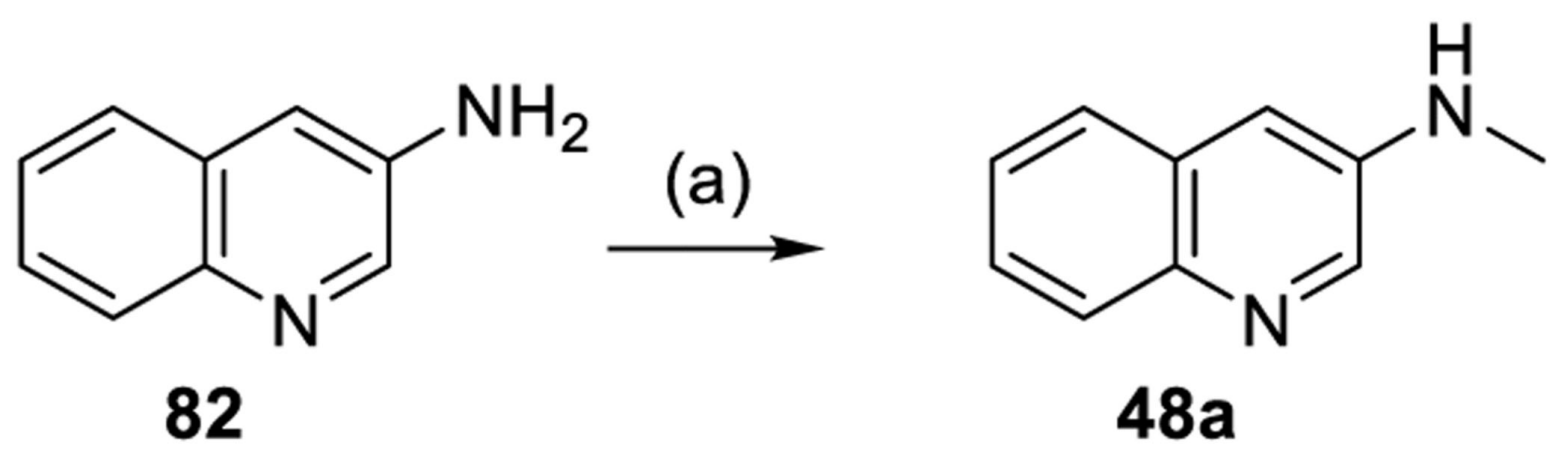
Synthesis of 45b.^a^ ^a^ Reagents & Conditions: (a) NaH, MeI, DMF, 0°C to rt, 4 h, 71%; (b) H_2_-atmosphere, Pd/C, EtOH, rt, 2 h, 42%.

**Scheme 6:**
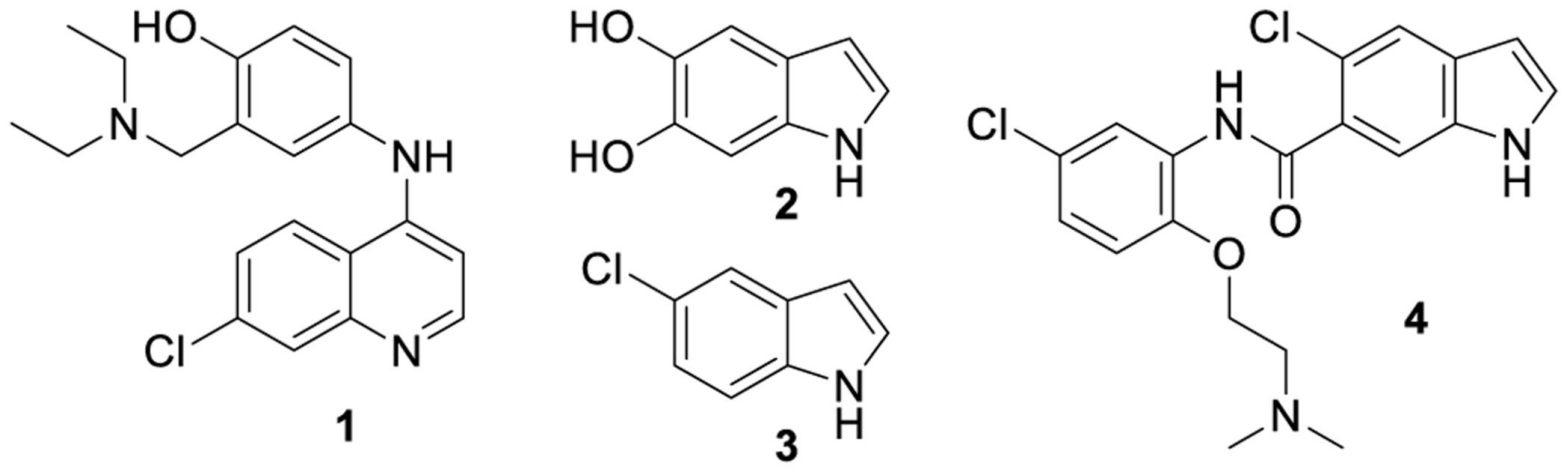
Synthesis of 48a.^a^ ^a^ Reagents & Conditions: (a) Triethyl orthoformate, TFA, 125°C, 12 h; then NaBH_4_, EtOH, rt, overnight, 49% over 2 steps.

Among the amines required for the synthesis of **5**-**51, 53-89** were commercially available, whereas **5b-7b, 39c, 41c-42c, 45b** and **48a** were prepared according to Schemes 3–6. Aniline **5b** was obtained via a nucleophilic aromatic substitution reaction between the fluorobenzene **92** and the alcohol **93** to **5a** and subsequent reduction of the nitro group in **5a** to **5b**. Similarly, nucleophilic substitution of phenol **94** and alkyl chloride **95** provided **6a** and subsequent reduction yielded aniline **6b**. For preparation of the benzyl amine **7b**, phenol **96** and alkyl chloride **95** were reacted under Finkelstein conditions and the resulting benzonitrile **7a** was subsequently reduced to **7b** using LiAlH_4_ (Scheme 3). The quinoline-3-amines **39c, 41c** and **42c** were prepared according to Scheme 4 by oxidizing the quinolines **97-99**, to the corresponding *N*-oxides **39a, 41a**, and **42a** using m-chloroperbenzoic acid (mCPBA) and subsequent selective radical nitration in 3-position using *tert*-butyl nitrite. The resulting 3-nitroquinoline-*N*-oxides **39b, 41b**, and **42b** were then reduced using Fe powder and NH_4_Cl to obtain **39c, 41c**, and **42c**.

Pyrrolo[2,3-*b*]pyridin-5-amine (**45b**) was obtained according to Scheme 5 by *N*-methylation of **100** with iodomethane and reduction of the resulting **45a** to **45b**. *N*-Methylquinoline-3-amine (**48a**) was obtained by a *N*-formylation of quinoline-3-amine (**82**) with triethyl orthoformate in TFA and subsequent reduction with sodium borohydride (NaBH_4_) in EtOH (Scheme 6).

## Experimental

### Chemistry

#### General

All chemicals were of reagent grade, purchased from commercial sources (e.g., Sigma-Aldrich, TCI, BLDpharm) and used without further purification. All reactions were conducted in absolute solvents purchased from Sigma-Aldrich. Other solvents, especially for work-up procedures, were of reagent grade or purified by distillation (*iso*hexane, cyclohexane, ethyl acetate, EtOH). Reactions were monitored by thin layer chromatography (TLC) on TLC Silica gel 60 F254 coated aluminum sheets by Merck and visualized under ultraviolet light (254 nm). Purification by column chromatography (CC) was performed on a puriFlash® X4420Plus system (Advion, Ithaca, NY, USA) using high performance spherical silica columns (SIHP, 50 μm) by Interchim and a gradient of *iso*hexane or cyclohexane to ethyl acetate, reversed-phase CC (RP-CC) was performed on a puriFlash® 5.250 system (Advion) using C18HP columns (SIHP, 15 μm) by Interchim. Mass spectra (MS) were obtained on a puriFlash®-CMS system (Advion) using atmospheric pressure chemical ionization (APCI). HRMS were obtained with a Thermo Finnigan LTQ FT instrument for electron impact ionization (EI) or electrospray ionization (ESI). NMR spectra were recorded on Bruker Avance III HD 400 MHz or 500 MHz spectrometers equipped with a CryoProbeTM Prodigy broadband probe (Bruker). Chemical shifts are reported in δ values (ppm) relative to residual protium signals in the NMR solvent (^1^H-NMR: Acetone-*d*_6_: δ = 2.04 ppm; DMSO-*d*_6_: δ = 2.50 ppm; MeOD-*d*_4_: δ = 3.31 ppm, ^13^C-NMR: Acetone-*d*_6_: δ = 206.26, 29.84 ppm; DMSO-*d*_6_: δ = 39.52 ppm; MeOD-*d*_4_: δ = 49.0 ppm), coupling constants (*J*) in hertz (Hz). The purity of compounds **5-47** and **49-51** was determined by ^1^H-NMR (qHNMR) according to the method described by Pauli *et al*.^40^: a sample of the analyte (1–4 mg) and an internal calibrant (1–4 mg maleic acid (Sigma Aldrich lot #BCCH2407)) were weighed into a 1.5 mL Eppendorf tube with ±0.01 mg accuracy. The mixture was treated with 600 μL of a deuterated solvent (Acetone-*d*_6_, DMSO-*d*_6_) and homogenized. The solution was transferred to a 5 mm NMR tube and the sample was submitted for measurement immediately. 2 dummy scans were performed prior to acquisition. Spectra were acquired at room temperature with 64 scans in a spectral range of 30 ppm (7.5–22.5 ppm relative to TMS). The relaxation time was set to 60 s to ensure complete relaxation, the acquisition time was set to 4 s. Recorded spectra were processed by applying zero-filling (256k), apodization (0.1 Hz exponential term), baseline correction (5th order Bernstein polynomial fit) and phase correction (manual). Purity was calculated by the following formula: ${\text{P}}_{A}[\text{\%}]=\frac{{\text{n}}_{IC}\cdot {\text{Int}}_{A}\cdot {\text{MW}}_{A}\cdot {\text{m}}_{IC}}{{\text{n}}_{A}\cdot {\text{Int}}_{IC}\cdot {\text{MW}}_{IC}\cdot {\text{m}}_{A}}\cdot {\text{P}}_{IC}$ where *A* denotes the analyte and *IC* denotes the internal calibrant. Integral ranges, masses of compounds and internal calibrants used for purity calculation are noted in the individual spectra. All compounds for biological testing had a purity >95% according to qHNMR. Purity of compound **48** was analyzed on a Shimadzu LCMS-2020 system. As stationary phase a Zorbax SBAq (3.5 μm, 100 mm x 3 mm, Agilent, protected with a 0.5 μm and a 0.2 μm frit) was used in combination with H_2_O (+ 0.1% FA) (A) and acetonitrile (+ 0.1% FA) (B) as mobile phase at a flow rate of 0.5 mL/min. **48** was investigated by injecting 10 μL sample solution under isocratic conditions (A/B 60:40, v/v). MS detection was done under negative ESI conditions recording corresponding [(M - H)]^-^ ion in the SIM mode (MS range 100 - 750 *m/z*). UV detection was done recording spectra at 254 nm.

#### General Procedure for Amide Coupling (GP1)

5-Chloro-1*H*-indole-6-carboxylic acid (**52**, 1.0 eq.) was dissolved in a mixture of MeCN (0.1 M) and dimethylformamide (DMF, 0.25 mL). The respective amine component (1.1–1.2 eq.) was added to the solution. Subsequently, 1-methylimidazole (NMI) (3.5 eq.) and *N*-[chloro(dimethylamino)methylene]-*N*-methylmethanaminium (TCFH, 1.1–1.2 eq.) were added in the specified order. The reaction mixture was stirred at rt for 24 h. After completion of the reaction (monitored by TLC), the solvent was removed under reduced pressure, and the crude product was purified by RP-CC (gradient elution from 5:95 MeCN/H_2_O to 100% MeCN).

#### General Procedure for *N*-Oxidation (GP2)

To a solution of the respective quinoline (1.0 eq.) in CH_2_Cl_2_ (0.1 M) was added m-chloroperbenzoic acid (mCPBA, 70%, 2.0 eq.) at 0°C. The reaction mixture was stirred at rt for 24 h. After completion (TLC), the mixture was diluted with CH_2_Cl_2_ (20 mL) and washed with saturated NaHCO_3_ solution (2 × 70 mL) and brine (70 mL). The organic layer was dried over anhydrous MgSO_4_, filtered, and concentrated under reduced pressure to yield the respective quinoline *N*-oxide.

#### General Procedure for Quinoline-*N*-oxide C3-Nitration (GP3)

The respective quinoline-*N*-oxide (1.0 eq.) was mixed with MeCN (0.1 M) and *tert*-butyl nitrite (3.5 eq.) and the reaction mixture was stirred at 100°C for 24 h. After cooling to rt, the mixture was poured into brine (20 mL) and extracted with EtOAc (3 × 20 mL). The combined organic layers were dried over anhydrous MgSO_4_, filtered, and concentrated under reduced pressure to yield the respective 3-nitroquinoline-*N*-oxide.

#### General Procedure for 3-Nitroquinoline-*N*-oxide Reduction to Quinoline-3-amines (GP4)

The respective 3-nitroquinoline-N-oxide (1.0 eq.) was dissolved in a mixture of MeOH and H_2_O (9:1, 0.1 M), ammonium chloride (NH_4_Cl, 7.0 eq.) was added to the solution and the mixture was sonicated under argon atmosphere for 15 min. Fe powder (10.0 eq.) was added, and the reaction was heated to 85°C for 2 h. After completion (TLC), the mixture was filtered through a pad of Celite®, concentrated to dryness, and purified by RP-CC (5:95 MeCN/H_2_0 to 100% MeCN) to obtain the respective quinoline-3-amine.

#### 5-Chloro-*N*-{2-[2-(dimethylamino)ethoxy]-5-(trifluoromethyl)phenyl}-1*H*-indole-6-carboxamid (5)

Preparation according to GP1 using **52** (41.6 mg, 0.213 mmol, 1.0 eq.), 2-[2-(dimethylamino)ethoxy]-5-(trifluoromethyl)aniline (**5b**, 76.3 mg, 0.307 mmol, 1.2 eq.), NMI (85.7 μL, 1.08 mmol, 3.5 eq.) and TCFH (86.2 mg, 0.307 mmol, 1.2 eq.) yielded compound **5** (10.3 mg, 5.5%) as a colorless solid. ^1^H NMR (500 MHz, DMSO-*d*_6_): δ = 11.59–11.39 (m, 1H), 10.31 (s, 1H), 8.65–8.50 (m, 1H), 7.78–7.68 (m, 2H), 7.59–7.54 (m, 1H), 7.52–7.46 (m, 1H), 7.39–7.34 (m, 1H), 6.53–6.48 (m, 1H), 4.20 (t, *J* = 5.5 Hz, 2H), 2.53 (t, *J* = 5.6 Hz, 2H), 1.98 (s, 6H) ppm. ^13^C NMR (126 MHz, DMSO-*d*_6_): δ = 166.1, 151.6, 133.8, 130.1, 129.8, 129.4, 128.1, 124.4 (q, J = 271.8 Hz), 122.3 (q, J = 32.3 Hz), 121.8 (q, J = 3.8 Hz), 120.8, 120.1, 118.2–117.3 (m), 116.1, 112.9, 101.2, 68.6, 57.2, 44.8 (2 C) ppm. qHNMR (400 MHz, acetone-*d*_6_, maleic acid as reference): purity = 96.1%. HRMS (ESI+): *m/z* calculated 426.1196 for C_20_H_20_ClF_3_N_3_O_2_, found 426.1193 ([M+H]^+^).

#### 5-Chloro-*N*-{2-[2-(dimethylamino)ethoxy]phenyl}-1*H*-indole-6-carboxamide (6)

Preparation according to GP1 using **52** (41.6 mg, 0.213 mmol, 1.0 eq.), 2-[2-(dimethylamino)ethoxy]aniline (**6b**, 40.5 mg, 0.225 mmol, 1.1 eq.), NMI (57.1 μL, 0.716 mmol, 3.5 eq.) and TCFH (63.1 mg, 0.225 mmol, 1.1 eq.) yielded compound **6** (10.3 mg, 14.1%) as a colorless solid. ^1^H NMR (500 MHz, DMSO-*d*_6_): δ = 11.67–11.29 (m, 1H), 10.27 (s, 1H), 8.34–8.09 (m, 1H), 7.75–7.63 (m, 2H), 7.62–7.45 (m, 1H), 7.29–6.97 (m, 3H), 6.61–6.42 (m, 1H), 4.09 (t, *J* = 5.4 Hz, 2H), 2.44 (t, *J* = 5.4 Hz, 2H), 1.88 (s, 6H) ppm. ^13^C NMR (126 MHz DMSO-*d*_6_): δ = 166.1, 149.2, 134.2, 130.7, 130.0, 129.6, 129.3, 125.1, 122.7, 121.8, 121.1, 120.9, 117.4, 112.9, 101.5, 69.3, 57.7, 50.0 (2 C) ppm. qHNMR (400 MHz, acetone-*d*_6_, maleic acid as reference): purity = 97.3%. HRMS (ESI+): *m/z* calculated 358.1322 for C_19_H_21_ClN_3_O_2_, found 358.1315 ([M+H]^+^).

#### 5-Chloro-*N*-{5-chloro-2-[2-(dimethylamino)ethoxy]benzyl}-1*H*-indole-6-carboxamide (7)

Preparation according to GP1 using **52** (47.4 mg, 0.242 mmol, 1.0 eq.), 2-[2-(aminomethyl)-4-chlorophenoxy]-*N*,*N*-dimethylethan-1-amine (**7b**, 61.0 mg, 0.267 mmol, 1.1 eq.), NMI (67.6 μL, 0.849 mmol, 3.5 eq.) and TCFH (74.8 mg, 0.267 mmol, 1.1 eq.) yielded compound **7** (16.0 mg, 16%) as a colorless solid. ^1^H NMR (400 MHz, acetone-*d*_6_): δ = 10.64–10.49 (m, 1H), 8.18 (s, 1H), 7.64–7.60 (m, 1H), 7.60–7.55 (m, 1H), 7.51–7.45 (m, 1H), 7.45–7.40 (m, 1H), 7.29–7.22 (m, 1H), 7.06–7.00 (m, 1H), 6.52–6.46 (m, 1H), 4.57 (d, *J* = 6.0 Hz, 2H), 4.15 (t, *J* = 5.3 Hz, 2H), 2.64 (t, *J* = 5.3 Hz, 2H), 1.94 (s, 6H). ppm. ^13^C NMR (101 MHz, acetone-*d*_6_): δ = 168.3, 156.8, 135.1, 131.2, 131.1, 130.6, 129.7, 129.0, 128.8, 125.7, 122.2, 121.5, 114.5, 113.0, 102.1, 67.1, 59.1, 45.5, 39.8 (2 C) ppm. qHNMR (400 MHz, acetone-*d*_6_, maleic acid as reference): purity = 96.8%. HRMS (EI+): *m/z* calculated 405.1011 for C_20_H_21_Cl_2_N_3_O_2_, found 405.0991 ([M]^+^).

#### 5-Chloro-*N*-(5-chloro-2-methoxyphenyl)-1*H*-indole-6-carboxamide (8)

Preparation according to GP1 using **52** (50.0 mg, 0.256 mmol, 1.0 eq.), 5-chloro-2-methoxyaniline (**53**, 44.3 mg, 0.281 mmol, 1.1 eq.), NMI (71.3 μL, 0.895 mmol, 3.5 eq.) and TCFH (78.9 mg, 0.281 mmol, 1.1 eq.) yielded compound **8** (22.3 mg; yield: 26%) as a colorless solid. ^1^H NMR (500 MHz, acetone-*d*_6_): δ = 10.86–10.60 (m, 1H), 9.07 (s, 1H), 8.68–8.60 (m, 1H), 7.93–7.89 (m, 1H), 7.73–7.70 (m, 1H), 7.61–7.56 (m, 1H), 7.18–7.02 (m, 2H), 6.58–6.53 (m, 1H), 3.92 (s, 3H) ppm. ^13^C NMR (126 MHz, acetone-*d*_6_): δ = 166.2, 148.4, 135.2, 131.5, 130.3, 130.0, 129.1, 126.0, 124.0, 122.1, 121.7, 120.2, 114.4, 112.7, 102.3, 56.8 ppm. qHNMR (400 MHz, DMSO-*d*_6_, maleic acid as reference): purity = 95.5%. HRMS (EI+): *m/z* calculated 334.0276 for C_16_H_12_Cl_2_N_2_O_2_, found 334.0269 ([M]^+^).

#### 5-Chloro-*N*-(3-chloro-5-methoxyphenyl)-1*H*-indole-6-carboxamide (9)

Preparation according to GP1 using **52** (30.0 mg, 0.153 mmol, 1.0 eq.), 3-chloro-5-methoxyaniline (**54**, 29.0 mg, 0.184 mmol, 1.2 eq.), NMI (42.8 μL, 0.537 mmol, 3.5 eq.) and TCFH (51.6 mg, 0.184 mmol, 1.2 eq.) yielded compound **9** (16.0 mg, yield: 31%) as a colorless solid. ^1^H-NMR (400 MHz, DMSO-*d*_6_): δ = 11.53–11.48 (m, 1H), 10.52 (s, 1H), 7.73–7.69 (m, 1H), 7.63–7.58 (m, 1H), 7.58–7.53 (m, 1H), 7.51–7.47 (m, 1H), 7.34–7.29 (m, 1H), 6.80–6.74 (m, 1H), 6.53–6.47 (m, 1H), 3.77 (s, 3H) ppm. ^13^C NMR (101 MHz, DMSO-*d*_6_): δ = 166.4, 160.4, 141.4, 133.8, 133.6, 129.5, 129.1, 128.9, 120.6, 120.4, 112.2, 111.5, 109.0, 104.0, 101.1, 55.6 ppm. qHNMR (400 MHz, acetone-*d*_6_, maleic acid as reference): purity = 99.3%. HRMS (ESI+): *m/z* calculated 335.0354 for C_16_H_13_Cl_2_N_2_O_2_, found 335.0342 ([M+H]^+^).

#### 5-Chloro-*N*-(3-chlorophenyl)-1*H*-indole-6-carboxamide (10)

Preparation according to GP1 using **52** (50.0 mg, 0.256 mmol, 1.0 eq.), 3-chloroaniline (**55**, 35.9 mg, 0.281 mmol, 1.1 eq.), NMI (61.1 μL, 0.895 mmol, 3.5 eq.) and TCFH (78.9 mg, 0.281 mmol, 1.1 eq.) yielded compound **10** (28.1 mg, yield: 36%) as a colorless solid. ^1^H-NMR (500 MHz, DMSO-*d*_6_): δ = 11.52–11.49 (m, 1H), 10.57 (s, 1H), 7.97–7.92 (m, 1H), 7.71 (s, 1H), 7.64–7.58 (m, 2H), 7.58-7.53 (m, 1H), 7.41–7.34 (m, 1H), 7.19–7.13 (m, 1H), 6.52–6.47 (m, 1H) ppm. ^13^C NMR (126 MHz, DMSO-*d*_6_): δ = 166.4, 1408, 133.6, 133.2, 130.6, 129.5, 129.1, 128.9, 123.3, 120.6, 120.5, 118.9, 118.0, 112.2, 101.1 ppm. qHNMR (400 MHz, acetone-*d*_6_, maleic acid as reference): purity = 95.0%. HRMS (ESI+): *m/z* calculated 305.0249 for C_15_H_11_Cl_2_N_2_O, found 305.0242 ([M+H]^+^).

#### 5-Chloro-*N*-(2-chlorophenyl)-1*H*-indole-6-carboxamide (11)

Preparation according to GP1 using **52** (75.0 mg, 0.383 mmol, 1.0 eq.), 2-chloroaniline (**56**, 58.7 mg, 0.460 mmol, 1.2 eq.), NMI (107 μL, 1.34 mmol, 3.5 eq.) and TCFH (129 mg, 0.460 mmol, 1.2 eq.) yielded compound **11** (7.0 mg, yield: 6.0%) as a colorless solid. ^1^H-NMR (500 MHz, acetone-*d*_6_): δ = 10.81–10.67 (m, 1H), 9.03 (s, 1H), 8.44–8.39 (m, 1H), 7.93–7.90 (m, 1H), 7.75–7.72 (m, 1H), 7.62–7.57 (m, 1H), 7.56–7.50 (m, 1H), 7.46–7.38 (m, 1H), 7.25–7.18 (m, 1H), 6.60–6.55 (m, 1H) ppm. ^13^C NMR (126 MHz, acetone-*d*_6_): δ = 166.5, 136.4, 135.2, 131.4, 130.2, 129.9, 129.4, 128.5, 126.3, 125.3, 124.3, 122.0, 121.9, 114.2, 102.3 ppm. qHNMR (400 MHz, acetone-*d*_6_, maleic acid as reference): purity = 95.6%. HRMS (EI+): *m/z* calculated 304.0170 for C_15_H_10_Cl_2_N_2_O, found 304.0163 ([M]^+^).

#### 5-Chloro-*N*-(4-chlorophenyl)-1*H*-indole-6-carboxamide (12)

Preparation according to GP1 using **52** (90.3 mg, 0.462 mmol, 1.0 eq.), 4-chloroaniline (**57**, 70.7 mg, 0.554 mmol, 1.2 eq.), NMI (129 μL, 1.62 mmol, 3.5 eq.) and TCFH (155 mg, 0.554 mmol, 1.2 eq.) yielded compound **12** (13.3 mg, yield: 9.4%) as a colorless solid. ^1^NMR (500 MHz, DMSO-*d*_6_): δ = 11.52–11.48 (m, 1H), 10.53 (s, 1H), 7.84–7.76 (m, 2H), 7.75–7.69 (m, 1H), 7.63–7.60 (m, 1H), 7.58–7.54 (m, 1H), 7.48–7.32 (m, 2H), 6.55–6.46 (m, 1H) ppm. ^13^C NMR (126 MHz, DMSO-*d*_6_): δ = 166.1, 138.3, 133.6, 129.4, 129.0, 129.0, 128.6 (2 C), 127.0, 121.0 (2 C), 120.5, 120.5, 112.1, 101.0 ppm. qHNMR (400 MHz, DMSO-*d*_6_, maleic acid as reference): purity = 95.7%. HRMS (EI+): *m/z* calculated 304.0170 for C_15_H_10_Cl_2_N_2_O, found 304.0163 ([M]^+^).

#### 5-Chloro-*N*-(*o*-tolyl)-1*H*-indole-6-carboxamide (13)

Preparation according to GP1 using **52** (50.0 mg, 0.256 mmol, 1.0 eq.), *o*-toluidine (**58**, 30.1 mg, 0.281 mmol, 1.1 eq.), NMI (71.3 μL, 0.895 mmol, 3.5 eq.) and TCFH (86.1 mg, 0.307 mmol, 1.2 eq.) yielded compound **13** (33.6 mg, yield: 46%) as a colorless solid. ^1^NMR (500 MHz, DMSO-*d*_6_): δ = 11.47–11.43 (m, 1H), 9.82 (s, 1H), 7.71–7.68 (m, 1H), 7.67–7.63 (m, 1H), 7.56–7.51 (m, 1H), 7.47–7.42 (m, 1H), 7.29–7.19 (m, 2H), 7.18–7.12 (m, 1H), 6.53–6.44 (m, 1H), 2.31 (s, 3H) ppm. 13C NMR (101 MHz, DMSO-*d*_6_): δ = 166.6, 136.7, 134.1, 133.4, 130.8, 129.8, 129.7, 129.2, 126.4, 126.4, 126.2, 121.0, 120.9, 112.6, 101.4, 18.5 ppm. qHNMR (400 MHz, acetone-*d*_6_, maleic acid as reference): purity = 98.7%. HRMS (EI+): *m/z* calculated 284.0716 for C_16_H_13_ClN_2_O, found 284.0709 ([M]^+^).

#### 5-Chloro-*N*-(*m*-tolyl)-1*H*-indole-6-carboxamide (14)

Preparation according to GP1 using **52** (63.1 mg, 0.332 mmol, 1.0 eq.), *m*-toluidine (**59**, 38.0 mg, 0.355 mmol, 1.1 eq.), NMI (89.9 μL, 1.13 mmol, 3.5 eq.) and TCFH (109 mg, 0.387 mmol, 1.2 eq.) yielded compound **14** (33.6 mg, yield: 46%) as a colorless solid. ^1^H NMR (500 MHz, DMSO-*d*_6_): δ = 11.70–11.40 (m, 1H), 10.29 (s, 1H), 7.72–7.66 (m, 1H), 7.63–7.47 (m, 4H), 7.27–7.13 (m, 1H), 7.00–6.87 (m, 1H), 6.66-6.37 (m, 1H), 2.30 (s, 3H) ppm. ^13^C NMR (126 MHz, DMSO-*d*_6_): δ = 166.4, 139.8, 138.4, 134.1, 129.9, 129.7, 129.3, 129.0, 124.7, 121.0, 120.9, 120.5, 117.2, 112.5, 101.4, 21.7 ppm. qHNMR (400 MHz, acetone-*d*_6_, maleic acid as reference): purity = 98.0%. HRMS (EI+): *m/z* calculated 284.0716 for C_16_H_13_ClN_2_O, found 284.0709 ([M]^+^).

#### 5-Chloro-*N*-(*p*-tolyl)-1*H*-indole-6-carboxamide (15)

Preparation according to GP1 using **52** (50.0 mg, 0.256 mmol, 1.0 eq.), *p*-toluidine (**60**, 32.9 mg, 0.307 mmol, 1.2 eq.), NMI (71.3 μL, 0.895 mmol, 3.5 eq.) and TCFH (86.1 mg, 0.307 mmol, 1.2 eq.) yielded compound **15** (68.0 mg, yield: 93%) as a colorless solid. ^1^H NMR (400 MHz, MeOD): δ 7.68–7.64 (m, 1H), 7.64–7.60 (m, 1H), 7.60–7.50 (m, 2H), 7.45–7.38 (m, 1H), 7.21–7.14 (m, 2H), 6.52–6.46 (m, 1H), 2.33 (s, 3H). ppm. ^13^C NMR (101 MHz, MeOD): δ = 169.0, 136.7, 134.8, 134.6, 130.9, 129.9, 129.7 (2 C), 128.7, 121.8, 121.4, 121.0 (2 C), 112.3, 101.7, 20.3 ppm. qHNMR (400 MHz, acetone-*d*_6_, maleic acid as reference): purity = 95.7%. HRMS (EI+): *m/z* calculated 284.0716 for C_16_H_13_ClN_2_O, found 284.0710 ([M]^+^).

#### 5-Chloro-*N*-(3-fluorophenyl)-1*H*-indole-6-carboxamide (16)

Preparation according to GP1, using **52** (50.0 mg, 0.256 mmol, 1.0 eq.), 3-fluoroaniline (**61**, 31.9 mg, 0.281 mmol, 1.1 eq.), NMI (71.3 μL, 0.895 mmol, 3.5 eq.) and TCFH (78.9 mg, 0.281 mmol, 1.1 eq.) yielded compound **16** (22.0 mg, yield: 30%) as a colourless solid. ^1^H NMR (500 MHz, DMSO-*d*_6_): δ = 11.57–11.46 (m, 1H), 10.59 (s, 1H), 7.75–7.68 (m, 2H), 7.63–7.60 (m, 1H), 7.57–7.53 (m, 1H), 7.50 –7.45 (m, 1H), 7.42–7.34 (m, 1H), 6.96–6.89 (m, 1H), 6.52–6.45 (m, 1H) ppm. ^13^C NMR (126 MHz, DMSO-*d*_6_): δ 166.4, 162.2 (d, J = 241.2 Hz), 141.1 (d, J = 11.1 Hz), 133.7, 130.5 (d, J = 9.4 Hz), 129.5, 129.1, 129.0, 120.6, 120.5, 115.3, 112.2, 110.1 (d, J = 21.1 Hz), 106.3 (d, J = 26.2 Hz), 101.1.ppm. qHNMR (400 MHz, DMSO-*d*_6_, maleic acid as reference): purity = 96.5%. HRMS (ESI-): *m/z* calculated for C_15_H_9_ClFN_2_O ([M-H]^-^) 287.0387, found 287.0393.

#### 5-Chloro-*N*-(4-fluorophenyl)-1*H*-indole-6-carboxamide (17)

Preparation according to GP1, using **52** (50.0 mg, 0.256 mmol, 1.0 eq.), 4-fluoroaniline (**62**, 31.9 mg, 0.281 mmol, 1.1 eq.), NMI (71.3 μL, 0.895 mmol, 3.5 eq.) and TCFH (78.9 mg, 0.281 mmol, 1.1 eq.) yielded compound **17** (24.4 mg, yield: 30%) as a colourless solid. ^1^H NMR (500 MHz, DMSO-*d*_6_): δ = 11.80–11.20 (m, 1H), 10.43 (s, 1H), 7.86–7.73 (m, 2H), 7.73–7.66 (m, 1H), 7.61–7.57 (m, 1H), 7.56–7.51 (m, 1H), 7.23–7.14 (m, 2H), 6.56–6.41 (m, 1H). ppm. ^13^C NMR (126 MHz, DMSO-*d*_6_): δ = 166.0, 158.2 (d, J = 240.1 Hz), 135.8 (d, J = 2.6 Hz), 133.7, 129.4, 129.3, 129.0, 121.3 (d, J = 7.6 Hz, 2 C), 120.6 (2 C), 115.4 (d, J = 22.1 Hz, 2 C), 112.2, 101.1.ppm. qHNMR (400 MHz, DMSO-*d*_6_, maleic acid as reference): purity = 96.0%. HRMS (ESI-): *m/z* calculated for C_15_H_9_ClFN_2_O ([M-H]^-^) 287.0387, found 287.0393.

#### 5-Chloro-*N*-phenyl-1*H*-indole-6-carboxamide (18)

Preparation according to GP1 using **52** (50.0 mg, 0.256 mmol, 1.0 eq.), aniline (**63**, 26.32 mg, 0.281 mmol, 1.1 eq.), NMI (71.3 μL, 0.895 mmol, 3.5 eq.) and TCFH (78.9 mg, 0.281 mmol, 1.1 eq.) yielded compound **18** (18.2 mg, yield: 26%) as a colourless solid. ^1^H NMR (500 MHz, DMSO-*d*_6_): δ = 11.71–11.29 (m, 1H), 10.37 (s, 1H), 7.82–7.67 (m, 3H), 7.61–7.58 (m, 1H), 7.56–7.52 (m, 1H), 7.38–7.31 (m, 2H), 7.12–7.06 (m, 1H), 6.51–6.47 (m, 1H) ppm. ^13^C NMR (126 MHz, DMSO-*d*_6_): δ = 166.1, 139.4, 133.7, 129.4, 129.4, 128.9, 128.8 (2 C), 123.6, 120.6, 120.5, 119.6 (2 C), 112.1, 101.1 ppm. qHNMR (400 MHz, acetone-*d*_6_, maleic acid as reference): purity = 99.4%. HRMS (EI+): *m/z* calculated 270.0560 for C_15_H_11_ClN_2_O, found 270.0553 ([M]^+^).

#### 5-Chloro-*N*-[3-(trifluoromethyl)phenyl]-1*H*-indole-6-carboxamide (19)

Preparation according to GP1 using **52** (50.0 mg, 0.256 mmol, 1.0 eq.), 3-(trifluoromethyl)aniline (**64**, 45.3 mg, 0.281 mmol, 1.1 eq.), NMI (71.3 μL, 0.895 mmol, 3.5 eq.) and TCFH (78.9 mg, 0.281 mmol, 1.1 eq.) yielded compound **19** (35.0 mg, yield: 40%) as a colorless solid. ^1^H NMR (500 MHz, DMSO-*d*_6_): δ = 11.53–11.50 (m, 1H), 10.72 (s, 1H), 8.27–8.23 (m, 1H), 7.95–7.90 (m, 1H), 7.73–7.70 (m, 1H), 7.66–7.62 (m, 1H), 7.61–7.57 (m, 1H), 7.57–7.54 (m, 1H), 7.47–7.42 (m, 1H), 6.52–6.47 (m, 1H). ppm. ^13^C NMR (126 MHz, DMSO-*d*_6_): δ = 166.5, 140.1, 133.6, 130.0, 129.5, 129.5 (q, *J* = 31.6 Hz), 129.1, 128.7, 126.3 (q, *J* = 272.2 Hz), 123.1, 120.6, 120.4, 119.9 (q, *J* = 4.0 Hz), 115.5 (q, *J* = 4.1 Hz), 112.2, 101.1 ppm. qHNMR (400 MHz, acetone-*d*_6_, maleic acid as reference): purity = 99.4%. HRMS (EI+): *m/z* calculated 338.0433 for C_16_H_10_ClF_3_N_2_O, found 338.0430 ([M]^+^).

#### 5-Chloro-*N*-[4-(trifluoromethyl)phenyl]-1*H*-indole-6-carboxamide (20)

Preparation according to GP1 using **52** (50.0 mg, 0.256 mmol, 1.0 eq.), 4-(trifluoromethyl)aniline (**65**, 45.3 mg, 0.281 mmol, 1.1 eq.), NMI (71.3 μL, 0.895 mmol, 3.5 eq.) and TCFH (78.9 mg, 0.281 mmol, 1.1 eq.) yielded compound **20** (32.1 mg, yield: 37%) as a colorless solid. ^1^H NMR (500 MHz, DMSO-*d*_6_): δ = 11.56–11.44 (m, 1H), 10.78 (s, 1H), 8.02–7.90 (m, 2H), 7.78–7.70 (m, 3H), 7.66–7.63 (m, 1H), 7.59–7.55 (m, 1H), 6.53–6.48 (m, 1H) ppm. ^13^C NMR (126 MHz, DMSO-*d*_6_): δ = 166.5, 142.9, 133.6, 129.6, 129.2, 128.8, 126.6 (q, *J* = 271.3 Hz), 126.2 (q, *J* = 3.9 Hz, 2 C), 123.5 (q, *J* = 32.4 Hz), 120.6, 120.5, 119.4 (2 C), 112.3, 101.1 ppm. qHNMR (400 MHz, DMSO-*d*_6_, maleic acid as reference): purity = 95.2%. HRMS (ESI+): *m/z* calculated 339.0512 for C_16_H_11_ClF_3_N_2_O, found 339.0509 ([M+H]^+^).

#### *N*-([1,1’-Biphenyl]-3-yl)-5-chloro-1*H*-indole-6-carboxamide (21)

Preparation according to GP1 using **52** (50.0 mg, 0.256 mmol, 1.0 eq.), [1,1’-biphenyl]-3-amine (**66**, 48.1 mg, 0.281 mmol, 1.1 eq.), NMI (71.3 μL, 0.895 mmol, 3.5 eq.) and TCFH (78.9 mg, 0.281 mmol, 1.1 eq.) yielded compound **21** (35.0 mg, yield: 40%) as a colorless solid. ^1^H NMR (400 MHz, acetone-*d*_6_): δ = 10.69–10.65 (m, 1H), 9.48–9.44 (m, 1H), 8.20 (s, 1H), 7.89–7.83 (m, 1H), 7.77–7.72 (m, 1H), 7.71–7.64 (m, 3H), 7.56–7.53 (m, 1H), 7.51–7.34 (m, 5H), 6.57–6.51 (m, 1H) ppm. ^13^C NMR (101 MHz, acetone-*d*_6_): δ = 167.0, 142.6, 141.8, 141.1, 135.1, 131.0, 130.8, 130.2, 129.8 (2 C), 129.4, 128.4, 127.8 (2 C), 123.0, 122.2, 121.7, 119.3, 118.9, 113.2, 102.3 ppm. qHNMR (400 MHz, acetone-*d*_6_, maleic acid as reference): purity = 97.4%. HRMS (ESI+): *m/z* calculated 347.0951 for C_21_H_16_ClN_2_O, found 347.0942 ([M+H]^+^).

#### *N*-([1,1’-Biphenyl]-4-yl)-5-chloro-1*H*-indole-6-carboxamide (22)

Preparation according to GP1 using **52** (50.0 mg, 0.256 mmol, 1.0 eq.), [1,1’-biphenyl]-4-amine (**67**, 47.6 mg, 0.281 mmol, 1.1 eq.), NMI (71.3 μL, 0.895 mmol, 3.5 eq.) and TCFH (78.9 mg, 0.281 mmol, 1.1 eq.) yielded compound **22** (50.9 mg, yield: 57%) as a colorless solid. ^1^H NMR (500 MHz, DMSO-*d*_6_): δ = 11.61–11.42 (m, 1H), 10.48 (s, 1H), 7.87–7.82 (m, 2H), 7.73–7.70 (m, 1H), 7.69–7.64 (m, 4H), 7.64–7.61 (m, 1H), 7.57–7.53 (m, 1H), 7.49–7.42 (m, 2H), 7.37–7.31 (m, 1H), 6.53–6.48 (m, 1H) ppm. ^13^C NMR (126 MHz, DMSO-*d*_6_): δ 166.2, 139.8, 138.9, 135.3, 133.7, 129.4, 129.4, 129.0 (2 C), 129.0, 127.2, 127.0 (2 C), 126.4 (2 C), 120.6, 120.6, 119.9 (2 C), 112.2, 101.1 ppm. qHNMR (400 MHz, DMSO-*d*_6_, maleic acid as reference): purity = 98.4%. HRMS (ESI+): *m/z* calculated 347.0951 for C_21_H_16_ClN_2_O, found 347.0944 ([M+H]^+^).

#### *N*-[3-(Tert-butyl)phenyl]-5-chloro-1*H*-indole-6-carboxamide (23)

Preparation according to GP1 using **52** (40.0 mg, 0.204 mmol, 1.0 eq.), 3-(*tert*-butyl)aniline (**68**, 33.6 mg, 0.225 mmol, 1.1 eq.), NMI (57.1 μL, 0.716 mmol, 3.5 eq.) and TCFH (68.9 mg, 0.245 mmol, 1.2 eq.) yielded compound **23** (55.0 mg, yield: 82%) as a colorless solid. ^1^H NMR (400 MHz, DMSO-*d*_6_): δ = 11.49–11.45 (m, 1H), 10.30 (s, 1H), 7.80–7.74 (m, 1H), 7.69 (s, 1H), 7.66–7.57 (m, 2H), 7.57–7.51 (m, 1H), 7.31–7.22 (m, 1H), 7.16–7.09 (m, 1H), 6.52–6.46 (m, 1H), 1.28 (s, 9H) ppm. ^13^C NMR (101 MHz, DMSO-*d*_6_): δ = 165.9, 151.2, 139.2, 133.6, 129.5, 129.2, 128.8, 128.3, 120.5, 120.5, 120.4, 116.6, 116.4, 112.0, 100.9, 34.4, 31.1 ppm. qHNMR (400 MHz, acetone-*d*_6_, maleic acid as reference): purity = 98.9%. HRMS (EI+): *m/z* calculated 326.1186 for C_19_H_19_ClN_2_O, found 326.1178 ([M]^+^).

#### *N*-[4-(Tert-butyl)phenyl]-5-chloro-1*H*-indole-6-carboxamide (24)

Preparation according to GP1 using **52** (50.0 mg, 0.256 mmol, 1.0 eq.), 4-(*tert*-butyl)aniline (**69**, 42.0 mg, 0.281 mmol, 1.1 eq.), NMI (71.3 μL, 0.895 mmol, 3.5 eq.) and TCFH (78.9 mg, 0.281 mmol, 1.1 eq.) yielded compound **24** (65.1 mg, yield: 78%) as a colorless solid. ^1^H NMR (400 MHz, acetone-*d*_6_): δ = 10.91–10.39 (m, 1H), 9.42–9.05 (m, 1H), 7.80–7.73 (m, 2H), 7.71–7.64 (m, 2H), 7.56–7.50 (m, 1H), 7.45–7.36 (m, 2H), 6.56–6.50 (m, 1H), 1.33 (s, 9H) ppm. ^13^C NMR (126 MHz, acetone-*d*_6_): δ = 166.3, 146.7, 137.5, 134.6, 130.6, 130.4, 128.8, 125.9 (2 C), 121.7, 121.2, 119.7 (2 C), 112.7, 101.7, 34.4, 31.3 ppm. qHNMR (400 MHz, acetone-*d*_6_, maleic acid as reference): purity = 99.7%. HRMS (ESI+): *m/z* calculated 327.1264 for C_19_H_20_ClN_2_O, found 327.1254 ([M+H]^+^).

#### 5-Chloro-*N*-(3-methoxyphenyl)-1*H*-indole-6-carboxamide (25)

Preparation according to GP1 using **52** (40.0 mg, 0.204 mmol, 1.0 eq), 3-methoxyaniline (**70**, 22.7 mg, 0.225 mmol, 1.1 eq.), NMI (57.1 μL, 0.716 mmol, 3.5 eq.) and TCFH (68.9 mg, 0.245 mmol, 1.2 eq.) yielded compound **25** (60.1 mg, yield: 98%) as a colorless solid. ^1^H NMR (500 MHz, DMSO-*d*_6_): δ =11.49–11.46 (m, 1H), 10.35 (s, 1H), 7.71–7.68 (m, 1H), 7.61–7.57 (m, 1H), 7.56–7.52 (m, 1H), 7.47–7.42 (m, 1H), 7.33–7.20 (m, 2H), 6.71–6.65 (m, 1H), 6.51–6.47 (m, 1H), 3.74 (s, 3H) ppm. ^13^C NMR (126 MHz, DMSO-*d*_6_): δ = 166.1, 159.6, 140.6, 133.7, 129.6, 129.4 (2 C), 128.9, 120.6, 120.5, 112.1, 111.9, 109.0, 105.3, 101.1, 55.1 ppm. qHNMR (400 MHz, acetone-*d*_6_, maleic acid as reference): purity = 96.8%. HRMS (EI+): *m/z* calculated 300.0666 for C_16_H_13_ClN_2_O_2_, found 300.0659 ([M]^+^).

#### 5-Chloro-*N*-(4-methoxyphenyl)-1*H*-indole-6-carboxamide (26)

Preparation according to GP1 using **52** (50.0 mg, 0.256 mmol, 1.0 eq.), 4-methoxyaniline (**71**, 37.8 mg, 0.281 mmol, 1.1 eq.), NMI (71.3 μL, 0.895 mmol, 3.5 eq.) and TCFH (78.9 mg, 0.281 mmol, 1.1 eq.) yielded compound **26** (24.0 mg, yield: 31%) as a colorless solid. ^1^H NMR (500 MHz, DMSO-*d*_6_): δ = 11.74–11.29 (m, 1H), 10.22 (s, 1H), 7.71–7.62 (m, 3H), 7.59–7.56 (m, 1H) (s, 1H), 7.55–7.53 (m, 1H), 6.95–6.89 (m, 2H), 6.51–6.46 (m, 1H), 3.74 (s, 3H) ppm. ^13^C NMR (126 MHz, DMSO-*d*_6_): δ = 165.6, 155.4, 133.6, 132.6, 129.5, 129.2, 128.8, 121.0 (2 C), 120.6, 120.4, 113.8 (2 C), 112.0, 100.9, 55.2. ppm. qHNMR (400 MHz, DMSO-*d*_6_, maleic acid as reference): purity = 98.2%. HRMS (ESI+): *m/z* calculated 301.0744 for C_16_H_14_ClN_2_O_2_, found 301.0732 ([M+H]^+^).

#### 5-Chloro-*N*-(3,4-dimethylphenyl)-1*H*-indole-6-carboxamide (27)

Preparation according to GP1 using **52** (35.0 mg, 0.179 mmol, 1.0 eq.), 3,4-dimethylaniline (**72**, 26.0 mg, 0.215 mmol, 1.2 eq.), NMI (49.4 μL, 0.626 mmol, 3.5 eq.) and TCFH (60.2 mg, 0.215 mmol, 1.2 eq.) yielded compound **27** (35.7 mg, yield: 67%) as a colorless solid. ^1^H NMR (500 MHz, DMSO-*d*_6_): δ = 11.52–11.35 (m, 1H), 10.19 (s, 1H), 7.75–7.65 (m, 1H), 7.59–7.50 (m, 3H), 7.46–7.40 (m, 1H), 7.11–7.06 (m, 1H), 6.51–6.46 (m, 1H), 2.21 (s, 3H), 2.19 (s, 3H) ppm. ^13^C NMR (126 MHz, DMSO-*d*_6_): δ = 165.9, 137.1, 136.3, 133.7, 131.3, 129.6, 129.6, 129.3, 128.8, 120.8, 120.6, 120.5, 117.1, 112.1, 101.0, 19.7, 18.9. ppm. qHNMR (400 MHz, acetone-*d*_6_, maleic acid as reference): purity = 98.9%. HRMS (ESI+): *m/z* calculated 299.0951 for C_17_H_16_ClN_2_O, found 299.0938 ([M+H]^+^).

#### 5-Chloro-*N*-(3,4-dichlorophenyl)-1*H*-indole-6-carboxamide (28)

Preparation according to GP1 using **52** (50.0 mg, 0.256 mmol, 1.0 eq.), 3,4-dichloroaniline (**73**, 48.6 mg, 0.281 mmol, 1.1 eq.), NMI (71.3 μL, 0.895 mmol, 3.5 eq.) and TCFH (78.9 mg, 0.281 mmol, 1.1 eq.) yielded compound **28** (31.1 mg, yield: 36%) as a colorless solid. ^1^H NMR (400 MHz, acetone-*d*_6_): δ = 10.72–10.67 (m, 1H), 9.67–9.62 (m, 1H), 8.26–8.21 (m, 1H), 7.78–7.71 (m, 2H), 7.71–7.66 (m, 1H), 7.59–7.52 (m, 2H), 6.58–6.51 (m, 1H) ppm. ^13^C NMR (101 MHz, acetone-*d*_6_): δ = 166.3, 139.5, 134.1, 131.8, 130.6, 130.3, 129.2, 128.7, 125.8, 121.1, 120.9, 120.9, 119.3, 112.4, 101.4 ppm. qHNMR (400 MHz, acetone-*d*_6_, maleic acid as reference): purity = 97.3%. HRMS (EI+): *m/z* calculated 337.9780 for C_15_H_9_Cl_3_N_2_O, found 337.9776 ([M]^+^).

#### 5-Chloro-*N*-(2,3-dihydro-1*H*-inden-5-yl)-1*H*-indole-6-carboxamide (29)

Preparation according to GP1 using **52** (50.0 mg, 0.256 mmol, 1.0 eq.), 2,3-dihydro-1*H*-inden-5-amine (**74**, 37.5 mg, 0.281 mmol, 1.1 eq.), NMI (71.3 μL, 0.895 mmol, 3.5 eq.) and TCFH (86.1 mg, 0.307 mmol, 1.2 eq.) yielded compound **29** (36.3 mg, yield: 46%) as a colorless solid. ^1^H NMR (400 MHz, MeOD): δ = 7.69–7.66 (m, 1H), 7.64–7.61 (m, 1H), 7.61–7.55 (m, 1H), 7.45–7.33 (m, 2H), 7.23–7.16 (m, 1H), 6.53–6.47 (m, 1H), 2.98–2.86 (m, 4H), 2.19–2.05 (m, 2H) ppm. ^13^C NMR (101 MHz, MeOD): δ = 169.0, 145.3, 140.9, 137.4, 134.8, 130.9, 130.0, 128.6, 124.6, 121.8, 121.4, 119.2, 117.3, 112.3, 101.7, 33.3, 32.7, 26.1 ppm. qHNMR (400 MHz, acetone-*d*_6_, maleic acid as reference): purity = 96.4%. HRMS (EI+): *m/z* calculated 310.0873 for C_18_H_15_ClN_2_O, found 310.0874 ([M]^+^).

#### 5-Chloro-*N*-(5,6,7,8-tetrahydronaphthalen-2-yl)-1*H*-indole-6-carboxamide (30)

Preparation according to GP1 using **52** (50.0 mg, 0.256 mmol, 1.0 eq.), 5,6,7,8-tetrahydronaphthalen-2-amine (**75**, 42.2 mg, 0.281 mmol, 1.1 eq.), NMI (71.3 μL, 0.895 mmol, 3.5 eq.) and TCFH (86.1 mg, 0.307 mmol, 1.2 eq.) yielded compound **30** (50.7 mg, yield: 61%) as a colorless solid. ^1^H NMR (500 MHz, DMSO-*d*_6_): δ = 11.47–11.44 (m, 1H), 10.21–10.17 (m, 1H), 7.71–7.67 (m, 1H),7.58 –7.52 (m, 2H), 7.52–7.48 (m, 1H), 7.42–7.36 (m, 1H), 7.03–6.98 (m, 1H), 6.51–6.46 (m, 1H), 2.77–2.59 (m, 4H), 1.83–1.62 (m, 4H) ppm. ^13^C NMR (126 MHz, DMSO-*d*_6_): δ = 165.8, 136.8, 136.7, 133.7, 131.8, 129.6, 129.3, 129.0, 128.8, 120.6, 120.4, 119.8, 117.2, 112.0, 101.0, 29.1, 28.3, 22.9, 22.8 ppm. qHNMR (400 MHz, acetone-*d*_6_, maleic acid as reference): purity = 97.7%. HRMS (EI+): *m/z* calculated 324.1029 for C_19_H_17_ClN_2_O, found 324.1023 ([M]^+^).

#### 5-Chloro-*N*-(naphthalen-2-yl)-1*H*-indole-6-carboxamide (31)

Preparation according to GP1 using **52** (40.0 mg, 0.204 mmol, 1.0 eq), naphthalen-2-amine (**76**, 32.2 mg, 0.225 mmol, 1.1 eq.), NMI (57.1 μL, 0.716 mmol, 3.5 eq.) and TCFH (68.9 mg, 0.245 mmol, 1.2 eq.) yielded compound **31** (33.4 mg, yield: 51%) as a colorless solid. ^1^H NMR (400 MHz, DMSO-*d*_6_): δ = 11.79–11.18 (m, 1H), 10.59 (s, 1H), 8.52–8.47 (m, 1H), 7.93–7.83 (m, 3H), 7.75–7.68 (m, 2H), 7.66 (s, 1H), 7.59–7.54 (m, 1H), 7.54–7.46 (m, 1H), 7.46–7.38 (m, 1H), 6.54–6.48 (m, 1H) ppm. ^13^C NMR (101 MHz, DMSO-*d*_6_): δ = 166.3, 136.9, 133.6, 133.4, 129.9, 129.4, 129.3, 128.9, 128.3, 127.5, 127.4, 126.5, 124.7, 120.6, 120.5, 120.2, 115.6, 112.2, 101.0 ppm. qHNMR (400 MHz, acetone-*d*_6_, maleic acid as reference): purity = 97.3%. HRMS (ESI+): *m/z* calculated 321.0795 for C_19_H_14_ClN_2_O, found 321.0782 ([M+H]^+^).

#### 5-Chloro-*N*-(1*H*-indol-5-yl)-1*H*-indole-6-carboxamide (32)

Preparation according to GP1 using **52** (50.0 mg, 0.256 mmol, 1.0 eq.), 1*H*-indol-5-amine (**77**, 37.2 mg, 0.281 mmol, 1.1 eq.), NMI (71.3 μL, 0.895 mmol, 3.5 eq.) and TCFH (86.1 mg, 0.307 mmol, 1.2 eq.) yielded compound **32** (46.4 mg, yield: 57%) as a colorless solid. ^1^H NMR (500 MHz, DMSO-*d*_6_): δ = 11.46–11.43 (m, 1H), 11.03–10.99 (m, 1H), 10.15 (s, 1H), 8.04–8.00 (m, 1H), 7.71–7.67 (m, 1H), 7.61–7.57 (m, 1H), 7.56–7.51 (m, 1H), 7.39–7.30 (m, 3H), 6.51–6.46 (m, 1H), 6.43–6.38 (m, 1H). ppm. ^13^C NMR (126 MHz, DMSO-*d*_6_): δ = 166.0, 134.2, 133.3, 131.8, 130.5, 129.6, 129.1, 127.9, 126.4, 121.1, 120.8, 115.6, 112.4, 111.6, 111.5, 101.6, 101.4 ppm. qHNMR (400 MHz, DMSO-*d*_6_, maleic acid as reference): purity = 96.2%. HRMS (EI+): *m/z* calculated 309.0669 for C_17_H_12_ClN_3_O, found 309.0662 ([M]^+^).

#### 5-Chloro-N-(1*H*-indol-6-yl)-1*H*-indole-6-carboxamide (33)

Preparation according to GP1, using **52** (50.0 mg, 0.256 mmol, 1.0 eq.), 1H-indol-6-amine (**78**, 37.2 mg, 0.281 mmol, 1.1 eq.), NMI (71.3 μL, 0.895 mmol, 3.5 eq.) and TCFH (86.1 mg, 0.307 mmol, 1.2 eq.) yielded compound **33** (53.2 mg, yield: 62%) as a colourless solid. ^1^H NMR (500 MHz, DMSO-*d*_6_): δ = 11.54–11.36 (m, 1H), 11.10–10.95 (m, 1H), 10.25 (s, 1H), 8.14–8.10 (m, 1H), 7.71–7.68 (m, 1H), 7.62–7.58 (m, 1H), 7.56–7.51 (m, 1H), 7.48–7.43 (m, 1H), 7.30–7.26 (m, 1H), 7.21–7.15 (m, 1H), 6.53–6.46 (m, 1H), 6.38–6.34 (m, 1H) ppm. ^13^C NMR (126 MHz, DMSO-*d*_6_): δ = 165.8, 135.9, 133.8, 133.6, 130.0, 129.2, 128.8, 125.2, 124.2, 120.7, 120.5, 119.8, 112.7, 112.1, 102.6, 101.0 (2 C) ppm. qHNMR (400 MHz, DMSO-*d*_6_, maleic acid as reference): purity = 96.9%. HRMS (ESI-): m/z calculated 308.05907 for C_17_H_11_ClN_3_O, found 308.0587 ([M-H]^-^).

#### 5-Chloro-*N*-(quinolin-2-yl)-1*H*-indole-6-carboxamide (34)

Preparation according to GP1 using **52** (50.0 mg, 0.256 mmol, 1.0 eq.), quinolin-2-amine (**79**, 40.5 mg, 0.281 mmol, 1.1 eq.), NMI (71.3 μL, 0.895 mmol, 3.5 eq.) and TCFH (86.1 mg, 0.307 mmol, 1.2 eq.) yielded compound **34** (13.5 mg, yield: 16%) as a colorless solid. ^1^H NMR (400 MHz, acetone-*d*_*6*_): δ = 10.88–10.57 (m, 1H), 10.02–9.75 (m, 1H), 8.63–8.56 (m, 1H), 8.42–8.35 (m, 1H), 7.97–7.90 (m, 1H), 7.90–7.86 (m, 1H), 7.84–7.77 (m, 1H), 7.76–7.65 (m, 2H), 7.60–7.54 (m, 1H), 7.54–7.45 (m, 1H), 6.59–6.53 (m, 1H) ppm. ^13^C NMR (101 MHz, acetone-*d*_*6*_): δ = 167.6, 152.7, 147.9, 139.1, 135.1, 131.4, 130.7, 129.7, 129.7, 128.6, 128.5, 127.3, 125.9, 122.1, 121.9, 115.3, 113.9, 102.3 ppm. qHNMR (400 MHz, acetone-*d*_6_, maleic acid as reference): purity = 97.5 %. HRMS (ESI+): *m/z* calculated 322.0747 for C_18_H_13_ClN_3_O, found 322.0739 ([M+H]^+^).

#### 5-Chloro-*N*-(quinolin-7-yl)-1*H*-indole-6-carboxamide (35)

Preparation according to GP1 using **52** (50.0 mg, 0.256 mmol, 1.0 eq.), quinolin-7-amine (**80**, 40.5 mg, 0.281 mmol, 1.1 eq.), NMI (71.3 μL, 0.895 mmol, 3.5 eq.) and TCFH (86.1 mg, 0.307 mmol, 1.2 eq.) yielded compound **35** (14.5 mg, yield: 18%) as a colorless solid. ^1^H NMR (500 MHz, DMSO-*d*_6_): δ = 11.54–11.51 (m, 1H), 10.75 (s, 1H), 8.88 –8.84 (m, 1H), 8.59–8.55 (m, 1H), 8.32–8.27 (m, 1H), 7.98–7.93 (m, 1H), 7.90–7.85 (m, 1H), 7.79–7.72 (m, 1H), 7.70–7.66 (m, 1H), 7.61–7.54 (m, 1H), 7.47–7.41 (m, 1H), 6.54–6.49 (m, 1H)) ppm. ^13^C NMR (126 MHz, DMSO-*d*_6_): δ = 166.5, 151.0, 148.5, 140.4, 140.1, 135.7, 135.6, 133.7, 129.5, 129.1 (2 C), 128.5, 124.6, 120.6, 120.5 (2 C), 120.2, 116.5, 112.3, 101.1 ppm. qHNMR (400 MHz, DMSO-*d*_6_, maleic acid as reference): purity = 96.1%. HRMS (EI+): *m/z* calculated 321.0669 for C_18_H_12_ClN_3_O, found 321.0664 ([M]^+^).

#### 5-Chloro-*N*-(quinolin-6-yl)-1*H*-indole-6-carboxamide (36)

Preparation according to GP1 using **52** (50.0 mg, 0.256 mmol, 1.0 eq.), quinolin-6-amine (**81**, 40.5 mg, 0.281 mmol, 1.1 eq.), NMI (71.3 μL, 0.895 mmol, 3.5 eq.) and TCFH (86.1 mg, 0.307 mmol, 1.2 eq.) yielded compound **36** (32.5 mg, yield: 40%) as a colorless solid. ^1^H NMR (500 MHz, DMSO-*d*_6_): δ = 11.53–11.50 (m, 1H), 10.73 (s, 1H), 8.83–8.78 (m, 1H), 8.60–8.56 (m, 1H), 8.37–8.31 (m, 1H), 8.03–7.97 (m, 1H), 7.95–7.89 (m, 1H), 7.75–7.72 (m, 1H), 7.69–7.65 (m, 1H), 7.59–7.55 (m, 1H), 7.54–7.48 (m, 1H), 6.56–6.44 (m, 1H) ppm. ^13^C NMR (101 MHz, acetone-*d*_6_): δ = 167.2, 150.0, 146.6, 138.3, 136.3, 135.0, 131.1, 130.9, 130.6, 129.7, 129.5, 124.2, 122.6, 122.2, 121.8, 116.3, 113.3, 102.3 ppm. qHNMR (400 MHz, DMSO-*d*_6_, maleic acid as reference): purity = 98.3%. HRMS (EI+): *m/z* calculated 321.0669 for C_18_H_12_ClN_3_O, found 321.0657 ([M]^+^).

#### 5-Chloro-*N*-(quinolin-3-yl)-1*H*-indole-6-carboxamide (37)

Preparation according to GP1 using **52** (67.0 mg, 0.343 mmol, 1.0 eq.), quinolin-3-amine (**82**, 54.3 mg, 0.377 mmol, 1.1 eq.), NMI (95.6 μL, 1.20 mmol, 3.5 eq.) and TCFH (115 mg, 0.411 mmol, 1.2 eq.) yielded compound **37** (10.3 mg, yield: 9.4%) as a colorless solid. ^1^H NMR (500 MHz, DMSO-*d*_6_): δ = 11.61–11.50 (m, 1H), 10.89 (s, 1H), 9.05–9.01 (m, 1H), 8.91–8.87 (m, 1H), 8.01–7.95 (m, 2H), 7.76–7.74 (m, 1H), 7.73–7.70 (m, 1H), 7.70–7.64 (m, 1H), 7.64–7.55 (m, 2H), 6.54–6.49 (m, 1H) ppm. ^13^C NMR (126 MHz, DMSO-*d*_6_): δ = 167.2, 145.2, 144.8, 134.1, 133.5, 130.0, 129.6, 129.1, 129.1, 128.4, 128.3, 128.3, 127.6, 122.8, 121.1, 121.0, 112.8, 101.5 ppm. qHNMR (400 MHz, DMSO-*d*_6_, maleic acid as reference): purity = 97.3%. HRMS (EI+): *m/z* calculated 321.0669 for C_18_H_12_ClN_3_O, found 321.0660 ([M]^+^).

#### 5-Chloro-*N*-(1,2,3,4-tetrahydroquinolin-3-yl)-1*H*-indole-6-carboxamide (38)

Preparation according to GP1 using **52** (50.0 mg, 0.256 mmol, 1.0 eq.), 1,2,3,4-tetrahydroquinolin-3-amine (**83**, 41.7 mg, 0.281 mmol, 1.1 eq.), NMI (71.3 μL, 0.895 mmol, 3.5 eq.) and TCFH (86.1 mg, 0.307 mmol, 1.2 eq.) yielded compound **38** (14.3 mg, yield: 17%) as a colorless solid. ^1^H NMR (400 MHz, acetone-*d*_6_): δ = 10.66–10.60 (m, 1H), 7.69–7.62 (m, 1H), 7.61 –7.55 (m, 1H), 7.50–7.42 (m, 1H), 7.37–7.29 (m, 1H), 6.95–6.80 (m, 2H), 6.59–6.47 (m, 2H), 6.52–6.43 (m, 1H), 5.21–5.07 (m, 1H), 4.55–4.42 (m, 1H), 3.58–3.47 (m, 1H), 3.35–3.23 (m, 1H), 3.13–3.01 (m, 1H), 2.96–2.91 (m, 1H) ppm. ^13^C NMR (101 MHz, acetone-*d*_6_): δ = 167.8, 145.5, 135.1, 130.8, 130.6, 130.2, 129.1, 127.7, 122.0, 121.6, 119.3, 117.4, 114.7, 113.5, 102.1, 46.2, 44.3, 33.7 ppm. qHNMR (400 MHz, DMSO-*d*_6_, maleic acid as reference): purity = 98.7%. HRMS (EI+): *m/z* calculated 325.0982 for C_18_H_16_ClN_3_O, found 325.0982 ([M]^+^).

#### 5-Chloro-*N*-(6-chloroquinolin-3-yl)-1*H*-indole-6-carboxamide (39)

Preparation according to GP1 using **52** (23.9 mg, 0.122 mmol, 1.0 eq.), 6-chloroquinolin-3-amine (**39c**, 24.0 mg, 0.134 mmol, 1.1 eq.), NMI (34.1 μL, 0.428 mmol, 3.5 eq.) and TCFH (41.2 mg, 0.147 mmol, 1.2 eq.) yielded compound **39** (6.41 mg, yield: 15%) as a colorless solid. ^1^H NMR (500 MHz, DMSO-*d*_6_): δ = 11.67–11.47 (m, 1H), 10.97 (s, 1H), 9.17–8.99 (m, 1H), 8.97–8.81 (m, 1H), 8.26–8.10 (m, 1H), 8.02–7.97 (m, 1H), 7.77–7.74 (m, 1H), 7.74–7.71 (m, 1H), 7.70–7.64 (m, 1H), 7.62–7.57 (m, 1H), 6.55–6.50 (m, 1H) ppm. ^13^C NMR (126 MHz, DMSO-*d*_6_): δ = 166.8, 145.2, 142.7, 133.8, 133.6, 131.6, 130.6, 129.6, 129.2, 128.9, 128.4, 128.4, 126.4, 121.3, 120.6, 120.5, 112.4, 101.1 ppm. qHNMR (400 MHz, DMSO-*d*_6_, maleic acid as reference): purity = 95.7%. HRMS (ESI+): *m/z* calculated 356.0358 for C_18_H_12_Cl_2_N_3_O, found 356.0348 ([M+H]^+^).

#### 5-Chloro-*N*-(7-chloroquinolin-3-yl)-1*H*-indole-6-carboxamide (40)

Preparation according to GP1 using **52** (50.0 mg, 0.256 mmol, 1.0 eq.), 7-chloroquinolin-3-amine (**84**, 50.2 mg, 0.281 mmol, 1.1 eq.), NMI (71.3 μL, 0.895 mmol, 3.5 eq.) and TCFH (86.1 mg, 0.307 mmol, 1.2 eq.) yielded compound **40** (14.6 mg, yield: 16%) as a colorless solid. ^1^H NMR (400 MHz, DMSO-*d*_6_): δ = 11.67–11.45 (m, 1H), 10.95 (s, 1H), 9.16–9.03 (m, 1H), 9.04–8.85 (m, 1H), 8.10–8.01 (m, 2H), 7.77–7.70 (m, 2H), 7.67–7.50 (m, 2H), 6.55–6.49 (m, 1H) ppm. ^13^C NMR (101 MHz, DMSO-*d*_6_): δ = 166.8, 145.9, 144.5, 133.6, 133.4, 132.4, 129.8, 129.7, 129.2, 128.4, 127.7, 127.3, 126.6, 122.3, 120.7, 120.5, 112.4, 101.1 ppm. qHNMR (400 MHz, acetone-*d*_6_ + 2 drops of DMSO-*d*_6_, maleic acid as reference): purity = 95.5%. HRMS (EI+): *m/z* calculated 355.0279 for C_18_H_11_Cl_2_N_3_O, found 355.0265 ([M]^+^).

#### 5-Chloro-*N*-(6-methylquinolin-3-yl)-1*H*-indole-6-carboxamide (41)

Preparation according to GP1 using **52** (40.0 mg, 0.204 mmol, 1.0 eq), 6-methylquinolin-3-amine (**41c**), 35.6 mg, 0.225 mmol, 1.1 eq.), NMI (57.1 μL, 0.716 mmol, 3.5 eq.) and TCFH (68.9 mg, 0.245 mmol, 1.2 eq.) yielded compound **41** (15.3 mg, yield: 22%) as a colorless solid. ^1^H NMR (500 MHz, DMSO-*d*_6_): δ = 11.56–11.52 (m, 1H), 10.84 (s, 1H), 9.01–8.92 (m, 1H), 8.81–8.71 (m, 1H), 7.90–7.84 (m, 1H), 7.79–7.66 (m, 3H), 7.60–7.55 (m, 1H), 7.53–7.47 (m, 1H), 6.54–6.49 (m, 1H) ppm, 3H overlap with DMSO signal. ^13^C NMR (126 MHz, DMSO-*d*_6_): δ = 166.7, 143.8, 143.0, 136.6, 133.6, 133.1, 130.1, 129.5, 129.1, 128.6, 128.4, 127.8, 126.5, 121.7, 120.6, 120.5, 112.4, 101.1, 21.2 ppm. qHNMR (400 MHz, DMSO-*d*_6_, maleic acid as reference): purity = 98.4%. HRMS (ESI+): *m/z* calculated 336.0904 for C_19_H_15_ClN_3_O, found 336.0892 ([M+H]^+^).

#### 5-Chloro-*N*-(7-methylquinolin-3-yl)-1*H*-indole-6-carboxamide (42)

Preparation according to GP1 using **52** (40.0 mg, 0.204 mmol, 1.0 eq), 7-methylquinolin-3-amine (**42c**, 35.6 mg, 0.281 mmol, 1.1 eq.), NMI (57.1 μL, 0.716 mmol, 3.5 eq.) and TCFH (68.9 mg, 0.245 mmol, 1.2 eq.) yielded compound **42** (21.0 mg, yield: 31%) as a colorless solid. ^1^H NMR (500 MHz, DMSO-*d*_6_): δ = 11.57–11.54 (m, 1H), 10.83 (s, 1H), 9.00–8.96 (m, 1H), 8.84–8.80 (m, 1H), 7.90–7.85 (m, 1H), 7.79–7.76 (m, 1H), 7.77–7.69 (m, 2H), 7.60–7.55 (m, 1H), 7.47–7.41 (m, 1H), 6.54–6.49 (m, 1H), 2.52 (s, 3H) ppm. ^13^C NMR (126 MHz, DMSO-*d*_6_): δ = 166.7, 144.7, 144.6, 137.7, 133.6, 132.4, 129.6, 129.3, 129.1, 128.7, 127.6, 127.5, 125.8, 122.4, 120.6, 120.5, 112.3, 101.1, 21.3 ppm. qHNMR (400 MHz, DMSO-*d*_6_, maleic acid as reference): purity = 95.4%. HRMS (ESI+): *m/z* calculated 336.0904 for C_19_H_15_ClN_3_O, found 336.0890 ([M+H]^+^).

#### 5-Chloro-*N*-(1,5-naphthyridin-3-yl)-1*H*-indole-6-carboxamide (43)

Preparation according to GP1 using **52** (50.0 mg, 0.256 mmol, 1.0 eq.), 1,5-naphthyridin-3-amine (**85**, 40.8 mg, 0.281 mmol, 1.1 eq.), NMI (71.3 μL, 0.895 mmol, 3.5 eq.) and TCFH (86.1 mg, 0.307 mmol, 1.2 eq.) yielded compound **43** (18.1 mg, yield: 22%) as a colorless solid. ^1^H NMR (400 MHz, DMSO-*d*_6_): δ = 11.65–11.51 (m, 1H), 11.07 (s, 1H), 9.22–9.17 (m, 1H), 9.02–8.96 (m, 1H), 8.93 (s, 1H), 8.43–8.36 (m, 1H), 7.81–7.74 (m, 2H), 7.75–7.67 (m, 1H), 7.63–7.57 (m, 1H), 6.56–6.50 (m, 1H) ppm. ^13^C NMR (101 MHz, DMSO-*d*_6_): δ = 166.9, 151.9, 145.3, 143.6, 139.5, 136.4, 136.1, 133.6, 129.7, 129.3, 128.3, 123.2, 122.6, 120.7, 120.5, 112.5, 101.1 ppm. qHNMR (400 MHz, acetone-*d*_6_, maleic acid as reference): purity = 100.0%. HRMS (EI+): *m/z* calculated 322.06214 for C_17_H_11_ClN_4_O, found 322.0616 ([M]^+^).

#### 5-Chloro-*N*-[1*H*-pyrrolo(2,3-b)pyridin-5-yl]-1*H*-indole-6-carboxamide (44)

Preparation according to GP1 using **52** (50.0 mg, 0.256 mmol, 1.0 eq.), 1*H*-pyrrolo[2,3-b]pyridin-5-amine (**86**, 37.4 mg, 0.281 mmol, 1.1 eq.), NMI (71.3 μL, 0.895 mmol, 3.5 eq.) and TCFH (86.1 mg, 0.307 mmol, 1.2 eq.) yielded compound **44** (16.8 mg, yield: 16%) as a colorless solid. ^1^H NMR (400 MHz, DMSO-*d*_6_): δ = 11.60–11.55 (m, 1H), 11.51–11.46 (m, 1H), 10.38 (s, 1H), 8.46–8.41 (m, 1H), 8.41–8.37 (m, 1H), 7.73–7.69 (m, 1H), 7.66–7.62 (m, 1H), 7.58–7.52 (m, 1H), 7.49–7.43 (m, 1H), 6.53–6.47 (m, 1H), 6.47–6.42 (m, 1H) ppm. ^13^C NMR (101 MHz, DMSO-*d*_6_): δ = 166.1, 145.5, 136.3, 133.7, 129.4, 129.3, 129.0, 128.8, 126.9, 120.6, 120.5, 119.4, 119.0, 112.1, 101.0, 99.8 ppm. qHNMR (400 MHz, acetone-*d*_6_ + 4 drops DMSO-*d*_6_, maleic acid as reference): purity = 97.2%. HRMS (ESI+): *m/z* calculated 311.0700 for C_16_H_12_ClN_4_O, found 311.0695 ([M+H]^+^).

#### 5-Chloro-*N*-[1-methyl-1H-pyrrolo(2,3-*b*)pyridin-5-yl]-1H-indole-6-carboxamide (45)

Preparation according to GP1 using **52** (50.0 mg, 0.256 mmol, 1.0 eq.), 1-methyl-1*H*-pyrrolo[2,3-*b*]pyridin-5-amine (**45b**, 41.4 mg, 0.281 mmol, 1.1 eq.), NMI (71.3 μL, 0.895 mmol, 3.5 eq.) and TCFH (86.1 mg, 0.307 mmol, 1.2 eq.) yielded compound **45** (14.0 mg, yield: 17%) as a colorless solid. ^1^H NMR (400 MHz, DMSO-*d*_6_): δ = 11.69–11.37 (m, 1H), 10.41 (s, 1H), 8.60–8.35 (m, 2H), 7.76–7.62 (m, 2H), 7.58–7.48 (m, 2H), 6.53 –6.44 (m, 2H), 3.81 (s, 3H) ppm. ^13^C NMR (126 MHz, DMSO-*d*_6_): δ = 166.1, 144.5, 136.2, 133.7, 130.8, 129.3 (2 C), 129.1, 128.9, 120.6, 120.5, 119.7, 119.4, 112.2, 101.0, 98.8, 31.0. ppm. qHNMR (400 MHz, acetone-*d*_6_, maleic acid as reference): purity = 95.3%. HRMS (ESI+): *m/z* calculated 325.0856 for C_17_H_14_ClN_4_O, found 325.0847 ([M+H]^+^).

#### *N*-(Benzofuran-5-yl)-5-chloro-1*H*-indole-6-carboxamide (46)

Preparation according to GP1 using **52** (50.0 mg, 0.256 mmol, 1.0 eq.), benzofuran-5-amine (**87**, 37.4 mg, 0.281 mmol, 1.1 eq.), NMI (71.3 μL, 0.895 mmol, 3.5 eq.) and TCFH (86.1 mg, 0.307 mmol, 1.2 eq.) yielded compound **46** (56.6 mg, yield: 71%) as a colorless solid. ^1^H NMR (500 MHz, DMSO-*d*_6_): δ = 11.61–11.31 (m, 1H), 10.40 (s, 1H), 8.23–8.08 (m, 1H), 8.02–7.92 (m, 1H), 7.74–7.68 (m, 1H), 7.64–7.59 (m, 1H), 7.59–7.52 (m, 3H), 7.01–6.86 (m, 1H), 6.52–6.42 (m, 1H) ppm. ^13^C NMR (126 MHz, DMSO-*d*_6_): δ = 165.9, 150.9, 146.6, 134.8, 133.7, 129.6, 129.3, 128.8, 127.3, 120.6, 120.5, 117.2, 112.1, 111.9, 111.1, 107.0, 101.0 ppm. qHNMR (400 MHz, acetone-*d*_6_, maleic acid as reference): purity = 97.5%. HRMS (ESI+): *m/z* calculated 311.0587 for C_17_H_12_ClN_2_O_2_, found 311.0576 ([M+H]^+^).

#### *N*-(Benzofuran-6-yl)-5-chloro-1*H*-indole-6-carboxamide (47)

Preparation according to GP1 using **52** (50.0 mg, 0.256 mmol, 1.0 eq.), benzofuran-6-amine (**88**, 37.4 mg, 0.281 mmol, 1.1 eq.), NMI (71.3 μL, 0.895 mmol, 3.5 eq.) and TCFH (86.1 mg, 0.307 mmol, 1.2 eq.) yielded compound **47** (30.3 mg, yield: 30%) as a colorless solid. ^1^H NMR (400 MHz, DMSO-*d*_6_): δ = 11.73–11.33 (m, 1H), 10.53 (s, 1H), 8.25–8.15 (m, 1H), 8.03–7.89 (m, 1H), 7.75–7.69 (m, 1H), 7.66–7.54 (m, 3H), 7.52–7.45 (m, 1H), 6.97–6.86 (m, 1H), 6.53–6.46 (m, 1H) ppm. ^13^C NMR (101 MHz, DMSO-*d*_6_): δ = 166.0, 154.4, 145.8, 136.5, 133.6, 129.3 (2 C), 128.9, 122.9, 121.0, 120.5, 120.5, 115.5, 112.1, 106.6, 102.3, 101.0 ppm. qHNMR (400 MHz, DMSO-*d*_6_, maleic acid as reference): purity = 96.9%. HRMS (ESI-): *m/z* calculated 309.0431 for C_17_H_10_ClN_2_O_2_, found 309.0436 ([M-H]^-^).

#### 5-Chloro-*N*-methyl-*N*-(quinolin-3-yl)-1*H*-indole-6-carboxamide (48)

Preparation according to GP1 using **52** (50.0 mg, 0.256 mmol, 1.0 eq.), *N*-methylquinolin-3-amine (**48a**, 44.5 mg, 0.281 mmol, 1.1 eq.), NMI (71.3 μL, 0.895 mmol, 3.5 eq.) and TCFH (86.1 mg, 0.307 mmol, 1.2 eq.) yielded compound **48** (6.0 mg, yield: 7.0%) as a colorless solid. ^1^H NMR (400 MHz, DMSO-*d*_6_, 80°C): δ = 11.25–11.07 (m, 1H), 8.86–8.68 (m, 1H), 8.32–8.26 (m, 1H), 7.93–7.83 (m, 2H), 7.73–7.64 (m, 1H), 7.59–7.53 (m, 1H), 7.51–7.45 (m, 2H), 7.41–7.34 (m, 1H), 6.55–6.20 (m, 1H), 3.49 (s, 3H) ppm. ^13^C NMR (101 MHz, DMSO-*d*_6_): 160.8, 149.1, 145.1, 136.7, 133.4, 130.8, 129.0, 128.7, 128.2, 128.1, 128.0, 127.4, 127.0, 126.6, 119.7, 119.6, 111.5, 100.6, 37.0 ppm. HRMS (ESI-): *m/z* calculated 334.0747 for C_19_H_13_ClN_3_O, found 334.0753 ([M-H]^-^).

#### *N*-[(5-Chloro-1*H*-indol-6-yl)methyl]quinolin-3-amine (49)

5-Chloro-1*H*-indole-6-carbaldehyde (**49c**, 50.5 mg, 0.283 mmol, 1.0 eq.) and quinolin-3-amine (**82**, 49 mg, 0.34 mmol μmol, 1.2 eq.) were dissolved in a mixture of DCM (3 mL) and DMF (1 mL). Acetic acid (31 μL, 566 μmol, 2.00 eq.) was added to the solution and the reaction mixture was stirred for 2 h at rt. Then, sodium triacetoxyborohydride (255 mg, 1.2 mmol, 4.24 eq) was added and the solution was stirred for 2 h at rt. The reaction was quenched with saturated NaHCO3 solution and the aqueous layer was extracted with EtOAc (3 x 25 mL). The combined organic layers were dried over MgSO_4_, filtered and evaporated. The resulting residue was purified by reverse CC and yielded compound **49** (10.3 mg, 12%) as a colorless solid.. ^1^H NMR (500 MHz, DMSO-*d*_6_): δ = 11.31–10.97 (m, 1H), 8.63–8.59 (m, 1H), 7.81–7.75 (m, 1H), 7.68–7.64 (m, 1H), 7.61–7.56 (m, 1H), 7.46–7.43 (m, 1H), 7.38–7.28 (m, 3H), 6.97–6.91 (m, 2H), 6.44–6.35 (m, 1H), 4.52 (d, *J* = 5.8 Hz, 2H) ppm. ^13^C NMR (126 MHz, DMSO-*d*_6_): δ = 143.6, 142.3, 141.0 134.8, 129.5, 128.5, 127.6 (2 C), 126.9, 126.6, 125.8, 123.9, 123.3, 120.1, 111.2, 107.9, 100.6, 44.6 ppm. qHNMR (400 MHz, acetone-*d*_6_, maleic acid as reference): purity = 97.7%. HRMS (ESI-): *m/z* calculated 306.0800 for C_18_H_13_ClN_3_, found 306.0804 ([M-H]^-^).

#### 5-Chloro-*N*-(quinolin-4-yl)-1*H*-indole-6-carboxamide (50)

Preparation according to GP1 using **52** (50.0 mg, 0.256 mmol, 1.0 eq.), quinolin-4-amine (**89**, 40.5 mg, 0.281 mmol, 1.1 eq.), NMI (71.3 μL, 0.895 mmol, 3.5 eq.) and TCFH (86.1 mg, 0.307 mmol, 1.2 eq.) yielded compound **50** (15.1 mg, yield: 18%) as a colorless solid. ^1^H NMR (500 MHz, DMSO-*d*_6_): δ = 11.65–11.52 (m, 1H), 10.79 (s, 1H), 8.90–8.85 (m, 1H), 8.40–8.35 (m, 1H), 8.15–8.11 (m, 1H), 8.06–8.01 (m, 1H), 7.82–7.73 (m, 3H), 7.66–7.56 (m, 2H), 6.58–6.47 (m, 1H) ppm. ^13^C NMR (126 MHz, DMSO-*d*_6_): δ = 167.3, 150.9, 148.8, 141.7, 133.7, 129.7, 129.6, 129.4, 129.2, 128.7, 126.1, 122.9, 121.5, 120.7, 120.6, 113.3, 112.8, 101.2 ppm. qHNMR (400 MHz, DMSO-*d*_6_, maleic acid as reference): purity = 98.8%. HRMS (ESI-): *m/z* calculated 320.0591 for C_18_H_11_ClN_3_O, found 320.0597 ([M-H]^-^).

#### N-(5-Chloro-1*H*-indol-6-yl)quinoline-3-carboxamide (51)

Preparation according to GP1 using quinoline-3-carboxylic acid (**90**, 50.0 mg, 0.289 mmol, 1.0 eq.), 5-chloro-1*H*-indol-6-amine (**91**, 52.9 mg, 0.318 mmol, 1.1 eq.), NMI (80.6 μL, 1.01 mmol, 3.5 eq.) and TCFH (97.2 mg, 0.346 mmol, 1.2 eq.) yielded compound **51** (7.3 mg, yield: 7.9%) as a colorless solid.. ^1^H NMR (500 MHz, DMSO-*d*_6_): δ = 11.39–11.31 (m, 1H), 10.37 (s, 1H), 9.43–9.39 (m, 1H), 9.03–8.99 (m, 1H), 8.20–8.11 (m, 2H), 7.95–7.88 (m, 1H), 7.77–7.70 (m, 2H), 7.70–7.66 (m, 1H), 7.49–7.44 (m, 1H), 6.49–6.44 (m, 1H) ppm. ^13^C NMR (126 MHz, DMSO-*d*_6_): δ = 164.4, 149.1, 148.6, 136.1, 134.4, 131.5, 129.3, 128.8, 127.7, 127.6, 127.5, 127.2, 127.1, 126.6, 121.2, 119.9, 111.4, 100.8 ppm. qHNMR (400 MHz, DMSO-*d*_6_, maleic acid as reference): purity = 97.1%. HRMS (ESI-): *m/z* calculated 320.0597 for C_18_H_11_ClN_3_O, found 320.0596 ([M-H]^-^).

#### *N*,*N-*Dimethyl-2-(2-nitro-4-(trifluoromethyl)phenoxy)ethan-1-amine (5a)

1-Fluoro-2-nitro-4-(trifluoromethyl)benzene (**92**, 300 mg, 1.43 mmol, 1.0 eq.) was dissolved in DMF (10 mL). To this solution, 2-(dimethylamino)ethan-1-ol (**93**, 0.188 mL, 1.87 mmol, 1.3 eq.) and Cs_2_CO_3_ (1.17 g, 3.59 mmol, 2.5 eq.) were added. The reaction mixture was stirred at 70°C for 2 h. After completion, the mixture was filtered and concentrated under reduced pressure. The residue was dissolved in EtOAc (20 mL) and washed with H_2_O (3 x 20 mL). The organic layer was dried over anhydrous MgSO_4_, filtered, and evaporated to dryness. The crude product was purified by CC, using a gradient from 100% cyclohexane to 100% EtOAc, yielding compound **5a** (215 mg, 54%) as a pale-yellow solid. ^1^H NMR (400 MHz, acetone-*d*_6_): δ = 8.24–8.16 (m, 1H), 8.02–7.94 (m, 1H), 7.64–7.57 (m, 1H), 4.41 (t, *J* = 5.7 Hz, 2H), 2.76 (t, *J* = 5.6 Hz, 2H), 2.28 (s, 6H) ppm. ^13^C NMR (101 MHz, DMSO-*d*_6_): δ = 153.7, 139.5, 131.0, 124.7 (q, *J* = 272.4 Hz), 122.4 (q, *J* = 4.1 Hz), 120.8 (q, *J* = 34.5 Hz), 116.1, 68.6, 57.1, 45.5 ppm. MS (APCI+): *m/z* 279.2 ([M+H]^+^).

#### 2-(2-(Dimethylamino)ethoxy)-5-(trifluoromethyl)aniline (5b)

*N*,*N*-Dimethyl-2-(2-nitro-4-(trifluoromethyl) phenoxy)ethan-1-amine (**5a**, 201 mg, 0.724 mmol, 1.0 eq.) was dissolved in MeOH (50 mL). Palladium on carbon (Pd/C, 10%) was added to the solution and the mixture was stirred under hydrogen atmosphere for 3 h. Upon completion, the mixture was filtered through a pad of Celite®, the solvent was removed under reduced pressure, and the crude product was purified by RP-CC using a gradient elution from 5:95 MeCN/H_2_O to 100% MeCN, yielding compound **5b** as a brownish solid (179 mg, 99%). ^1^H NMR (400 MHz, acetone-*d*_6_): δ = 7.01–6.94 (m, 2H), 6.92–6.84 (m, 1H), 4.85 (s, 2H), 4.16 (t, *J* = 5.8 Hz, 2H), 2.71 (t, *J* = 5.8 Hz, 2H), 2.28 (s, 6H) ppm. ^13^C NMR (101 MHz, acetone-*d*_6_): δ = 149.5, 139.7, 125.9 (q, *J* = 270.4 Hz), 123.6 (q, *J* = 31.8 Hz), 114.4 (q, *J* = 4.4 Hz), 112.5, 110.8, 68.0, 58.9, 46.1 ppm. MS (APCI+): *m/z* 249.2 ([M+H]^+^).

#### *N*,*N*-Dimethyl-2-(2-nitrophenoxy)ethan-1-amine (6a)

2-Nitrophenol (**94**, 2.50 g, 17.8 mmol, 1.0 eq.) was dissolved in EtOH (20 mL), and added to a mixture of 2-chloro-*N*,*N*-dimethylethylamine hydrochloride (**95**, 2.93 g, 20.0 mmol, 1.3 eq.) and KOH (2.11 g, 37.7 mmol, 2.1 eq.) in *n*-butanol (25.0 mL) and DMF (5.0 mL) and heated to reflux overnight. After cooling to rt, the reaction mixture was concentrated under reduced pressure. H_2_O (400 mL) was added and acidified with conc. HCl to pH 2. The mixture was washed with EtOAc (3 x 100 mL). The aqueous layer was then adjusted to pH 10 with NaOH (2 M) and re-extracted with EtOAc (3 x 100 mL). The combined organic extracts were dried over anhydrous MgSO_4_, filtered, and concentrated under reduced pressure. The crude product was purified by CC using a gradient of *iso-*hexane and EtOAc (4:6) to yield compound **6a** (2.10 g, 56%) as a yellow solid. ^1^H NMR (400 MHz, acetone-*d*_6_): δ = 7.81 (dd, *J* = 8.0, 1.7 Hz, 1H), 7.66–7.58 (m, 1H), 7.38–7.31 (m, 1H), 7.15–7.07 (m, 1H), 4.26 (t, *J* = 5.8 Hz, 2H), 2.71 (t, *J* = 5.7 Hz, 2H), 2.26 (s, 6H) ppm. ^13^C NMR (101 MHz, acetone-*d*_6_): δ = 151.7, 140.5, 133.9, 124.8, 120.4, 114.9, 68.4, 57.6, 45.3 ppm. MS (APCI+): *m/z* 211.2 ([M+H]^+^).

#### 2-[2-(Dimethylamino)ethoxy]aniline (6b)

*N*,*N*-Dimethyl-2-(2-nitrophenoxy)ethan-1-amine (**6a**, 489 mg, 2.33 mmol, 1.0 eq.) was dissolved in MeOH (30 mL). Palladium on carbon (Pd/C, 10%) was added to the solution and the mixture was stirred under hydrogen atmosphere for 2 h at rt. Upon completion (TLC), the reaction mixture was filtered through a pad of Celite®. The solvent was removed under reduced pressure, and the crude product was purified by CC using a gradient of *iso-*hexane and EtOAc (2:8), yielding compound **6b** (357 mg, 85%) as a pale-yellow solid. ^1^H NMR (400 MHz, acetone-*d*_6_): δ = 6.85–6.78 (m, 1H), 6.76–6.65 (m, 2H), 6.62–6.51 (m, 1H), 4.05 (t, *J* = 5.8 Hz, 2H), 2.66 (t, *J* = 5.8 Hz, 2H), 2.27 (s, 6H) ppm. ^13^C NMR (101 MHz, acetone-*d*_6_): δ = 146.2, 138.4, 121.4, 116.9, 114.4, 112.8, 67.1, 58.4, 45.3 ppm. MS (APCI+): *m/z* 181.3 ([M+H]^+^).

#### 5-Chloro-2-[2-(dimethylamino)ethoxy]benzonitrile (7a)

5-Chloro-2-hydroxybenzonitrile (**96**, 300 mg, 1.95 mmol, 1.0 eq.) was dissolved in THF (10 mL) and 2-chloro-*N*,*N*-dimethylethylamine hydrochloride (**95**, 338 mg, 2.34 mmol, 1.2 eq.), Cs_2_CO_3_ (1.91 g, 5.86 mmol, 3.0 eq.), and KI (32.4 mg, 0.195 mmol, 0.1 eq.) were added sequentially. The reaction mixture was heated to reflux for 16 h. After completion, the mixture was filtered through a pad of Celite® and the solvent was removed under reduced pressure yielding compound **7a** (411 mg, 94%) as a colorless solid. ^1^H NMR (400 MHz, acetone-*d*_6_): δ = 7.75–7.69 (m, 1H), 7.69–7.61 (m, 1H), 7.32–7.25 (m, 1H), 4.28 (t, *J* = 5.7 Hz, 2H), 2.74 (t, *J* = 5.7 Hz, 2H), 2.29 (s, 6H) ppm. ^13^C NMR (101 MHz, acetone-*d*_6_): δ = 159.6, 134.5, 132.7, 125.0, 114.7, 114.6, 103.2, 68.3, 57.5, 45.3 ppm. MS (APCI+): *m/z* 225.7 ([M+H]^+^).

#### 2-[2-(Aminomethyl)-4-chlorophenoxy]-*N*,*N*-dimethylethan-1-amine (7b)

A solution of 5-chloro-2-[2-(dimethylamino)ethoxy]benzonitrile (**7a**, 143 mg, 0.636 mmol, 1.0 eq.) in THF (4 mL) was added at 0°C to a solution of LiAlH_4_ (4 M in diethyl ether, 0.32 mL, 1.28 mmol, 2.0 eq.) in dry THF (10 mL) under nitrogen atmosphere. The mixture was stirred at rt for 18 h. After the reaction was complete, the excess LiAlH_4_ was quenched with saturated aqueous Na_2_SO_4_ solution. The mixture was then filtered, and the solvent was removed under reduced pressure. The residue was partitioned between H_2_O (30 mL) and EtOAc (30 mL), the layers were separated, and the aqueous layer was further extracted with EtOAc (3 × 20 mL). The combined organic layers were dried over anhydrous MgSO_4_, filtered, and concentrated. The crude product was purified by RP-CC using a gradient from 5:95 MeCN/H_2_O to 100% MeCN to obtain compound **7b** (63.8 mg, 44%) as a colorless solid. ^1^H NMR (400 MHz, acetone-*d*_6_): δ = 7.49–7.44 (m, 1H), 7.25–7.14 (m, 1H), 7.00–6.93 (m, 1H), 4.39–4.34 (m, 2H), 4.11 (t, *J* = 5.8 Hz, 2H), 2.85–2.77 (m, 2H), 2.69 (t, *J* = 5.8 Hz, 2H), 2.27 (s, 6H) ppm. ^13^C NMR (101 MHz, acetone-*d*_6_): δ = 155.9, 132.8, 129.0, 127.5, 125.7, 113.5, 68.0, 58.9, 49.5, 46.2 ppm. MS (APCI+): *m/z* 229.7 ([M+H]^+^).

#### 6-Chloroquinoline 1-oxide (39a)

Preparation according to GP2 using 6-chloroquinoline (**97**, 500 mg, 3.06 mmol, 1.0 eq.) and mCPBA (70%, 1.51 g, 6.12 mmol, 2.0 eq.) in CH_2_Cl_2_ (30 mL) yielded compound **39a** (420 mg, 77%) as a colorless solid. ^1^H NMR (400 MHz, acetone-*d*_6_): δ = 8.62 (d, *J* = 9.3 Hz, 1H), 8.53–8.47 (m, 1H), 8.13 (d, *J* = 2.3 Hz, 1H), 7.90–7.73 (m, 2H), 7.55–7.47 (m, 1H) ppm. ^13^C NMR (101 MHz, acetone-*d*_6_): δ = 141.2, 136.3, 135.0, 132.6, 131.2, 128.1, 124.4, 124.0, 122.5 ppm. MS (APCI+): *m/z* 180.6 ([M+H]^+^).

#### 6-Chloroquinolin-3-amine (39c)

Preparation according to GP3 using 6-chloroquinoline 1-oxide (**39a**, 316 mg, 1.76 mmol, 1.0 eq.) and *tert*-butyl nitrite (0.814 mL, 6.16 mmol, 3.5 eq.) in MeCN (4 mL) yielded 6-chloro-3-nitroquinoline 1-oxide (**39b**) as yellow solid. **39b** was used in the next step without further purification. **39c** was prepared according to GP4 using 6-chloro-3-nitroquinoline 1-oxide (**39b**), NH_4_Cl (233 mg, 4.36 mmol, 7.0 eq.), and Fe powder (348 mg, 6.23 mmol, 10.0 eq.) to yield compound **39c** (22.0 mg, 7.0% over two steps) as a colorless solid. ^1^H NMR (400 MHz, DMSO-*d*_6_): δ = 8.43 (d, *J* = 2.7 Hz, 1H), 7.80–7.69 (m, 2H), 7.28 (dd, *J* = 8.8, 2.4 Hz, 1H), 7.11–7.05 (m, 1H), 5.86 (s, 2H) ppm. ^13^C NMR (126 MHz, DMSO-*d*_6_): δ = 143.9, 143.2, 139.2, 130.9, 130.6, 129.9, 124.0, 124.0, 110.1 ppm. MS (APCI+): *m/z* 179.6 ([M+H]^+^).

#### 6-Methylquinoline 1-oxide (41a)

Preparation according to GP2 using 6-methylquinoline (**98**, 929 mg, 6.49 mmol, 1.0 eq.) and mCPBA (70%, 2.40 g, 9.74 mmol, 1.5 eq.) in CH_2_Cl_2_ (65 mL) yielded compound **41a** (856 mg, 85%) as a colorless solid. ^1^H NMR (400 MHz, DMSO-*d*_6_): δ = 8.53–8.46 (m, 1H), 8.44–8.37 (m, 1H), 7.85–7.78 (m, 2H), 7.63 (dd, *J* = 8.9, 1.9 Hz, 1H), 7.45–7.37 (m, 1H), 2.48 (s, 3H) ppm. ^13^C NMR (126 MHz, DMSO-*d*_6_): δ = 139.8, 139.0, 135.0, 132.8, 130.9, 127.8, 125.0, 122.4, 119.2, 21.3 ppm. MS (APCI+): *m/z* 160.2 ([M+H]^+^).

#### 6-Methyl-3-nitroquinoline 1-oxide (41b)

Preparation according to GP3 using 6-methylquinoline 1-oxide (**41a**, 603 mg, 1.52 mmol, 1.0 eq.) and *tert*-butyl nitrite (1.75 mL, 13.3 mmol, 3.5 eq.) yielded compound **41b** (530 mg, 69%) as a yellow solid. ^1^H NMR (400 MHz, DMSO-*d*_6_): δ = 9.12 (d, *J* = 1.9 Hz, 1H), 8.89–8.84 (m, 1H), 8.52–8.45 (m, 1H), 8.21–8.16 (m, 1H), 7.89 (dd, *J* = 9.0, 1.9 Hz, 1H), 2.55 (s, 3H) ppm. ^13^C NMR (101 MHz, DMSO-*d*_6_): δ = 142.6, 142.0, 141.3, 136.5, 130.5, 129.5, 128.1, 121.3, 119.5 ppm. MS (APCI+): *m/z* 205.2 ([M+H]^+^).

#### 6-Methylquinolin-3-amine (41c)

Preparation according to GP4 using 6-methyl-3-nitroquinoline-1-oxide (**41b**, 450 mg, 2.20 mmol, 1.0 eq.), NH_4_Cl (825 mg, 15.4 mmol, 7.0 eq.), and Fe powder (1.23 g, 22.0 mmol, 10 eq.) yielded compound **41c** (106 mg, 30.5%) as a colorless solid. ^1^H NMR (400 MHz, DMSO-*d*_6_): δ = 8.35 (d, *J* = 2.6 Hz, 1H), 7.64 (d, *J* = 8.4 Hz, 1H), 7.38–7.33 (m, 1H), 7.14 (dd, *J* = 8.5, 2.0 Hz, 1H), 2.40 (s, 3H), 7.06–7.00 (m, 1H), 5.64–5.52 (m, 2H) ppm. ^13^C NMR (101 MHz, DMSO-*d*_6_): δ = 142.5, 142.4, 139.7, 135.6, 129.4, 128.3, 126.0, 124.3, 111.0, 21.2 ppm. MS (APCI+): *m/z* 159.2 ([M+H]^+^).

#### 7-Methylquinoline 1-oxide (42a)

Preparation according to GP2 using 7-methylquinoline (**99**, 1.60 g, 11.2 mmol, 1.0 eq.) in CH_2_Cl_2_ (50 mL) and mCPBA (70%, 4.13 g, 16.9 mmol, 1.5 eq.) in CH_2_Cl_2_ (100 mL) yielded compound **42a** (1.16 g, 65%) as a colorless solid. ^1^H NMR (400 MHz, DMSO-*d*_6_): δ = 8.57–8.51 (m, 1H), 8.37–8.31 (m, 1H), 8.02–7.94 (m, 1H), 7.92–7.79 (m, 1H), 7.60–7.53 (m, 1H), 7.43–7.35 (m, 1H), 2.55 (s, 3H) ppm. ^13^C NMR (101 MHz, DMSO-*d*_6_): δ = 141.3, 135.7, 131.2, 130.3, 128.9, 128.9, 125.4, 121.4, 118.3, 22.1 ppm. MS (APCI+): *m/z* 160.2 ([M+H]^+^).

#### 7-Methyl-3-nitroquinoline 1-oxide (42b)

Preparation according to GP3 using 7-methylquinoline 1-oxide (**42a**, 1.10 g, 6.90 mmol, 1.0 eq.) and *tert*-butyl nitrite (3.20 mL, 24.0 mmol, 3.5 eq.) yielded compound **42b** (660 mg, 47%) as a yellow solid. ^1^H NMR (400 MHz, DMSO-*d*_6_): δ = 9.16 (d, *J* = 2.0 Hz, 1H), 8.96–8.91 (m, 1H), 8.44–8.39 (m, 1H), 8.35–8.28 (m, 1H), 7.78 (dd, *J* = 8.4, 1.7 Hz, 1H), 2.64–2.60 (m, 3H). ^13^C NMR (126 MHz, DMSO-*d*_6_): δ = 146.1, 143.5, 141.9, 133.0, 131.6, 130.2, 126.1, 121.8, 118.7, 22.4 ppm. MS (APCI+): *m/z* 204.2 ([M+H]^+^).

#### 7-Methylquinolin-3-amine (42c)

Preparation according to GP3 using 7-Methyl-3-nitroquinoline-1-oxide (**42b**, 430 mg, 2.11 mmol, 1.0 eq.), NH_4_Cl (789 mg, 14.7 mmol, 7.0 eq.), and Fe powder (1.18 g, 21.1 mmol, 10 eq.) yielded compound **42c** (101 mg, 30 %) as a colorless solid. ^1^H NMR (400 MHz, DMSO-*d*_6_): δ = 8.38 (d, *J* = 2.7 Hz, 1H), 7.58–7.47 (m, 2H), 7.23–7.21 (m, 1H), 7.10 (d, *J* = 2.7 Hz, 1H), 2.40 (s, 3H) ppm. ^13^C NMR (101 MHz, DMSO-*d*_6_): δ = 143.3, 141.8, 141.3, 133.1, 128.5, 127.6, 127.4, 125.4, 111.8, 21.0 ppm. MS (APCI+): *m/z* 159.2 ([M+H]^+^).

#### 1-Methyl-5-nitro-1*H*-pyrrolo[2,3-b]pyridine (45a)

A solution of 5-nitro-7-azaindole (**100**, 0.195 g, 1.2 mmol) in DMF (10 mL) was cooled to 0°C and treated with NaH (60%, 0.057 g, 1.44 mmol), followed by iodomethane (0.204 g, 1.44 mmol). The reaction mixture was stirred at rt for 4 h, then poured into water (200 mL) and extracted with CH_2_Cl_2_ (3 × 100 mL). The combined organic layers were washed with brine (100 mL) and dried over anhydrous MgSO_4_. The solvent was removed under reduced pressure to yield **45a** as a yellow solid (0.151 g, 70.6%). ^1^H NMR (400 MHz, DMSO-*d*_6_): δ = 9.13 (d, *J* = 2.5 Hz, 1H), 8.88 (d, *J* = 2.5 Hz, 1H), 7.80 (d, *J* = 3.5 Hz, 1H), 6.77 (d, *J* = 3.4 Hz, 1H), 3.90 (s, 3H) ppm. ^13^C NMR (126 MHz, DMSO-*d*_6_): δ = 148.9, 138.7, 138.5, 134.2, 125.0, 119.1, 101.9, 31.4 ppm. MS (APCI+): *m/z* 178.2 ([M+H]^+^).

#### 1-Methyl-1*H*-pyrrolo[2,3-b]pyridin-5-amine (45b)

10% Pd/C (12 mg, 0.113 mmol) was added under nitrogen atmosphere to a solution of **45a** (200 mg, 1.13 mmol) in EtOH (20 mL). The flask was repeatedly evacuated and flushed with H_2_ gas before the resulting mixture was stirred at rt for 2 h under H_2_. The flask was subsequently purged with N_2_ gas and the solution was filtered through celite^©^. The filtrate was dried over anhydrous MgSO_4_, concentrated in vacuo and purified by RP-CC (gradient elution from 5:95 MeCN/H_2_O to 100% MeCN) to obtain **45b** as a colorless solid (70.0 mg, 42.1%). ^1^H NMR (400 MHz, DMSO-*d*_6_): δ = 7.75 (d, *J* = 2.5 Hz, 1H), 7.27 (d, *J* = 3.4 Hz, 1H), 7.08 (d, *J* = 2.5 Hz, 1H), 6.15 (d, *J* = 3.3 Hz, 1H), 4.79–4.51 (m, 2H), 3.70 (s, 3H) ppm. ^13^C NMR (101 MHz, DMSO-*d*_6_): δ = 142.0, 138.6, 132.5, 129.5, 120.2, 111.7, 97.0, 30.8 ppm. MS (APCI+): *m/z* 148.2 ([M+H]^+^).

#### *N*-Methylquinolin-3-amine (48a)

Quinoline-3-amine (**82**, 500 mg, 3.47 mmol, 1.0 eq.), triethyl orthoformate (3.5 mL, 20.6 mmol, 5.9 eq.) and TFA (26.7 μL, 0.347 mmol, 0.1 eq.) were added to 10 mL pressure vessel and heated to 125°C for 12 h. After cooling to rt, the reaction mixture was concentrated under reduced pressure and dissolved in EtOH (10 mL). Sodium borohydride (NaBH_4_, 723 mg, 19.1 mmol, 5.5 eq.) was added to the solution and stirred at rt overnight. The reaction was quenched with H_2_O (10 mL) and extracted with CH_2_Cl_2_ (3 × 25 mL). The combined organic phases were dried over MgSO_4_, filtered and concentrated under reduced pressure. The crude product was purified by normal-phase CC (gradient elution, 0-50% EtOAc in cyclohexane) to obtain **48a** (268 mg, 48.8%) as light brownish solid. ^1^H NMR (400 MHz, DMSO-*d*_6_): δ = 8.47–8.42 (m, 1H), 7.81–7.74 (m, 1H), 7.70–7.63 (m, 1H), 7.45–7.35 (m, 1H), 7.35–7.27 (m, 1H), 6.99–6.94 (m, 1H), 6.36–6.28 (m, 1H), 2.78 (s, 3H) ppm. ^13^C NMR (101 MHz, DMSO-*d*_6_): δ = 143.6 (2 C), 140.8, 129.7, 128.5, 126.6, 125.7, 123.7, 106.9, 29.4 ppm. MS (APCI+): *m/z* 158.2 ([M+H]^+^).

#### 5-Chloro-1*H*-indole-6-ylmethanol (49b)

Methyl 5-chloro-1*H*-indole-6-carboxylate (**49a**, 500 mg, 2.39 mmol, 1.0 eq.) was dissolved in THF (20 mL) under nitrogen. The solution was stirred at 0°C and a solution of lithium aluminium hydride (596 μL, 2.39 mmol, 1.0 eq., 4 M in diethyl ether) was added dropwise. The reaction mixture was stirred for 2 h and quenched carefully with EtOAc and water. The aqueous layer was extracted with EtOAc and the combined organic layer was dried over MgSO4, filtered and evaporated. The residue was purified by CC yielded compound **49b** as a light red solid (109 mg, 67%). ^1^H NMR (400 MHz, acetone-*d*_6_): δ = 10.35 (s, 1H), 7.72–7.65 (m, 1H), 7.57 (s, 1H), 7.40–7.31 (m, 1H), 6.48–6.41 (m, 1H), 4.81–4.73 (m, 2H), 4.25 (t, J = 5.7 Hz, 1H) ppm. ^13^C NMR (101 MHz, acetone-*d*_6_): δ = 135.3, 132.3, 127.9, 126.1, 123.0, 119.8, 110.7, 101.0, 61.8 ppm. MS (+APCI): m/z 181.6 ([M+H]^+^).

#### 5-Chloro-1*H*-indole-6-carbaldehyde (49c)

5-Chloro-1*H*-indole-6-ylmethanol (**49b**, 200 mg, 1.1 mmol, 1.0 eq.) was dissolved in a mixture of DCM (10 mL) and DMF (1 mL) at 0°C. Dess-Martin-Periodinan (DMP, 700 mg, 1.65 μmol, 1.5 eq.) was added to the solution at 0°C and the reaction mixture was allowed to warm to rt and stirred for 1.5 h. A solution of 1 N NaOH was added and the biphasic mixture was filtered through Celite. The biphasic mixture was extracted with EtOAc and the organic phase was dried over MgSO_4_. The organic solvent was removed under reduced pressure and the residue was purified by CC to yield compound **49c** as a light red solid (mixture of tautomers, 120 mg, 61%). ^1^H NMR (400 MHz, acetone-*d*_6_): δ = 10.91 (s, 1H), 10.48 (s, 1H), 8.07 (s, 1H), 7.75–7.67 (m, 2H), 6.63–6.56 (m, 1H) ppm. ^13^C NMR (101 MHz, acetone-*d*_6_): δ = 190.1, 135.5, 135.4, 134.4, 134.4, 132.5, 132.4, 128.1, 126.8, 121.9, 113.9, 113.8, 102.9, 102.8 ppm. MS (+APCI): m/z 179.7 ([M+H]^+^).

#### In vitro Characterization

##### Hybrid reporter gene assays

Nuclear receptor modulation was determined in Gal4 hybrid reporter gene assays in HEK293T cells (German Collection of Microorganisms and Cell Culture GmbH, DSMZ) as described previously^41^ using pFR-Luc (Stratagene, La Jolla, CA, USA; reporter), pRL-SV40 (Promega, Madison, WI, USA; internal control) and a pFA-CMV-hNR-LBD clone, coding for the hinge region and ligand binding domain of the canonical isoform of the respective human nuclear receptor. HEK293T cells were cultured in Dulbecco’s modified Eagle’s medium (DMEM), high glucose supplemented with 10% fetal calf serum (FCS), sodium pyruvate (1 mM), penicillin (100 U/mL), and streptomycin (100 μg/mL) at 37 °C and 5% CO_2_ and seeded in 96-well plates (3×10^4^ cells/well). After 24 h, medium was changed to Opti-MEM without supplements and cells were transiently transfected using Lipofectamine LTX reagent (Invitrogen, Carlsbad, CA, USA) according to the manufacturer’s protocol. Five hours after transfection, cells were incubated with the test compounds in Opti-MEM supplemented with penicillin (100 U/mL), streptomycin (100 μg/mL) and 0.1% DMSO for 16 h before luciferase activity was measured using the Dual-Glo Luciferase Assay System (Promega) according to the manufacturer’s protocol on a Tecan Spark luminometer (Tecan Deutschland GmbH, Crailsheim, Germany). Firefly luminescence was divided by Renilla luminescence and multiplied by 1000 resulting in relative light units (RLU) to normalize for transfection efficiency and cell growth. Fold activation was obtained by dividing the mean RLU of test compound by the mean RLU of the untreated control. All samples were tested in at least three biologically independent experiments in duplicates. For dose-response curve fitting and calculation of EC_50_ values, the equation “[Agonist] vs. response -- Variable slope (four parameters)” was used in GraphPad Prism (version 7.00, GraphPad Software, La Jolla, CA, USA). The following reference ligands were used: Triiodothyronine (1 μM, THR), tretinoin (1 μM, RAR), GW7647 (1 μM, PPARα), pioglitazone (1 μM, PPARγ), L165,041 (1 μM, PPARδ), SR1001 (1 μM, RORγ), calcitriol (1 μM, VDR), CITCO (10 μM, CAR), rifampicin (10 μM, PXR), TO901317 (1 μM, LXR), GW4064 (1 μM, FXR), bexarotene (1 μM, RXR). Activity on the Nurr1 C566S mutant was determined as for wild-type Nurr1 using the previously described mutant clone^21^.

##### Full-length Nurr1 reporter gene assays

Activation of full length human Nurr1 was studied in transiently transfected HEK293T cells using the reporter plasmids pFR-Luc-NBRE, pFR-Luc-POMC or pFR-Luc-DR5 each containing one copy of the respective human Nurr1 response element NBRE Nl3, NurRE, or DR5^16^. The full length human nuclear receptor Nurr1 (pcDNA3.1-hNurr1-NE; Addgene plasmid #102363) and, for DR5, RXRα (pSG5-hRXR)^42^ were overexpressed. pRL-SV40 (Promega) was used for normalization of transfection efficacy and to observe test compound toxicity. Cells were cultured in Dulbecco’s modified Eagle’s medium (DMEM), high glucose supplemented with 10% fetal calf serum (FCS), sodium pyruvate (1 mM), penicillin (100 U/mL), and streptomycin (100 μg/mL) at 37 °C and 5 % CO_2_ and seeded in 96-well plates (3×10^4^ cells/well). After 24 h, medium was changed to Opti-MEM without supplements and cells were transiently transfected using Lipofectamine LTX reagent (Invitrogen) according to the manufacturer’s protocol. Five hours after transfection, cells were incubated with the test compounds in Opti-MEM supplemented with penicillin (100 U/mL), streptomycin (100 μg/mL) and 0.1% DMSO for 16 h before luciferase activity was measured using the Dual-Glo Luciferase Assay System (Promega) according to the manufacturer’s protocol on a Tecan Spark luminometer (Tecan Deutschland GmbH). Firefly luminescence was divided by Renilla luminescence and multiplied by 1000 resulting in relative light units (RLU) to normalize for transfection efficiency and cell growth. Fold activation was obtained by dividing the mean RLU of test compound by the mean RLU of the untreated control. All samples were tested in at least three biologically independent experiments in duplicates. For dose-response curve fitting and calculation of EC_50_ values, the equation “[Agonist] vs. response -- Variable slope (four parameters)” was used in GraphPad Prism (version 7.00, GraphPad Software).

##### Isothermal Titration Calorimetry (ITC)

ITC experiments were conducted on an Affinity ITC instrument (TA Instruments, New Castle, DE) at 25°C with a stirring rate of 75 rpm. Nurr1 LBD protein (10-20 μM, expressed as described previously^43^) in buffer (20 mM Tris pH 7.5, 100 mM NaCl, 5% glycerol) containing 4% DMSO was titrated with the test compounds (50-100 μM in the same buffer containing 4% DMSO) in 26 injections (1x 1 μL, 25x 4 μL) with an injection interval of 150 s. As control experiments, the test compounds were titrated to the buffer, and the buffer was titrated to the Nurr1 LBD protein under otherwise identical conditions. Results were analyzed using NanoAnalyze software (version 3.11.0, TA Instruments, New Castle, DE) with independent binding models.

##### Evaluation of Nurr1-regulated gene expression in N27 cells

N27 rat dopaminergic neural cells (SCC048, Sigma-Aldrich, Darmstadt, Germany) were cultured in RPMI 1640 medium (Gibco, Thermo Fisher Scientific, Waltham, MA, USA) supplemented with 10% FCS, penicillin (100 U/mL), and streptomycin (100 μg/mL) at 37 °C and 5% CO_2_ and seeded in 12-well plates (3×10^5^ cells/well). After 8 h, the medium was changed to RPMI 1640 medium (Gibco, Thermo Fisher Scientific) supplemented with 0.2% FCS, penicillin (100 U/mL), and streptomycin (100 μg/mL), and the cells were incubated for another 22 h, before the medium was changed again to RPMI 1640 medium (Gibco, Thermo Fisher Scientific) supplemented with 0.2% FCS, penicillin (100 U/mL), and streptomycin (100 μg/mL), additionally containing either **37** (0.3, 1 or 3 μM) or 5-chloroindole (**3**, 100 μM) solubilized with 0.1% DMSO or 0.1% DMSO alone as negative control. After 21 h of incubation, the medium was removed, cells were washed with phosphate-buffered saline (PBS), and after full aspiration of residual liquids immediately frozen at -80 °C until further procession. All samples were prepared in six biologically independent repeats. Total RNA was isolated using E.Z.N.A. Total RNA Kit I (Omega Bio-tek, Norcross, GA, USA) following the manufacturer’s instructions. RNA concentration and purity were assessed using a NanoDrop One UV-vis spectrophotometer (Thermo Fisher Scientific) at 260/280 nm. Right before reverse transcription (RT), RNA was linearized at a concentration of 133 ng/μL at 65 °C for 10 min and then immediately incubated on ice for at least 1 min. Reverse transcription was performed using 2 μg of total RNA, 20 U Recombinant RNasin Ribonuclease Inhibitor (Promega, Mannheim, Germany), 100 U SuperScript IV Reverse Transcriptase including 5× First Strand Buffer and 0.1 M dithiothreitol (Thermo Fisher Scientific), 3.75 ng of linear acrylamide, 625 ng of random hexamer primers (Merck, Darmstadt, Germany), and 11.25 nmol of deoxynucleoside triphosphate mix (2.8 nmol each ATP, TTP, CTP, GTP; Thermo Fisher Scientific) at a volume of 22.45 μL at 50 °C for 10 min and 80 °C for 10 min using a Thermal cycler XT96 (VWR International, Darmstadt, Germany). Quantitative polymerase chain reaction (qPCR) was conducted using a QuantStudio 1 (Applied Biosystems, Waltham, MA, USA) and a SYBR green-based detection method. 0.2 μL of prepared cDNA was added to 6 pmol each of forward and reverse primer, 0.8 U Taq DNA Polymerase (New England Biolabs, Ipswich, MA, USA), 40 ppm SYBR Green I (Sigma-Aldrich), 15 nmol of deoxynucleoside triphosphate mix (as indicated above), 60 nmol of MgCl_2_, 4 μg of bovine serum albumin (Thermo Fisher Scientific), 20% BioStab PCR Optimizer II (Merck, Darmstadt, Germany), and 10% Taq buffer without detergents (Thermo Fisher Scientific), topped up to a final volume of 20 μL with ddH_2_O. Samples underwent 40 cycles of 15 s denaturation at 95 °C, 15 s of primer annealing at primer-specific temperatures and 20 s of elongation at 68 °C. PCR product specificity was evaluated using a melting curve analysis ranging from 65 to 95 °C. Nurr1 target gene expression was normalized to rGAPDH mRNA expression per sample using the ΔCt-method. The following primers and annealing temperatures were used: rGAPDH (59.4 °C): 5’-CAG CCG CAT CTT CTT GTG C-3’ (fwd), 5’-AAC TTG CCG TGG GTA GAG TC-3’ (rev); rTH (59.4 °C): 5’-TGG GGA GCT GAA GGC TTA TG-3’ (fwd), 5’-AGA GAA TGG GCG CTG GAT AC-3’ (rev); rBIRC5/Survivin (59.4 °C): fwd 5’-TCC ACT GCC CTA CCG AGA AT-3’, rev 5’-AGG GGA GTG CTT CCT ATG CT-3’; rSOD1 (61.1 °C): 5’-GAA GGC GAG CAT GGG TTC C-3’ (fwd), 5’-CAG GTC TCC AAC ATG CCT CTC T-3’ (rev); rSOD2 (59.0 °C): fwd 5’-CGG GGG CCA TAT CAA TCA CA-3’, rev 5’-TCC AGC AAC TCT CCT TTG GG-3’; rBDNF (58.0 °C): 5’-AGT CTA GAA CCT TGG GGA CC-3’ (fwd), 5’-GCC TTC ATG CAA CCG AAG TA-3’ (rev); rSESN3 (62.4 °C): 5’-TCG GCC AAC TAC CTG CTC TG-3’ (fwd), 5’-CGT GTT TGC TTG GAC AAC TTC CT-3’ (rev).

##### Evaluation of neuroprotection in paraquat-treated N27 cells

N27 rat dopaminergic neural cells (SCC048, Sigma-Aldrich, Darmstadt, Germany) were cultured in RPMI 1640 medium (Gibco, Thermo Fisher Scientific, Waltham, MA, USA) supplemented with 10% FCS, penicillin (100 U/mL), and streptomycin (100 μg/mL) at 37 °C and 5% CO_2_ and seeded in transparent 96-well plates (6×10^4^ cells/well) that were pre-coated with a 10 μg/mL collagen G solution (Merck KgaA, Darmstadt, Germany, L7213) at 37 °C for 30 min before seeding to increase cell adherence. After 24 h, the medium was changed to RPMI 1640 medium (Gibco, Thermo Fisher Scientific) supplemented with 0.2% FCS, penicillin (100 U/mL), and streptomycin (100 μg/mL), and the cells were incubated for another 22 h, before the medium was changed again to RPMI 1640 medium (Gibco, Thermo Fisher Scientific) supplemented with 0.2% FCS, penicillin (100 U/mL), and streptomycin (100 μg/mL), additionally containing either **37** (0.3, 1 or 3 μM) solubilized with 0.1% DMSO or 0.1% DMSO alone as negative control. After 24 h of incubation, the medium was changed to serum-free RPMI 1640 medium (Gibco, Thermo Fisher Scientific) supplemented with penicillin (100 U/mL) and streptomycin (100 μg/mL), additionally containing either **37** (0.3, 1 or 3 μM) solubilized with 0.1% DMSO or 0.1% DMSO alone as negative control as well as either 600 μM paraquat (PQ) in serum-free RPMI 1640 medium to induce neurotoxicity or serum-free RPMI 1640 medium alone as unstressed control. Each condition was tested in five biologically independent repeats. After another 4 h, medium was changed to phenol red-free RPMI 1640 medium (Gibco, Thermo Fisher Scientific) supplemented with 0.2% FCS, penicillin (100 U/mL), and streptomycin (100 μg/mL) and 10 μL of Cell Counting Kit-8 solution (CCK-8, MedChem Express LLC, Monmouth Junction, NJ, USA, #HY-K0301) were added to each well, and absorbance at 450 nm was measured after 5.5 h on a Tecan Spark Cyto multiplate reader (Tecan Group AG, Männedorf, Switzerland). Cells were then co-stained with Hoechst 33342 (4 μM, abcam, Cambridge, UK, ab228551) and Live-or-Dye Nuc-Fix Red (0.05x, Biotium, Inc., Fremont, USA, #32010) to determine total cell count and detect necrotic cells, respectively. After light-protected incubation for 45 min, 5 fluorescence images per well at 10x magnification were taken to detect Hoechst 33342-positive (Ex: 381–400 nm, Em: 414–450 nm) and Live-or-Dye-positive cells (Ex: 543−566 nm, Em: 580−611 nm) using a Tecan Spark Cyto multiplate reader and corresponding algorithms, yielding blue (BOC) and red object counts (ROC), respectively. Relative necrosis was then calculated as the quotient of ROC/BOC.

##### Determination of aqueous solubility

Aqueous solubility was assessed by mixing 1 mg of the test compound with an appropriate volume of water for a theoretical concentration of 4 mM to obtain an oversaturated mixture. The mixture was agitated in a VWR Thermal Shake lite (VWR International GmbH, Darmstadt, Germany) for 48 h at 600 rpm and constant temperature of 25 °C. The supersaturated mixtures were subsequently centrifuged at 25.200 g for 15 min (25 °C). Part of the supernatant was taken off for quantification by UV absorbance with external calibration. The external calibration samples contained 1% DMSO and the test samples were spiked with DMSO to 1% concentration right before the measurement. Absorbance was measured with a Tecan Spark luminometer using a Quartz 96-well plate (Tecan Deutschland GmbH, Crailsheim, Germany). The solubility test was repeated in three independent experiments.

##### Multiplex cytotoxicity assay

HEK293T cells (DSMZ #ACC305) or COS-7 cells (DSMZ #ACC 60) were cultured at 37°C and 5% CO_2_ in DMEM high glucose medium supplemented with sodium pyruvate (1 mM), penicillin (100 U/mL), streptomycin (100 μg/mL), and 10% FCS. To increase adherence of HEK293T cells, the respective 96-well plates were pre-coated with a 10 μg/mL collagen G solution (Merck KgaA, L7213) at 37°C for 30 min before seeding. Cells were seeded at 20,000 (HEK293T) or 10,000 (COS-7) cells per well in complete culture medium. After 24 hours, the cells were treated with the respective test compounds in DMEM high glucose medium supplemented with 0.1% FCS, penicillin (100 U/mL), streptomycin (100 μg/mL) and 0.1% DMSO, or 0.1% DMSO alone as an untreated control. Each sample was prepared in at least three biologically independent replicates. After incubation for 24 h, the medium was removed and the cells were incubated for 30 min with staining medium containing 1 μM NucView® 405 fluorogenic caspase-3 substrate (#10405, Biotium, Fremont, USA) and Live-or-Dye Nuc-Fix Red (0.05x, Biotium, Inc., Fremont, CA, 1691 USA) to detect apoptosis and necrosis, respectively. After incubation, a total of 6 fluorescence images per well at 10X magnification were taken to detect NucView®-positive (Ex: 381–400 nm, Em: 414–450 nm) and Live-or-Dye-positive cells (Ex: 543−566 nm, Em: 580−611 nm) using on a Tecan Spark Cyto (Tecan Group AG). Reference readings for background correction and detection of auto-fluorescence were taken at 414–450 nm prior to staining. Thereafter, the staining medium was removed and cells were incubated for 3 h with 90 μL culture medium (0.2% FCS) and 10 μL Cell Counting Kit-8 solution (CCK-8, MedChem Express #HY-K0301), and absorbance was measured at 1 h, 2 h, and 3 h of incubation at 450 nm on a Tecan Spark Cyto to assess metabolic activity of the cells. Before drug administration, after the first medium exchange, 24 h after drug administration, and after fluorescence imaging cell confluence was assessed using the Tecan Spark Cyto, to account for changes in cell confluence due to drug administration and cell handling. The number of apoptotic cells, metabolic activity, and changes in cell confluence in response to drug treatment were all normalized to the DMSO control of the respective biological replicate.

##### Evaluation of microsomal stability

To determine microsomal stability, test compounds (10 μM) were incubated in 100 mM potassium phosphate buffer at pH 7.4 (total volume 100 μL) containing 0.5 mg/mL male rat liver microsomes (Sprague-Dawley, no. M9066, Merck KGaA, Darmstadt) and 1 mM NADPH at 37 °C and constant shaking for 0, 15, 30, or 60 min. At the end of the incubation time, microsomal activity was terminated by the addition of 500 μL MeCN and subsequent centrifugation at 1700g for 5 min. A reaction mixture containing heat-inactivated microsomes (95 °C, 10 min) was prepared as control for each compound. 250 μL supernatant were diluted with 250 μL mobile phase. 10 μL of this dilution were analyzed and the remaining concentration of the respective test compound at each time point was determined by LC-MS/MS on an API-3200-QTrap (Sciex) with an Agilent Technologies 1100 series setup including a binary pump (G1311A), a degasser (G1322A), and a Shimadzu SIL 20A HT autosampler under the control of Analyst 1.6 (Sciex). A ZORBAX SB-Aq (3.5 μm, 3.0 x 100 mm, protected with a 0.5 μm frit and a 0.2 μm frit) stationary phase was used in combination with 0.1% formic acid in H_2_O/ MeCN (4:6) as mobile phase at a flow rate of 400 μL/min. The analytes were detected and quantified per Area of MRM (multiple reaction monitoring) with the following transitions: *m/z* 321.9/145.0 (**37**); m/z 260.2/116.1 (propranolol).

#### Computational Methods

##### Docking

The Nurr1 structure 6dda^19^ was taken from the PDB, chain B and C and all ions and water molecules were deleted. The covalently bound ligand DHI was removed, and protonation and partial charges of the remaining protein were calculated using the Schrödinger Protein Preparation Wizard. The ligand **37** was converted into 3D and the partial charges were calculated with LigPrep using Epik^44^. Docking was performed with GLIDE^45^ using standard precision (SP). All GLIDE parameters were left at their default values.

## Supplementary Material

Supporting info.

## Figures and Tables

**Table 1:** Deconstruction of the 5-chloro-2-(2-(dimethylamino)ethoxy)aniline substituent

ID	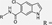	EC_50_(Nurr1) (max. activation) ^a^
**4**	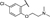	3±1 μM (1.3±0.1-fold)
**5**	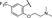	inactive (10 μM)
**6**	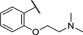	inactive (10 μM)
**7**	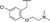	inactive (10 μM)
**8**	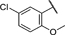	0.06±0.03 μM (1.5±0.1-fold)
**9**	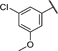	0.7±0.1 μM (1.4±0.1-fold)
**10**	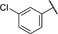	0.6±0.2 μM (1.5±0.1-fold)
**11**	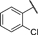	1.5±0.1 μM (1.6±0.1-fold)
**12**	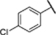	0.08±0.02 μM (1.4±0.1-fold)
**13**	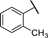	inactive (10 μM)
**14**	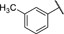	0.5±0.2 μM (1.5±0.1-fold)
**15**	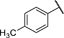	0.33±0.09 μM (1.6±0.1-fold)
**16**	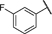	1.0±0.2 μM (1.4±0.1-fold)
**17**	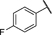	1.0±0.2 μM (1.3±0.1-fold)
**18**	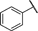	1.7±0.5 μM (1.3±0.1-fold)

a Nurr1 modulation was determined in a Gal4-Nurr1 hybrid reporter gene assay. Max. activation refers to the maximum effect vs. 0.1% DMSO control. Data are the mean±SD; n≥3.

**Table 2:** Evaluation of alternative m/p-substituents

ID	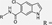	EC_50_(Nurr1) (max. activation) ^a^
**19**	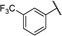	inactive (10 μM)
**20**	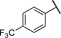	1.1±0.2 μM (1.4±0.1-fold)
**21**	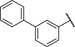	inactive (10 μM)
**22**	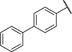	inactive (10 μM)
**23**	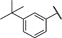	inactive (10 μM)
**24**	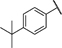	0.26±0.04 μM (1.6±0.1-fold)
**25**	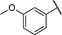	0.5±0.2 μM (1.4±0.1-fold)
**26**	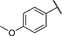	0.04±0.01 μM (1.5±0.1-fold)
**27**	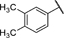	0.32±0.08 μM (1.4±0.1-fold)
**28**	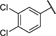	0.7±0.1 μM (1.9±0.1-fold)
**29**	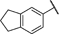	0.07±0.02 μM (1.5±0.1-fold)
**30**	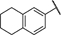	0.9±0.2 μM (1.5±0.1-fold)
**31**	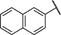	0.2±0.1 μM (1.5±0.1-fold)

a Nurr1 modulation was determined in a Gal4-Nurr1 hybrid reporter gene assay. Max. activation refers to the maximum effect vs. 0.1% DMSO control. Data are the mean±SD; n≥3.

**Table 3:** SAR of bicyclic amide substituents

ID	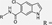	EC_50_(Nurr1) (max. activation) ^a^
**32**	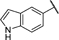	0.4±0.1 μM (2.0±0.1-fold)
**33**	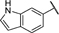	0.12±0.04 μM (1.5±0.1-fold)
**34**	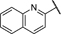	0.10±0.03 μM (1.8±0.1-fold)
**35**	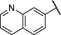	0.13±0.04 μM (1.7±0.1-fold)
**36**	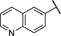	0.13±0.04 μM (1.8±0.1-fold)
**37**	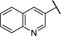	0.06±0.02 μM (1.9±0.1-fold)
**38**	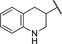	1.4±0.5 μM (1.5±0.1-fold)
**39**	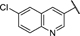	inactive (10 μM)
**40**	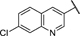	inactive (10 μM)
**41**	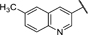	inactive (10 μM)
**42**	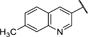	0.5±0.2 μM (1.4±0.1-fold)
**43**	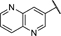	0.24±0.07 μM (1.5±0.1-fold)
**44**	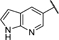	0.31±0.05 μM (2.1±0.1-fold)
**45**	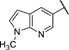	0.4±0.2 μM (2.1±0.1-fold)
**46**	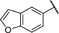	0.10±0.04 μM (2.0±0.1-fold)
**47**	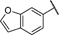	0.10±0.04 μM (1.5±0.1-fold)

a Nurr1 modulation was determined in a Gal4-Nurr1 hybrid reporter gene assay. Max. activation refers to the maximum effect vs. 0.1% DMSO control. Data are the mean±SD; n≥3.

**Table 4:** Modification of the amide linker

ID		EC_50_(Nurr1) (max. activation) ^a^
**37**	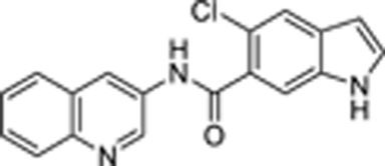	0.06±0.02 μM (1.9±0.1-fold)
**48**	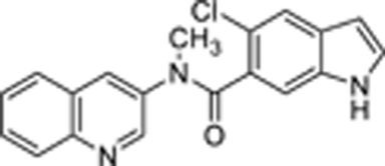	inactive (10 μM)
**49**	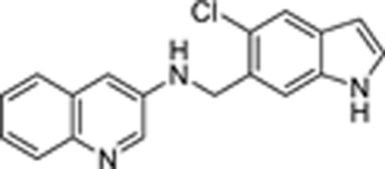	1.2±0.2 μM (1.5±0.1-fold)
**50**	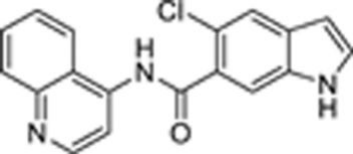	inactive (10 μM)
**51**	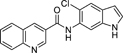	0.21±0.07 μM (1.6±0.1-fold)

a Nurr1 modulation was determined in a Gal4-Nurr1 hybrid reporter gene assay. Max. activation refers to the maximum effect vs. 0.1% DMSO control. Data are the mean±SD; n≥3.

**Table 5:** Characteristics of the Nurr1 agonist 37

	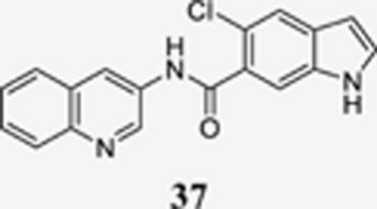
EC_50_ (Nurr1) ^a^	0.06±0.02 μM
efficacy ^a^	1.9±0.1-fold
Kd (Nurr1) ^b^	0.12 μM
EC_50_ (NBRE) ^a^	0.07±0.02 μM
EC_50_ (NurRE) ^a^	0.027±0.008 μM
EC_50_ (DR5) ^a^	0.014±0.006 μM
EC_50_ (Nur77) ^a^	0.04±0.01 μM
EC_50_ (NOR1) ^a^	0.07±0.03 μM
selectivity (NR1-3) ^a^	> 50-fold
toxicity ^c^	> 10 μM
logP ^d^	2.83±0.02
aq. sol. ^e^	4.4 mg/L (13.8 μM)
microsomal half-life	> 4 h
LE ^f^	0.43
LLE ^e^	4.4

a From reporter gene assay; data are the mean±S.E.M., n≥3.

b From ITC.

c From a multiplex toxicity assay monitoring confluence, metabolic activity, necrosis and apoptosis in HEK293T and COS-7 cells.

d The logP was experimentally determined following the OECD test guideline #117.

e Refers to thermodynamic solubility.

f Ligand efficiency (LE) and lipophilic ligand efficiency (LLE) were calculated according to ^38^.

## Abbreviations

AD: Alzheimer’s disease
AQ: amodiaquine
BDNF: brain derived neurotrophic factor
DHI: 5,6-dihydroxyindole
LE: ligand efficiency
LLE: lipophilic ligand efficiency
Nurr1: nuclear receptor related 1
PD: Parkinson’s disease
ROS: reactive oxygen species
SENS3: sestrin 3
SOD1: superoxide dismutase 1
TCFH: *N*-[chloro(dimethylamino)methylene]-*N*-methylmethanaminium
TH: tyrosine hydroxylase
