# Supplementary material for: A Nurr1 agonist derived from the natural ligand DHI induces neuroprotective gene expression
Source: J Med Chem. Author manuscript; Available in PMC 2025 Mar 24. (PMC7617521; doi:10.1021/acs.jmedchem.4c03104)
Supplement: Supporting info. [file EMS203174-supplement-Supporting_info_.pdf]

- Supporting Information -

A Nurr1 agonist derived from the natural ligand DHI induces neuroprotective gene expression

Markus Egner[1], Romy Busch[1], Úrsula López-García[1], Max Lewandowski[1], Georg Höfner[1], Thomas Wein[1], Julian A. Marschner[1], Daniel Merk[1]*

[1] Ludwig-Maximilians-Universität (LMU) München, Department of Pharmacy, 81377 Munich, Germany

* daniel.merk@cup.lmu.de

Table of Contents

Figures S1-S3 .... S2

LogP determination S3

NMR and HRMS spectra and purity analysis of **5-51** S4

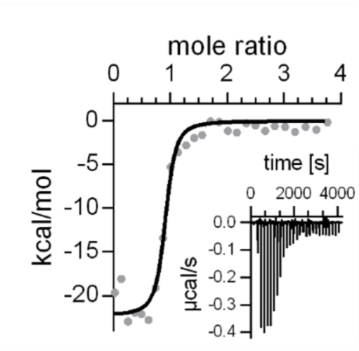

Figure S1. Isothermal titration calorimetry (ITC) demonstrated high affinity (K_d 0.08 µM) binding of **29** to the recombinant Nurr1 LBD. The fitting of the heat of binding is shown and the isotherm at 25°C is shown as inset.

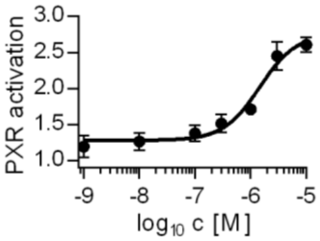

Figure S2. PXR activation by **37** (EC_{50} = 1.5±0.6 µM) in a Gal4-PXR hybrid reporter gene assay. Data are the mean±S.E.M.; n≥3.

	logP	RT 1	RT 2	k(mean)
triphenylamine	5.70	21.18	21.48	1.226
bromobenzoate	3.00	4.15	4.09	0.444
benzonitrile	1.60	2.53	2.54	0.143
ethyl benzoate	2.60	3.73	3.75	0.391
benzyl benzoate	4.00	7.43	7.47	0.741

**34**	n1	n2	n3	n4
RT	3.94	3.95	3.87	3.87
k	0.42	0.43	0.42	0.42
k(mean)	0.42			
logP	2.83			

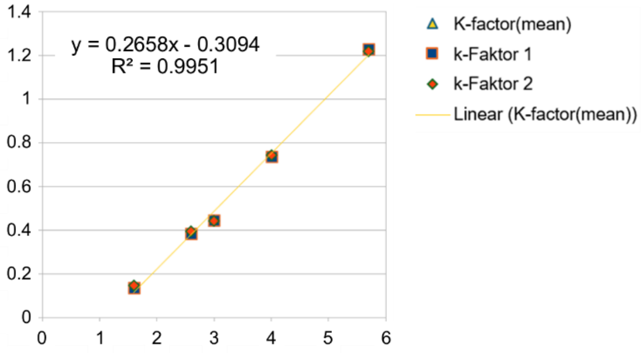

Figure S3. LogP determination for **37** by HPLC using benzonitrile, ethyl benzoate, bromobenzene, benzyl benzoate and triphenylamine as reference compounds according to the OECD test guideline #117 (June, 30th 2022).

LogP determination

The logP of **37** was experimentally determined by HPLC in accordance with the OECD test guideline #117 (June, 30th 2022) using benzonitrile, ethyl benzoate, bromobenzene, benzyl benzoate and triphenylamine as reference compounds, spanning an OECD documented logP range from 1.6 to 5.7. Test and reference compounds were diluted to a final concentration of 0.1 mg/mL in mobile phase consisting of acetonitrile (45%) and phosphate buffer at pH 7.6 (55%). All samples were spiked with thiourea to a final concentration of 0.5 mg/mL to detect dead time t_0. Separation and detection was achieved using liquid chromatography on an Agilent Technologies 1100 series setup including a binary pump (G1312A), a degasser (G1379A), a Variable Wavelength Detector (G1314A), and an autosampler (G1329A) with a ZORBAX SB-Aq (3.5 µm, 3.0 x 100 mm, protected with a 0.5 µm frit) stationary phase in combination with the mobile phase described above under isocratic conditions. 10 µL of aspirated supernatant was loaded onto the column, separated at a flow rate of 400 µL/min, and detected and quantified via absorbance at a UV-wavelength suitable for the respective compound. Measurements for **37** were performed in four independent experiments and logP was calculated from the capacity factor via a linear regression model derived from the reference compounds with $R^2 = 0.995$ (Figure S2).

NMR and HRMS spectra and purity analysis of **5-51**

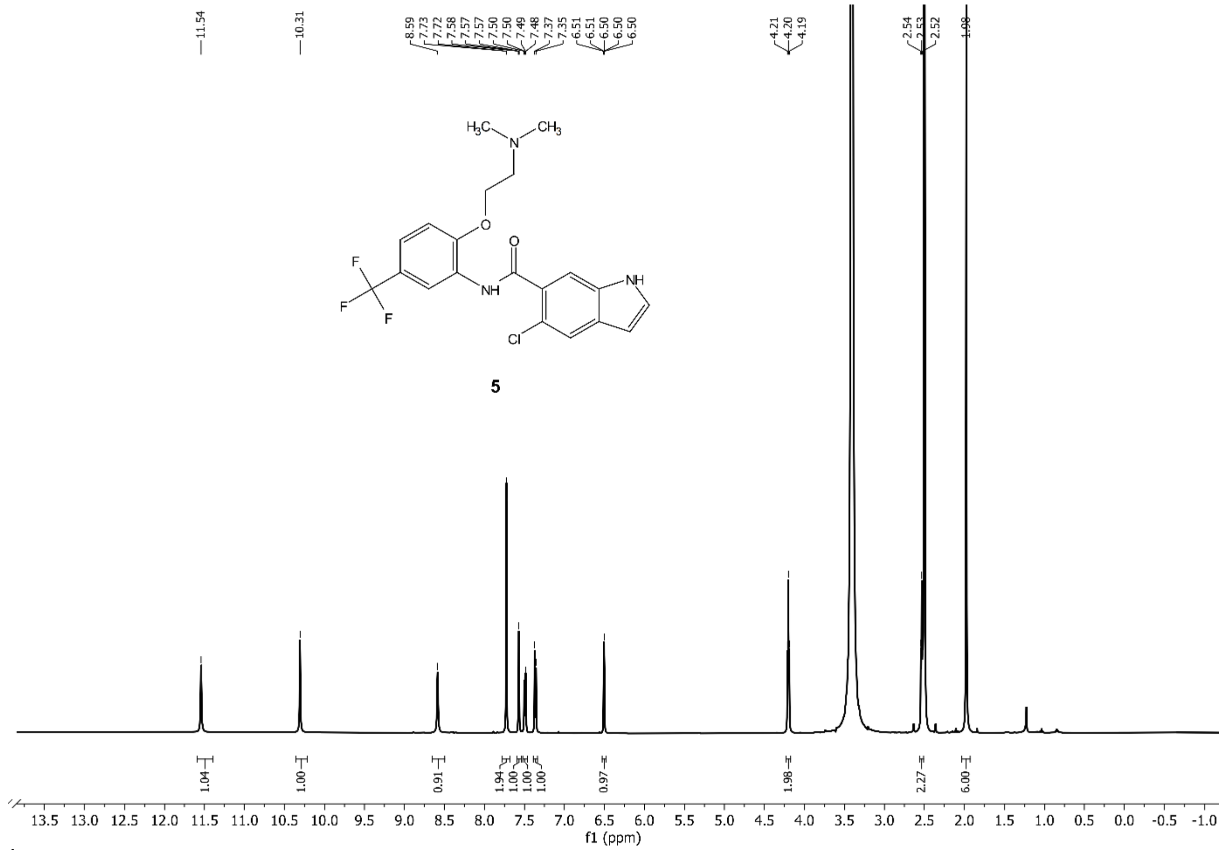

^{1}H-NMR (500 MHz, DMSO-d$_6$) of compound **5**.

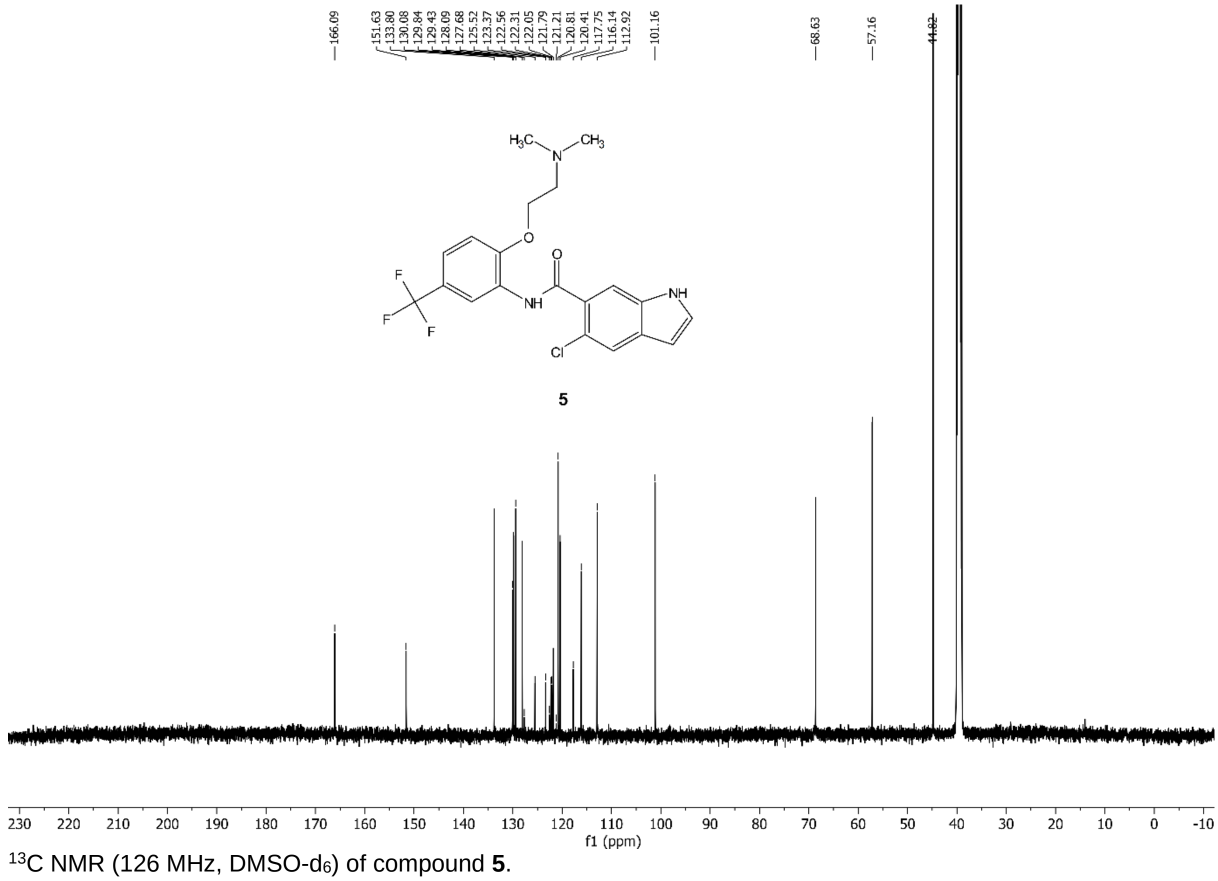

^{13}C NMR (126 MHz, DMSO-d$_6$) of compound **5**.

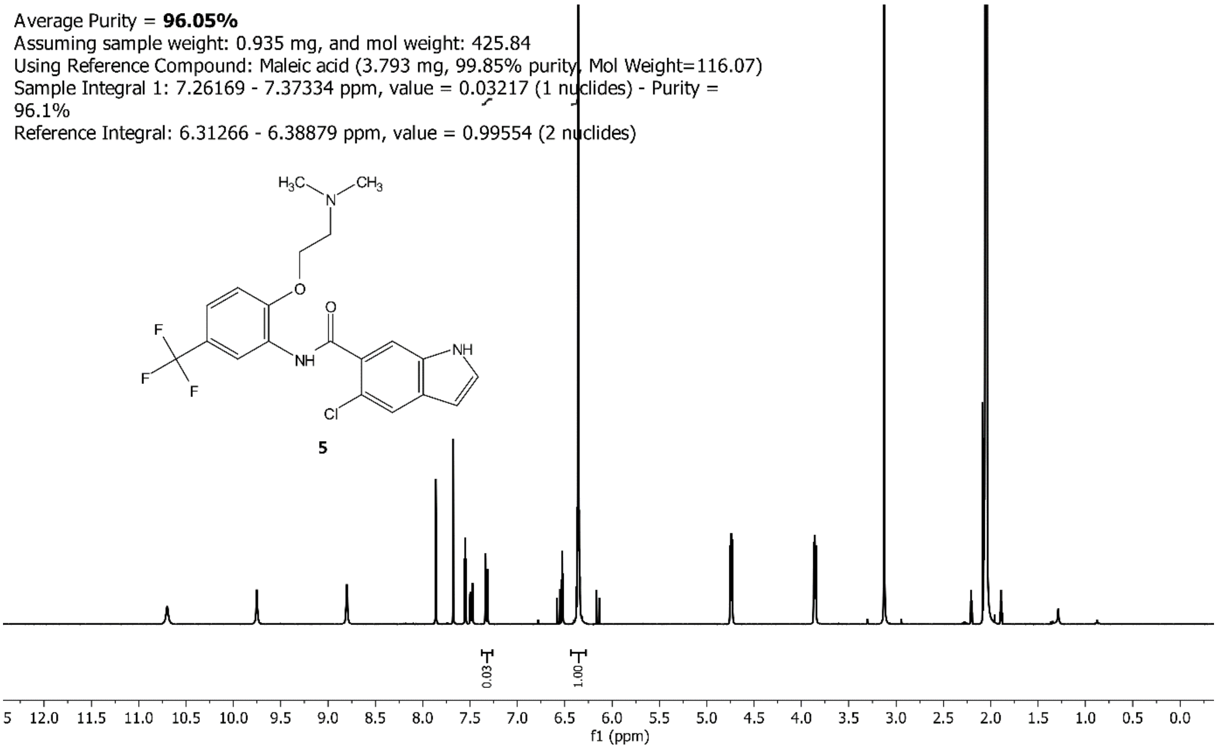

qHNMR (400 MHz, acetone-d_6, maleic acid as reference) of compound **5**.

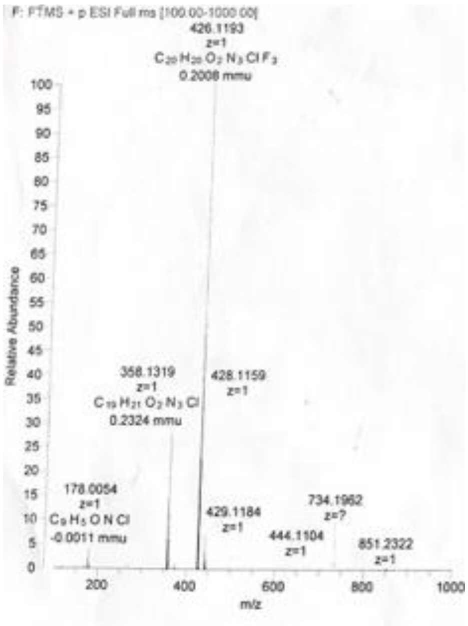

HRMS (ESI+) of compound **5**.

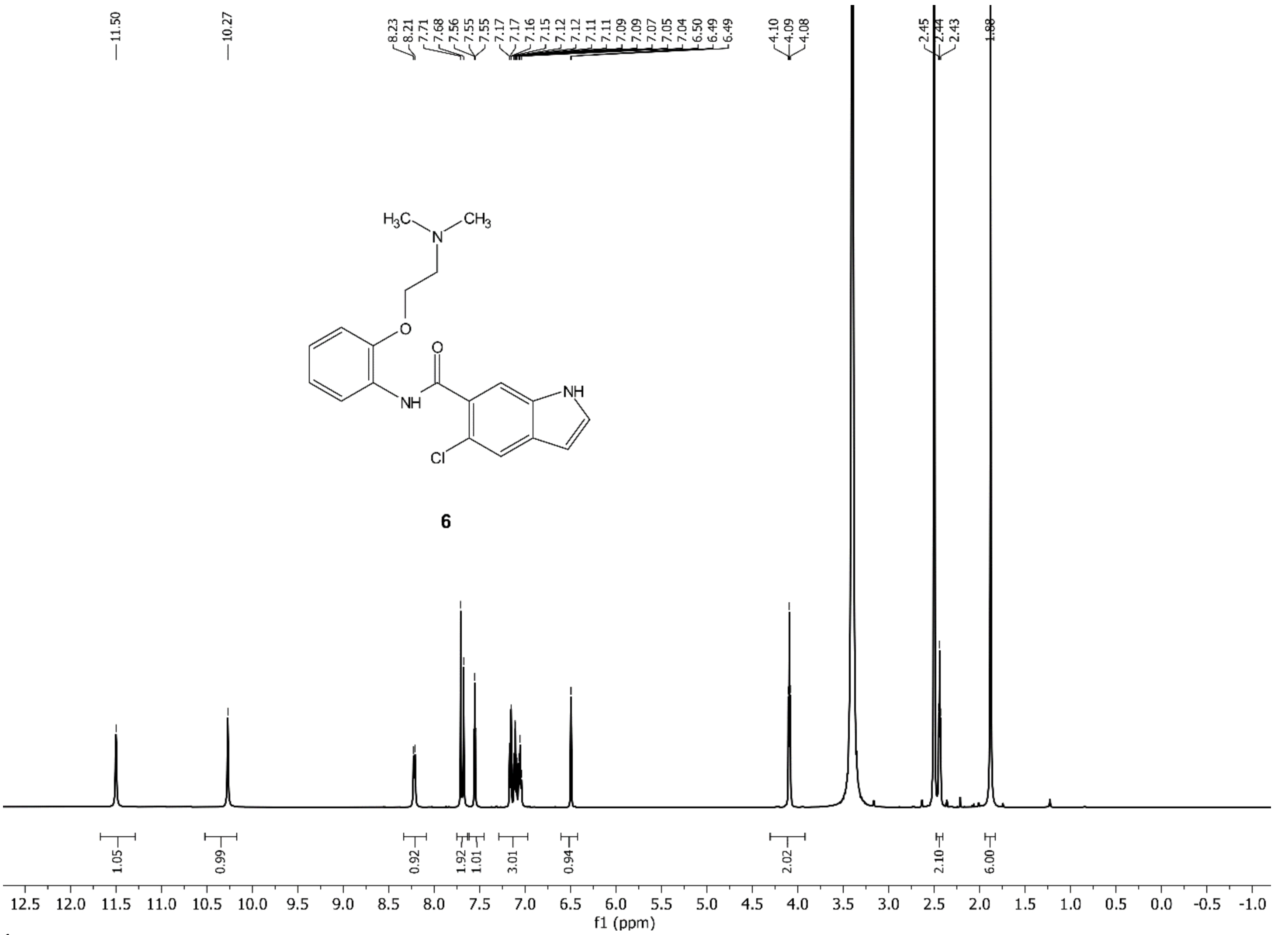

^{1}H-NMR (500 MHz, DMSO-d6) of compound **6**.

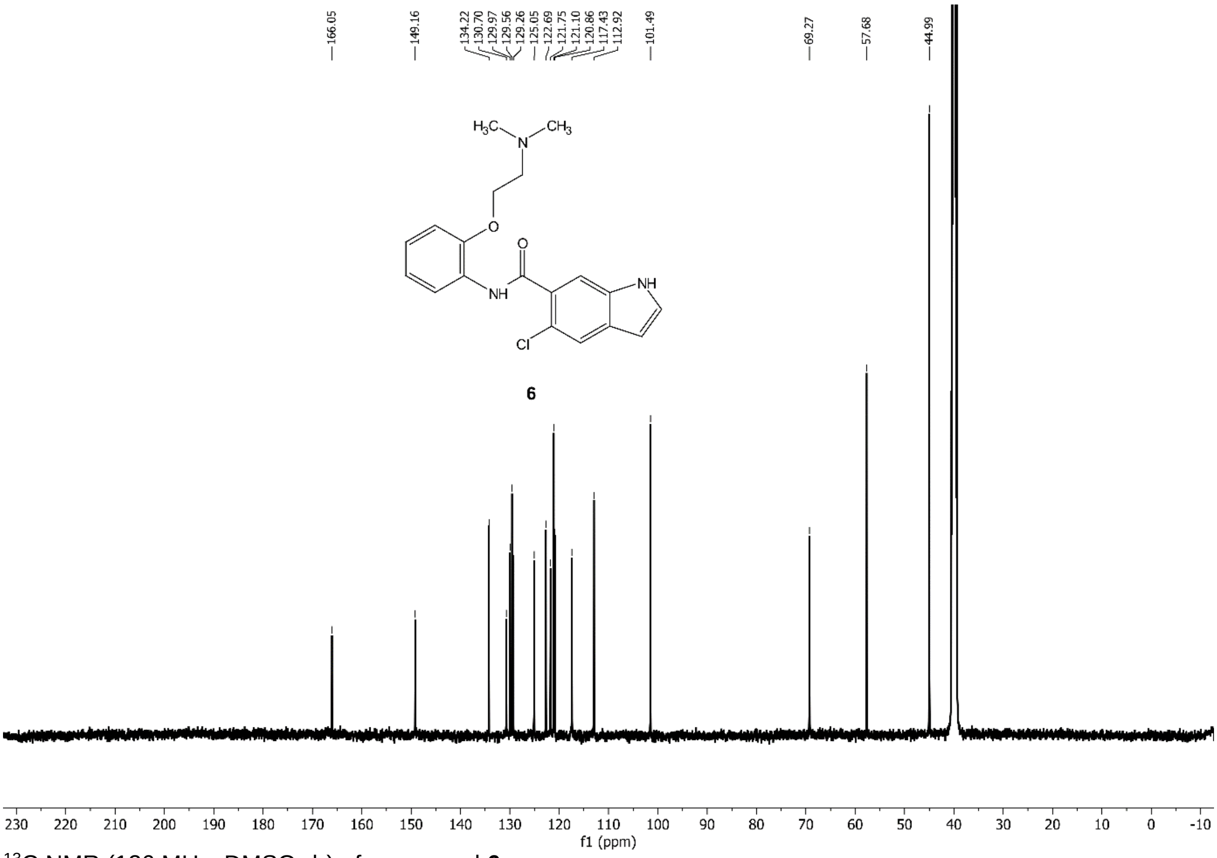

^{13}C NMR (126 MHz, DMSO-d6) of compound **6**.

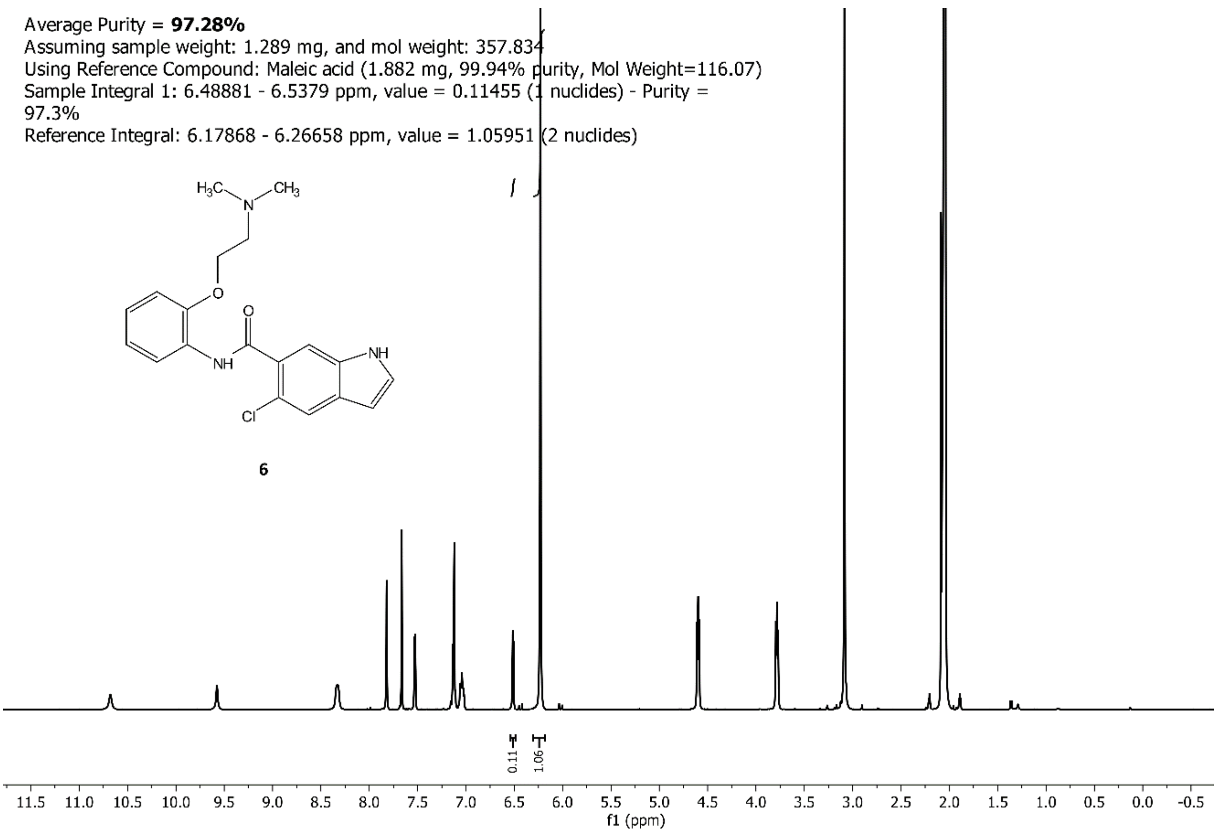

qHNMR (400 MHz, acetone-d_6, maleic acid as reference) of compound **6**.

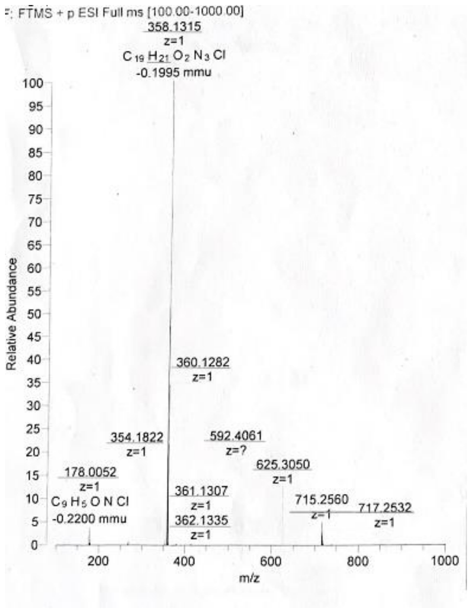

HRMS (ESI+) of compound **6**.

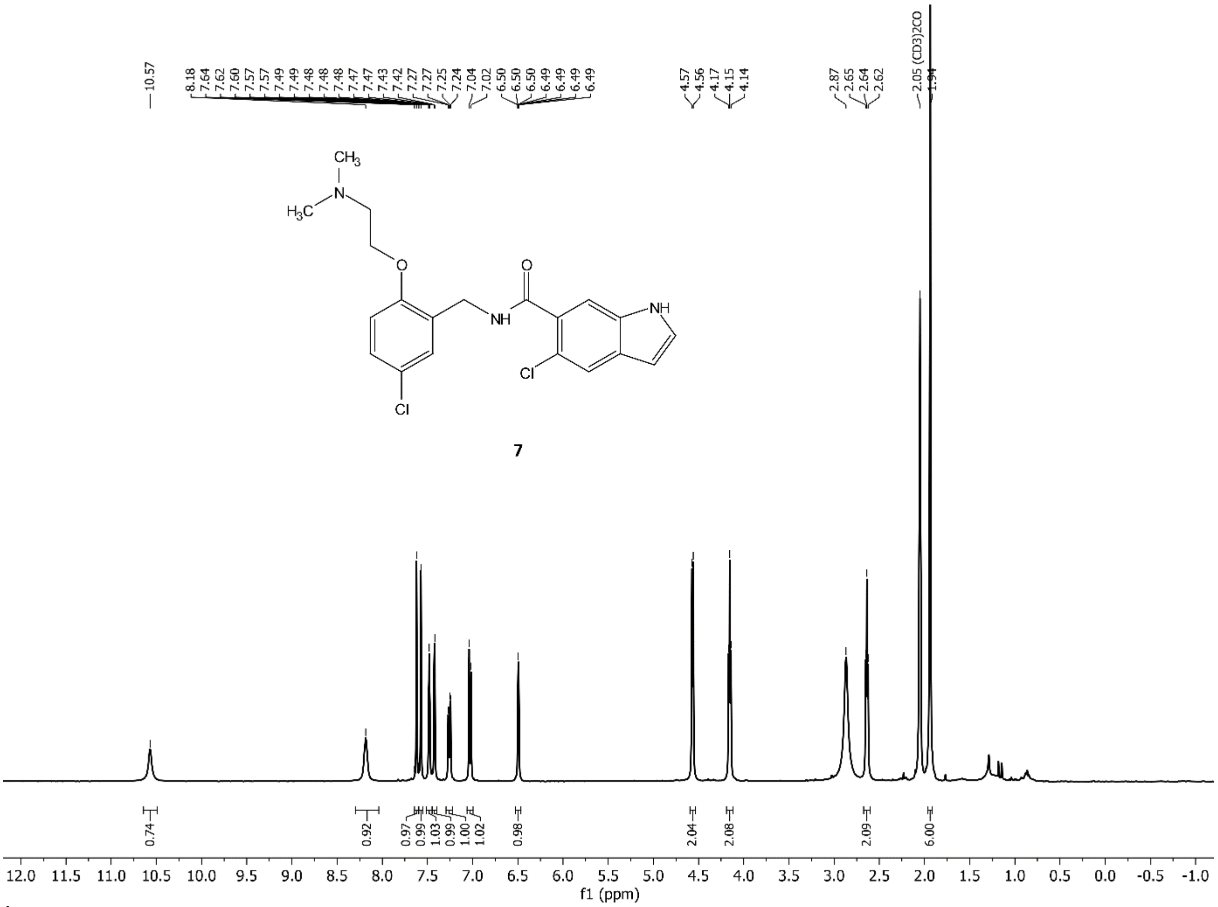

^{1}H-NMR (400 MHz, acetone-d6) of compound **7**.

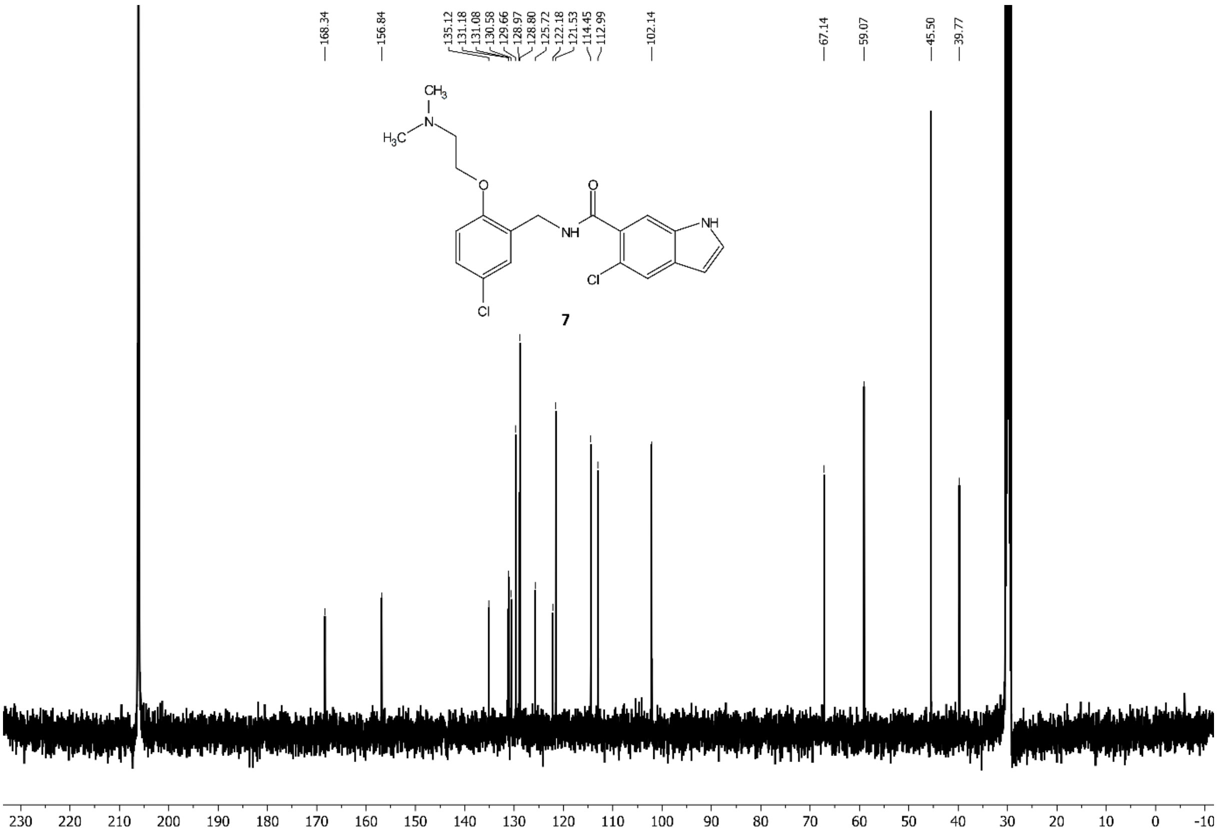

^{13}C-NMR (101 MHz, acetone-d6) of compound **7**.

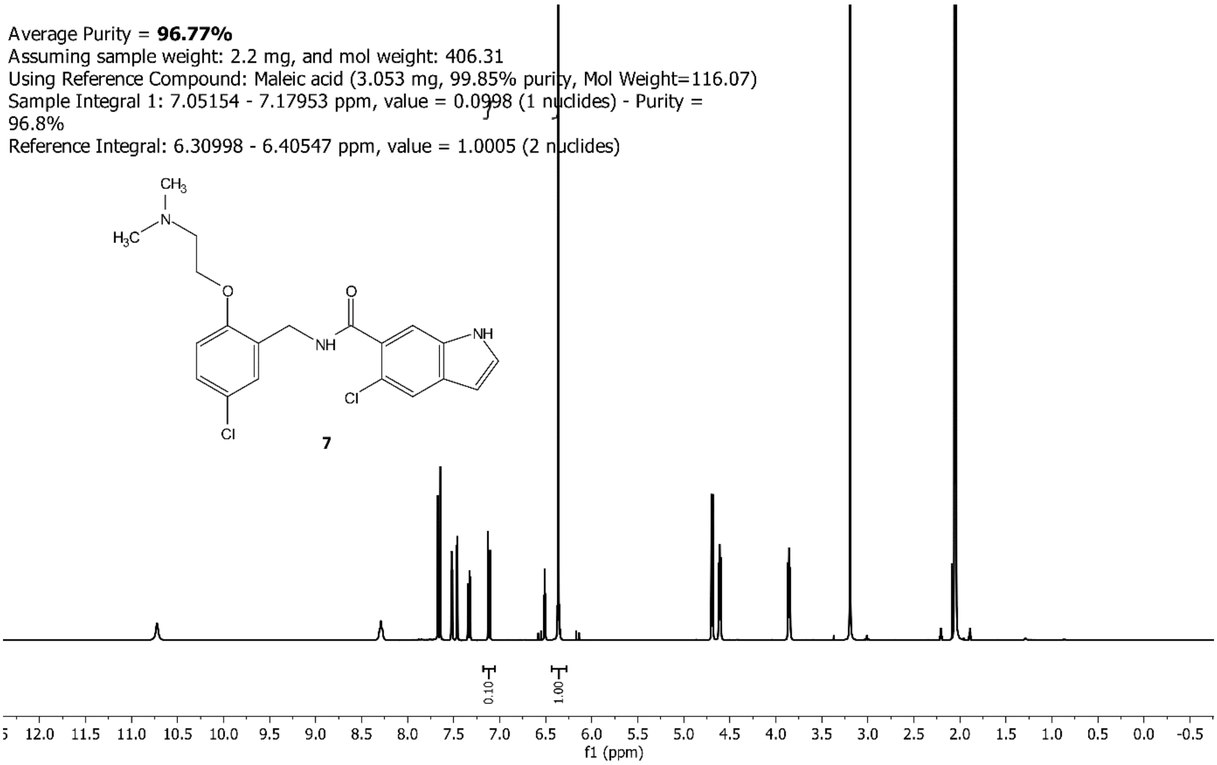

qHNMR (400 MHz, acetone-d6, maleic acid as reference) of compound **7**.

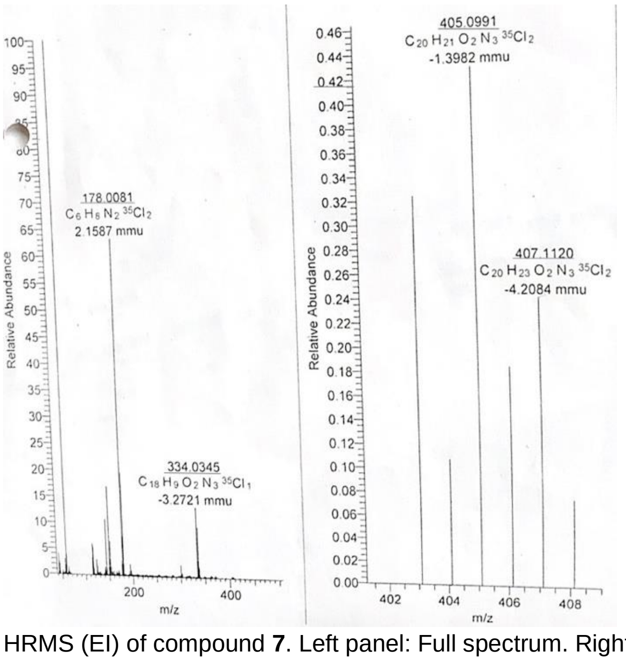

HRMS (EI) of compound **7**. Left panel: Full spectrum. Right panel: Zoom in.

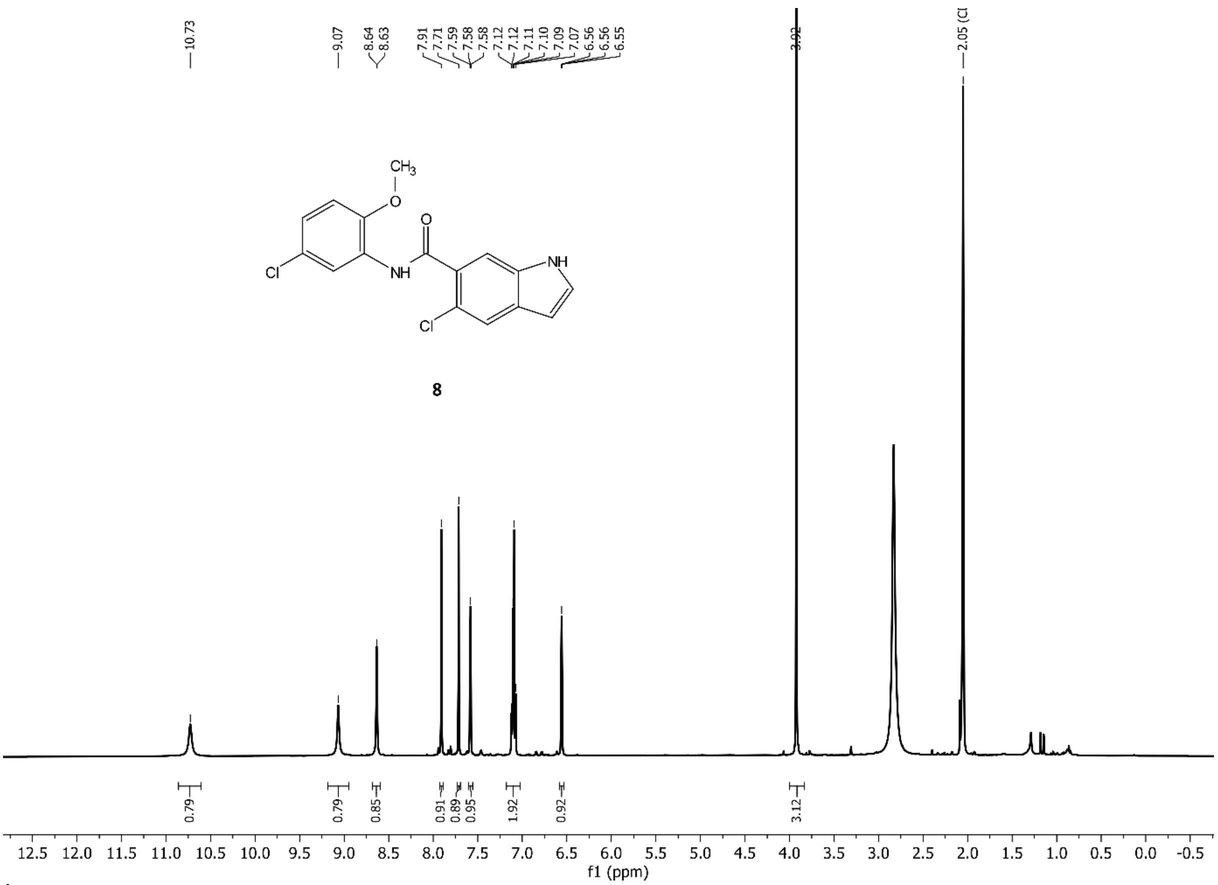

^{1}H-NMR (500 MHz, acetone-d$_6$) of compound **8**.

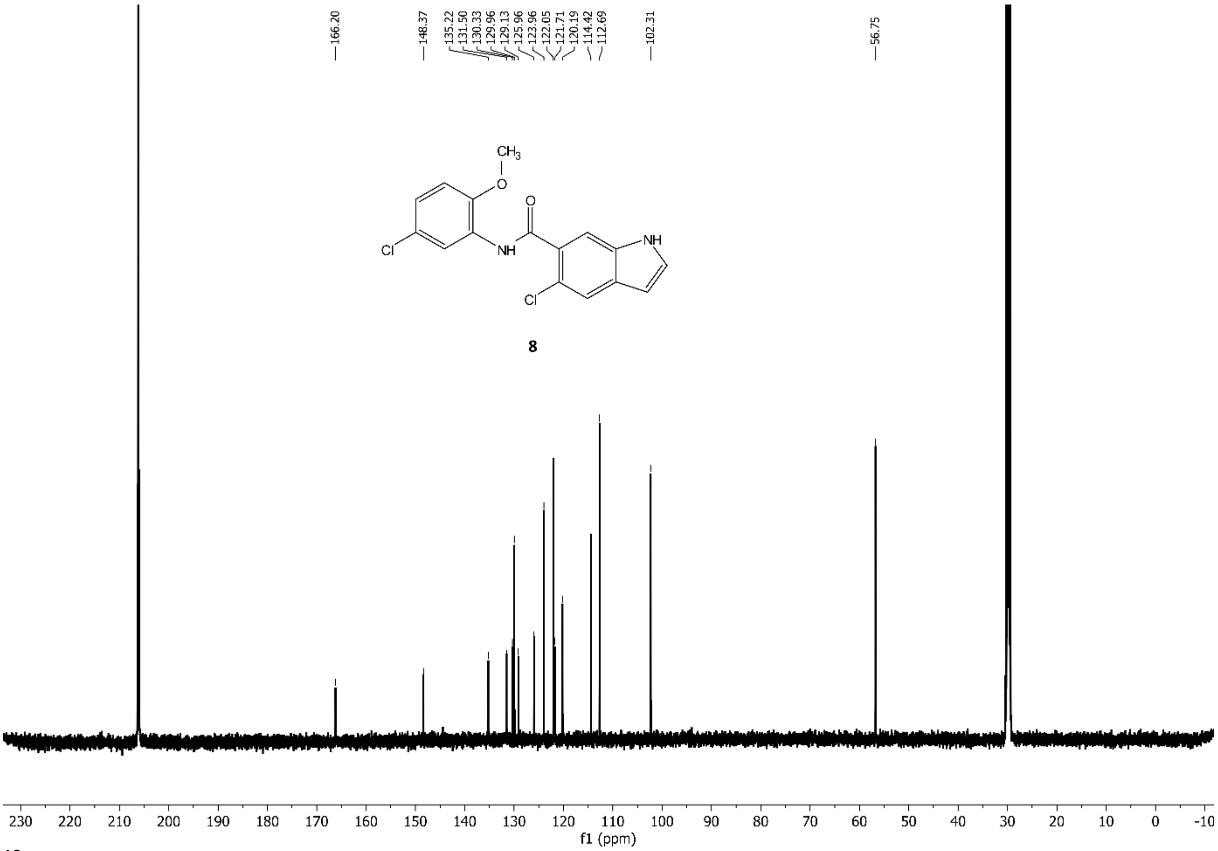

^{13}C-NMR (126 MHz, acetone-d$_6$) of compound **8**.

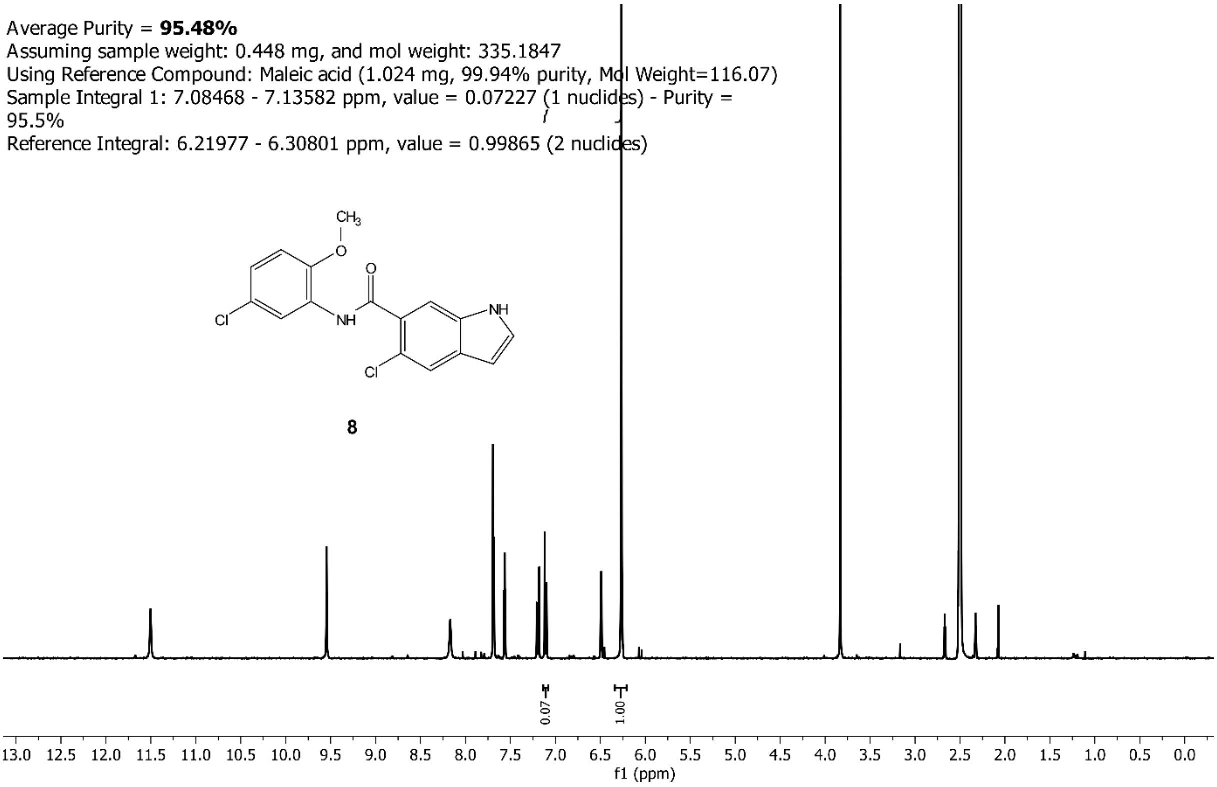

qHNMR (400 MHz, DMSO-d6, maleic acid as reference) of compound **8.**

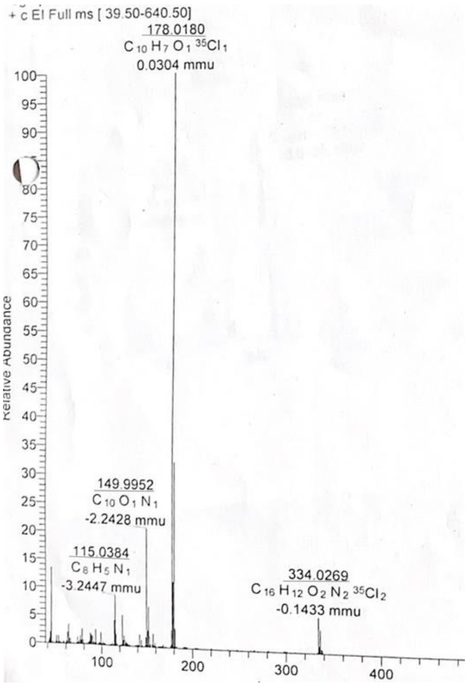

HRMS (EI) of compound **8**.

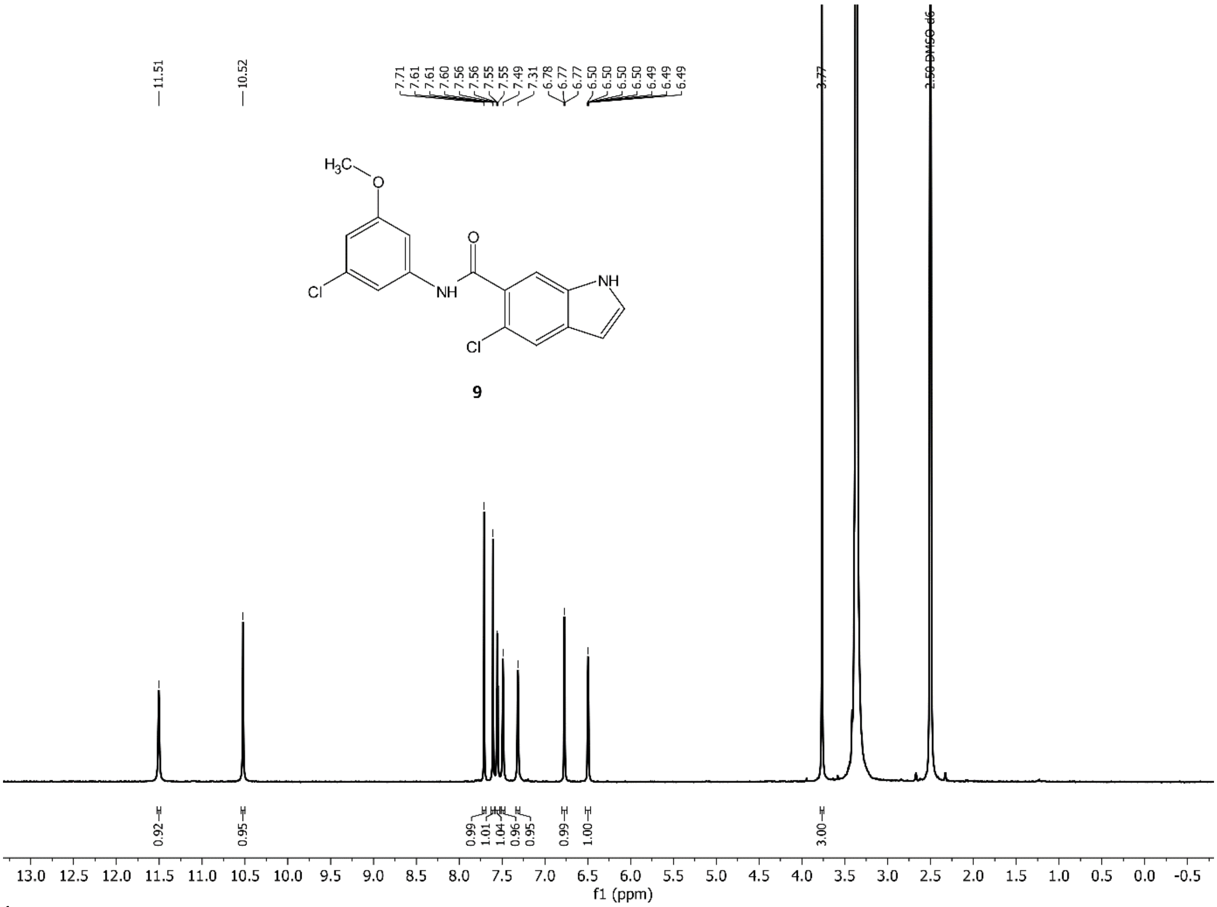

^{1}H-NMR (400 MHz, DMSO-d_6) of compound **9**.

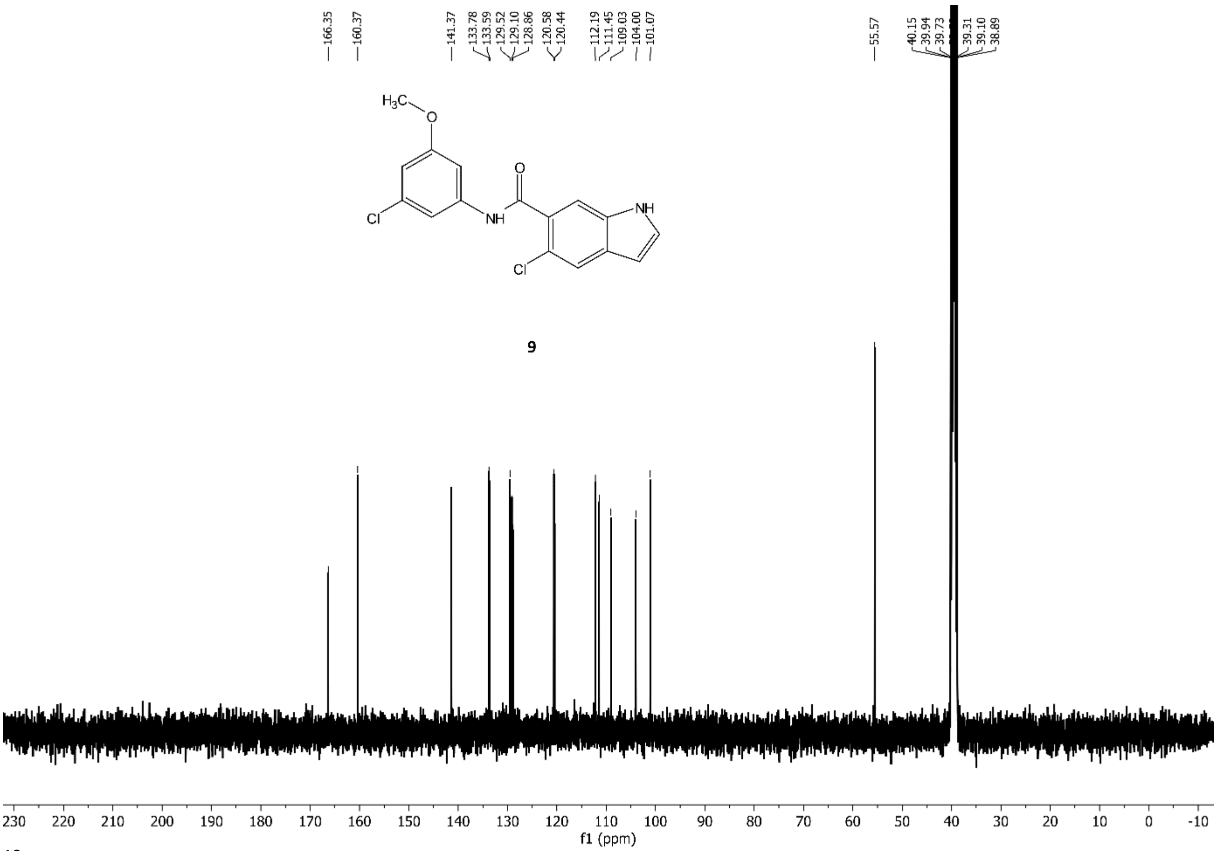

^{13}C-NMR (101 MHz, DMSO-d_6) of compound **9**.

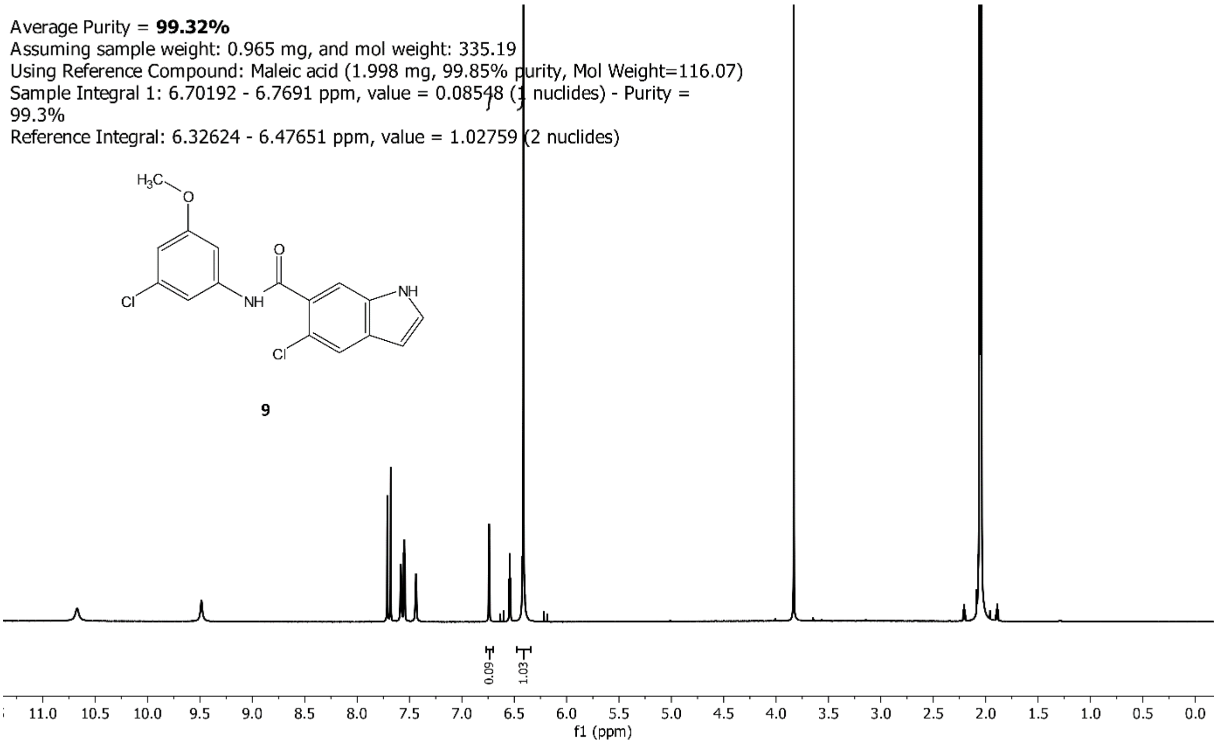

qHNMR (400 MHz, acetone-d_6, maleic acid as reference) of compound **9**.

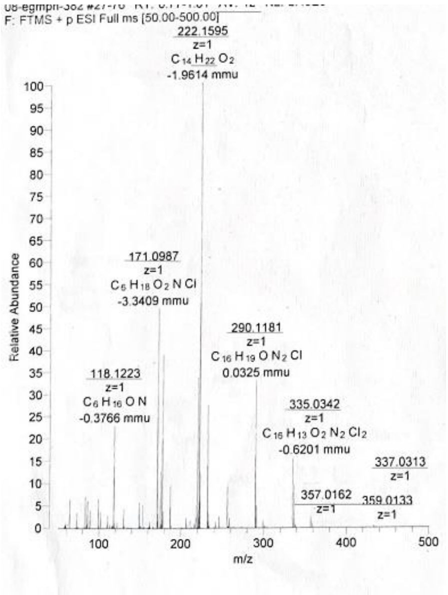

HRMS (ESI+) of compound **9**.

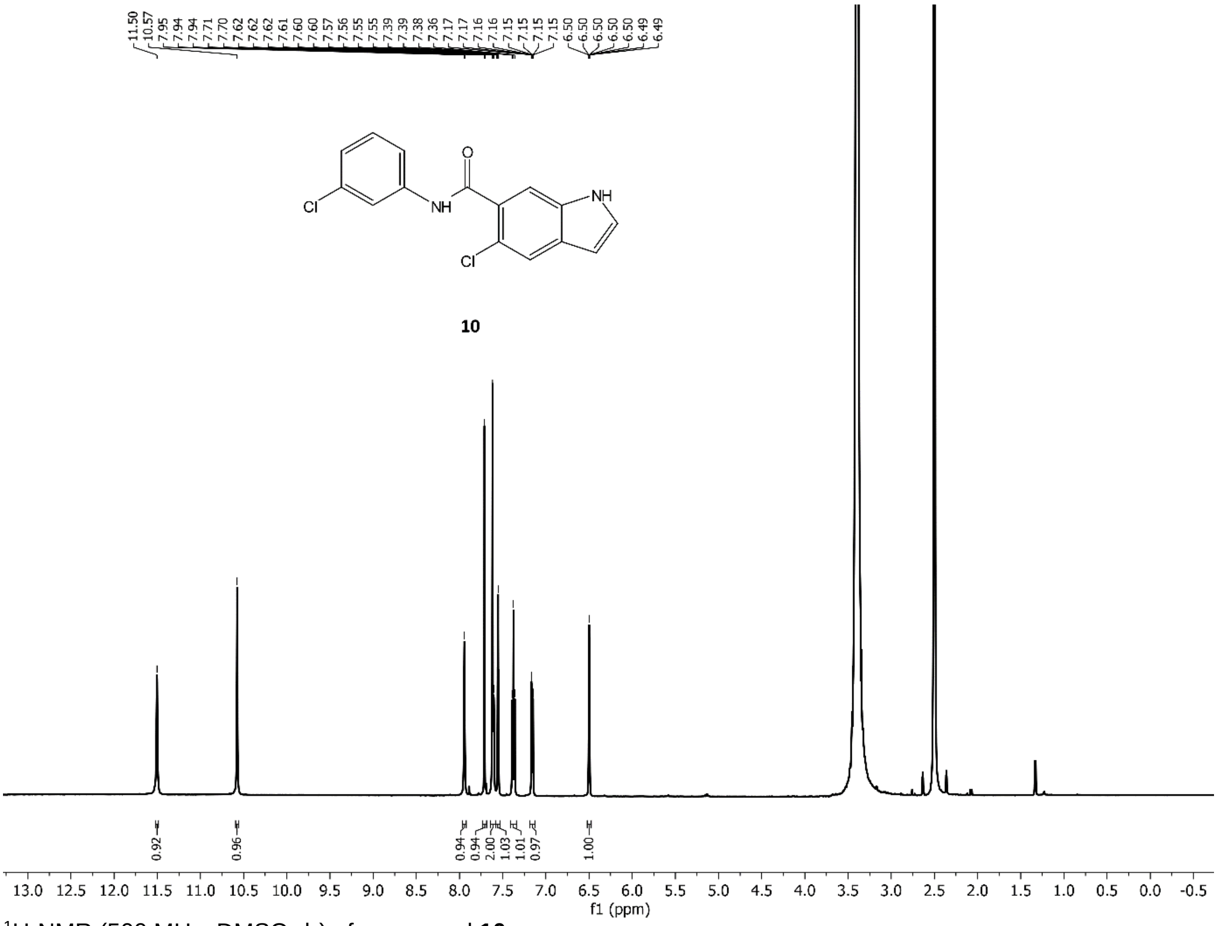

^{1}H-NMR (500 MHz, DMSO-d_6) of compound **10**.

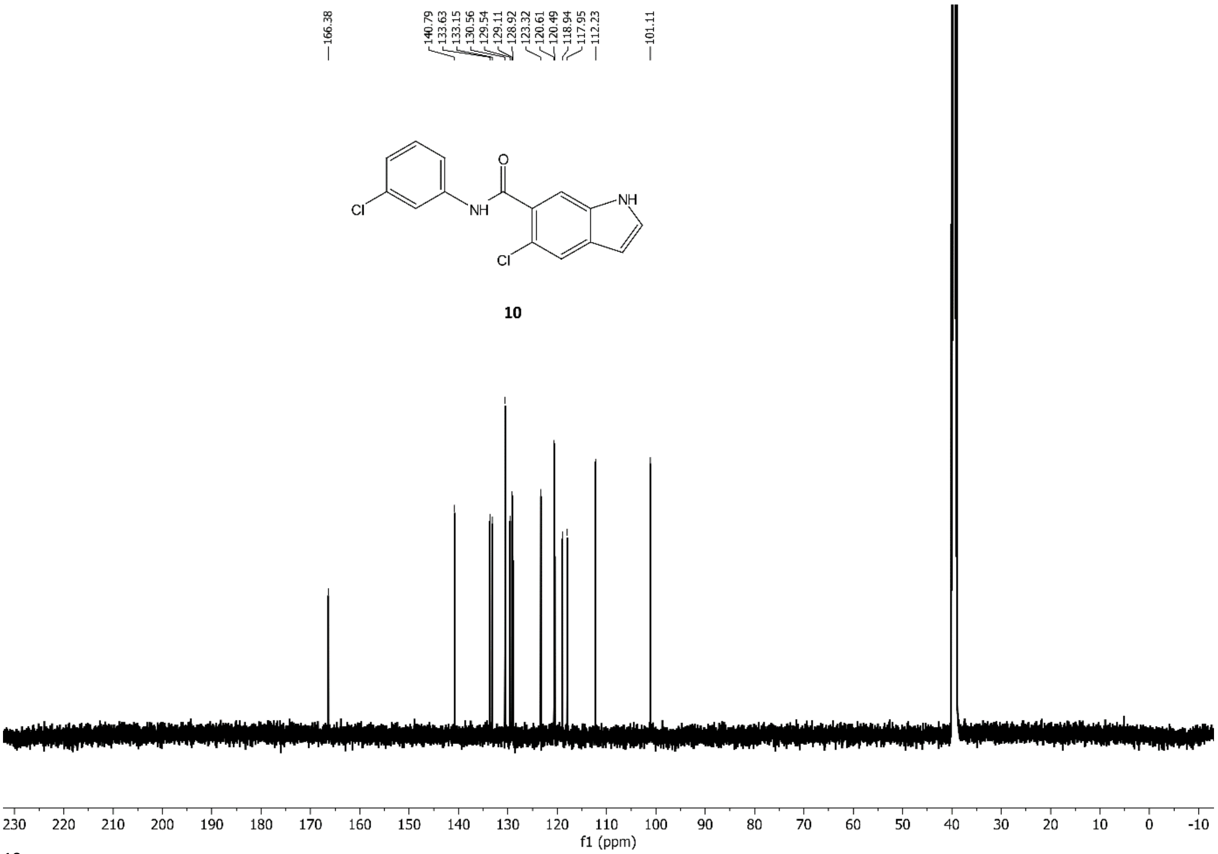

^{13}C-NMR (126 MHz, DMSO-d_6) of compound **10**.

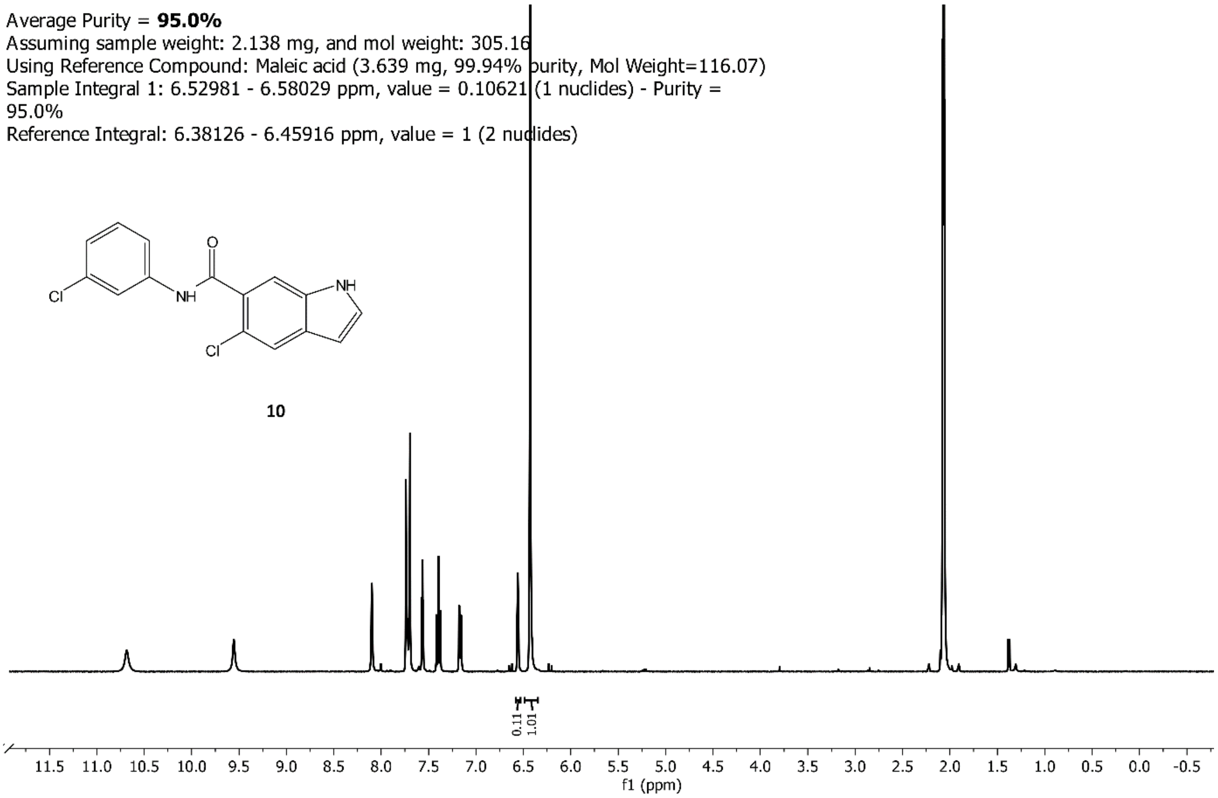

qHNMR (400 MHz, acetone-d$_6$, maleic acid as reference) of compound **10**.

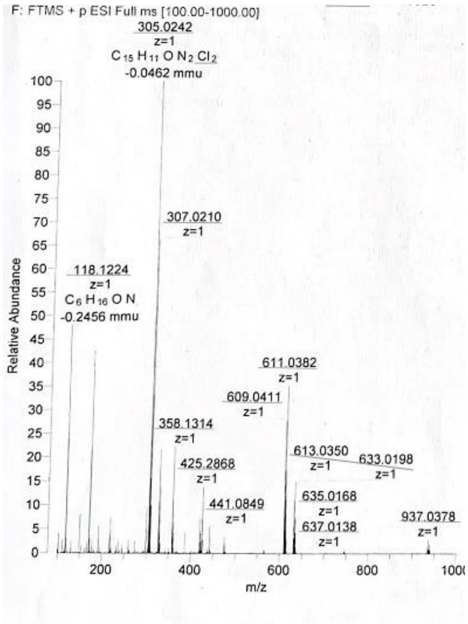

HRMS (ESI+) of compound **10**.

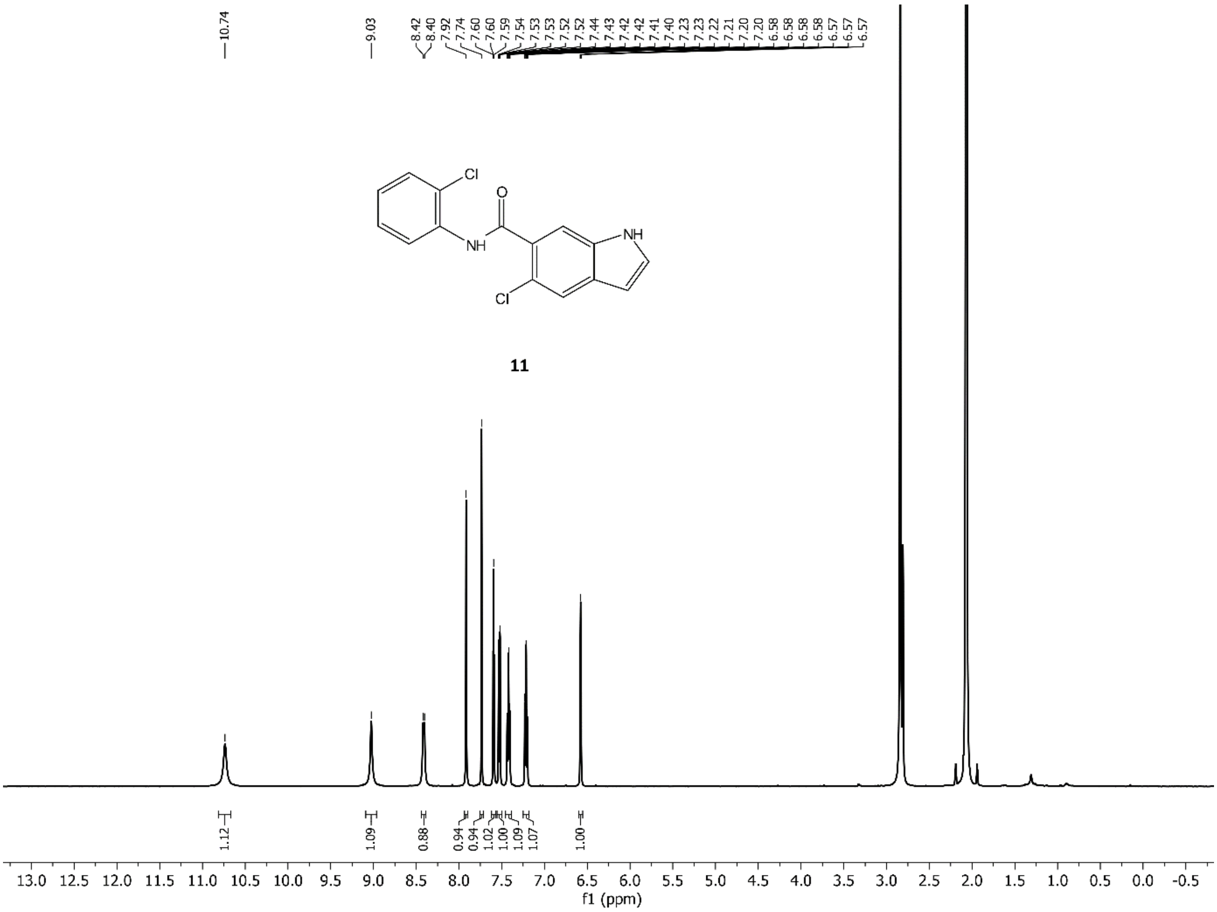

^{1}H-NMR (500 MHz, acetone-d_6) of compound **11**.

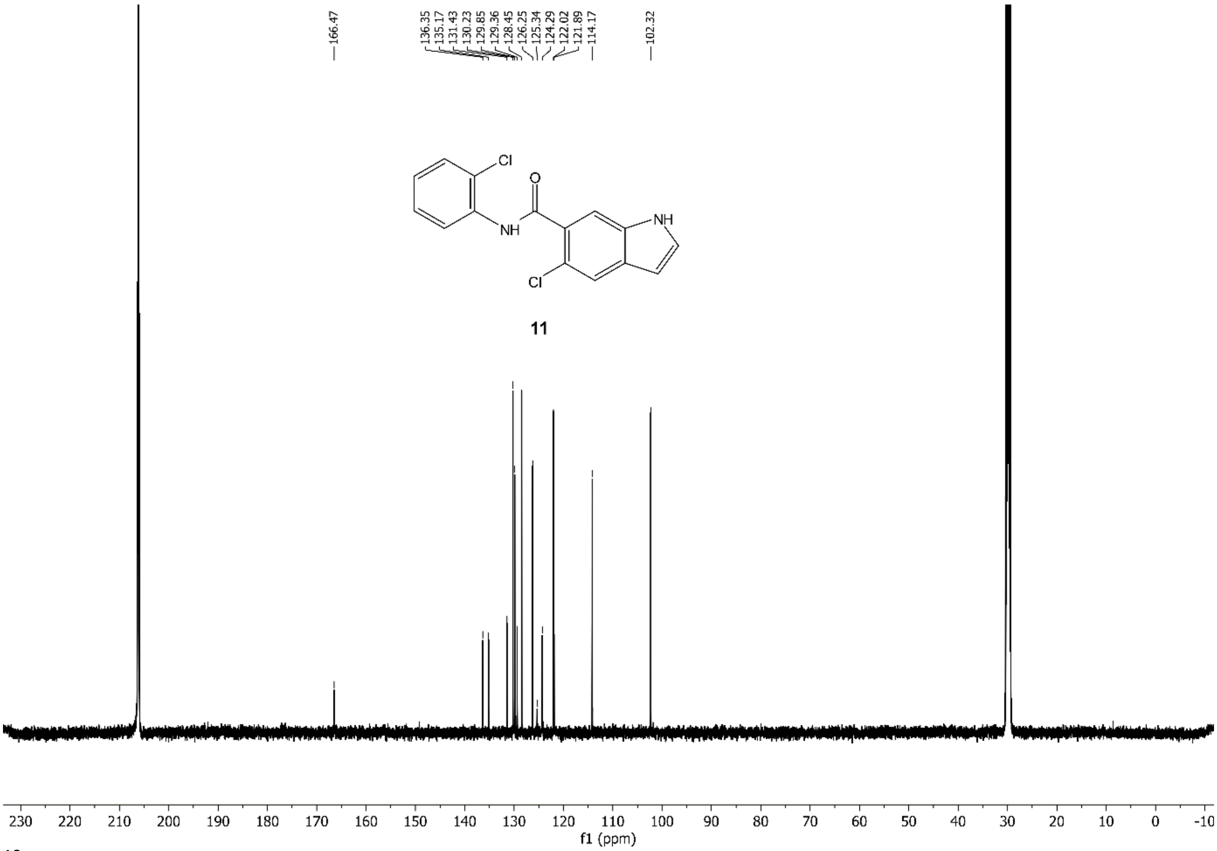

^{13}C-NMR (126 MHz, acetone-d_6) of compound **11**.

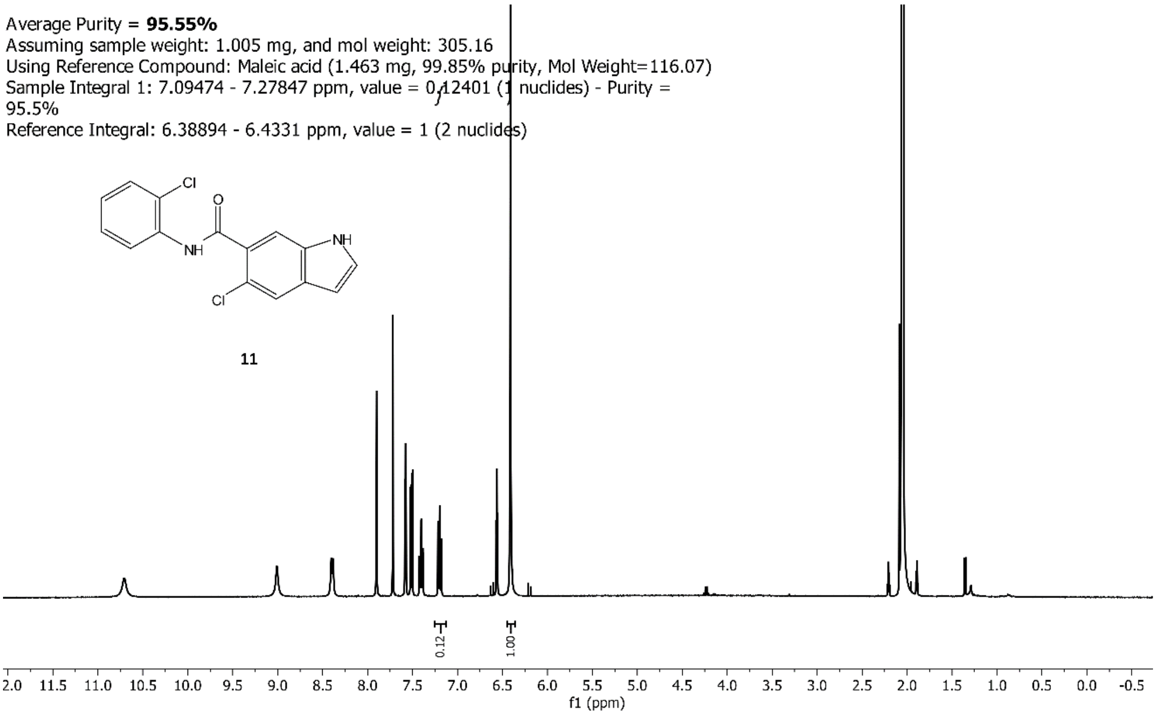

qHNMR (400 MHz, acetone-d_6, maleic acid as reference) of compound **11**.

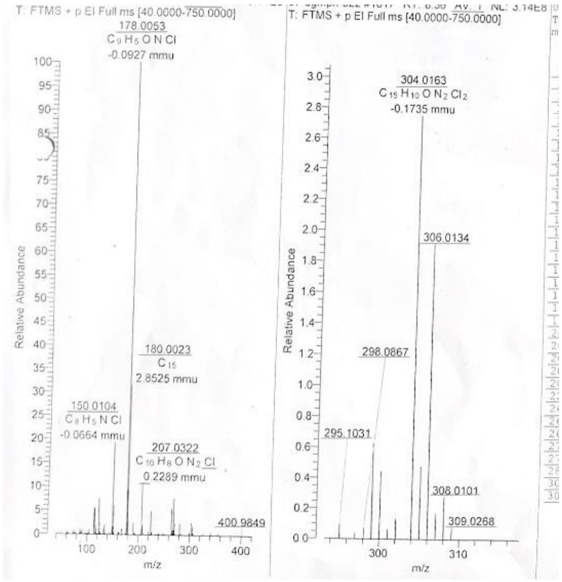

HRMS (EI+) of compound **11**. Left panel: Full spectrum. Right panel: Zoom in.

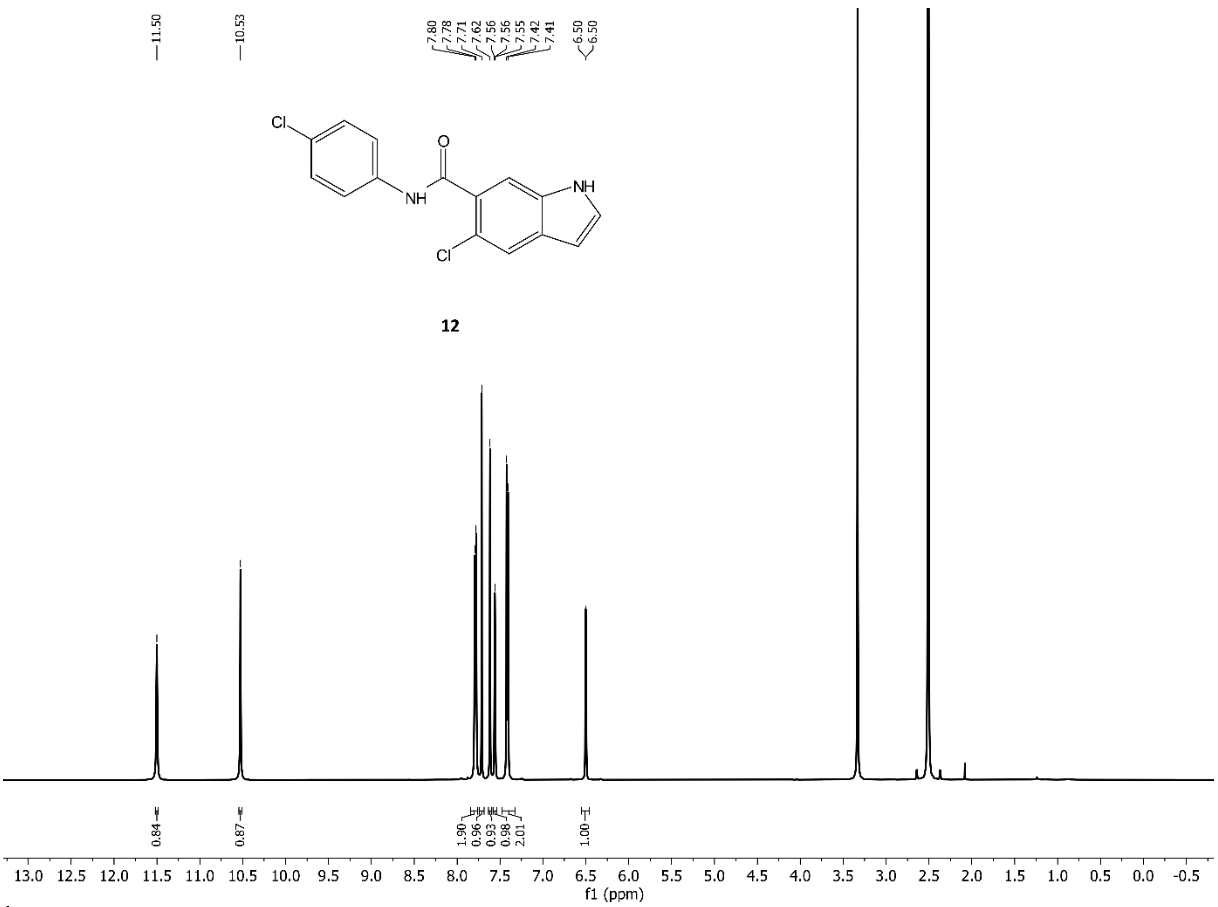

^{1}H-NMR (500 MHz, DMSO-d$_6$) of compound **12**.

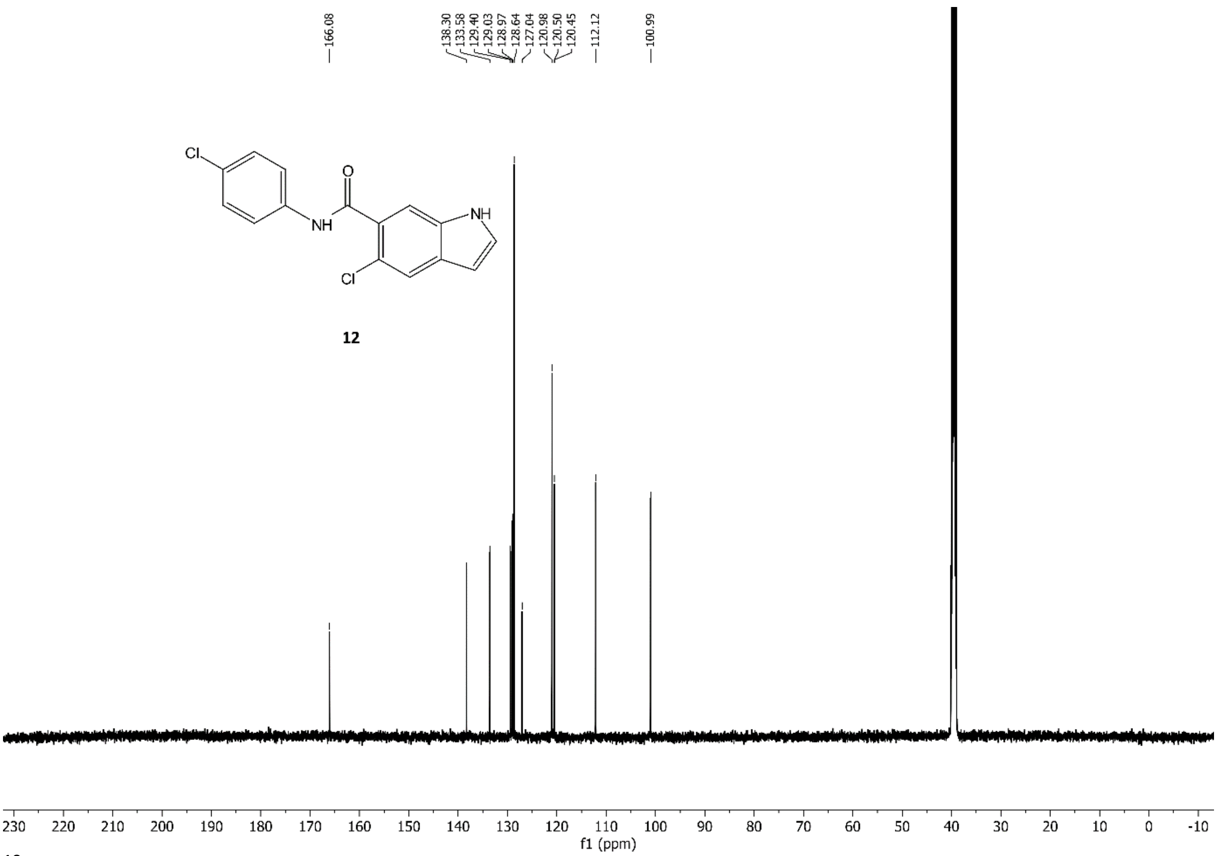

^{13}C-NMR (126 MHz, DMSO-d$_6$) of compound **12**.

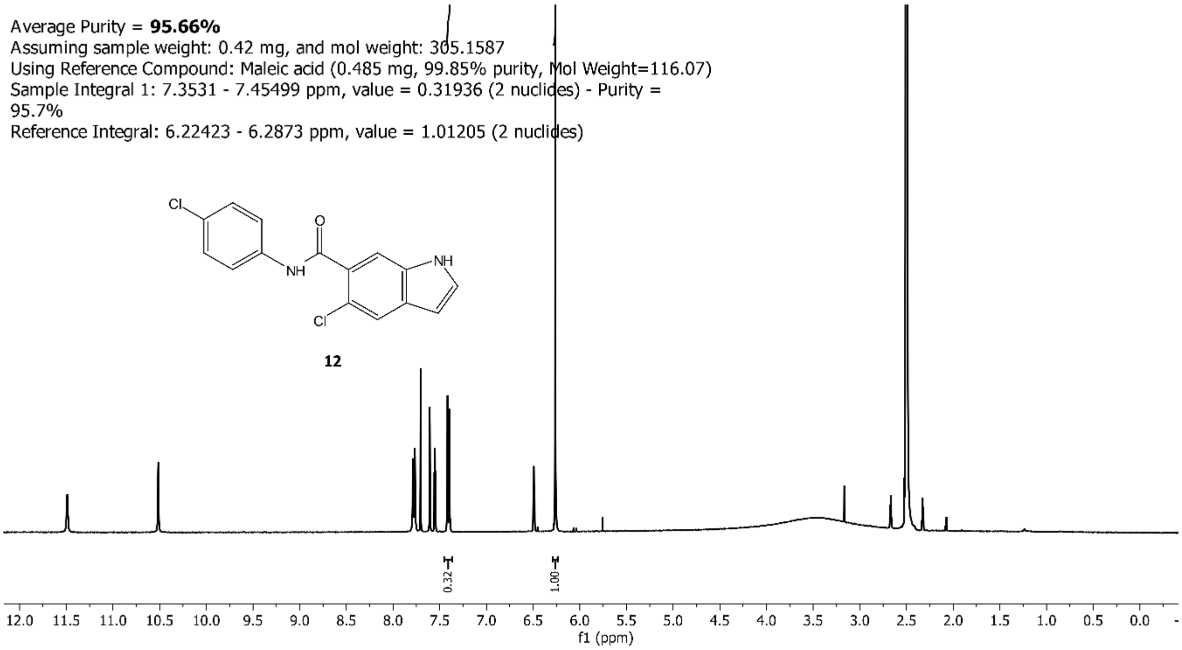

qHNMR (400 MHz, DMSO-d$_6$, maleic acid as reference) of compound **12**.

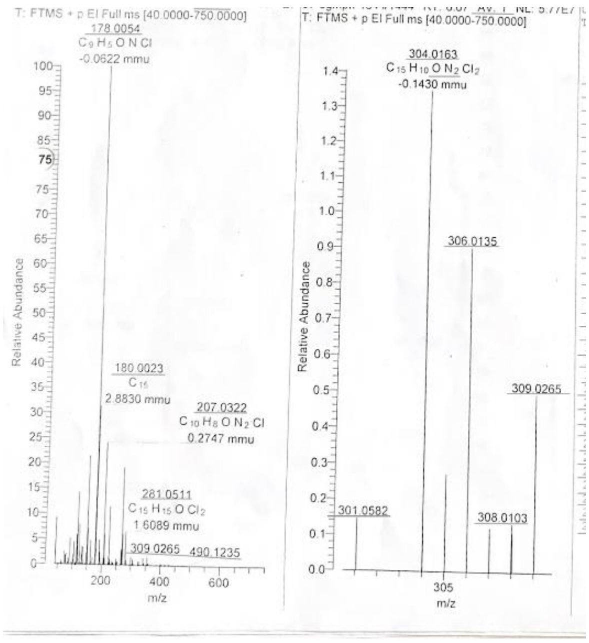

HRMS (EI+) of compound **12**. Left panel: Full spectrum. Right panel: Zoom in.

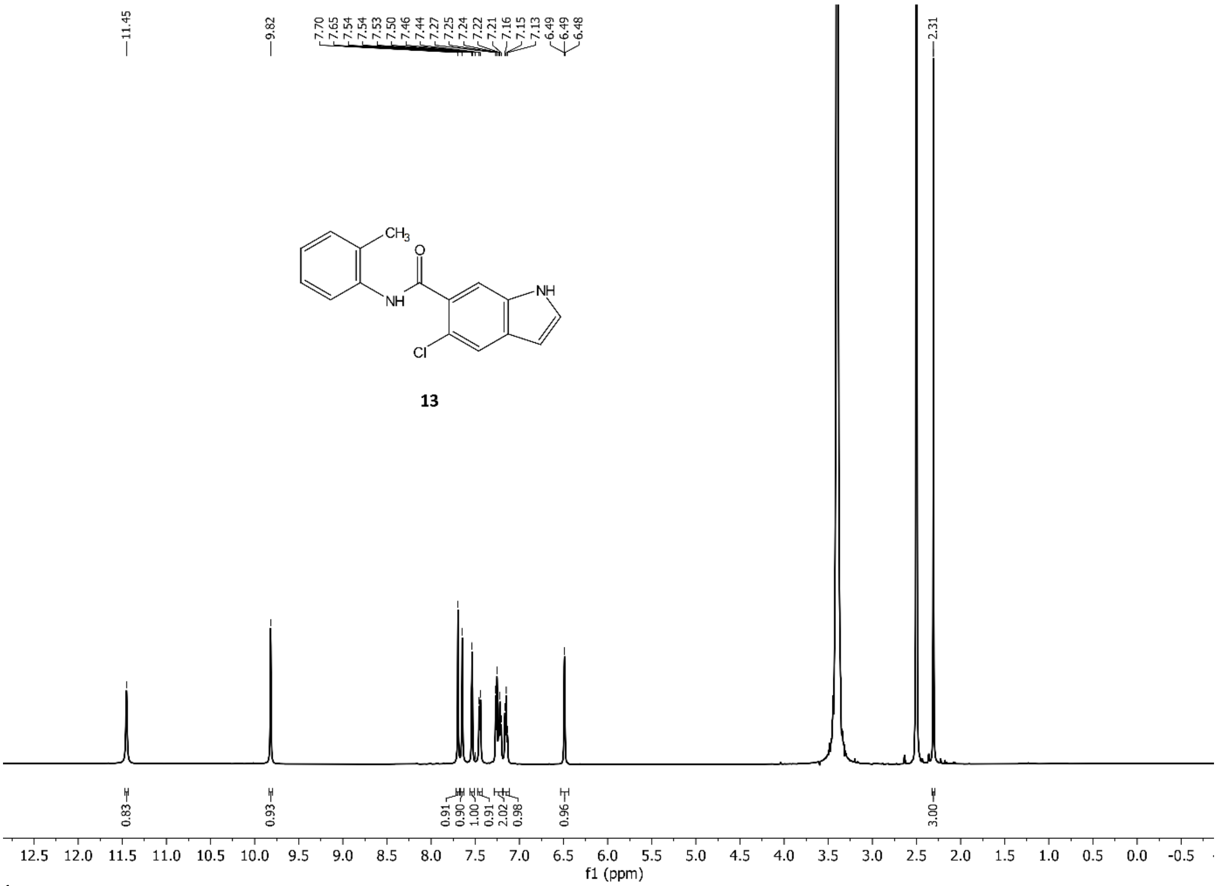

^{1}H-NMR (500 MHz, DMSO-d_6) of compound **13**.

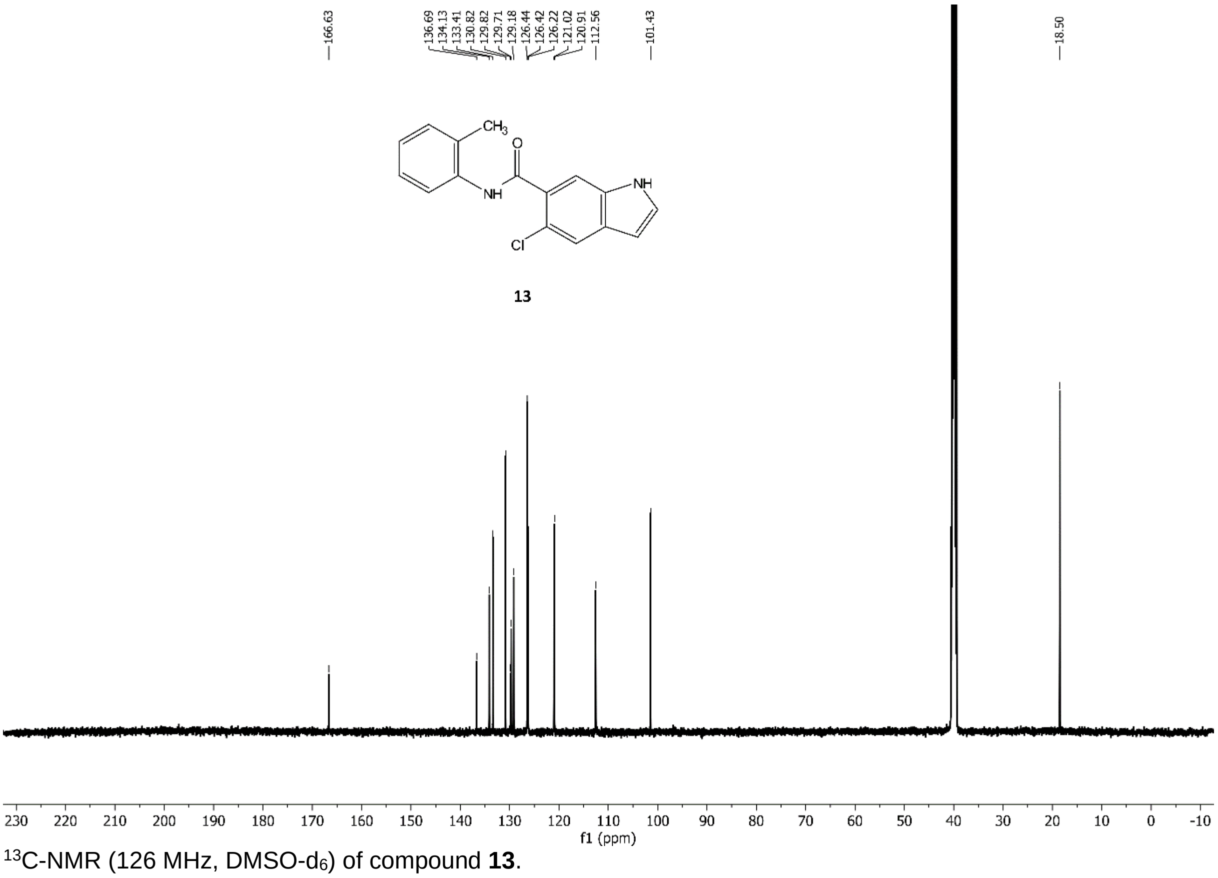

^{13}C-NMR (126 MHz, DMSO-d_6) of compound **13**.

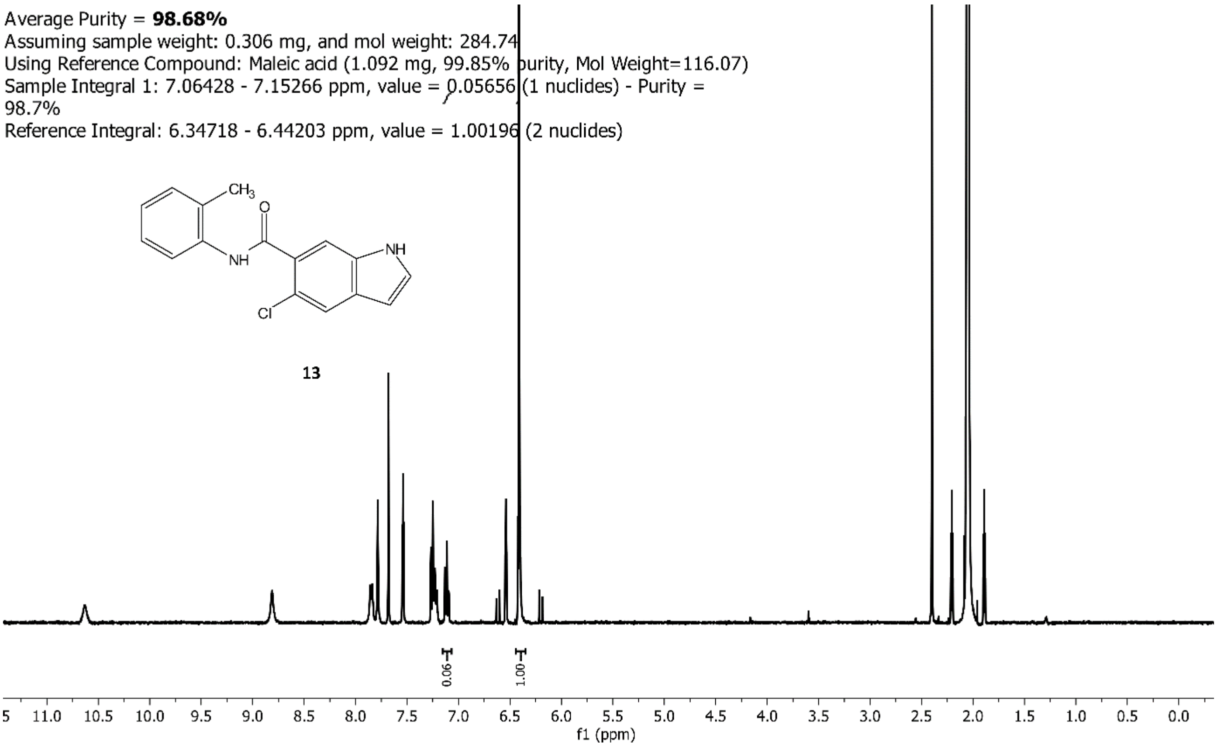

qHNMR (400 MHz, acetone-d_6, maleic acid as reference) of compound **13**.

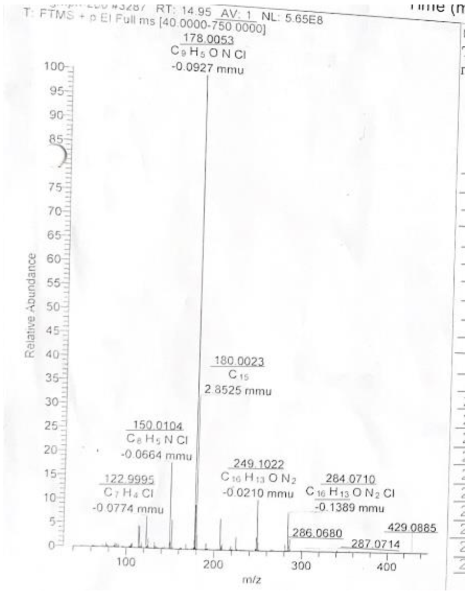

HRMS (EI+) of compound **13**.

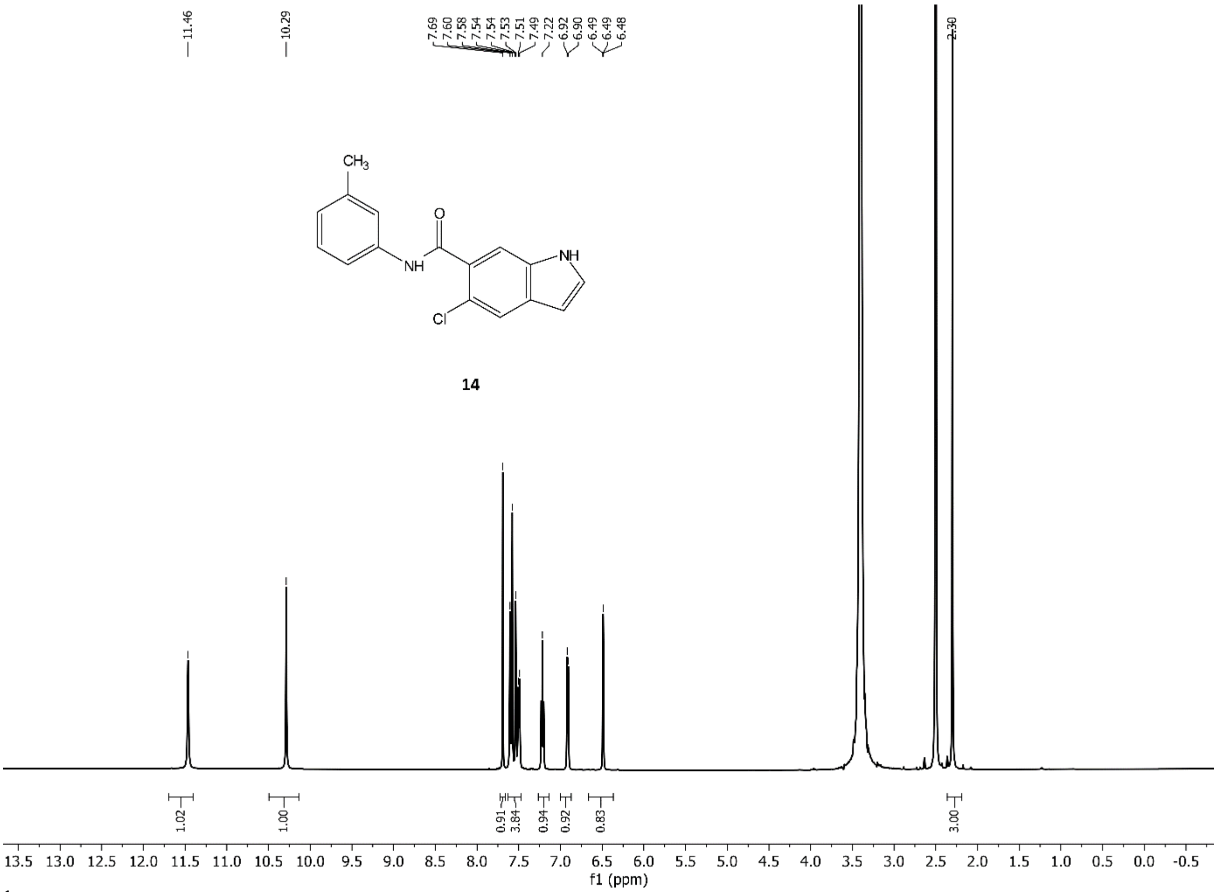

^{1}H-NMR (500 MHz, DMSO-d6) of compound **14**.

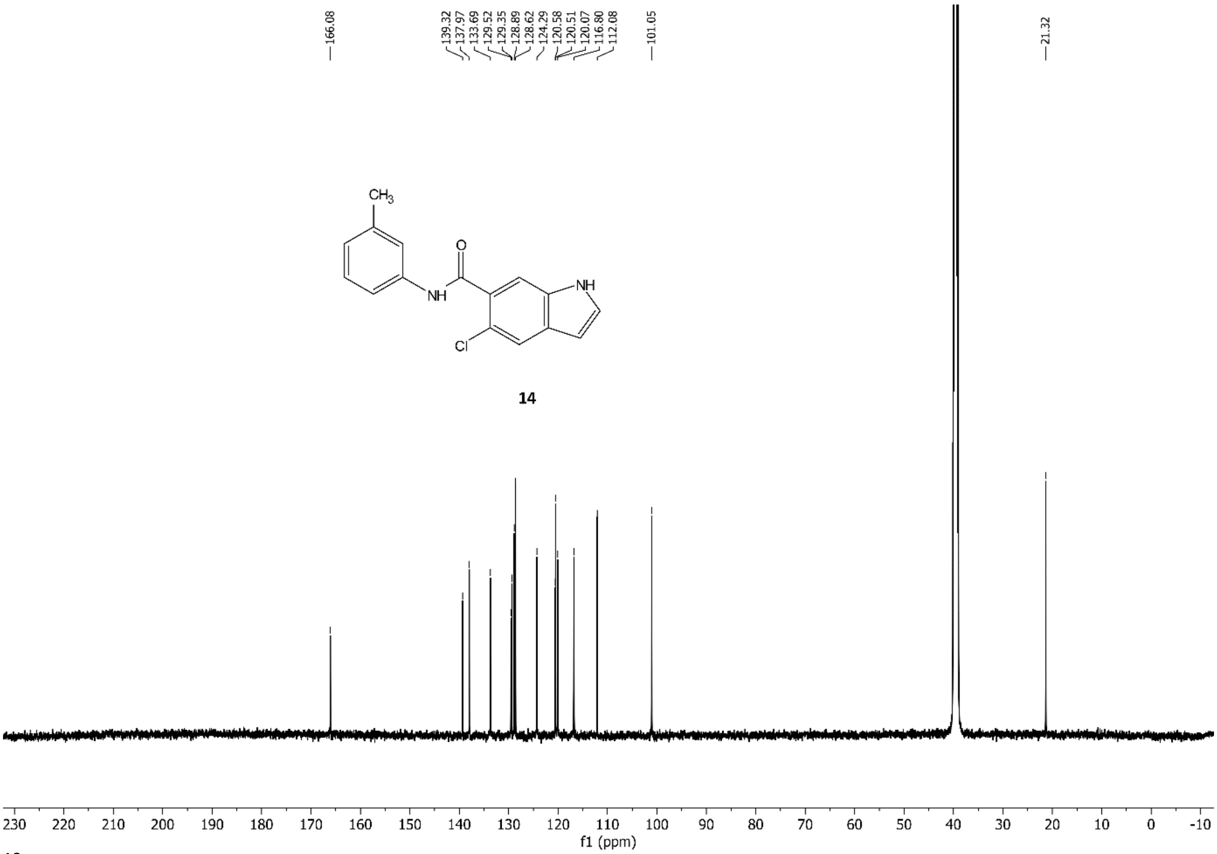

^{13}C-NMR (126 MHz, DMSO-d6) of compound **14**.

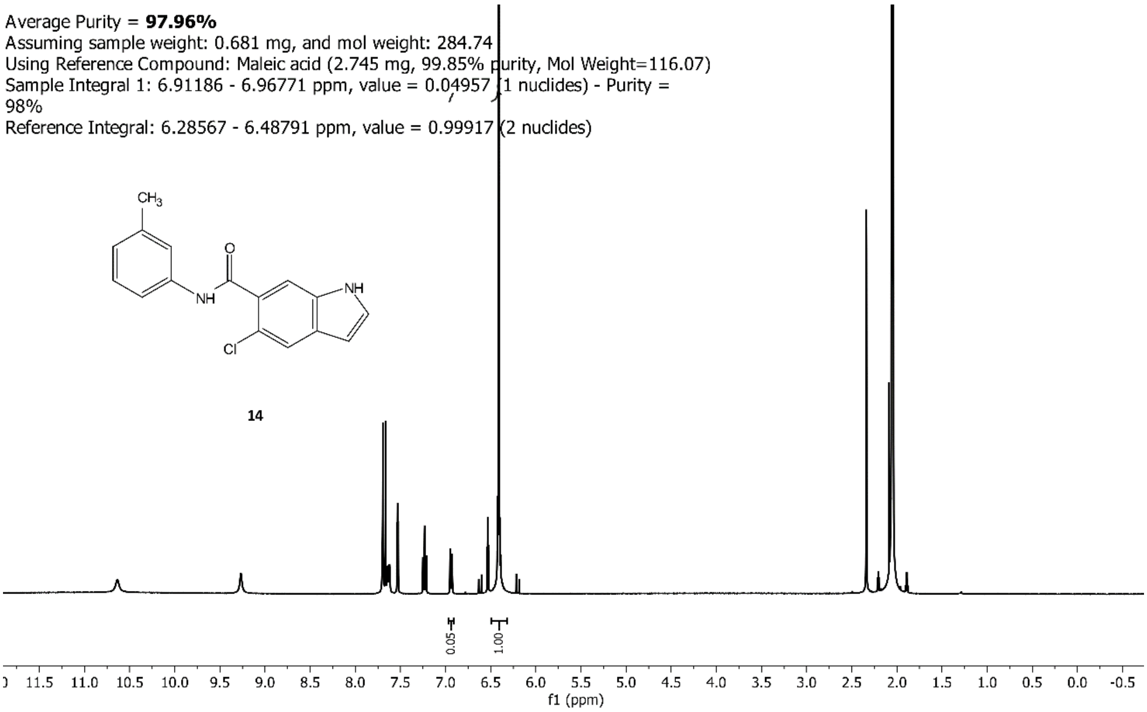

qHNMR (400 MHz, acetone-d_6, maleic acid as reference) of compound **14**.

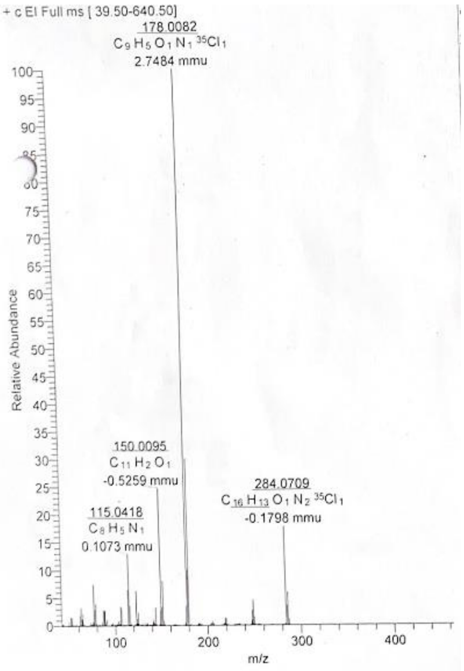

HRMS (EI+) of compound **14**.

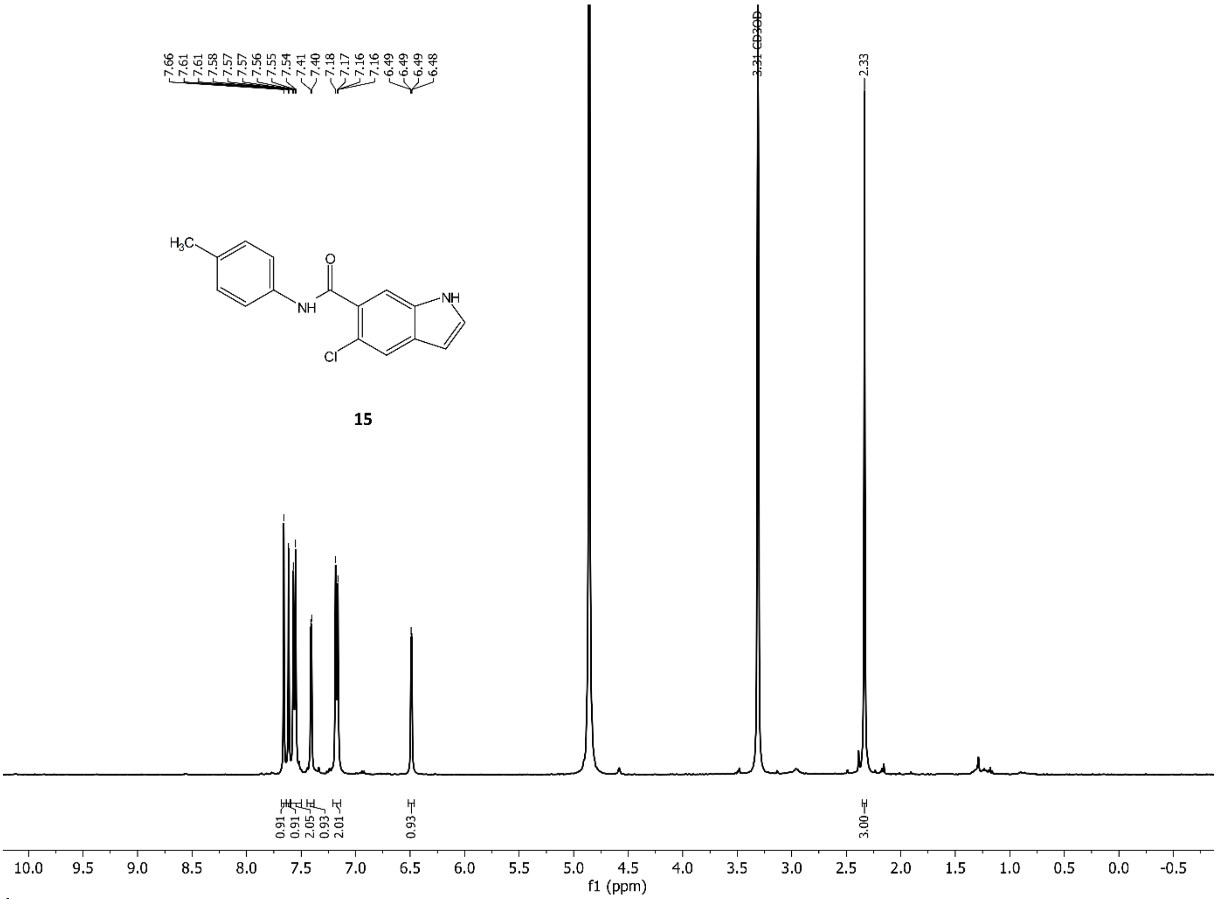

^{1}H-NMR (400 MHz, MeOD) of compound **15**.

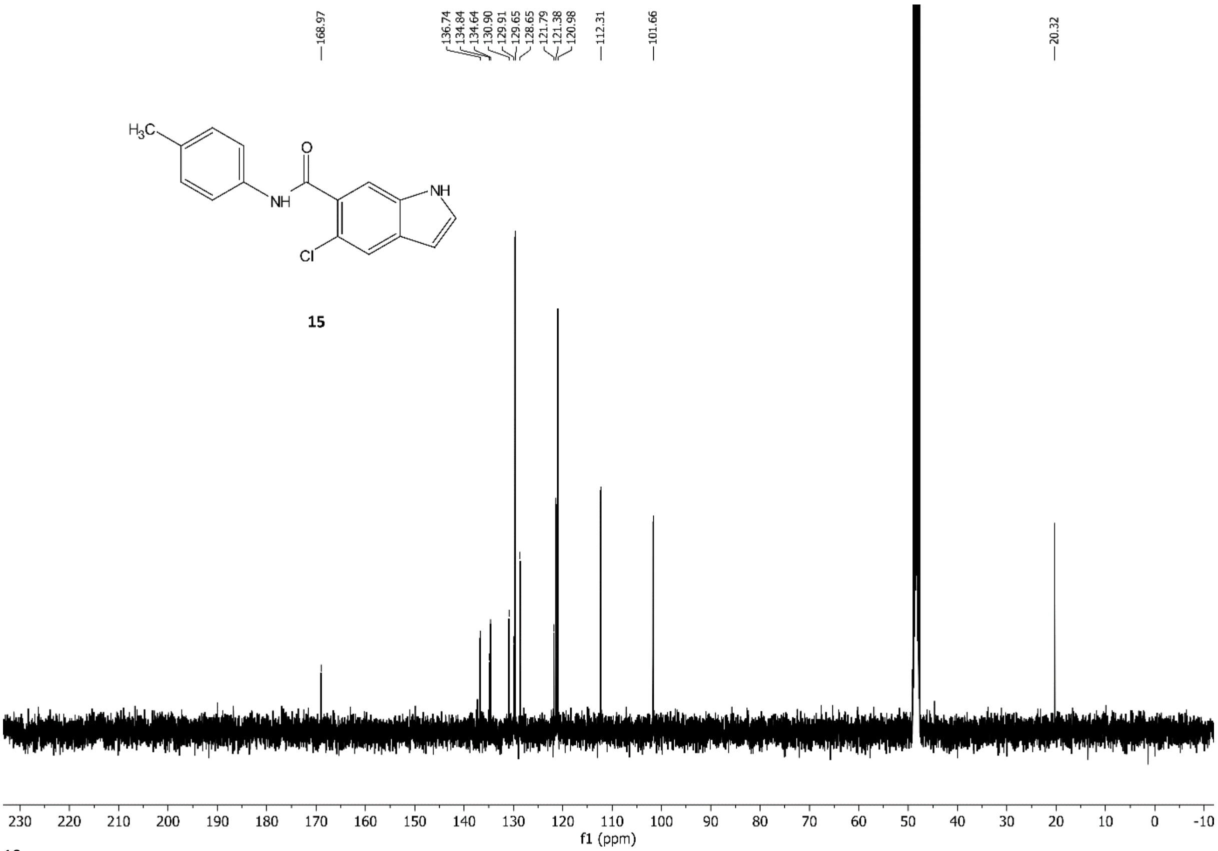

^{13}C-NMR (101 MHz, MeOD) of compound **15.**

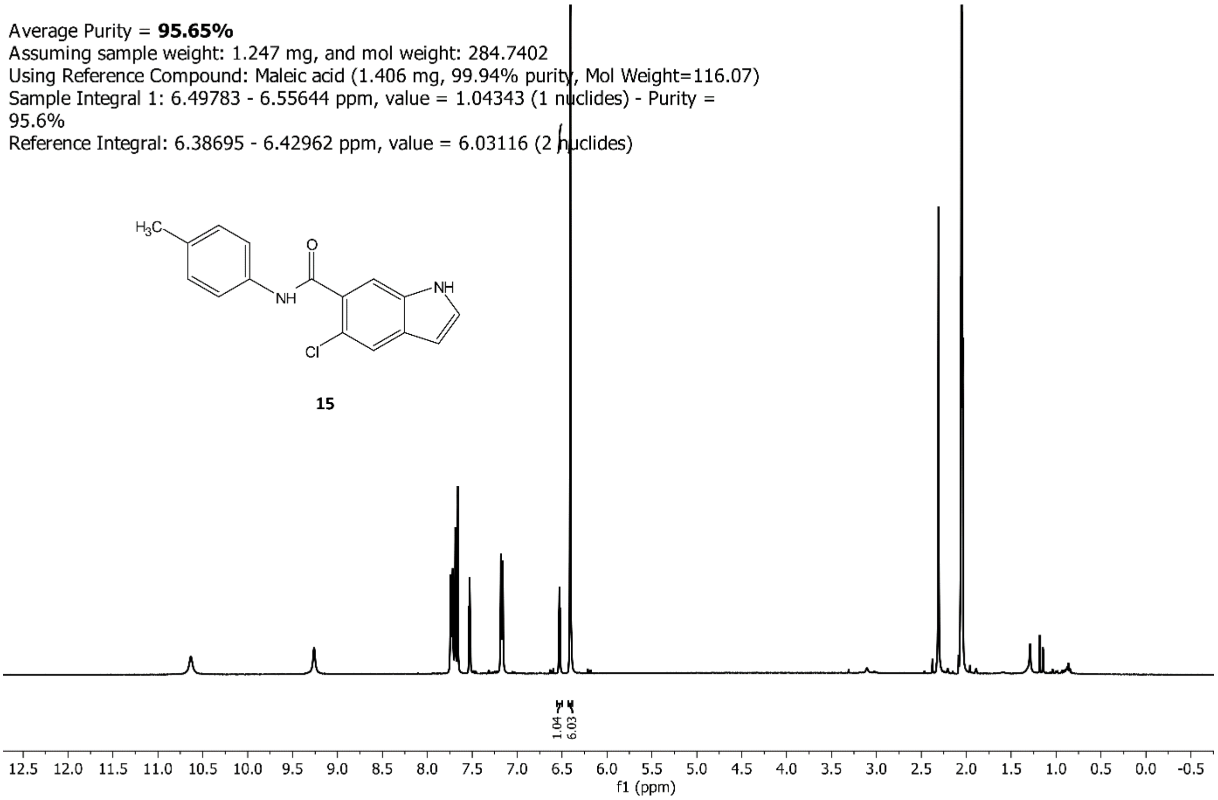

qHNMR (400 MHz, acetone-d$_6$, maleic acid as reference) of compound **15**.

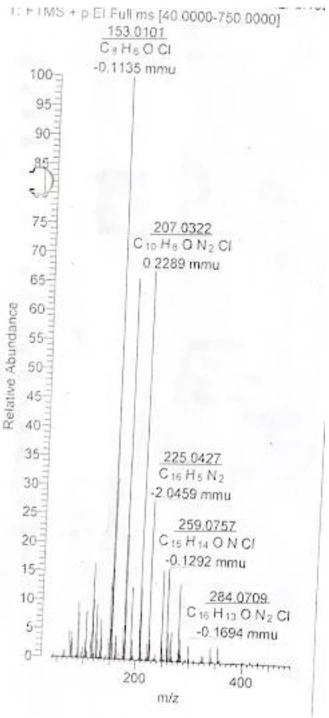

HRMS (EI+) of compound **15**.

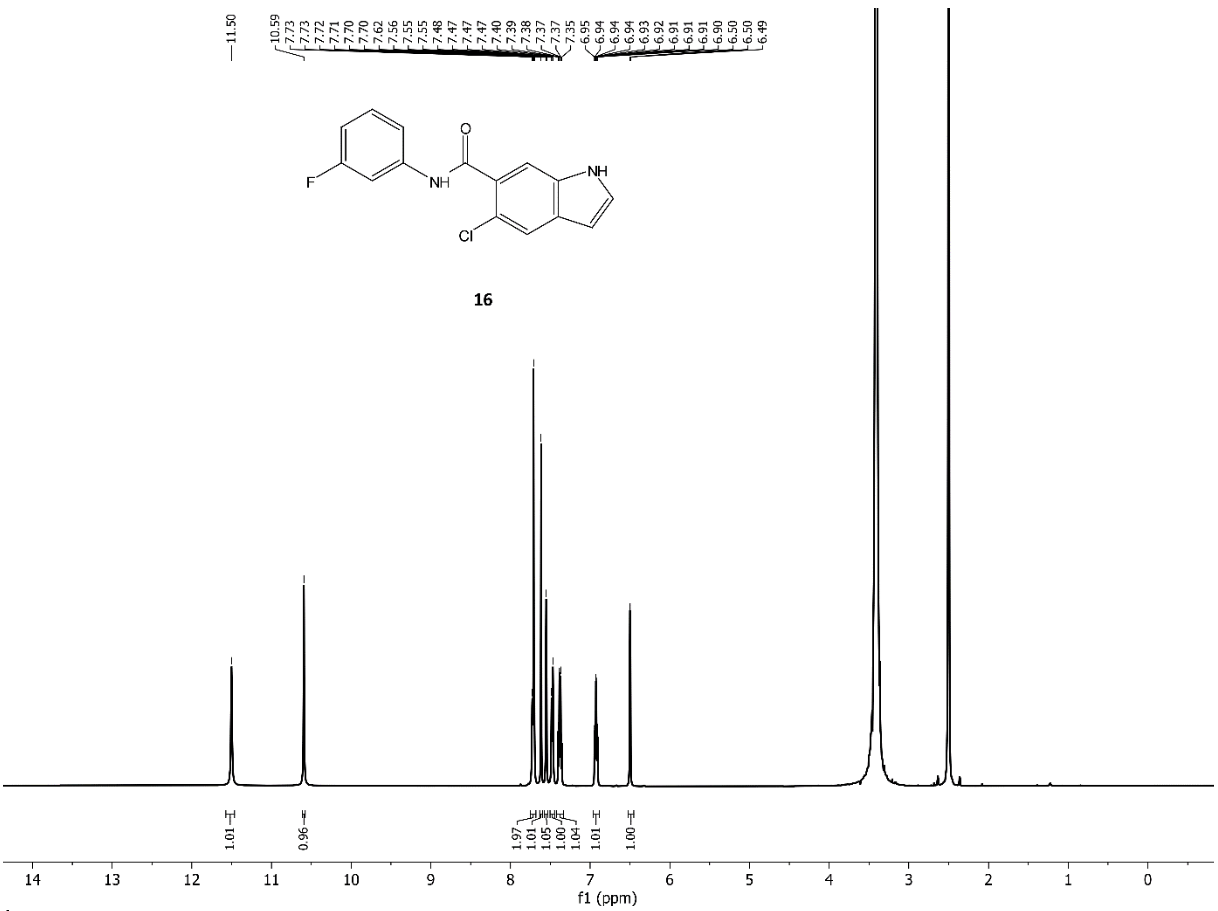

^{1}H-NMR (500 MHz, DMSO-d$_6$) of compound **16**.

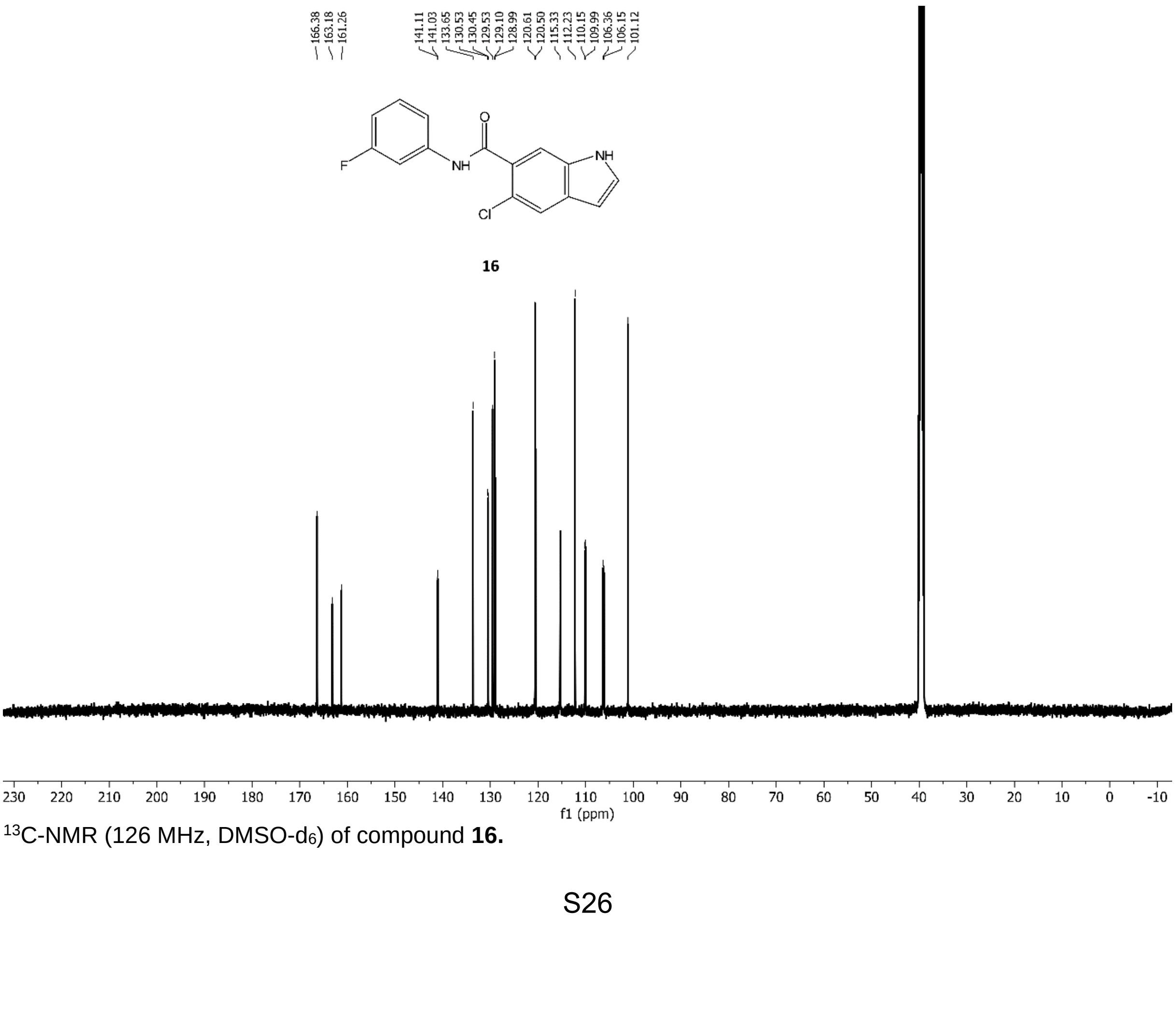

^{13}C-NMR (126 MHz, DMSO-d$_6$) of compound **16.**

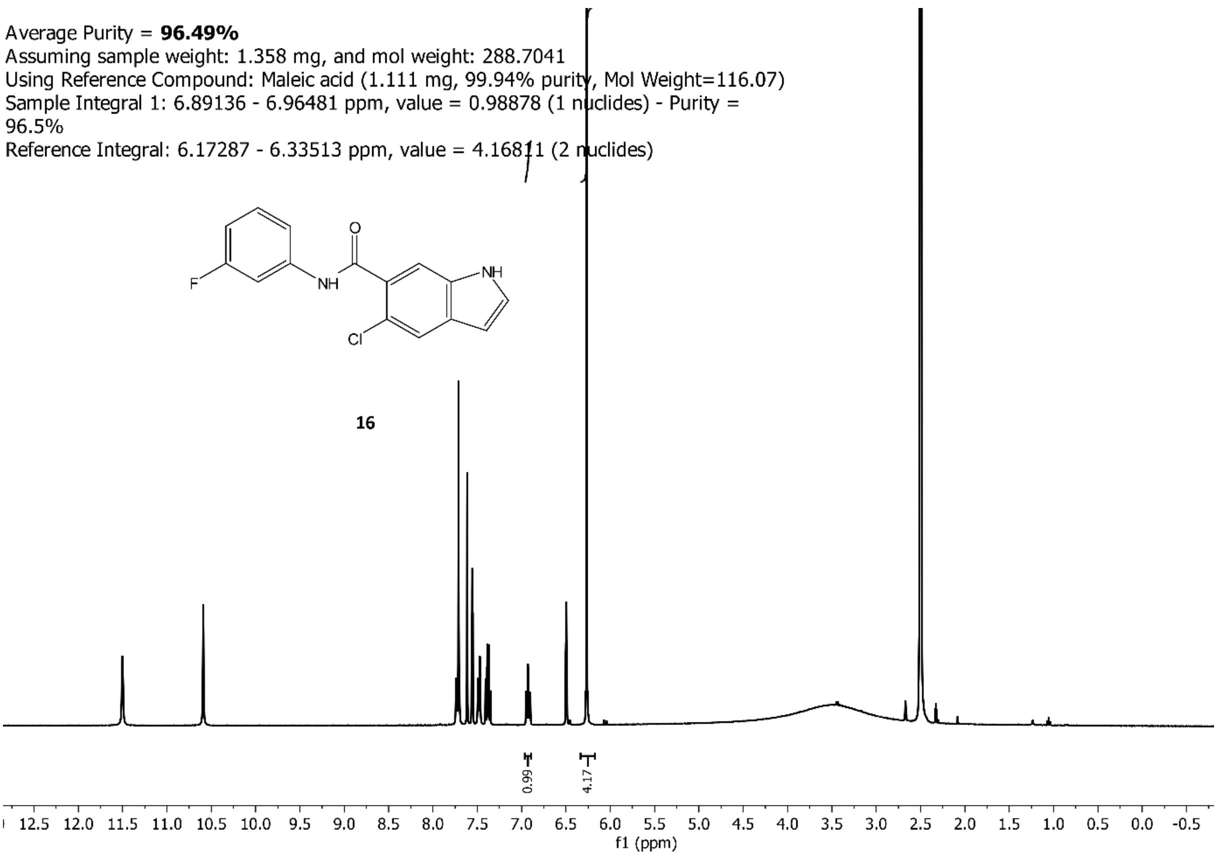

qHNMR (400 MHz, DMSO-d$_6$, maleic acid as reference) of compound **16**.

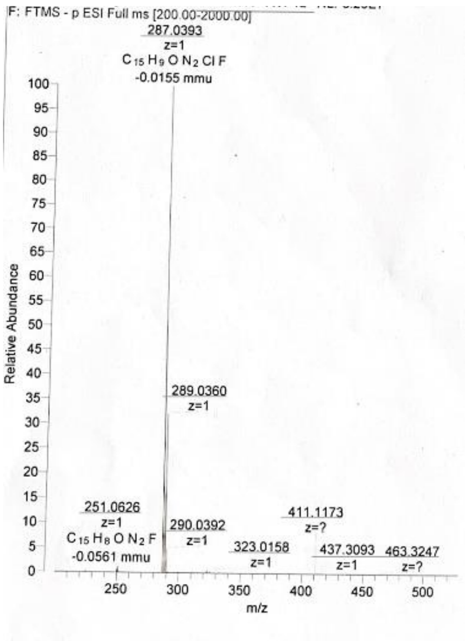

HRMS (ESI-) of compound **16**.

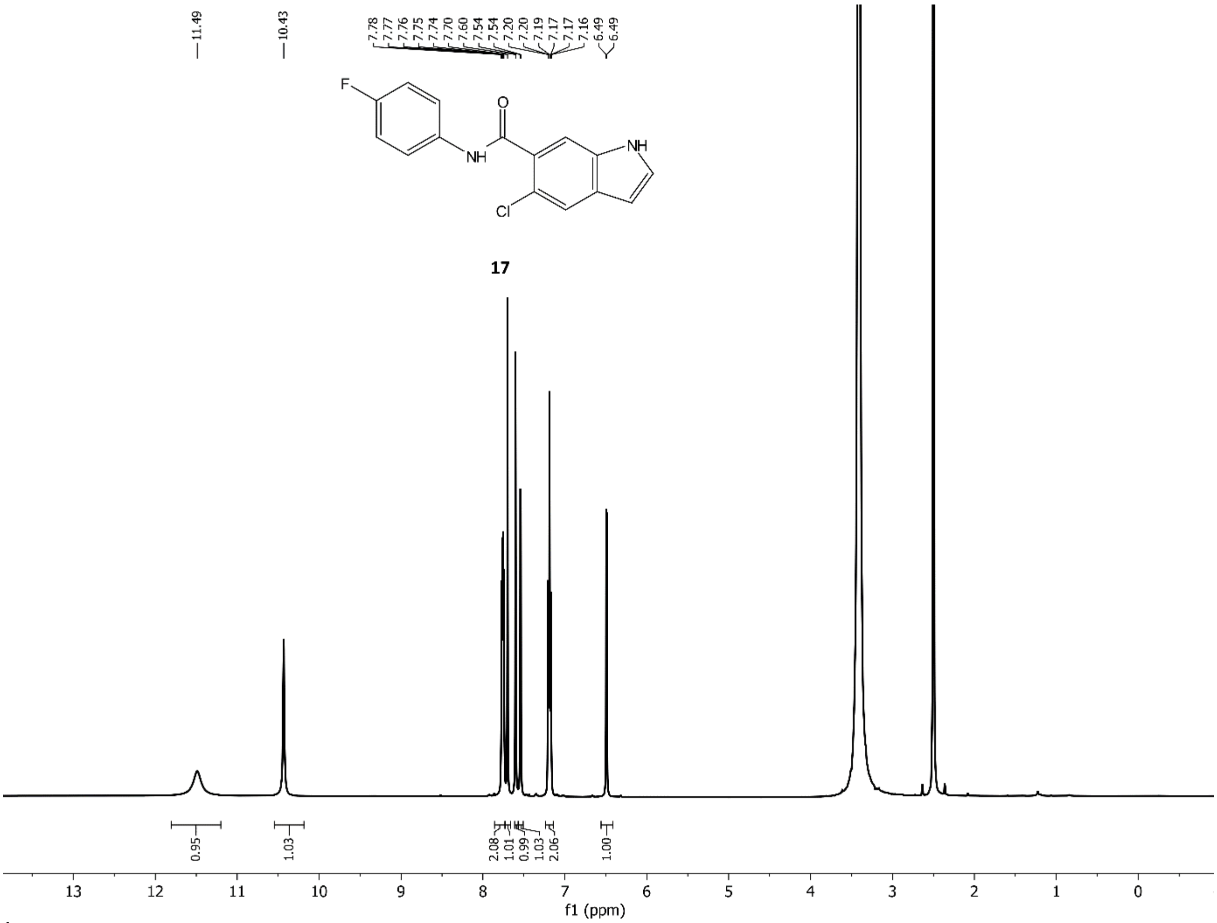

^{1}H-NMR (500 MHz, DMSO-d$_6$) of compound **17**.

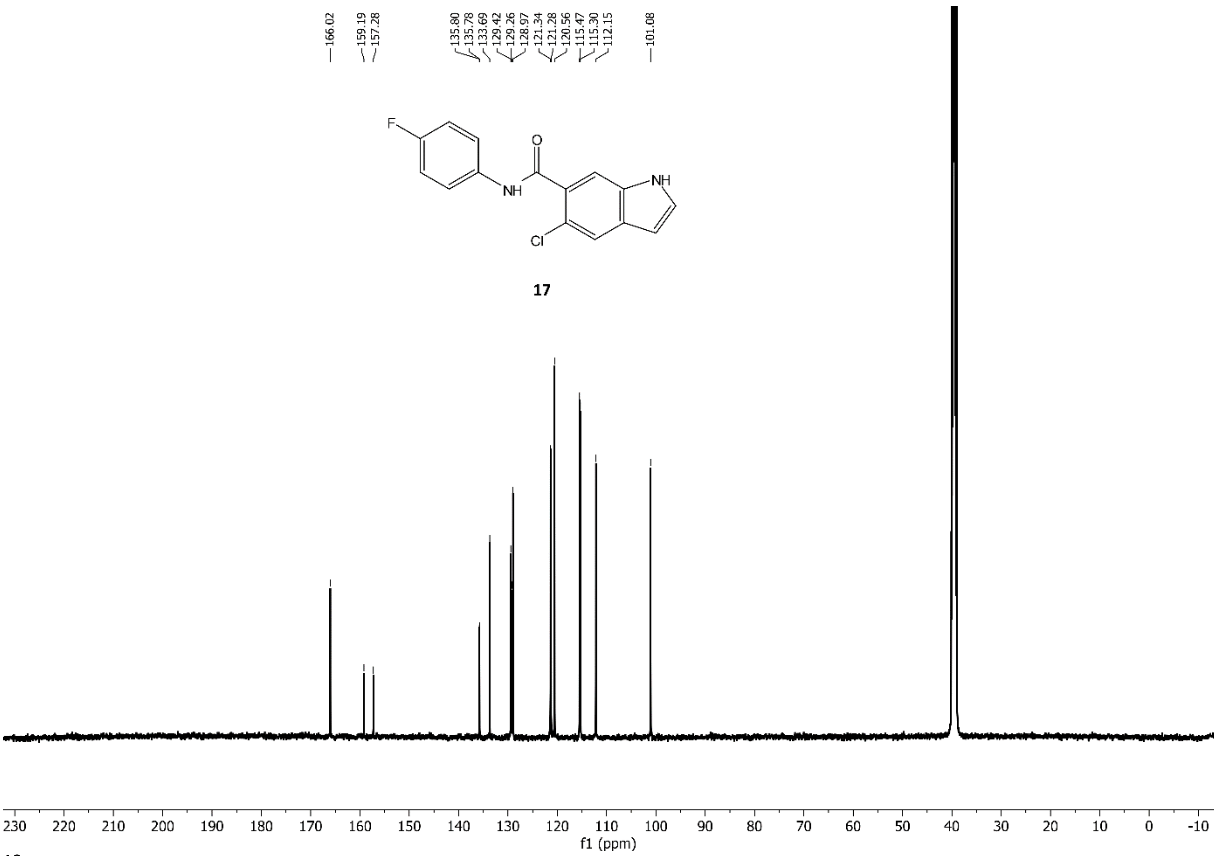

^{13}C-NMR (126 MHz, DMSO-d$_6$) of compound **17.**

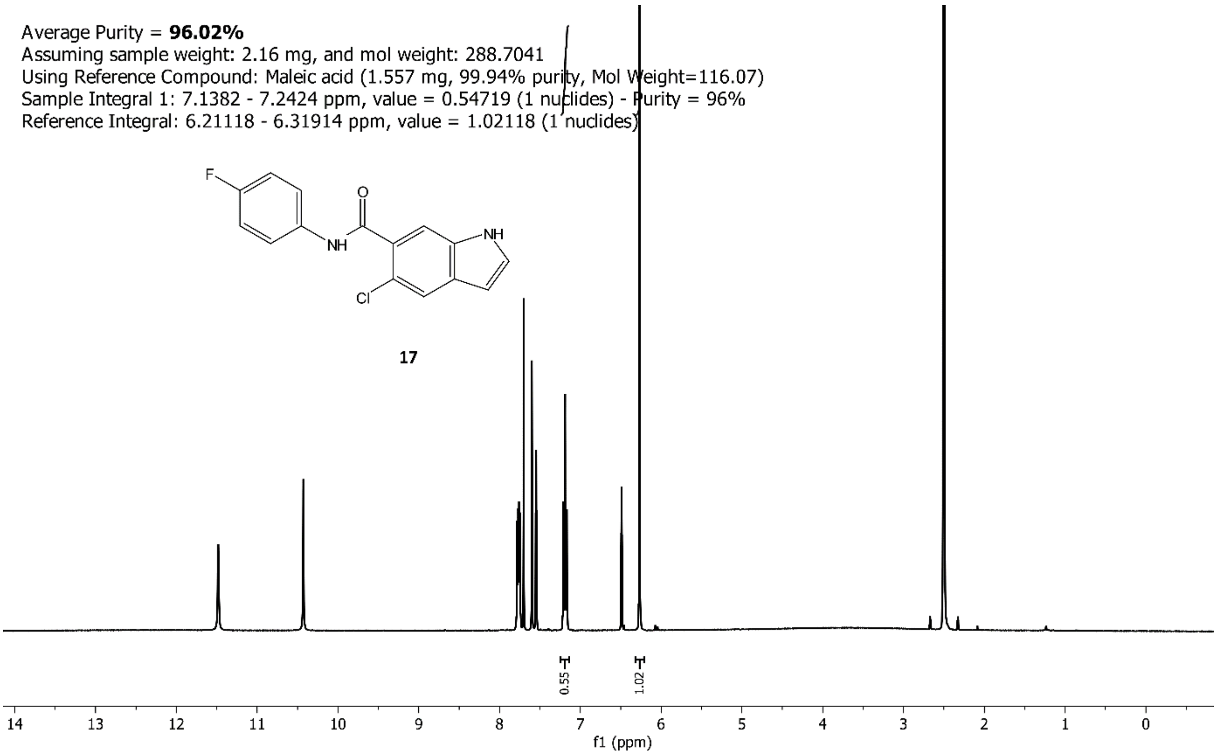

qHNMR (400 MHz, DMSO-d$_6$, maleic acid as reference) of compound **17**.

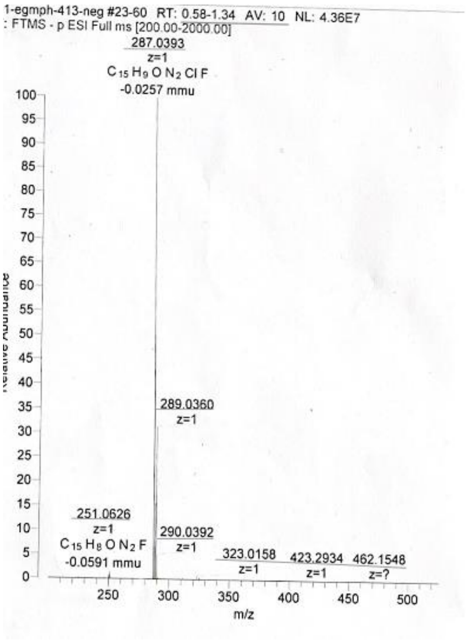

HRMS (ESI-) of compound **17**.

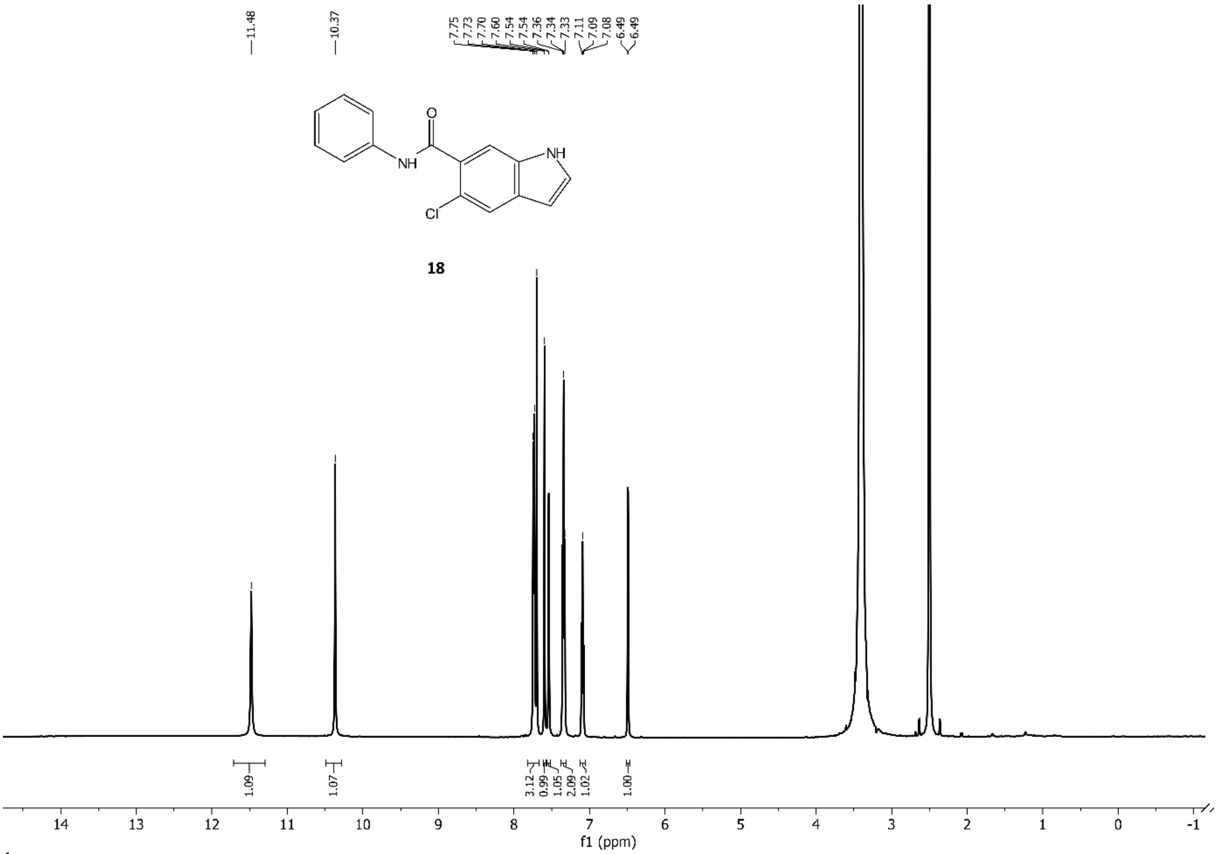

^{1}H-NMR (500 MHz, DMSO-d_6) of compound **18**.

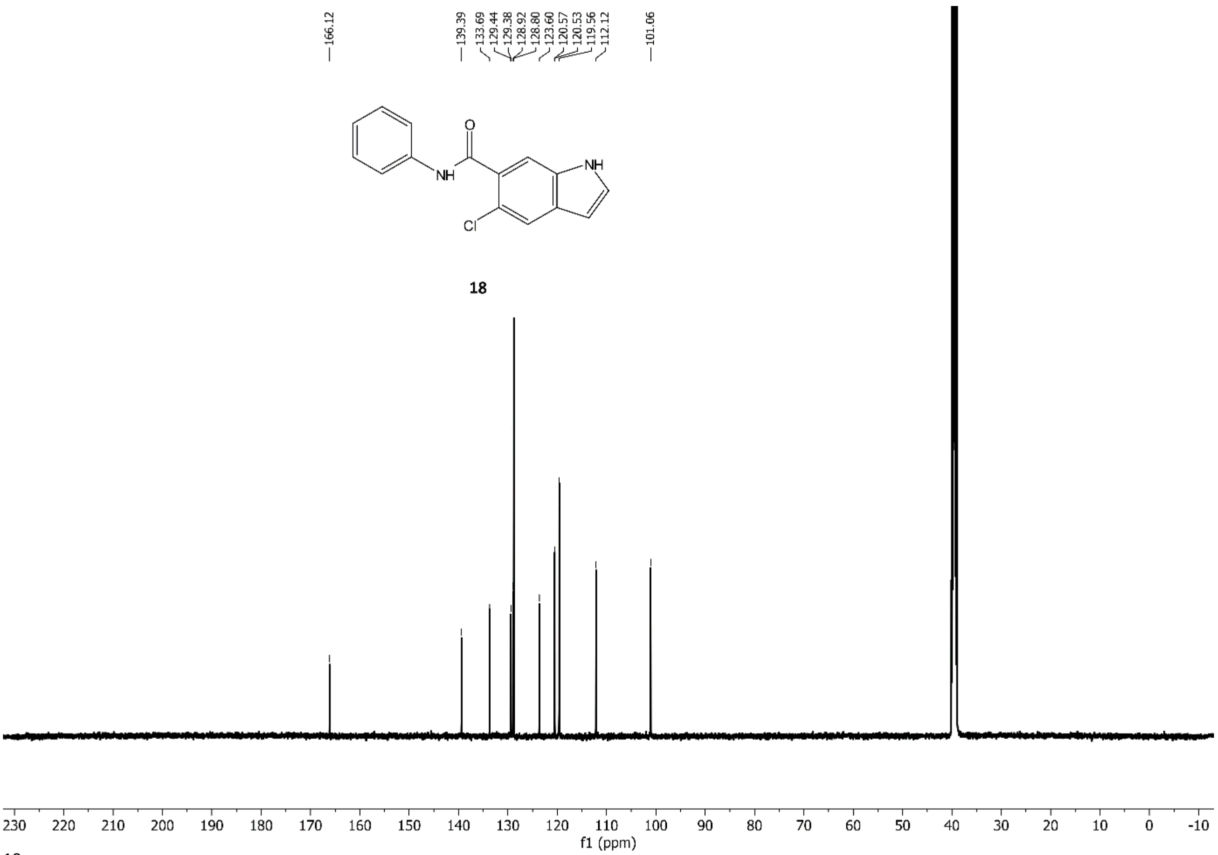

^{13}C-NMR (126 MHz, DMSO-d_6) of compound **18**.

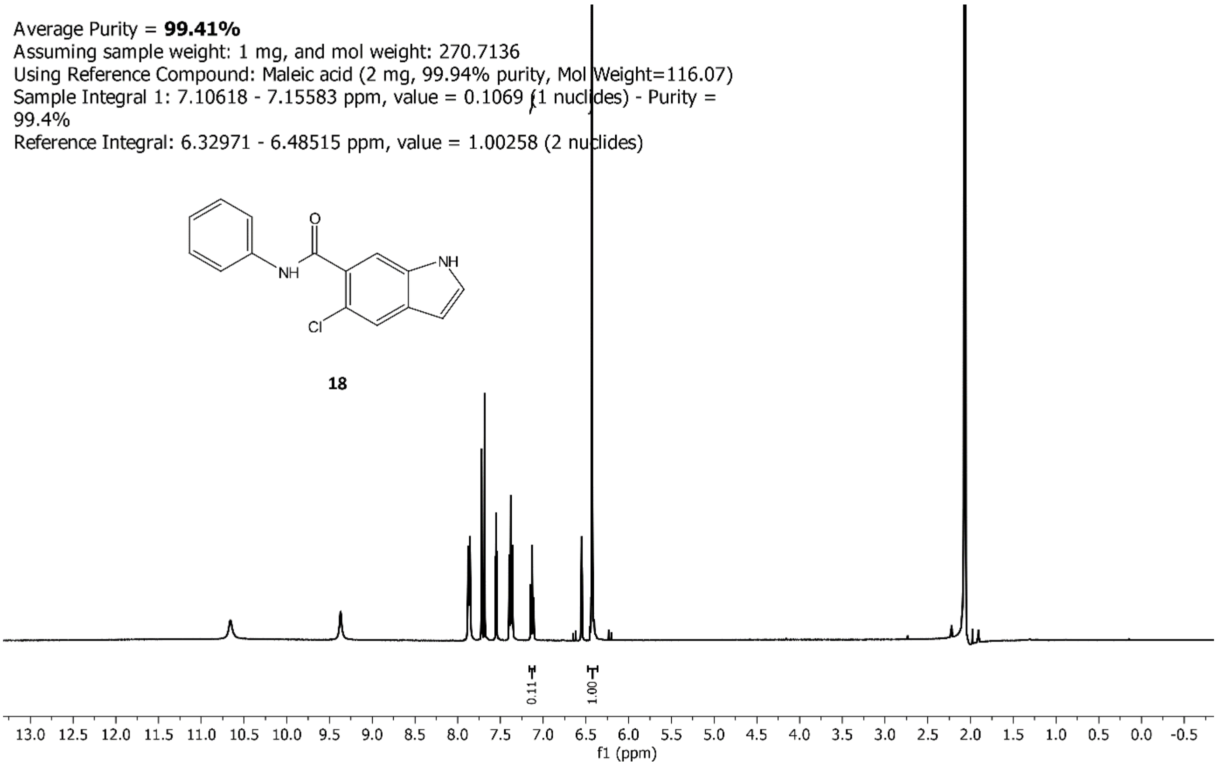

qHNMR (400 MHz, acetone-d$_6$, maleic acid as reference) of compound **18**.

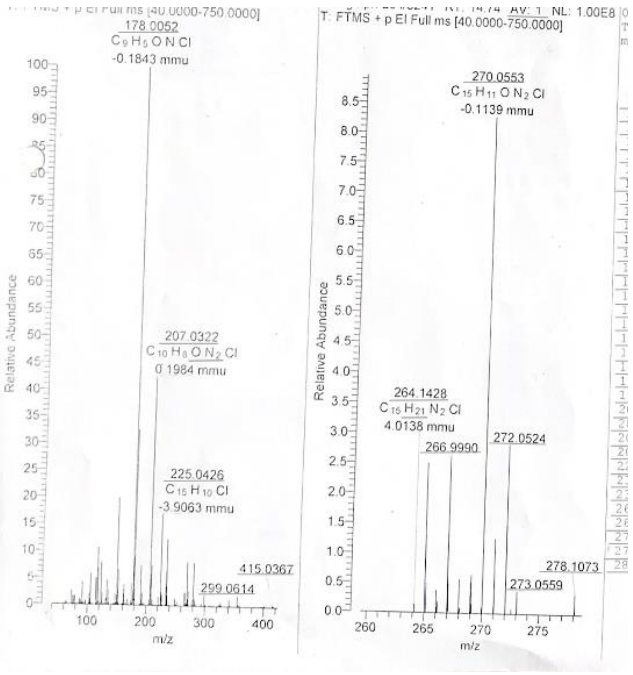

HRMS (EI+) of compound **18**. Left panel: Full spectrum. Right panel: Zoom in.

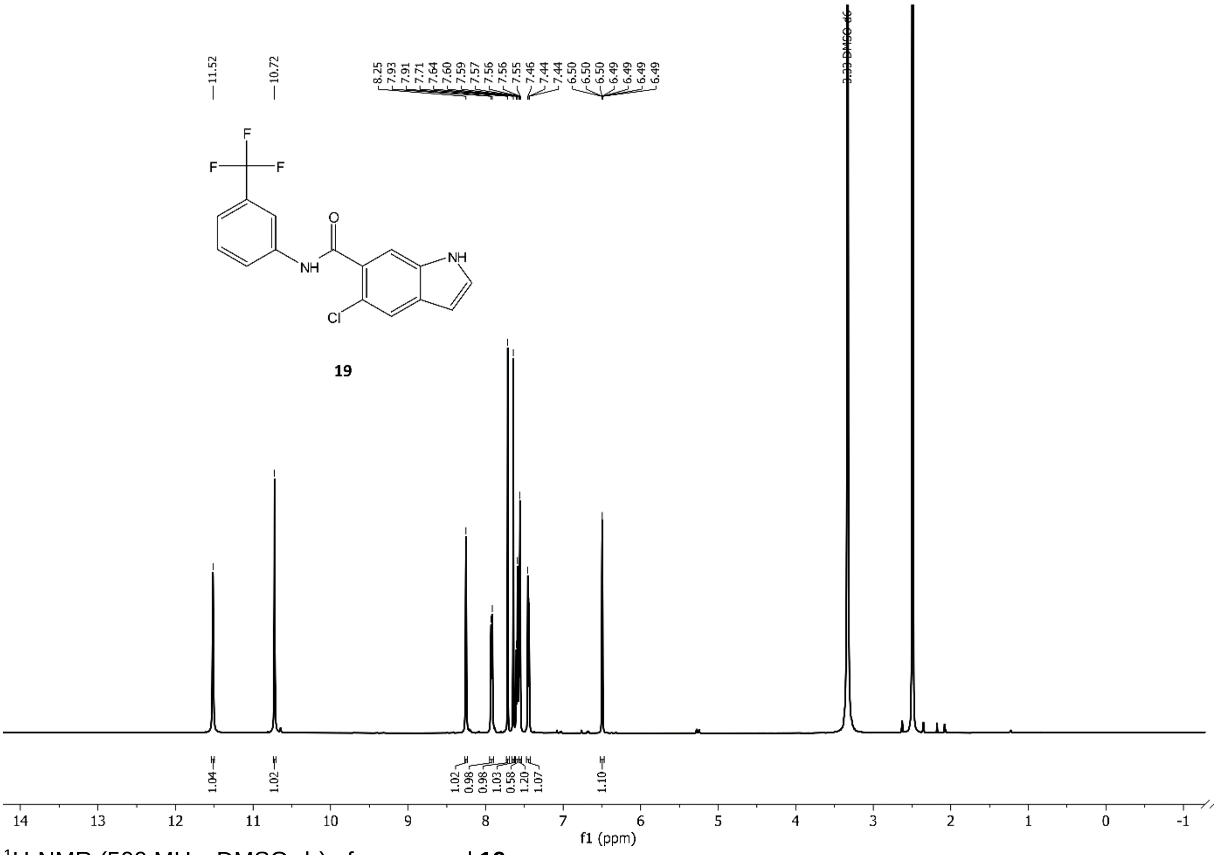

^{1}H-NMR (500 MHz, DMSO-d_6) of compound **19**.

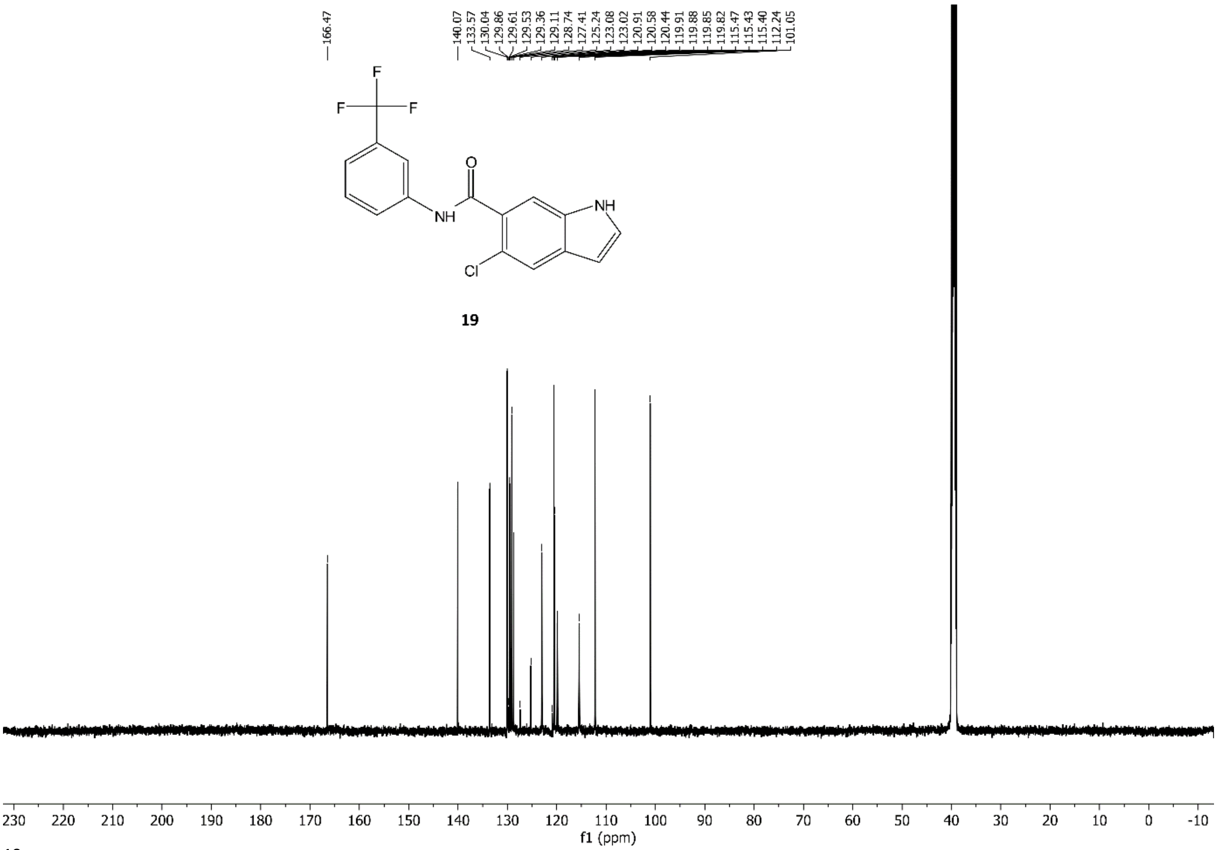

^{13}C-NMR (126 MHz, DMSO-d_6) of compound **19**.

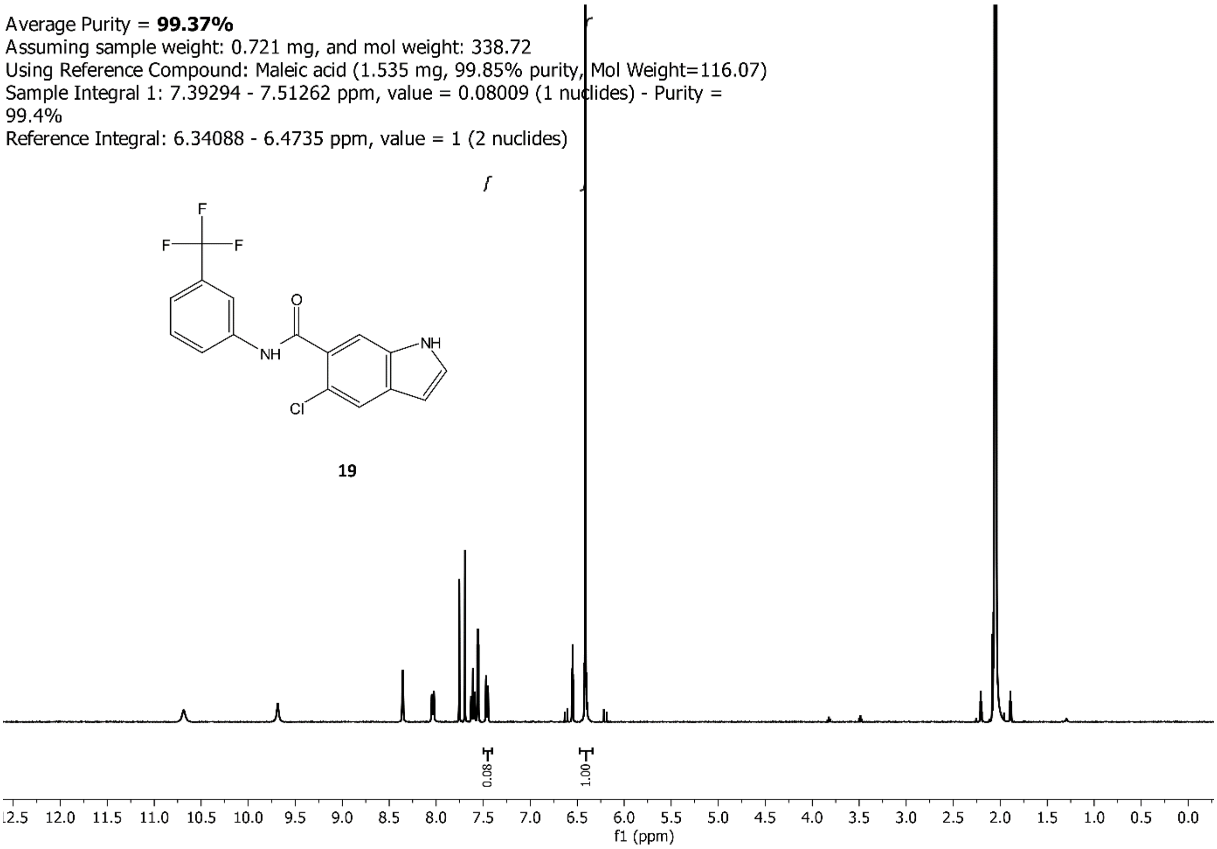

qHNMR (400 MHz, acetone-d$_6$, maleic acid as reference) of compound **19**.

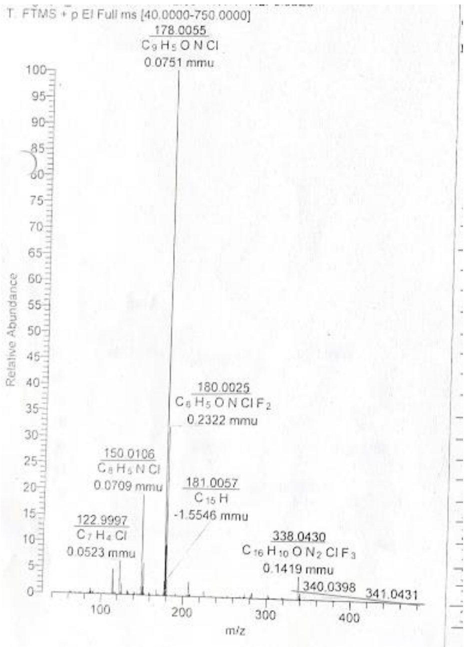

HRMS (EI+) of compound **19**.

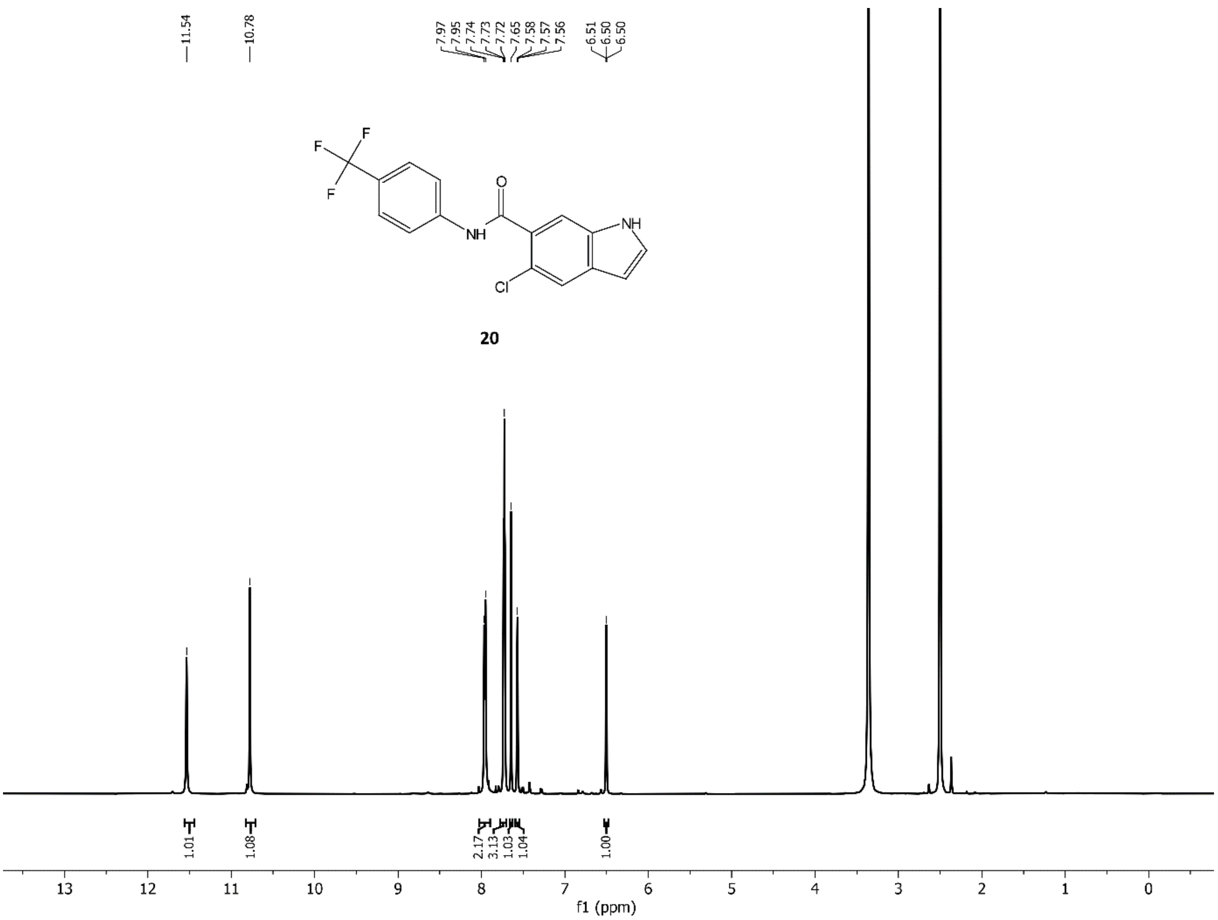

^{1}H-NMR (500 MHz, DMSO-d$_6$) of compound **20.**

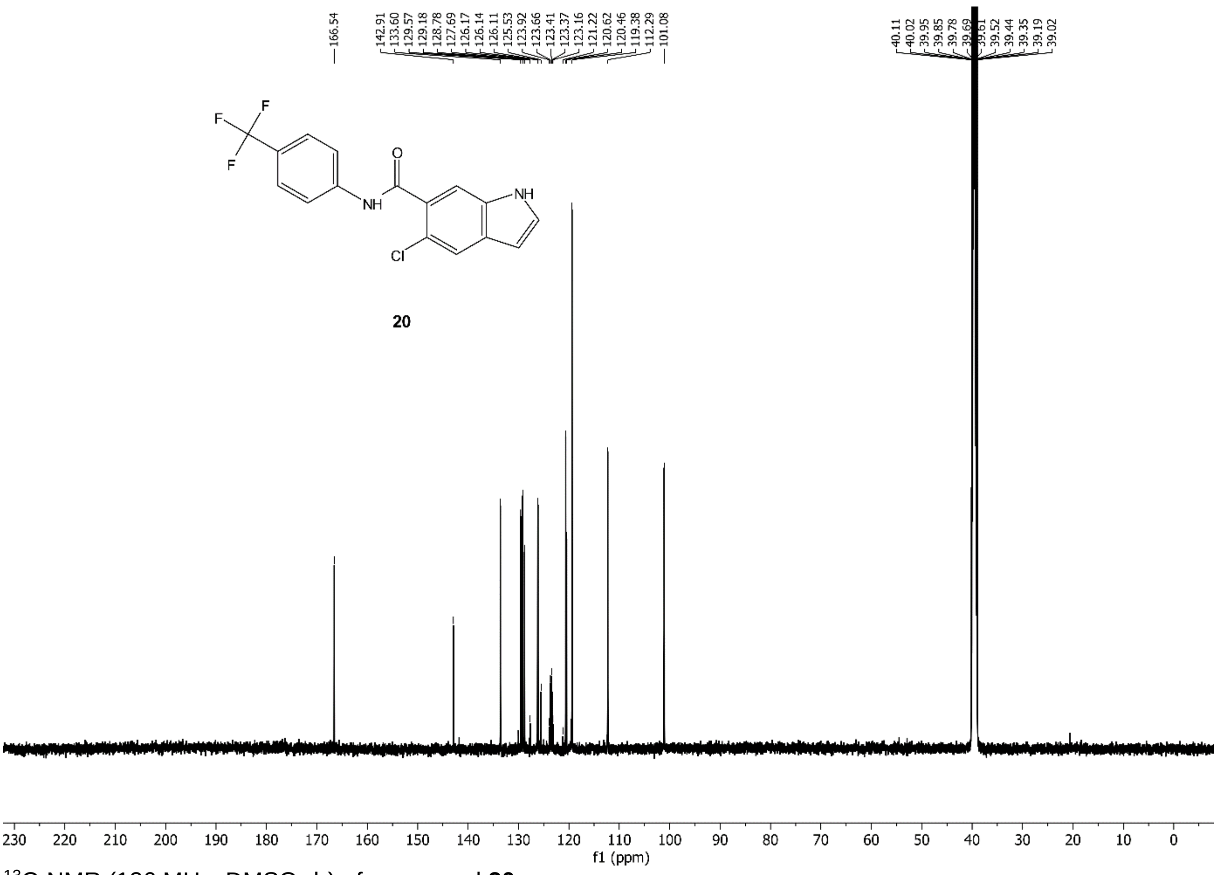

^{13}C-NMR (126 MHz, DMSO-d$_6$) of compound **20**.

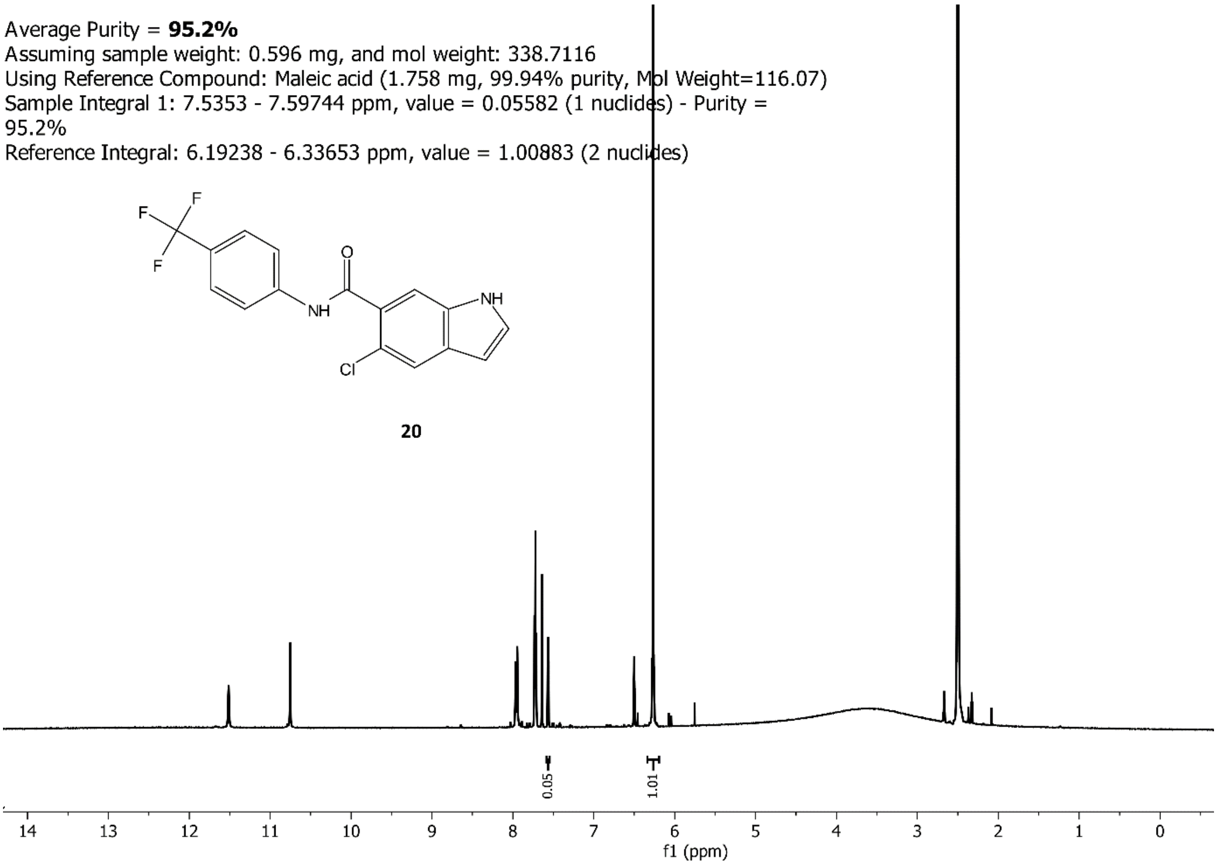

qHNMR (400 MHz, DMSO-d$_6$, maleic acid as reference) of compound **20**.

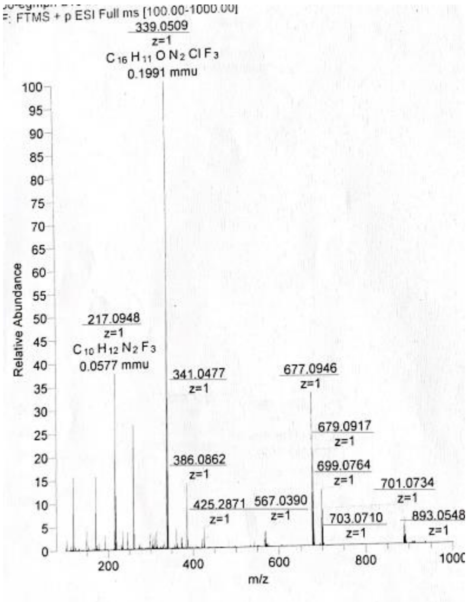

HRMS (ESI+) of compound **20**.

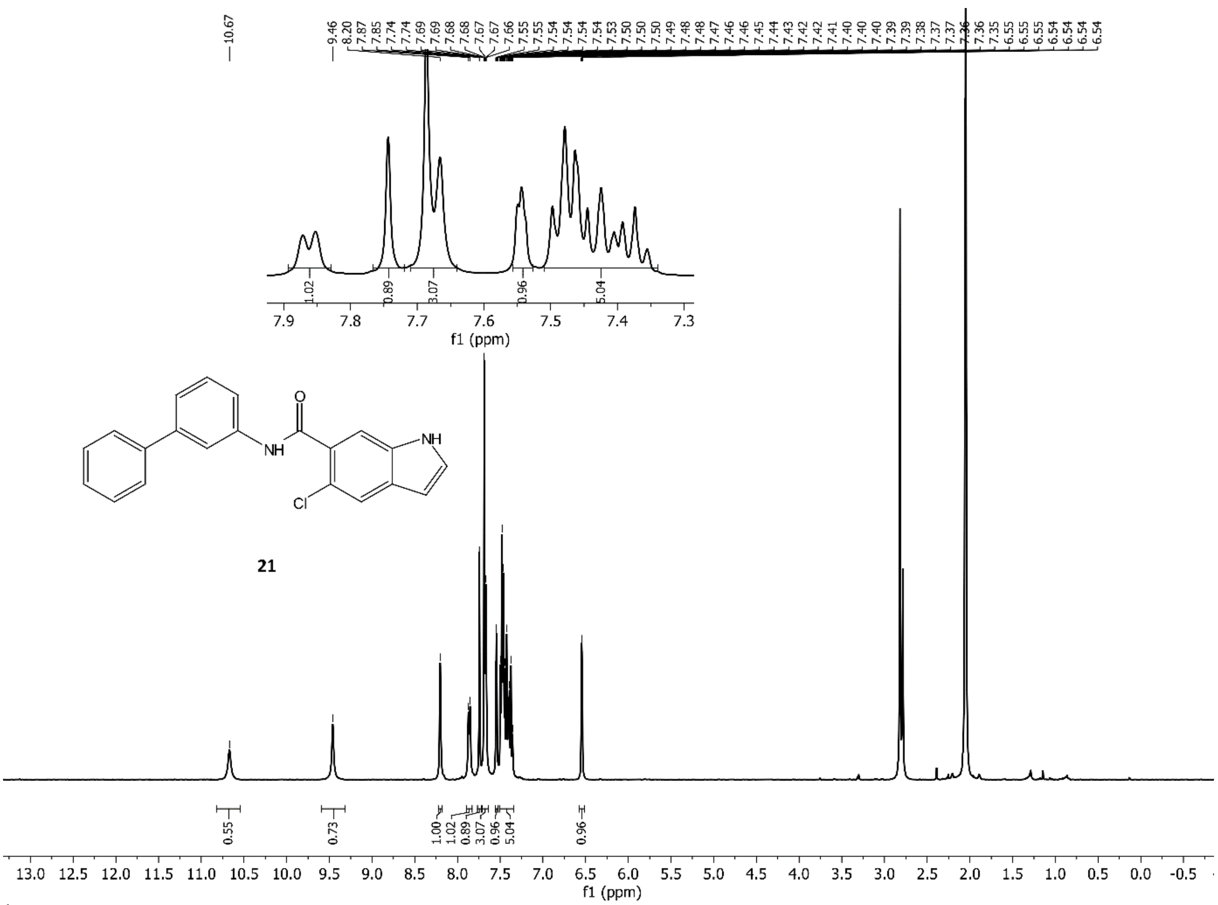

^{1}H-NMR (400 MHz, acetone-d6) of compound **21**.

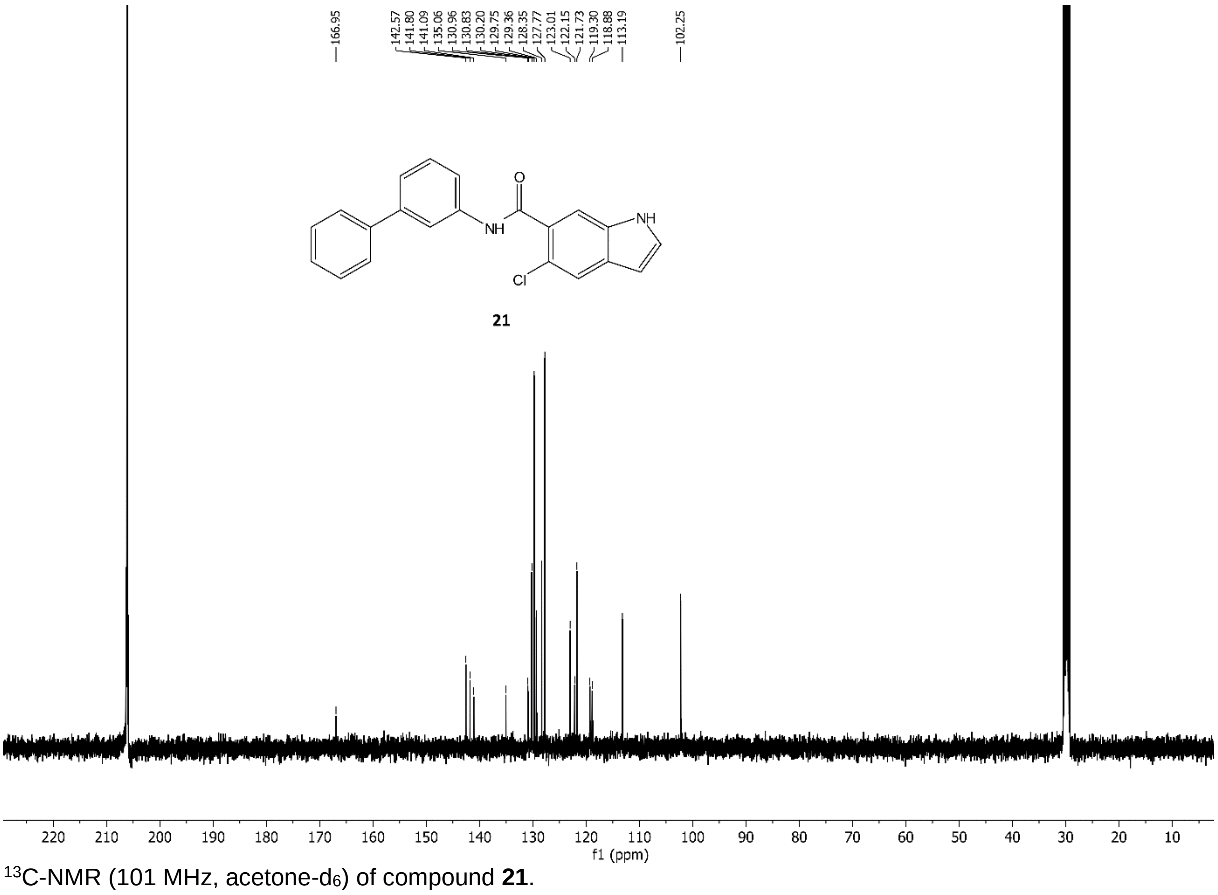

^{13}C-NMR (101 MHz, acetone-d6) of compound **21**.

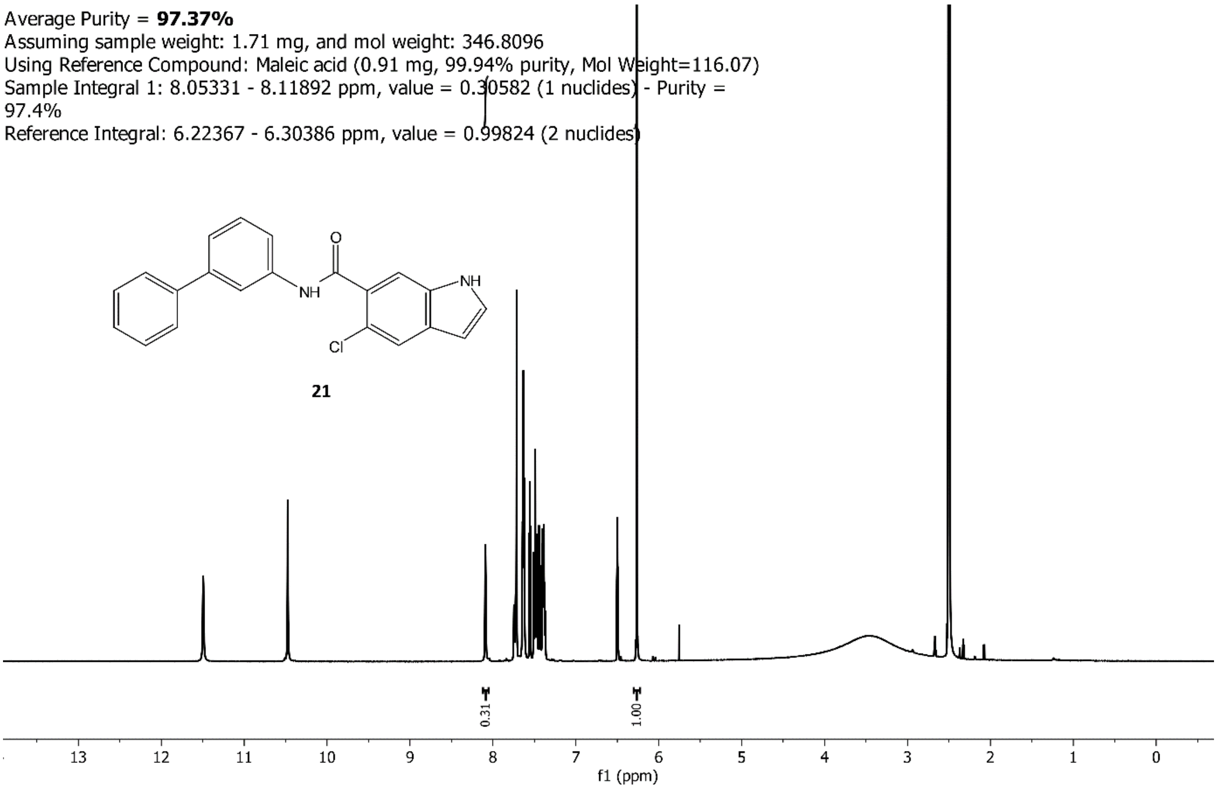

qHNMR (400 MHz, DMSO-d$_6$, maleic acid as reference) of compound **21**.

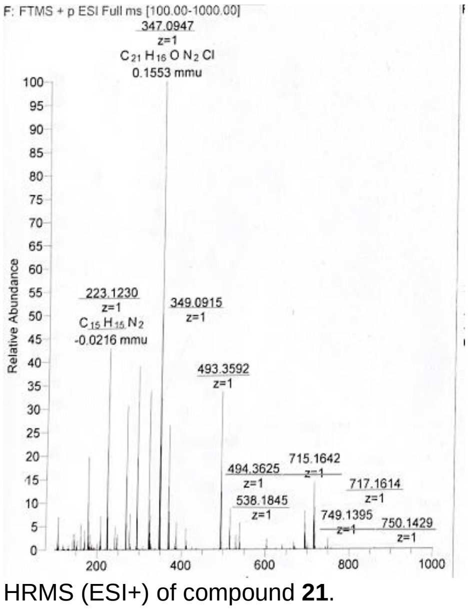

HRMS (ESI+) of compound **21**.

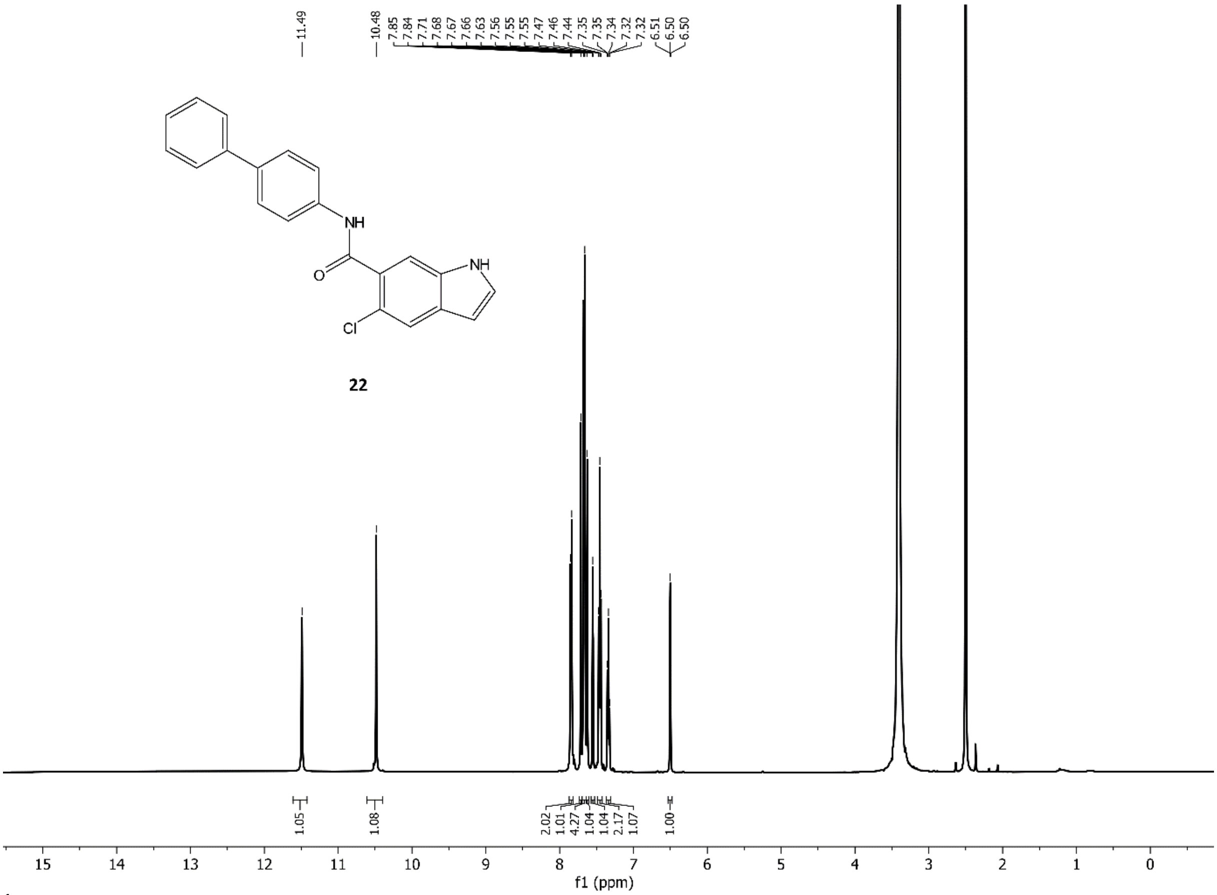

^{1}H-NMR (500 MHz, DMSO-d6) of compound **22.**

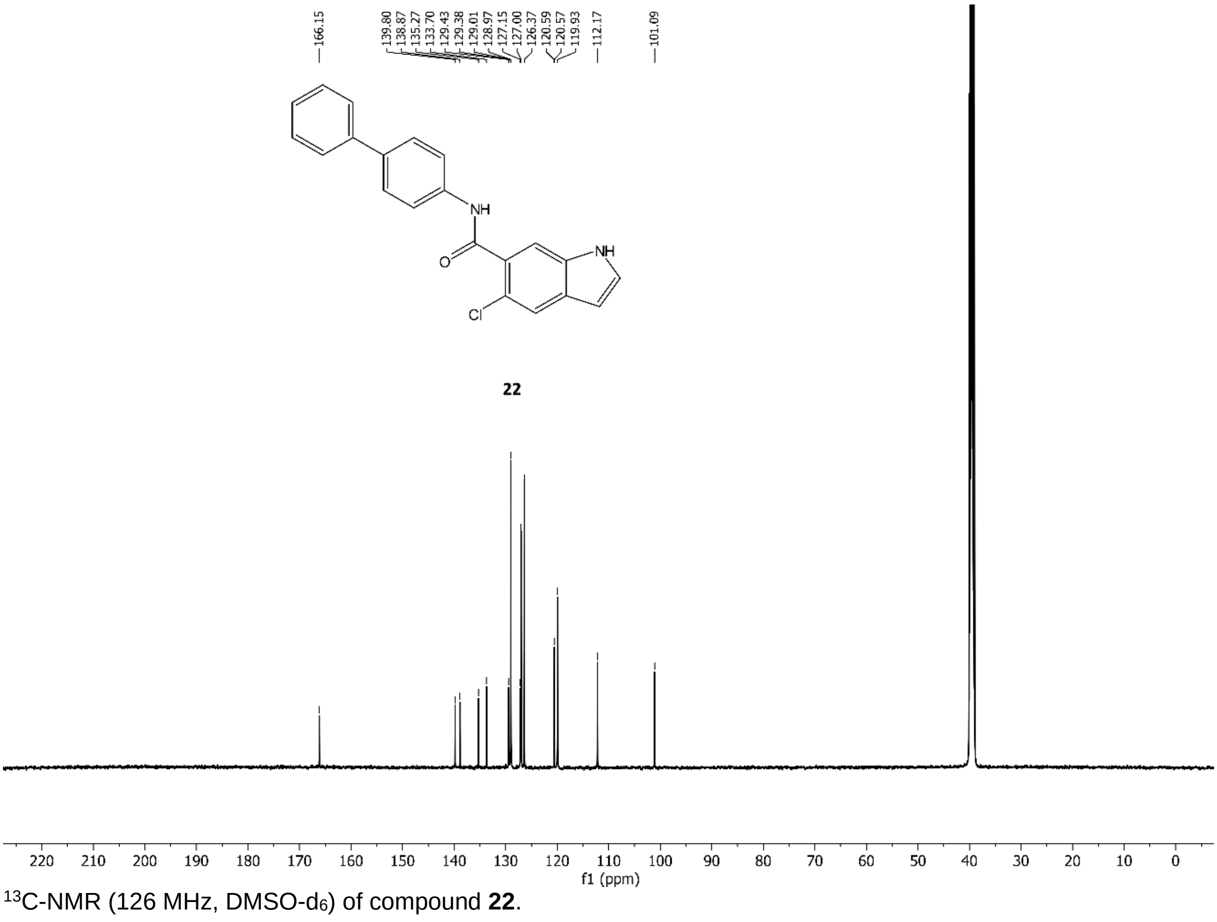

^{13}C-NMR (126 MHz, DMSO-d6) of compound **22**.

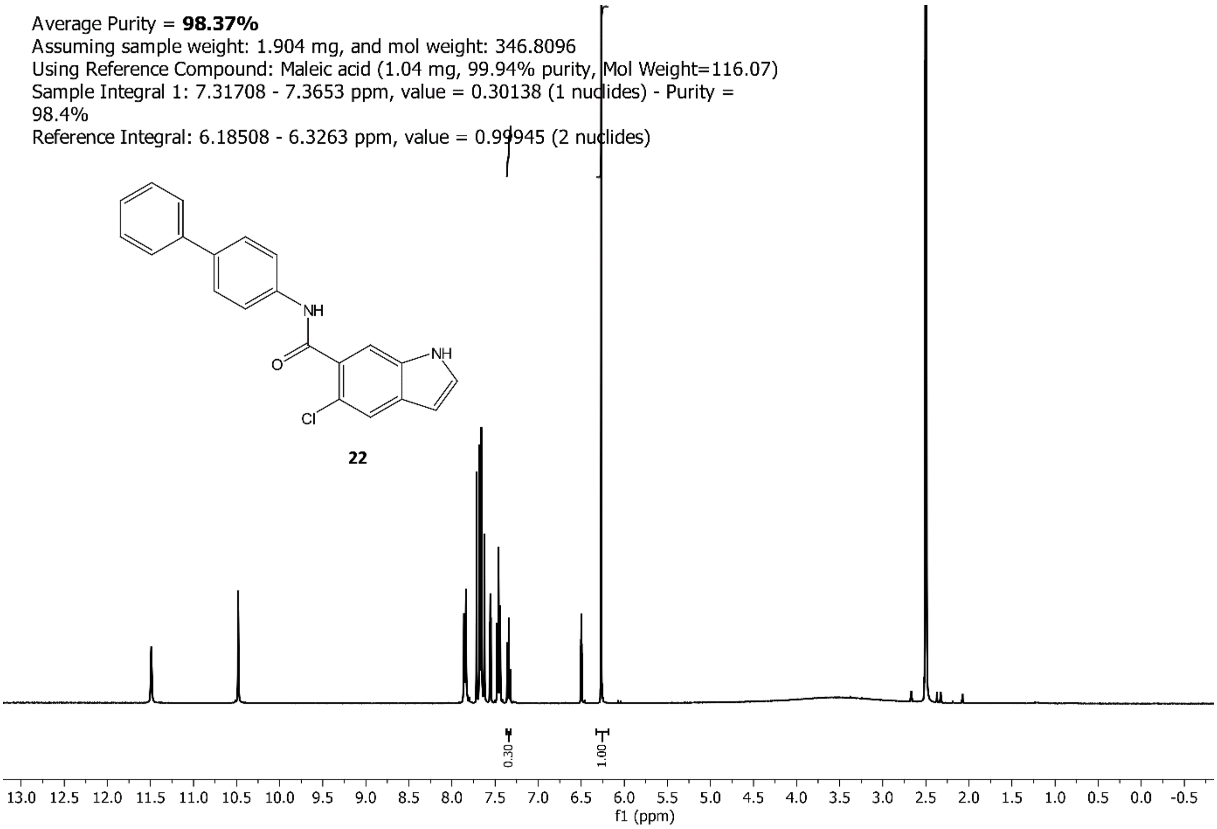

qHNMR (400 MHz, DMSO-d_6, maleic acid as reference) of compound **22**.

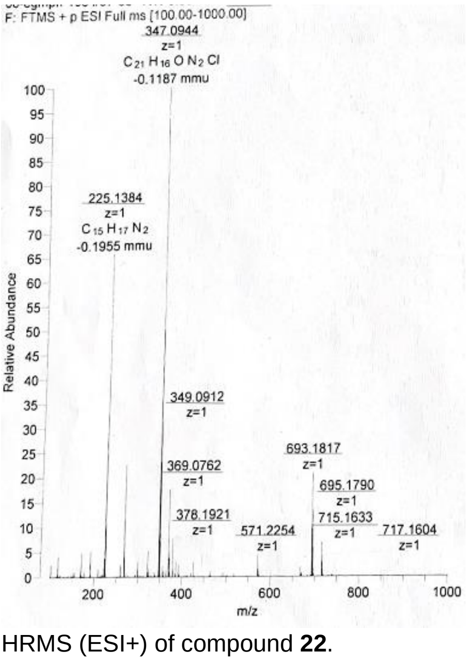

HRMS (ESI+) of compound **22**.

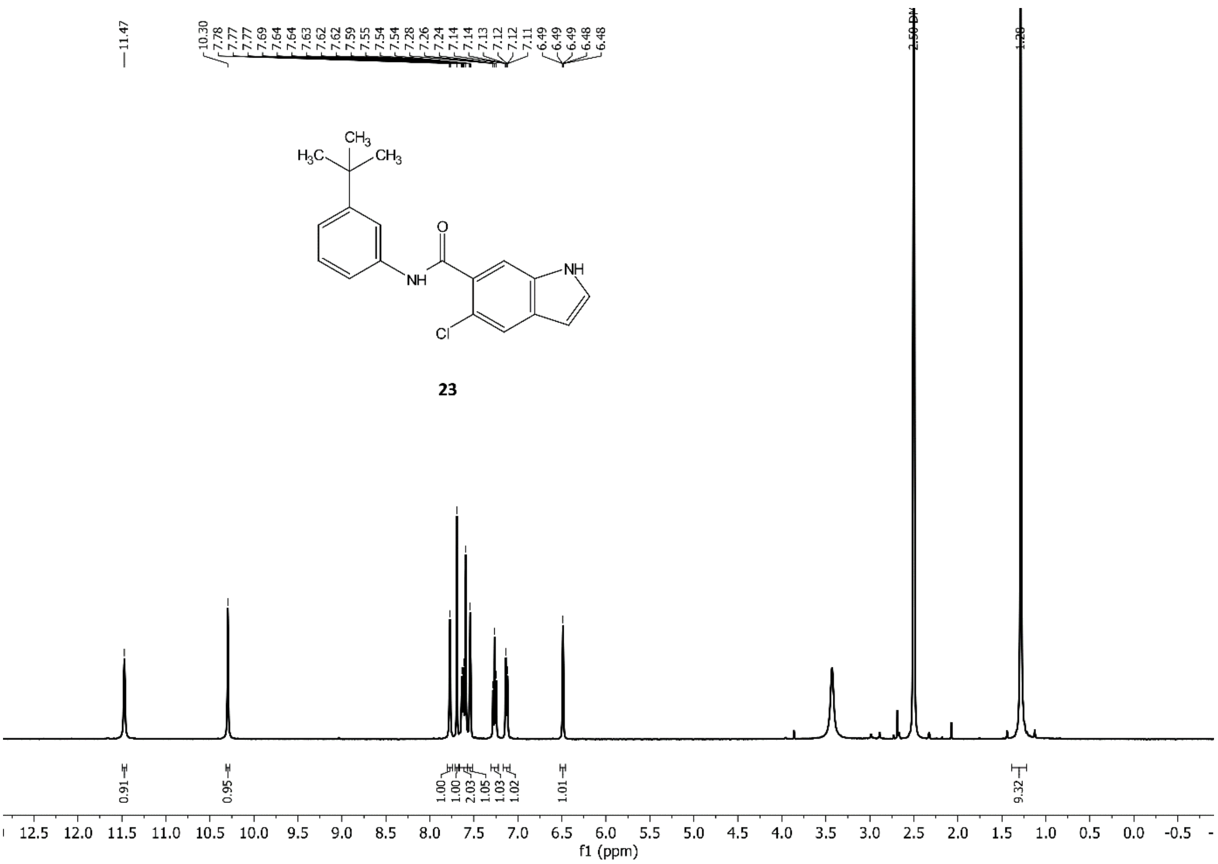

^{1}H-NMR (400 MHz, DMSO-d_6) of compound **23.**

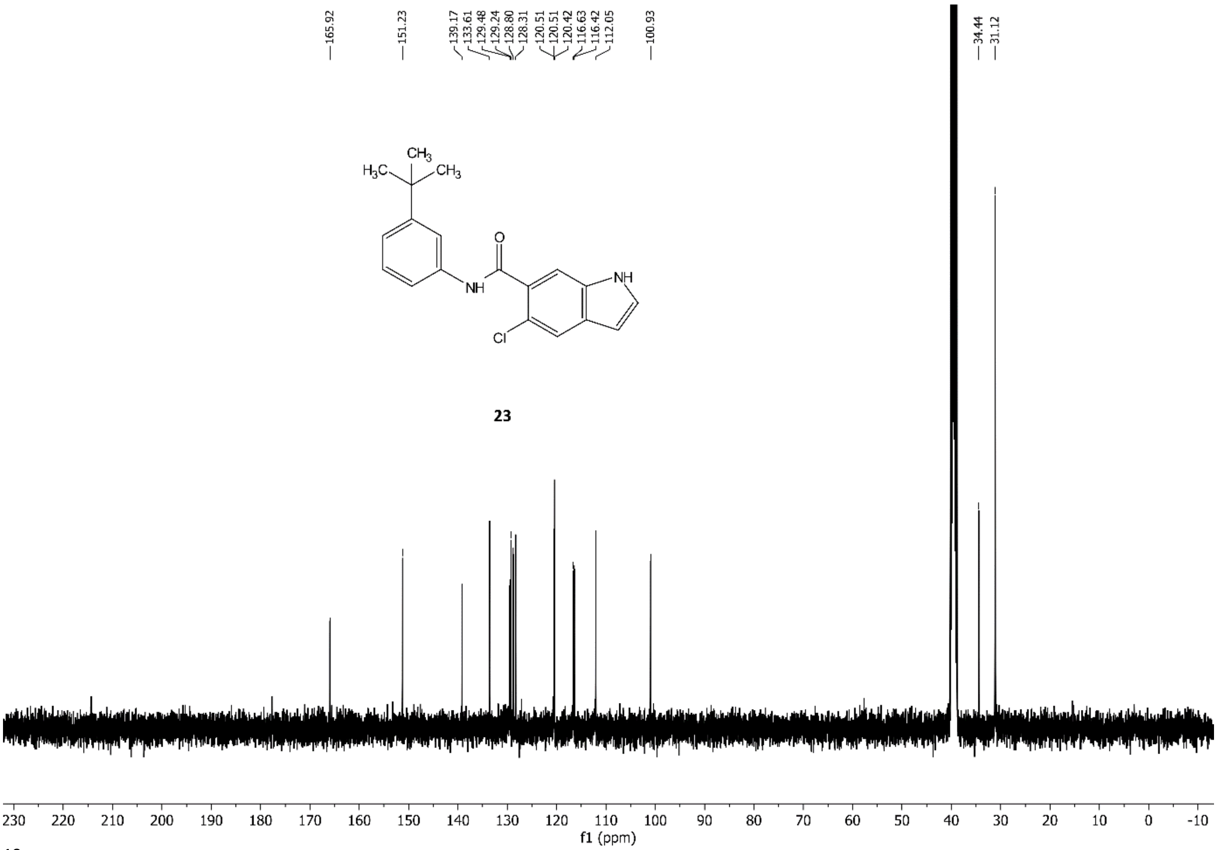

^{13}C-NMR (101 MHz, DMSO-d_6) of compound **23**.

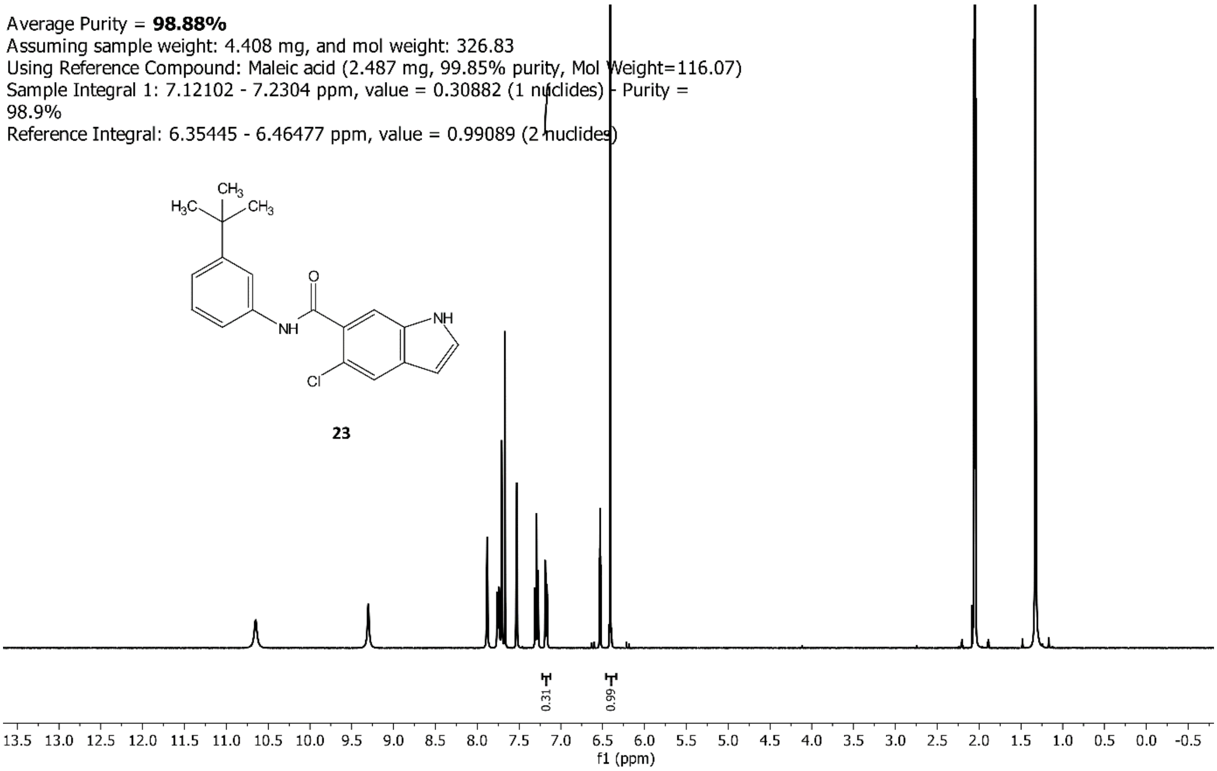

qHNMR (400 MHz, acetone-d$_6$, maleic acid as reference) of compound **23**.

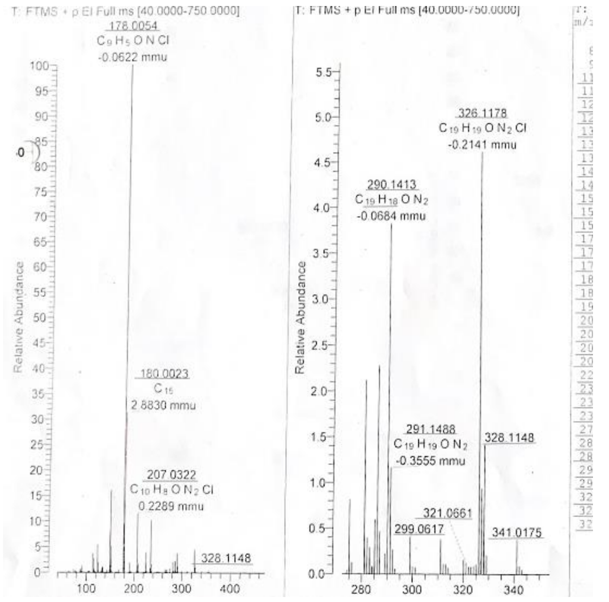

HRMS (EI+) of compound **23**. Left panel: Full spectrum. Right panel: Zoom in.

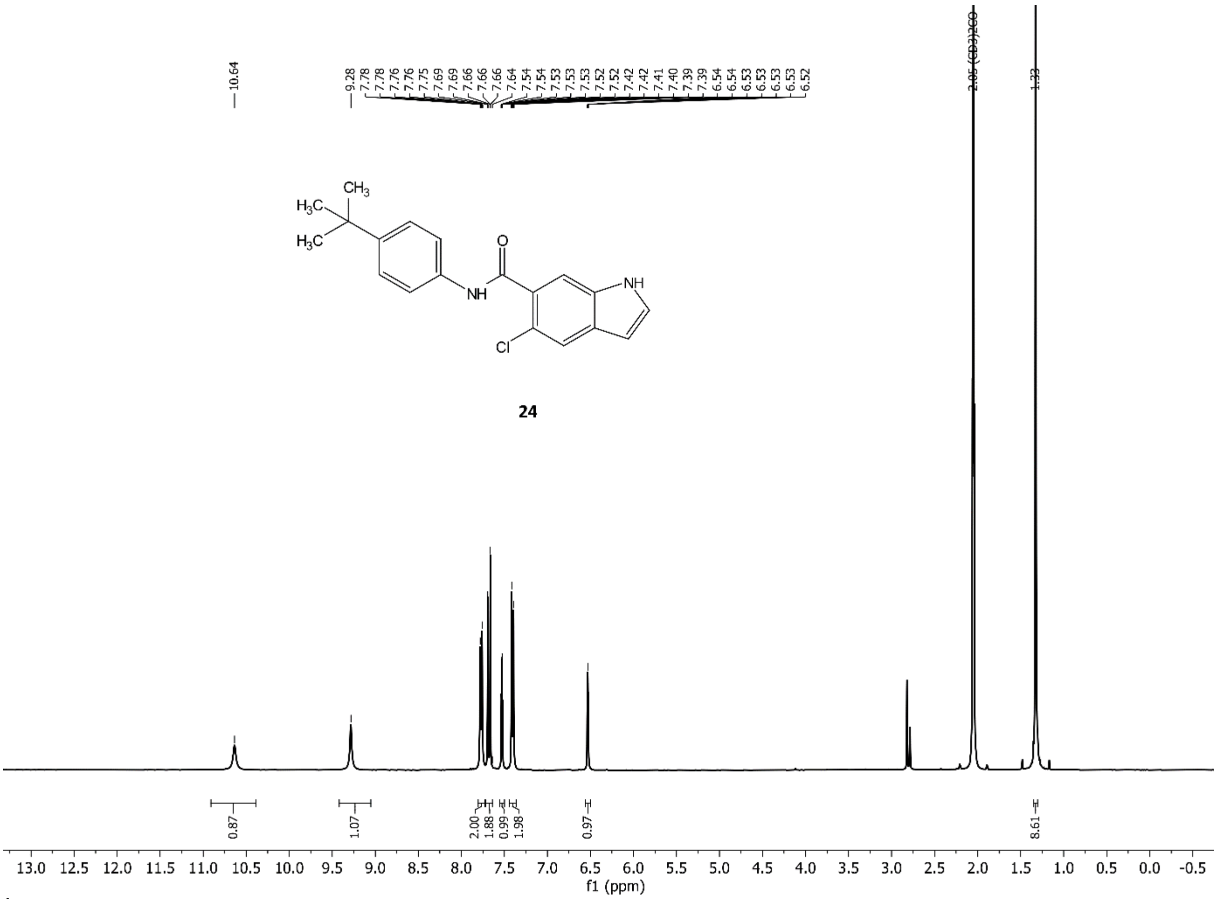

^{1}H-NMR (400 MHz, acetone-d_6) of compound **24.**

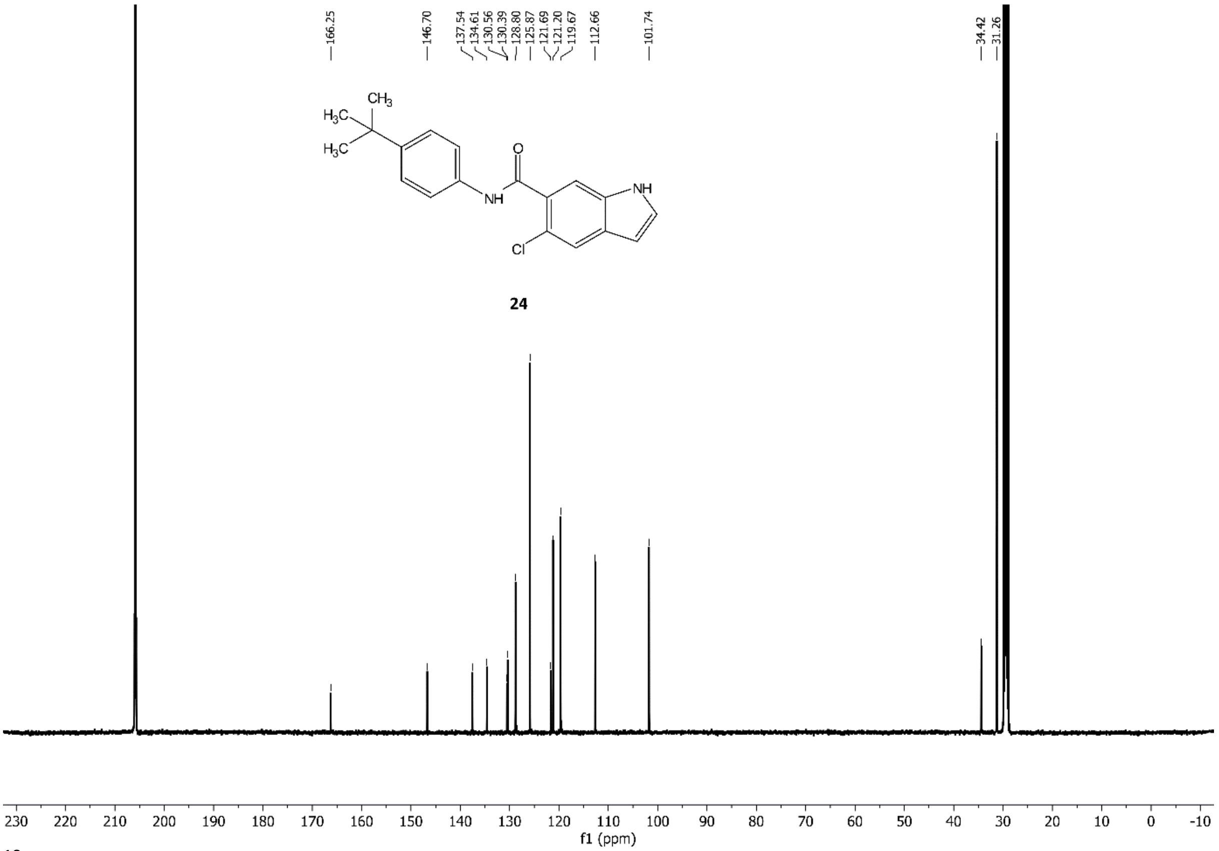

^{13}C-NMR (101 MHz, acetone-d_6) of compound **24**.

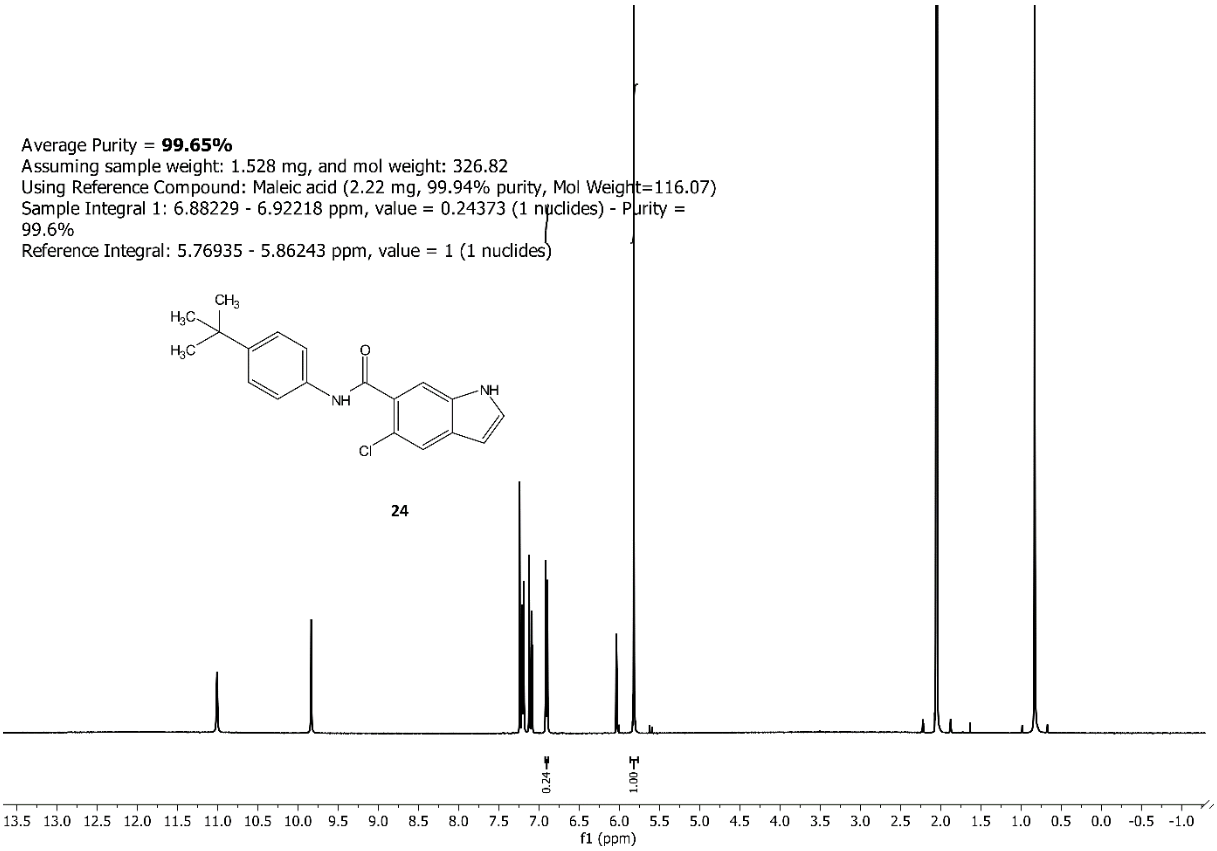

qHNMR (400 MHz, acetone-d_6, maleic acid as reference) of compound **24**.

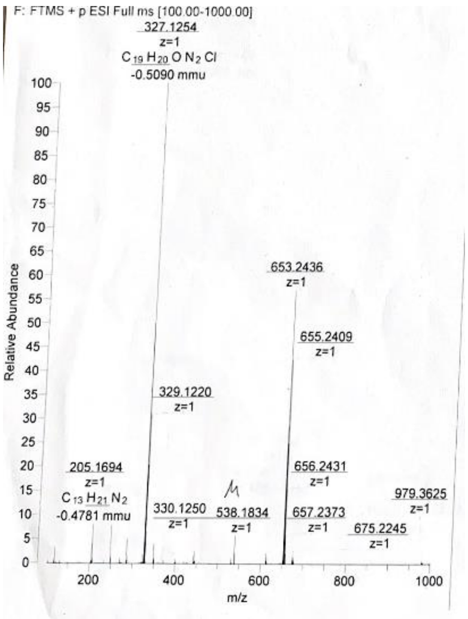

HRMS (ESI+) of compound **24**.

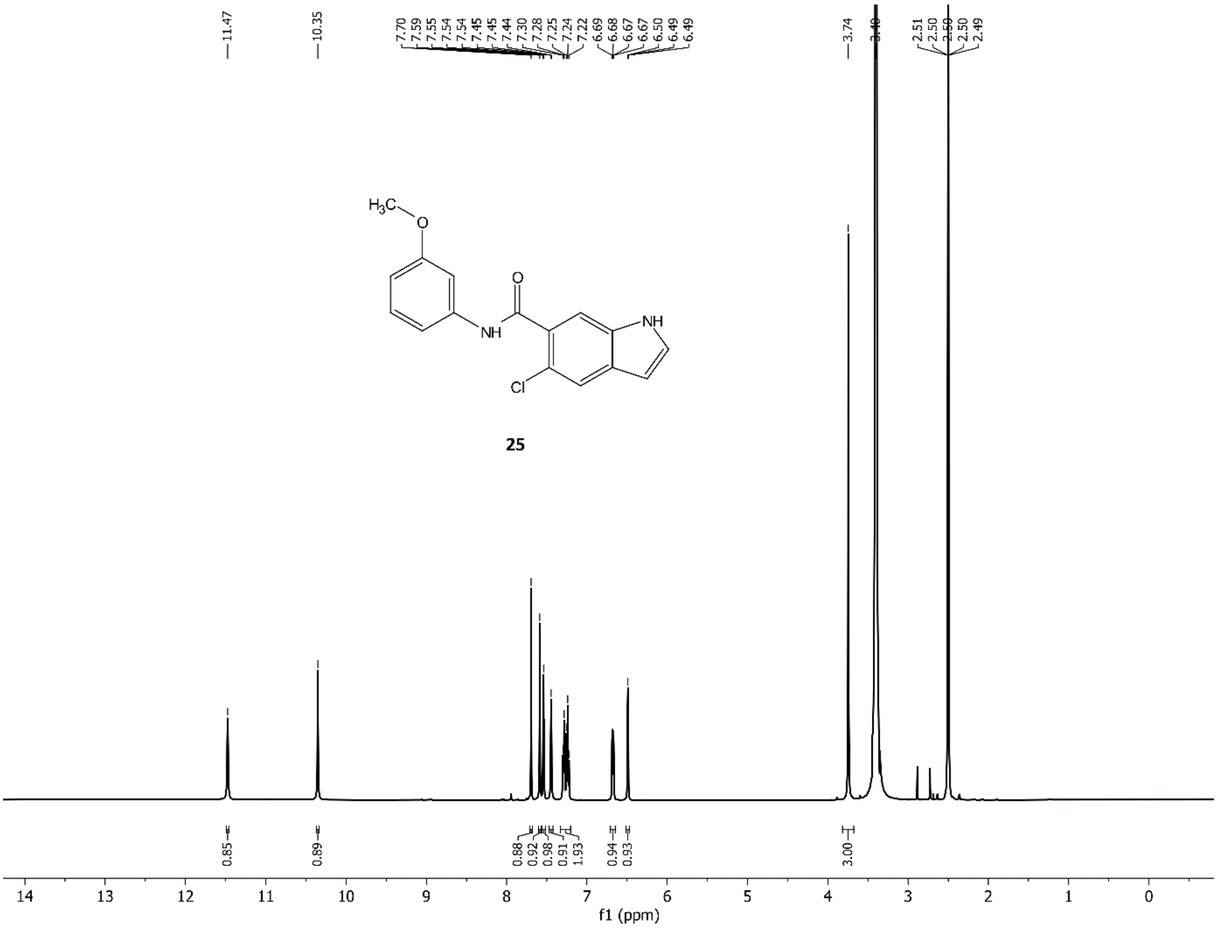

^{1}H-NMR (500 MHz, DMSO-d$_6$) of compound **25**.

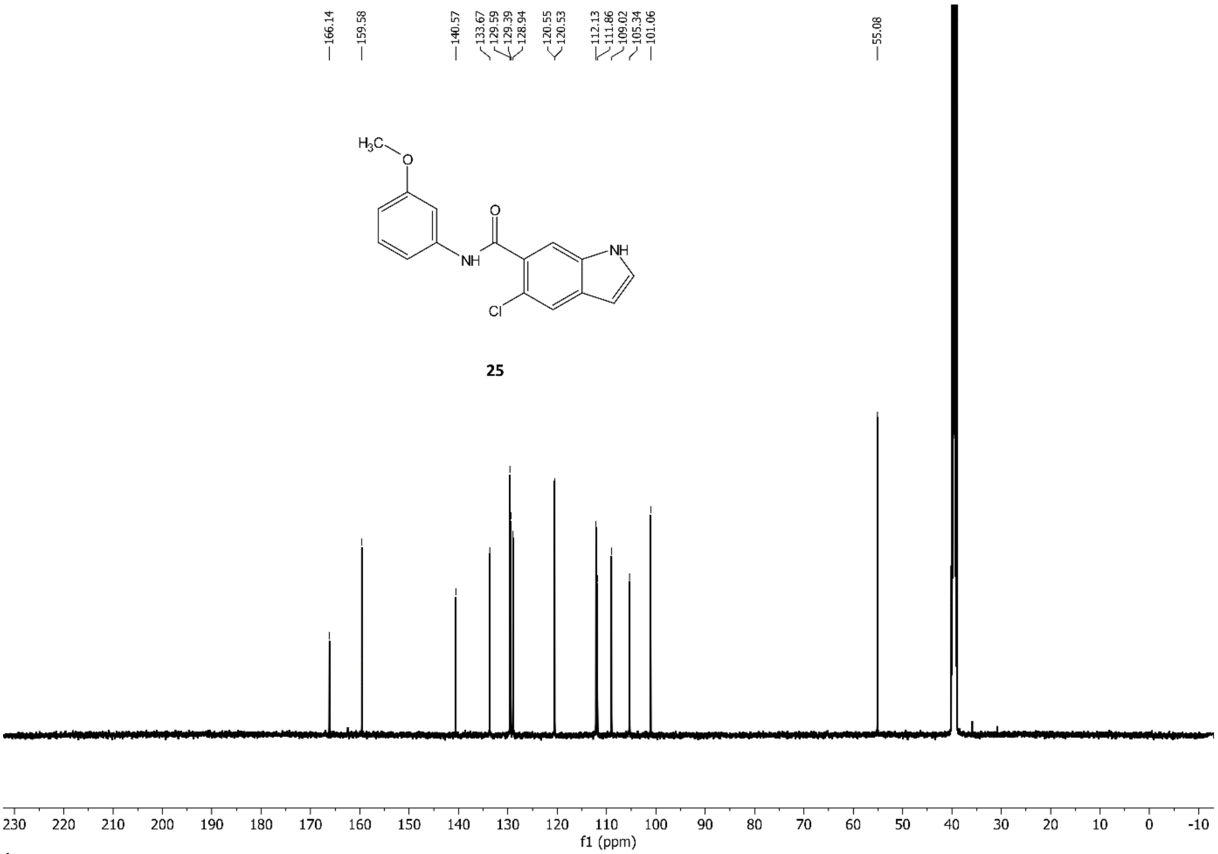

^{1}H-NMR (126 MHz, DMSO-d$_6$) of compound **25**.

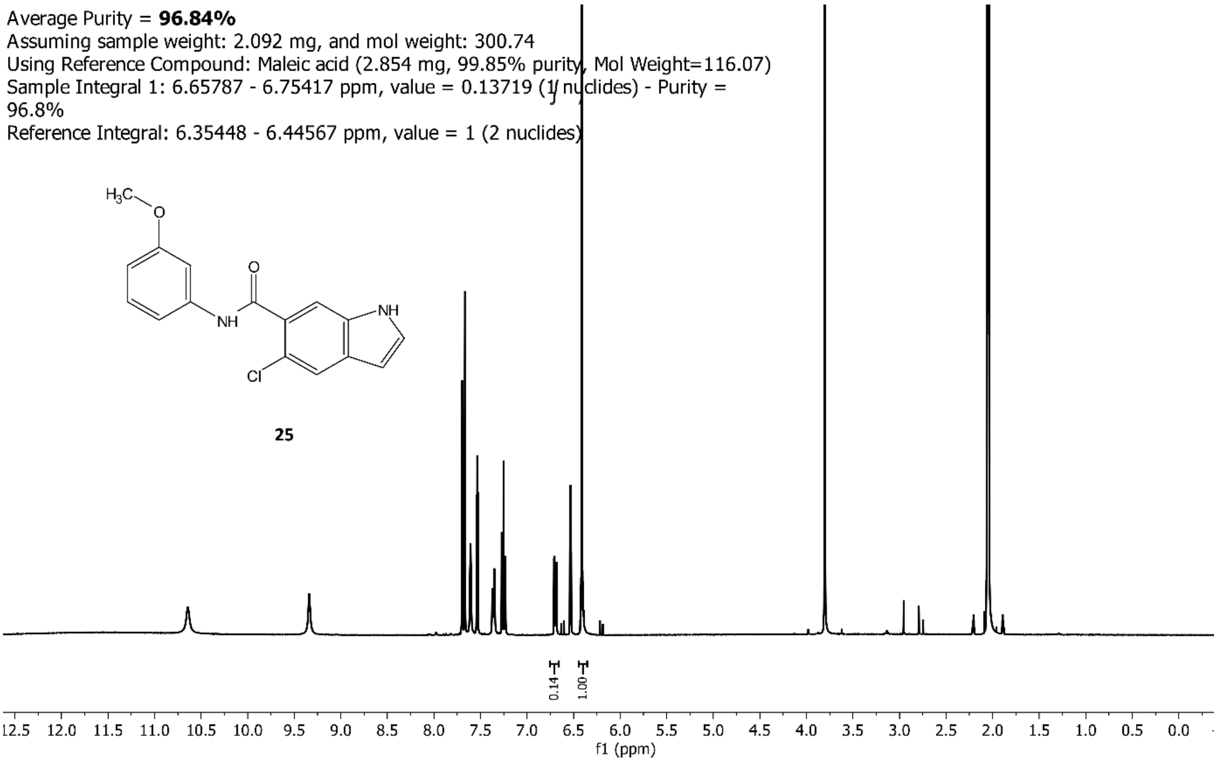

qHNMR (400 MHz, acetone-d_6, maleic acid as reference) of compound **25**.

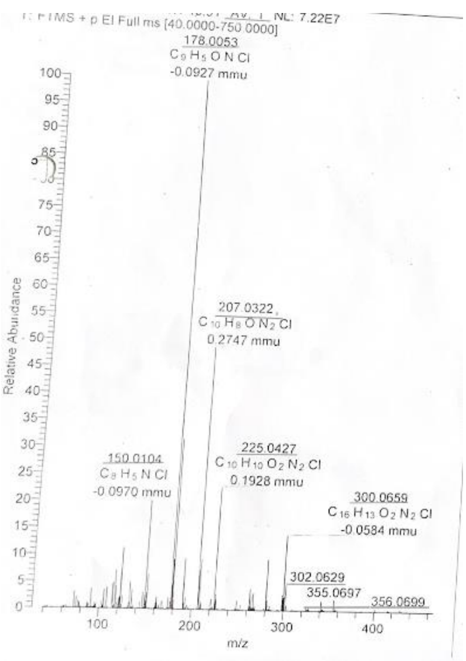

HRMS (EI+) of compound **25**.

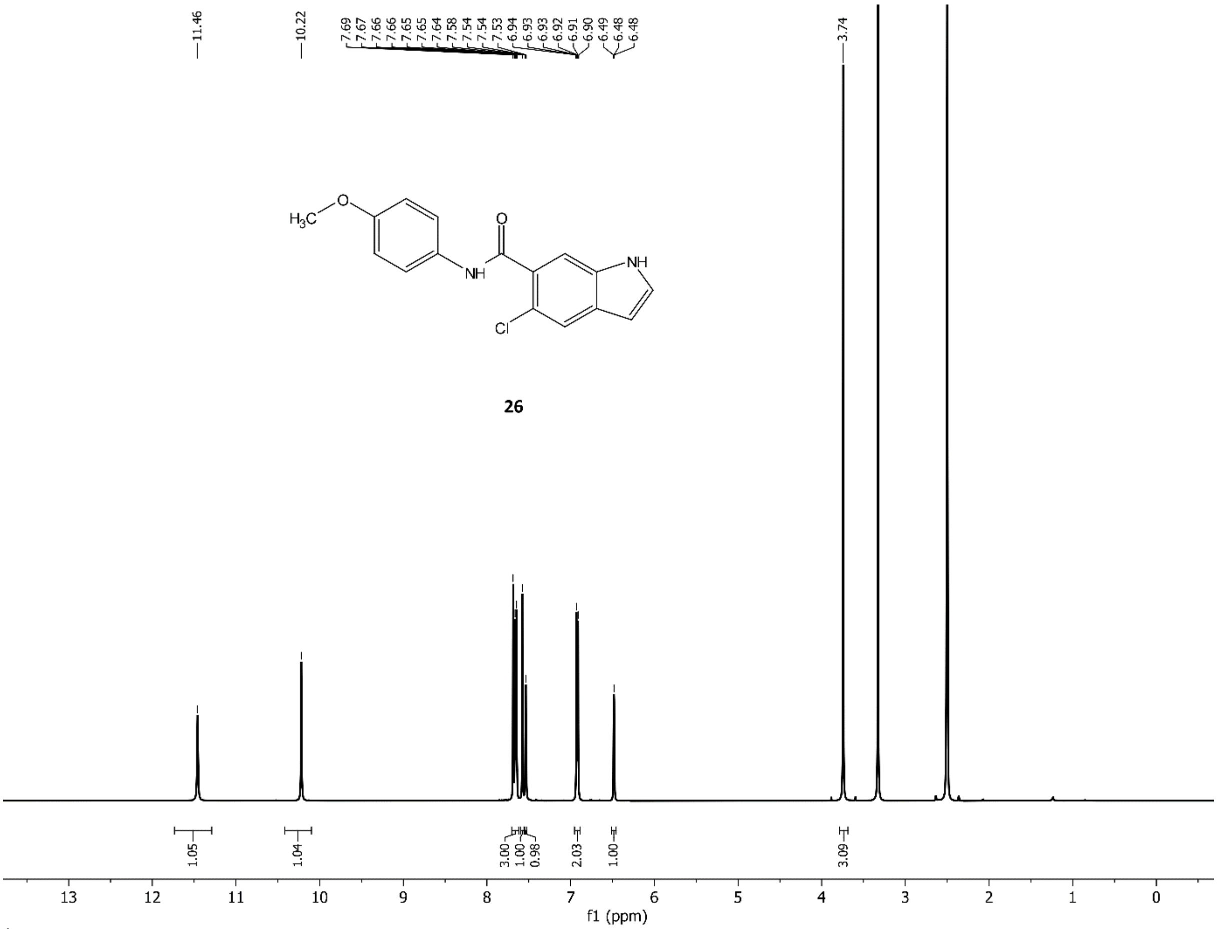

^{1}H-NMR (500 MHz, DMSO-d_6) of compound **26**.

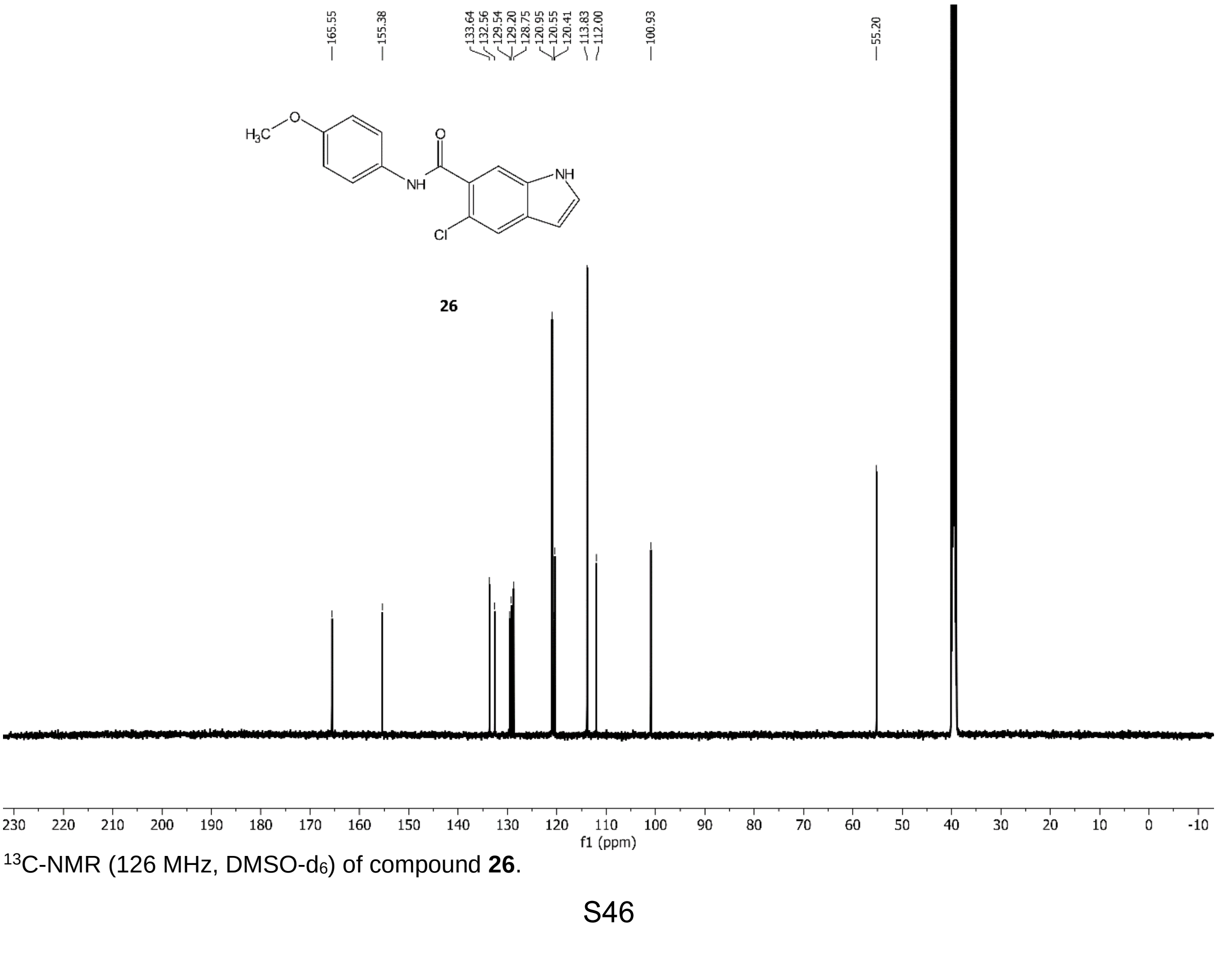

^{13}C-NMR (126 MHz, DMSO-d_6) of compound **26**.

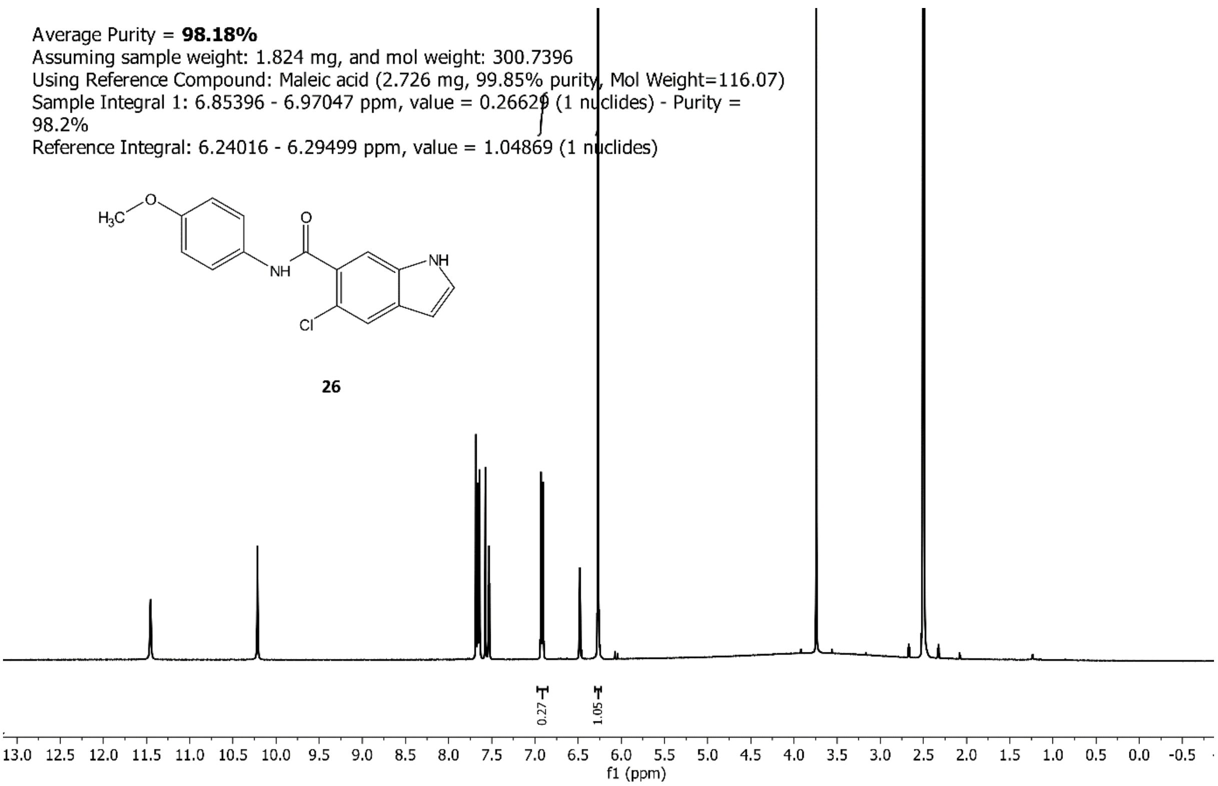

qHNMR (400 MHz, DMSO-d$_6$, maleic acid as reference) of compound **26**.

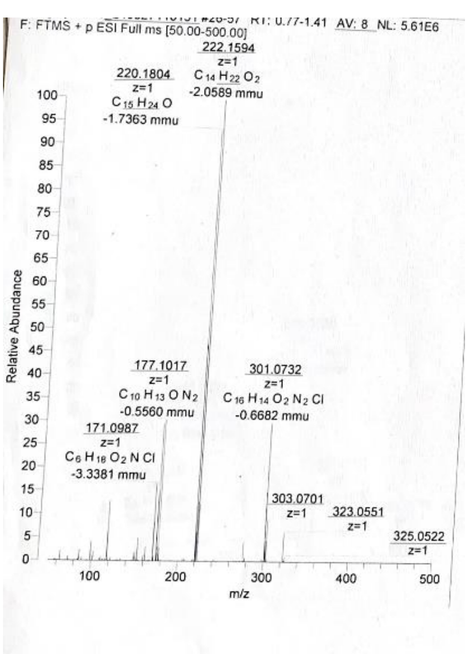

HRMS (ESI+) of compound **26**.

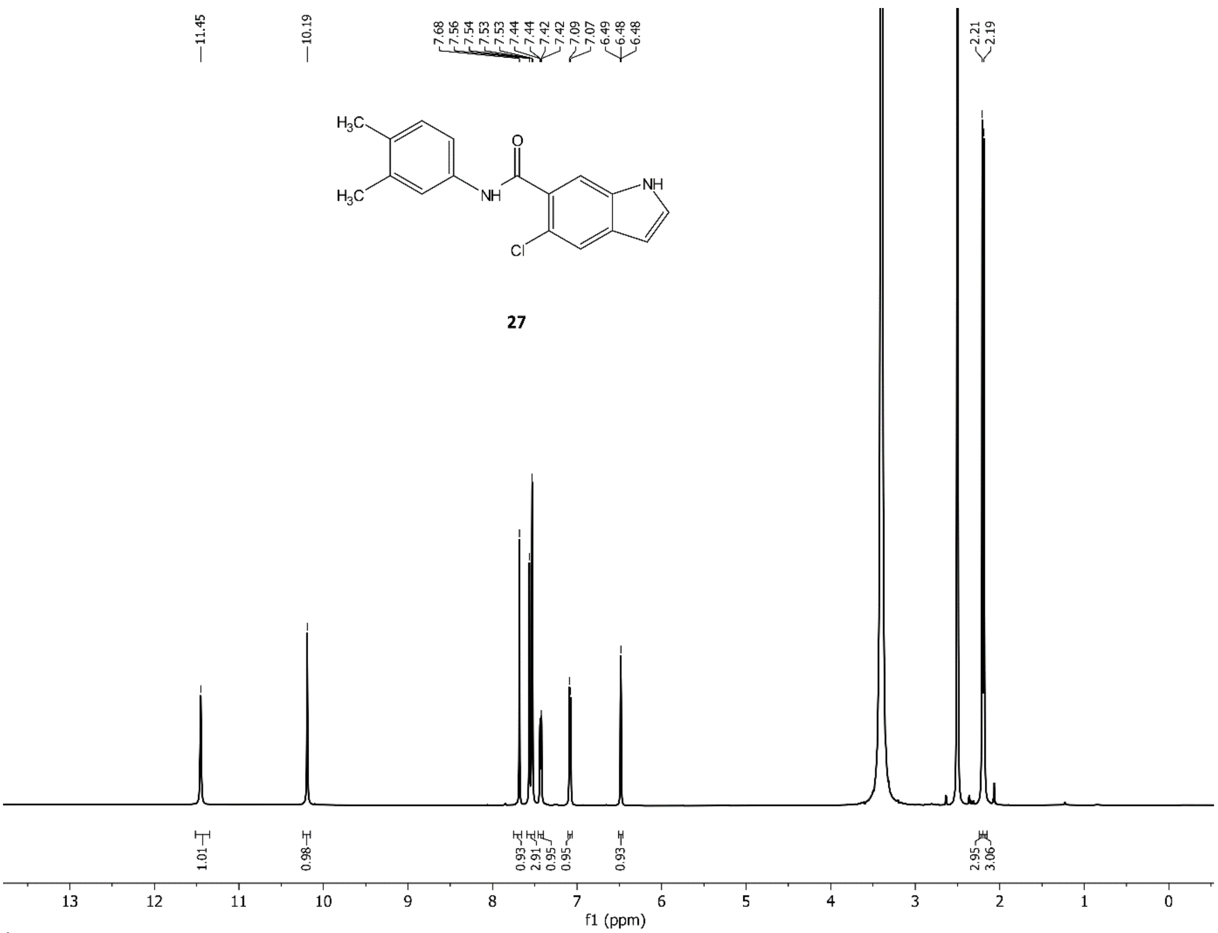

27

^{1}H-NMR (500 MHz, DMSO-d6) of compound **27**.

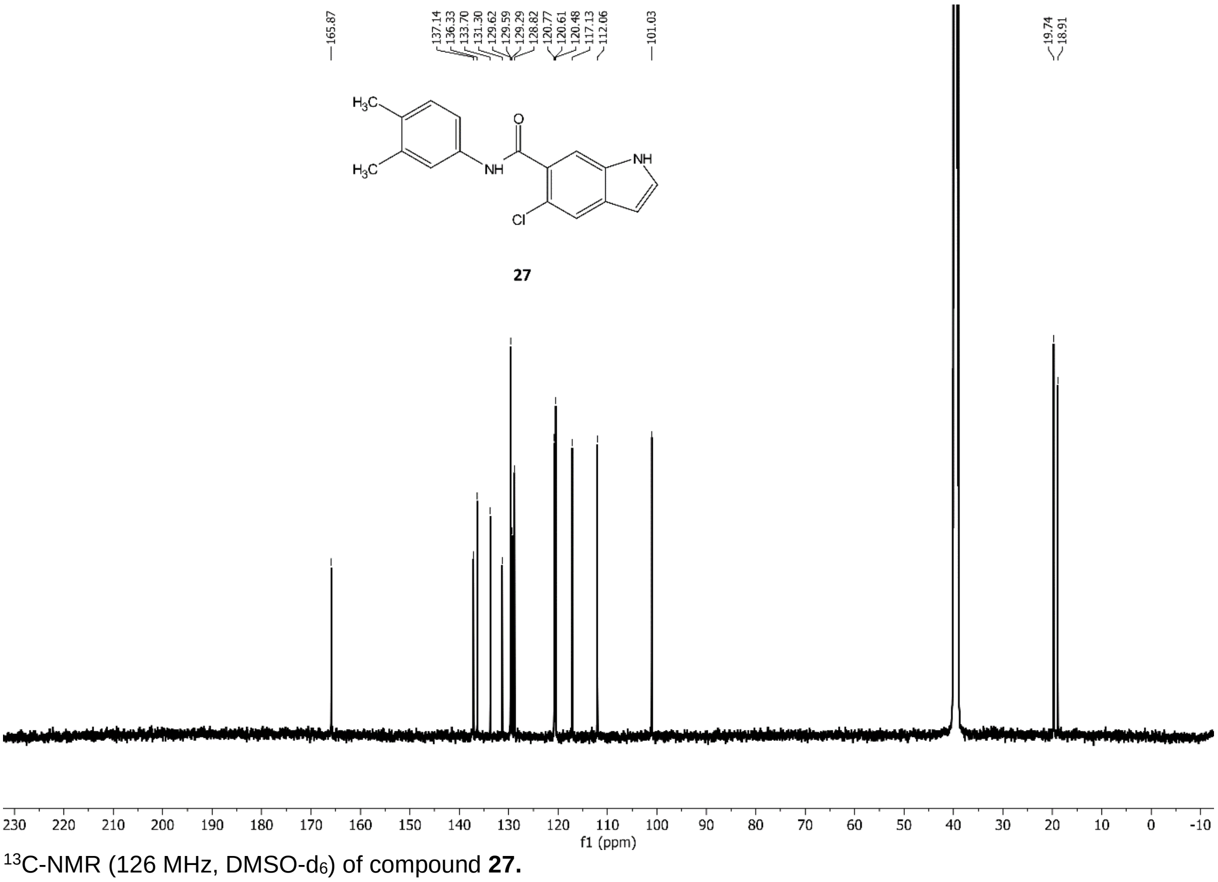

27

^{13}C-NMR (126 MHz, DMSO-d6) of compound **27.**

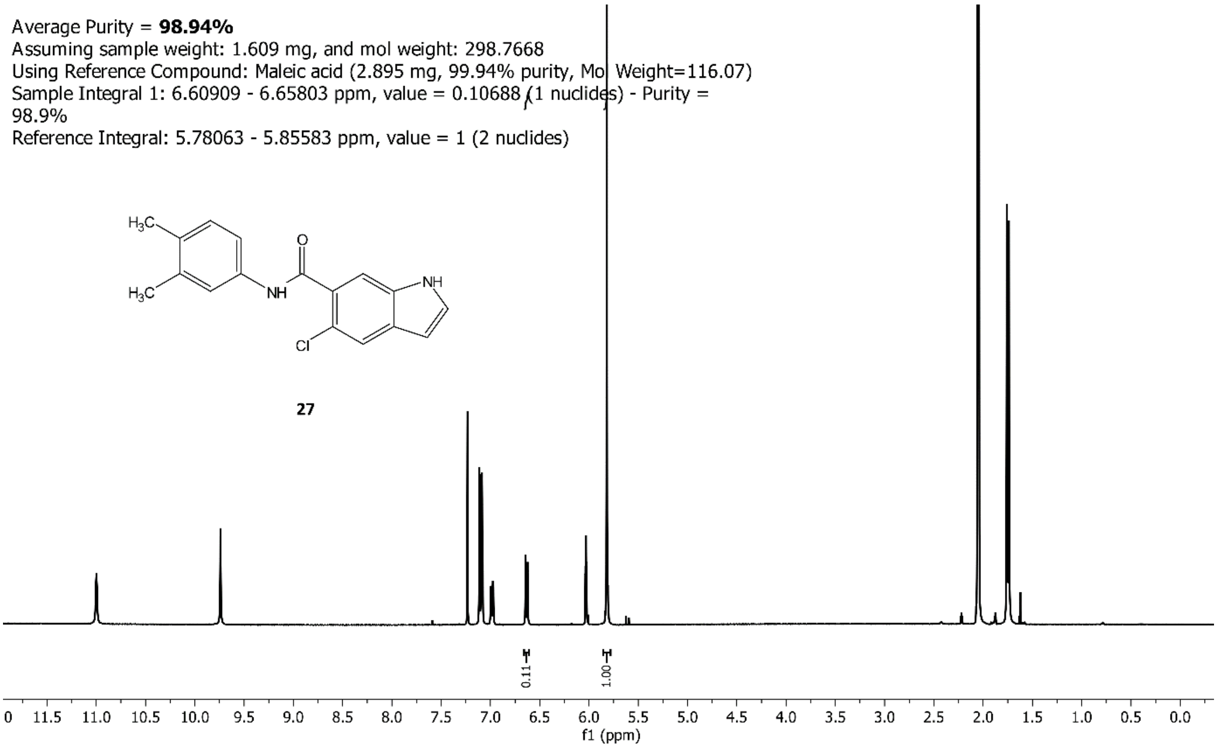

qHNMR (400 MHz, acetone-d$_6$, maleic acid as reference) of compound **27**.

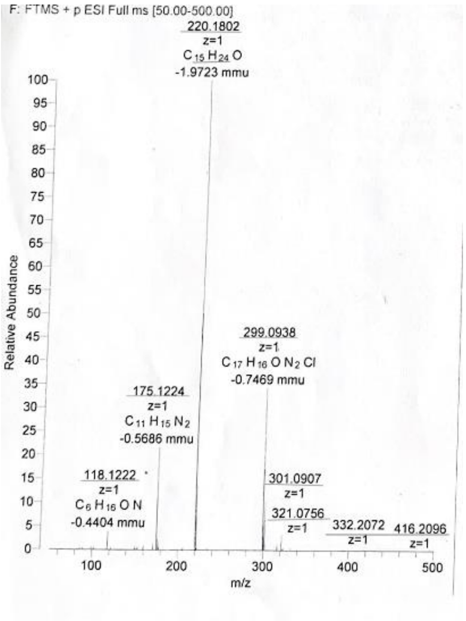

HRMS (ESI+) of compound **27**.

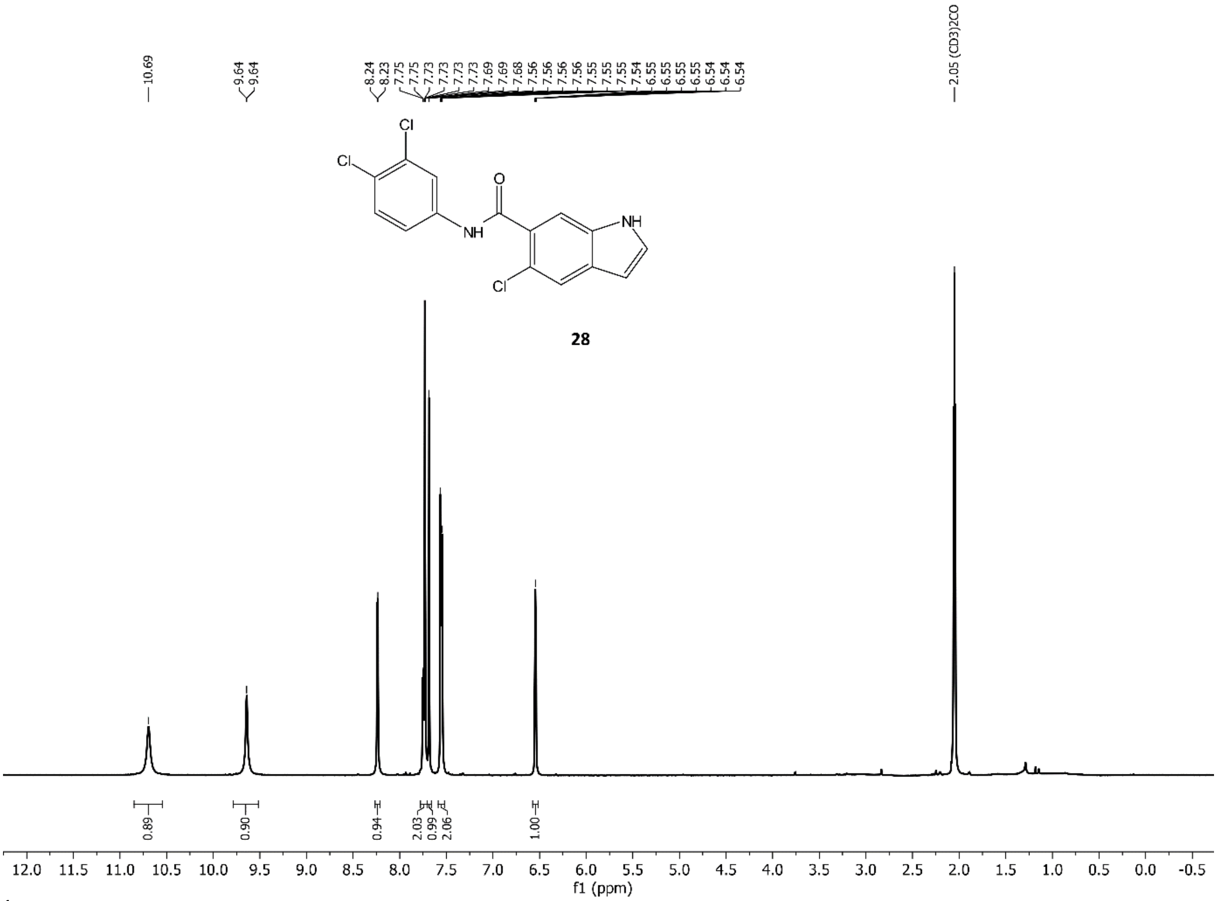

^{1}H-NMR (400 MHz, acetone-d$_6$) of compound **28**.

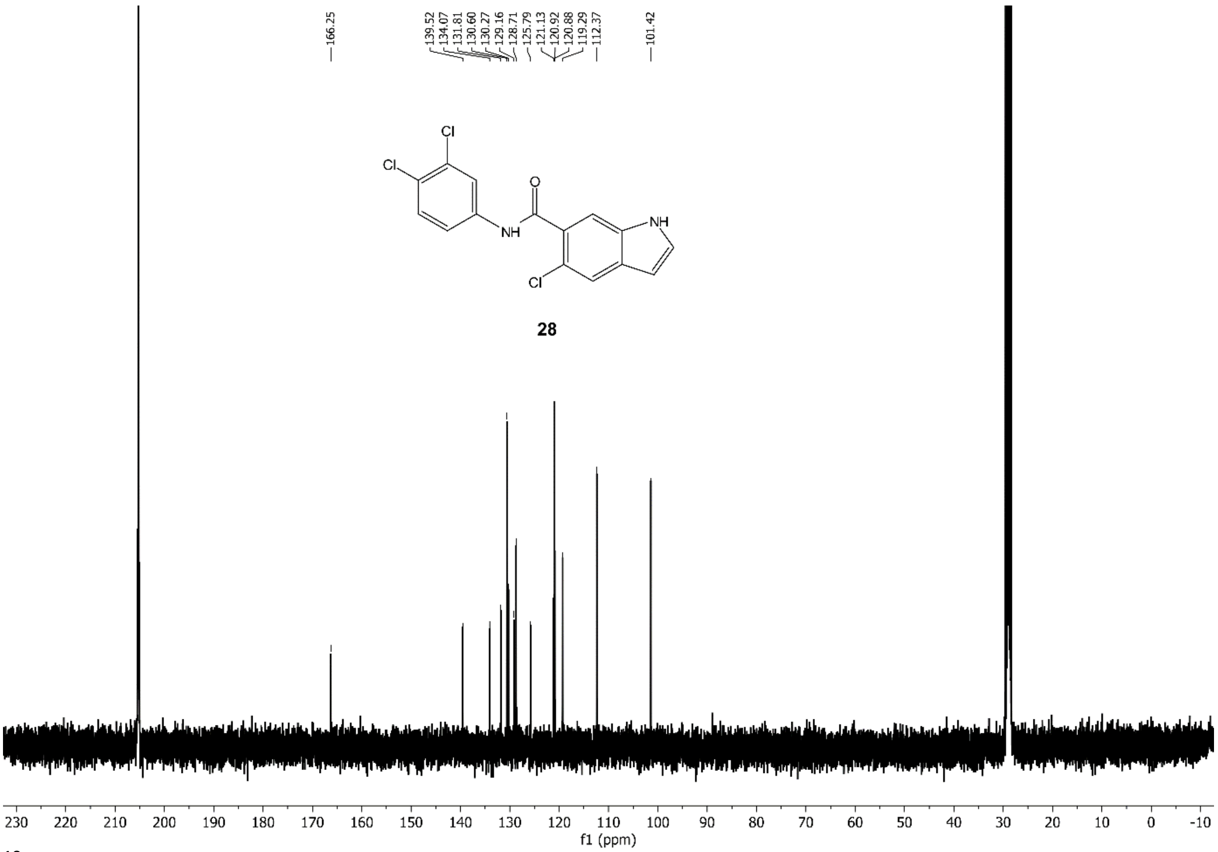

^{13}C-NMR (101 MHz, acetone-d$_6$) of compound **28**.

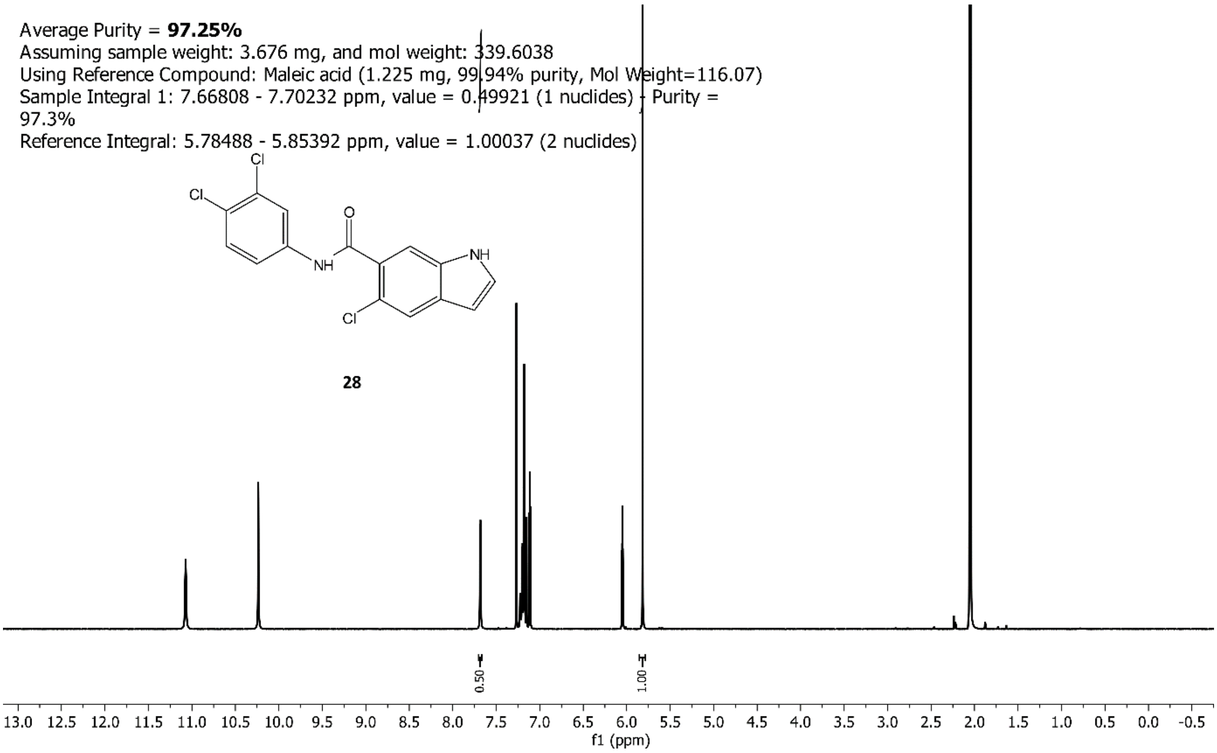

qHNMR (400 MHz, acetone-d_6, maleic acid as reference) of compound **28**.

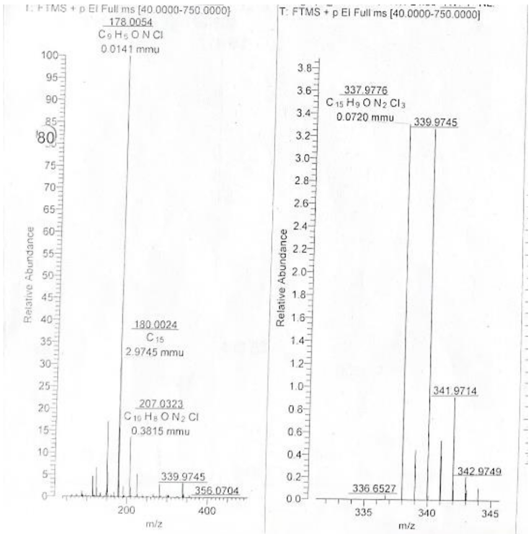

HRMS (EI+) of compound **28**. Left panel: Full spectrum. Right panel: Zoom in.

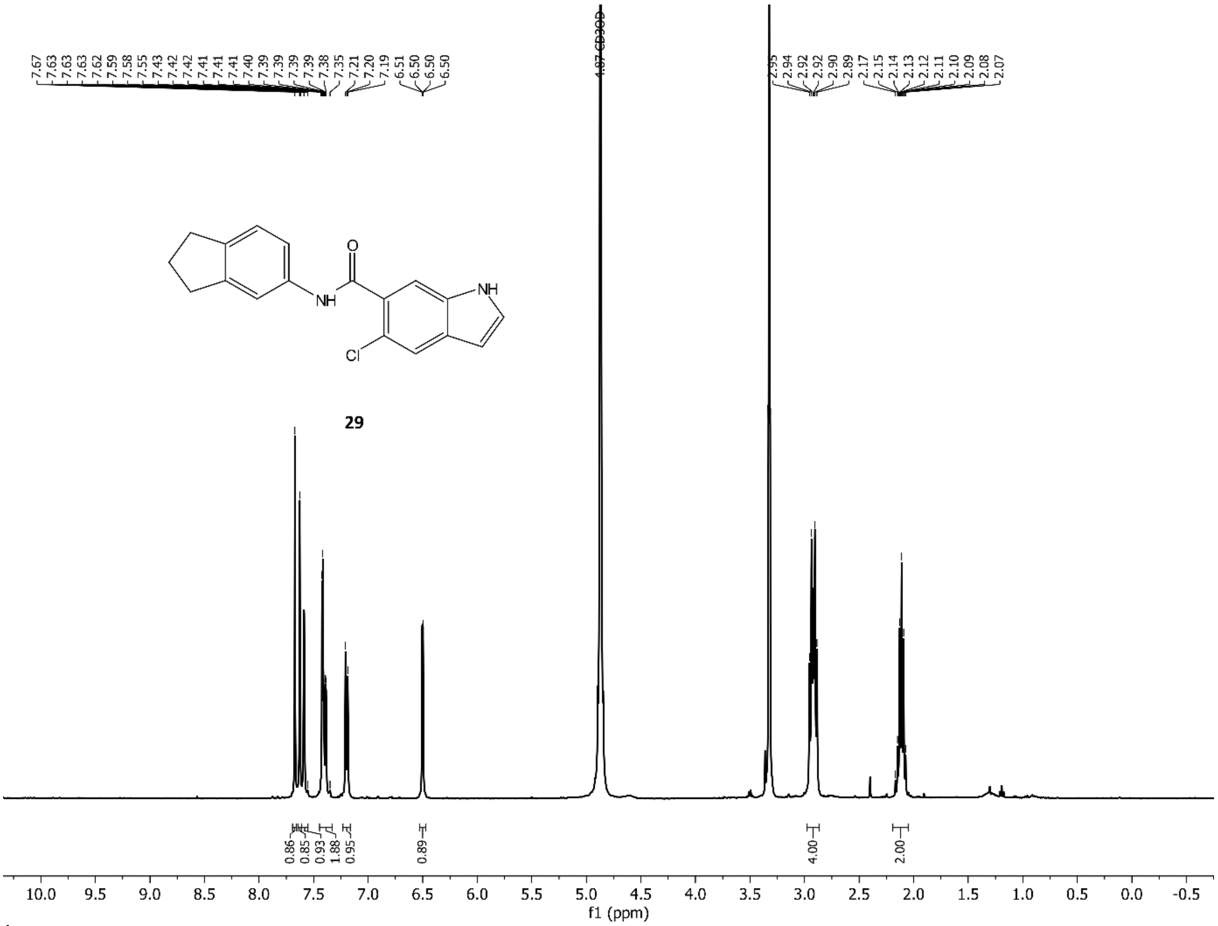

^{1}H-NMR (400 MHz, MeOD) of compound **29**.

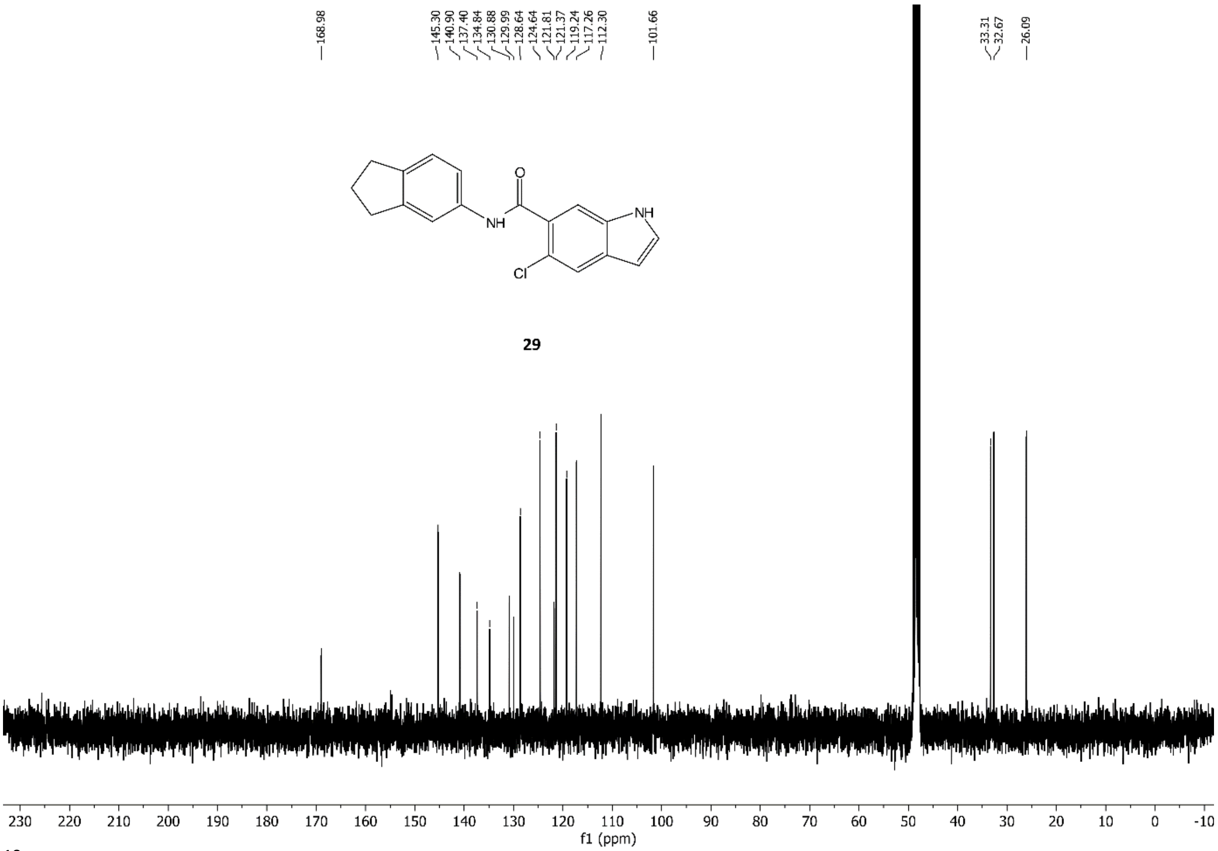

^{13}C-NMR (101 MHz, MeOD) of compound **29**.

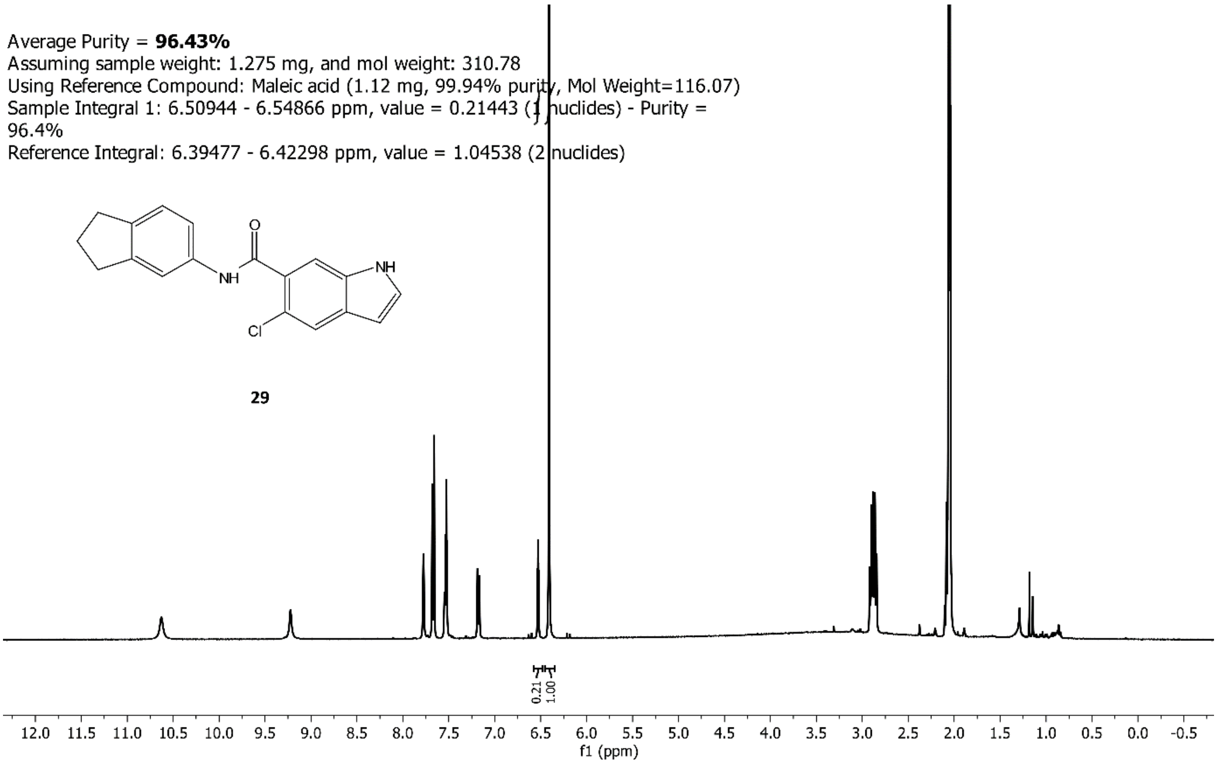

qHNMR (400 MHz, acetone-d_6, maleic acid as reference) of compound **29**.

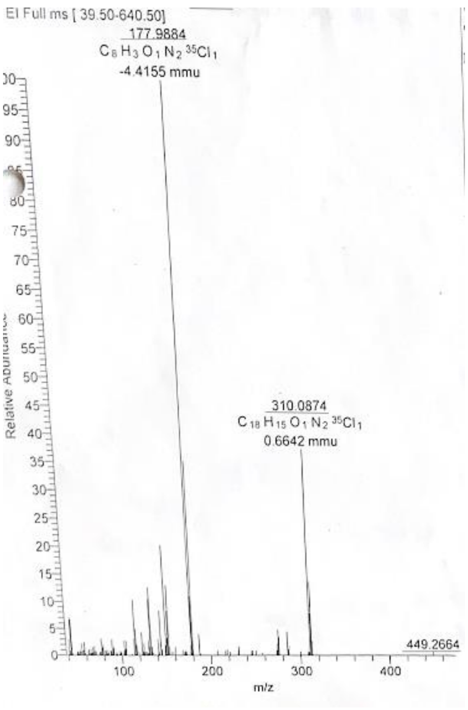

HRMS (EI+) of compound **29**.

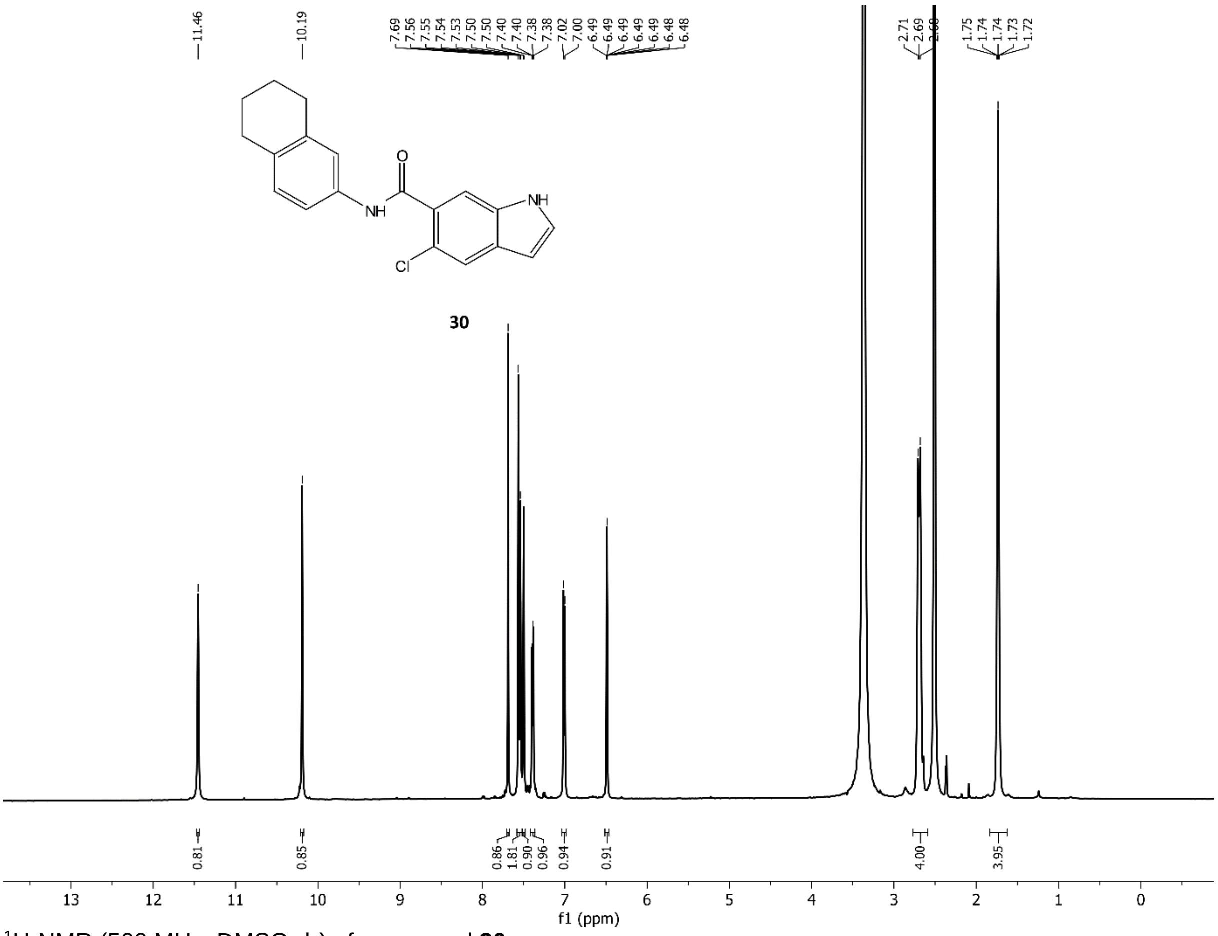

^{1}H-NMR (500 MHz, DMSO-d_6) of compound **30**.

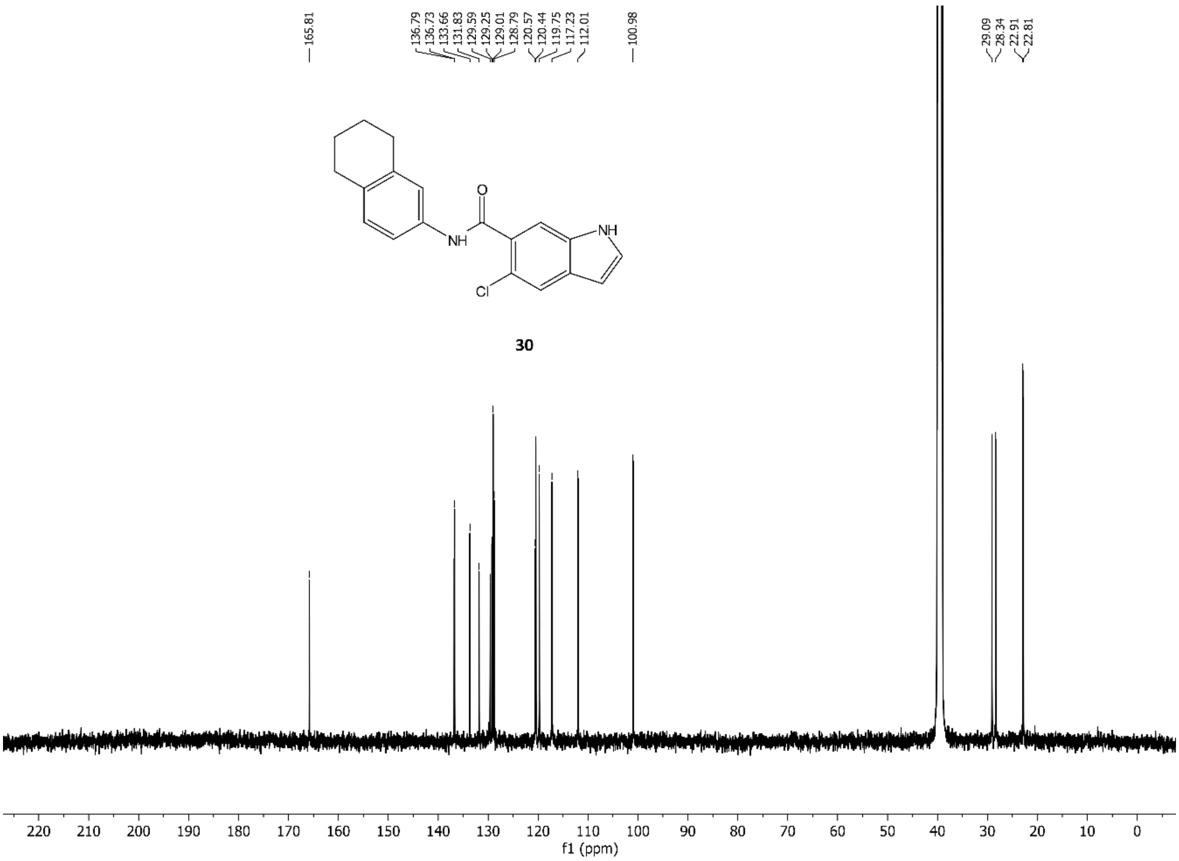

^{13}C-NMR (126 MHz, DMSO-d_6) of compound **30**.

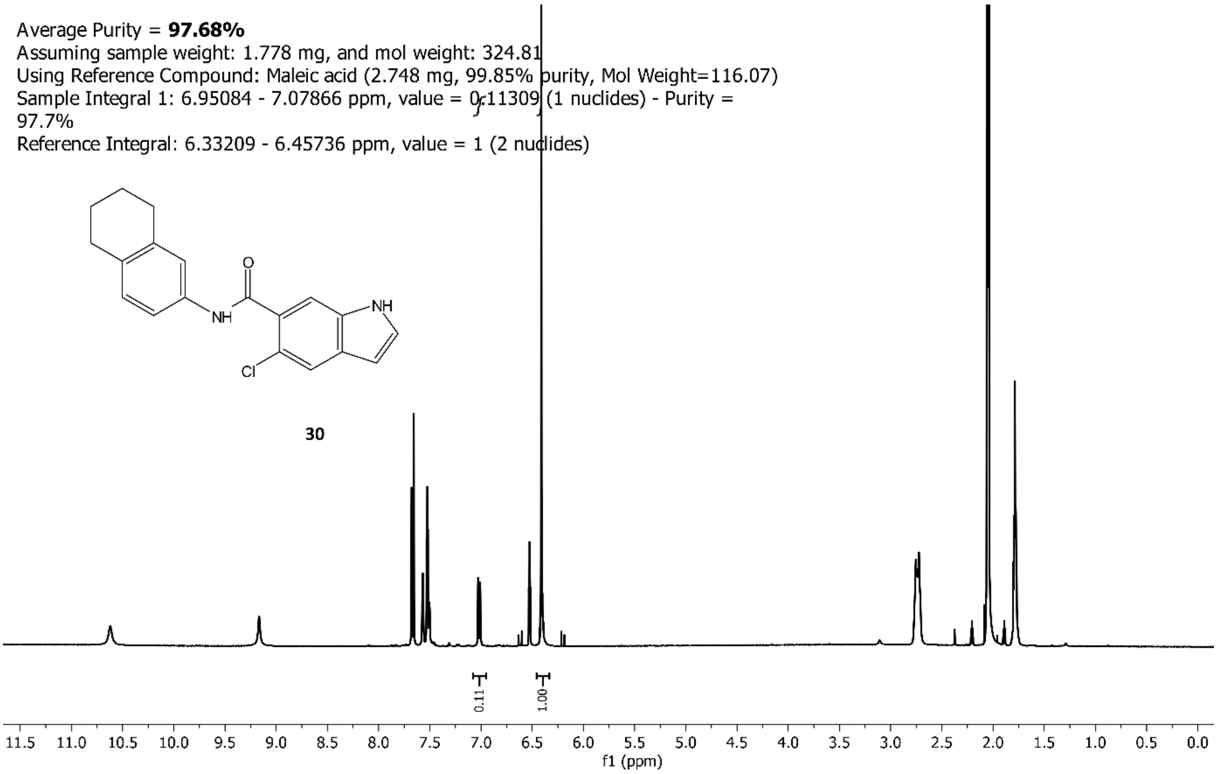

qHNMR (400 MHz, acetone-d$_6$, maleic acid as reference) of compound **30**.

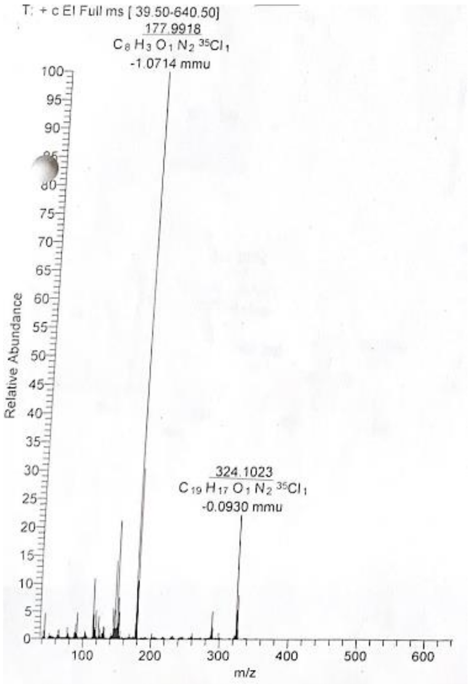

HRMS (EI+) of compound **30**.

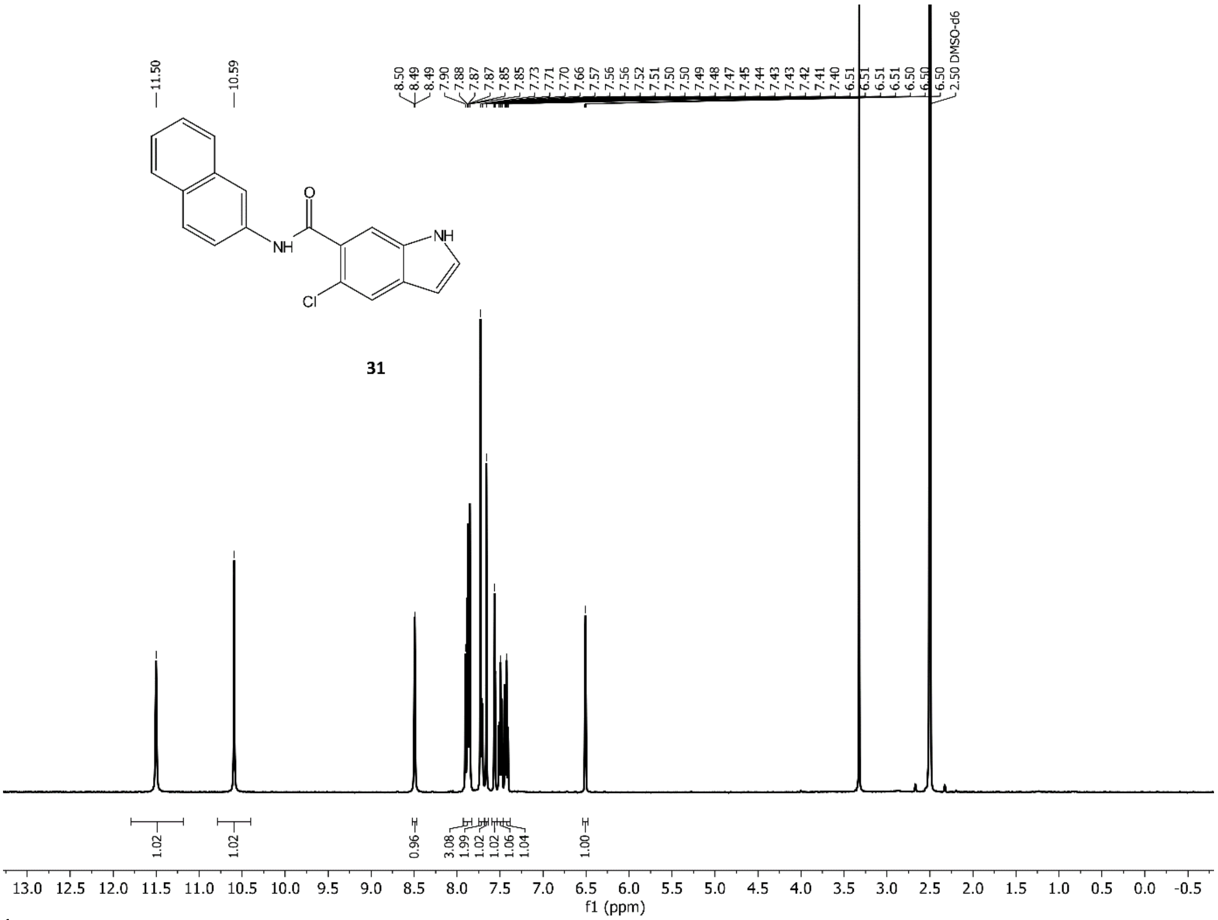

^{1}H-NMR (400 MHz, DMSO-d$_6$) of compound **31**.

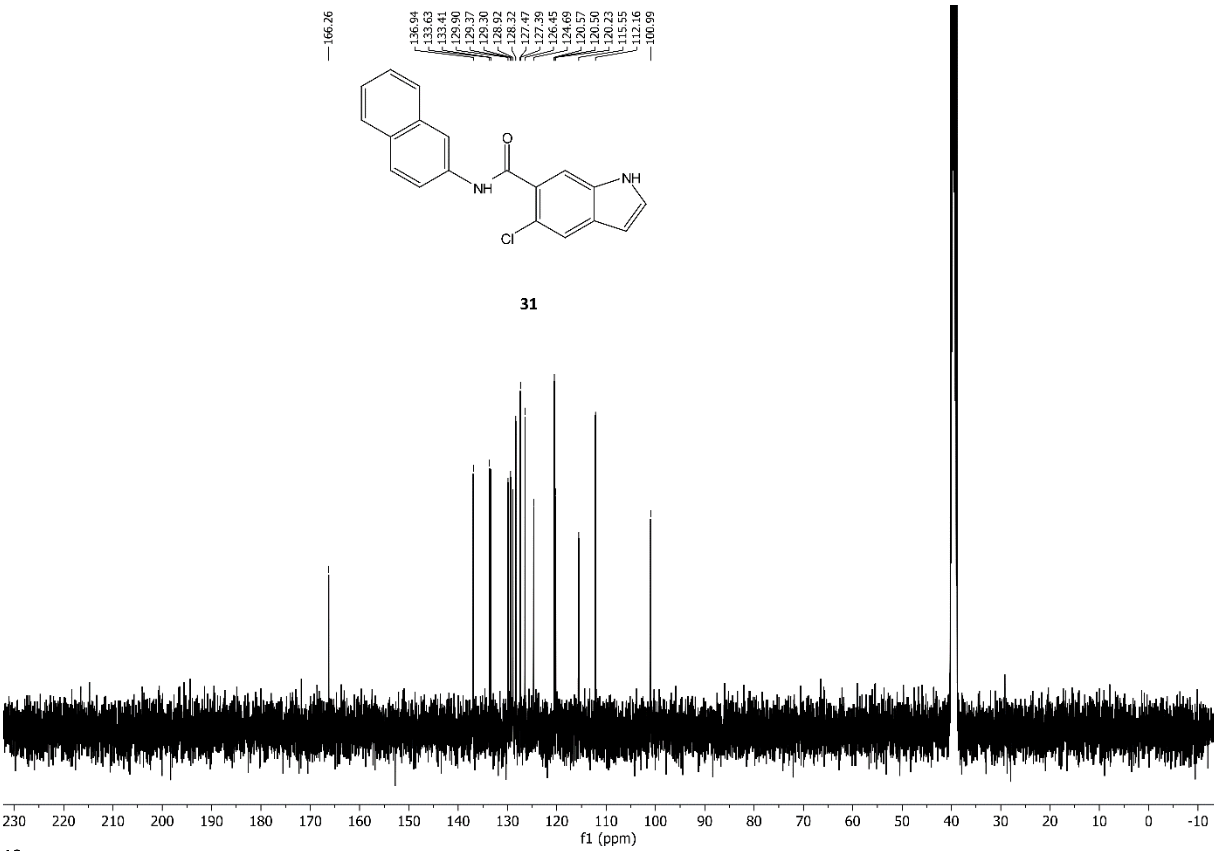

^{13}C-NMR (101 MHz, DMSO-d$_6$) of compound **31**.

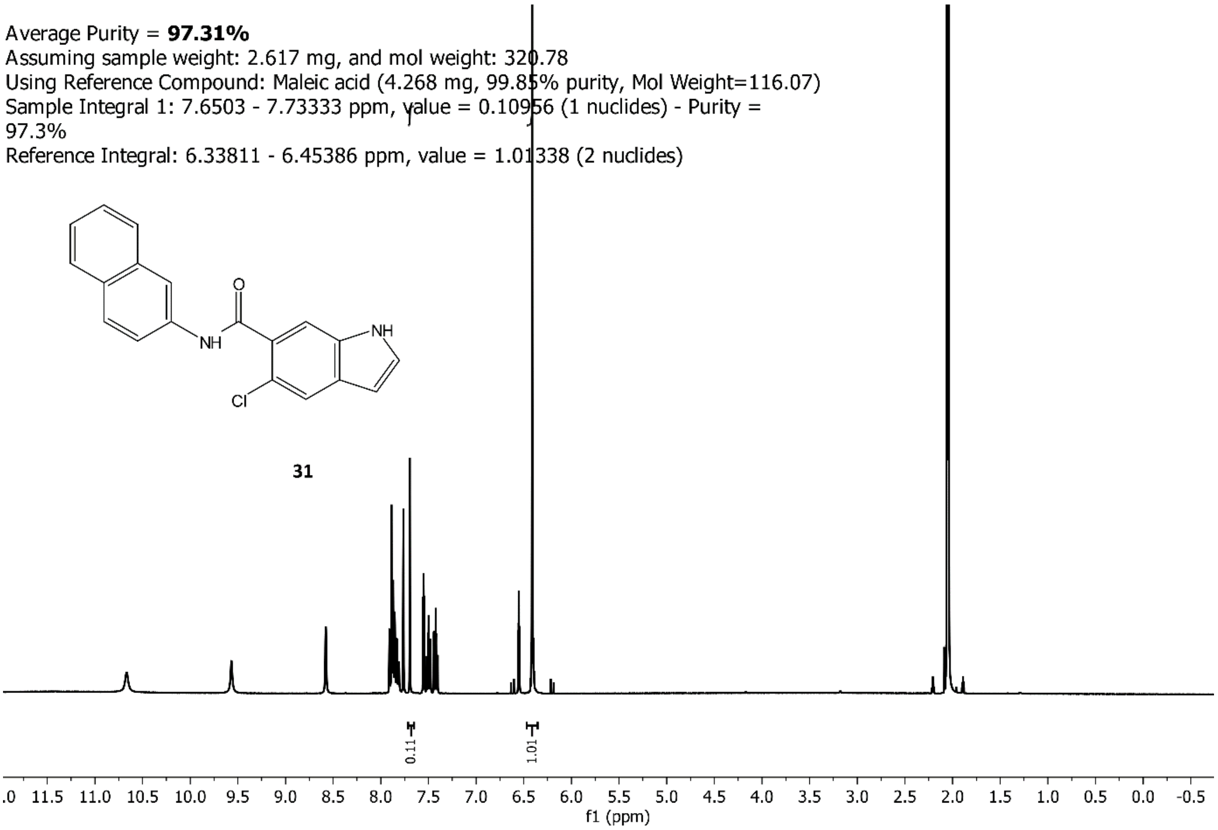

qHNMR (400 MHz, acetone-d_6, maleic acid as reference) of compound **31**.

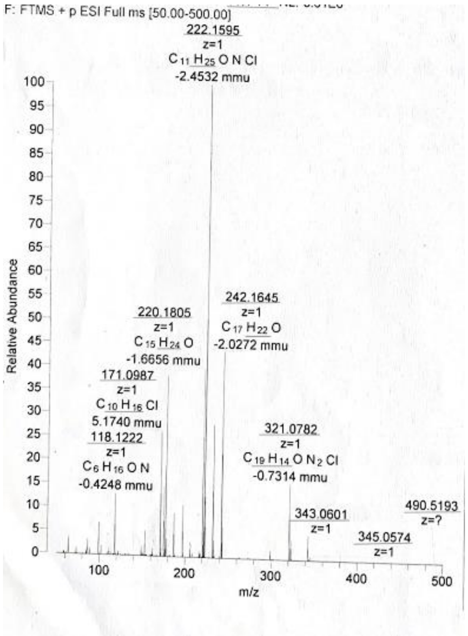

HRMS (EI+) of compound **31**.

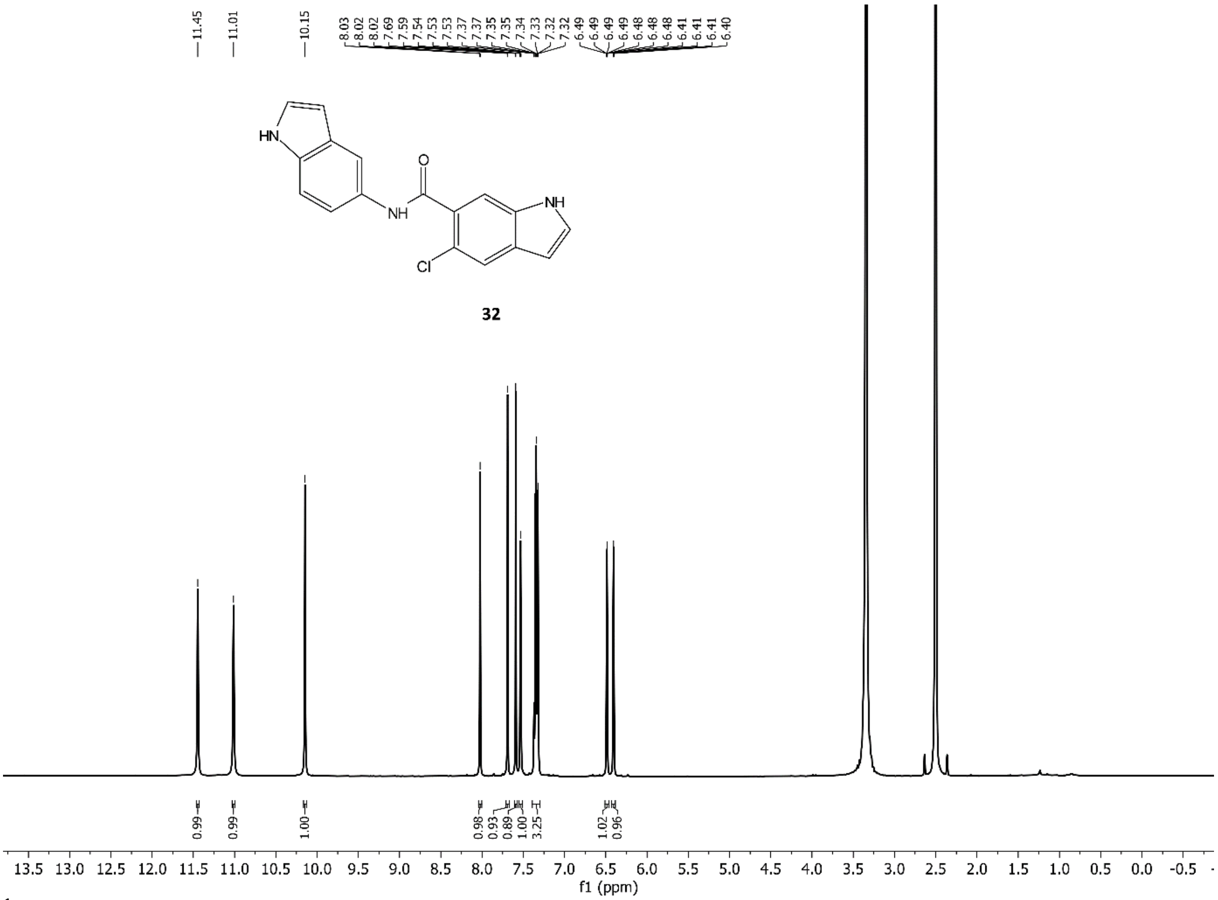

^{1}H-NMR (500 MHz, DMSO-d_6) of compound **32**.

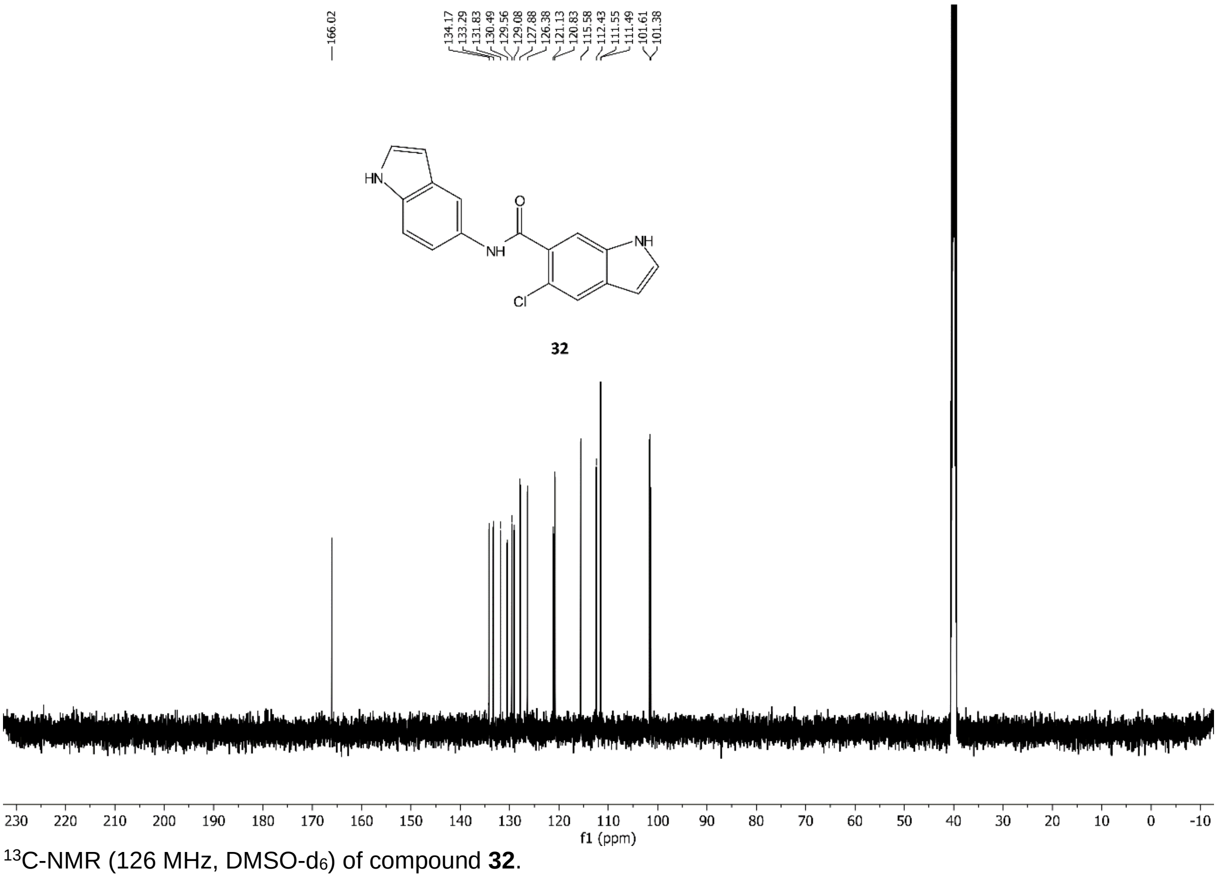

^{13}C-NMR (126 MHz, DMSO-d_6) of compound **32**.

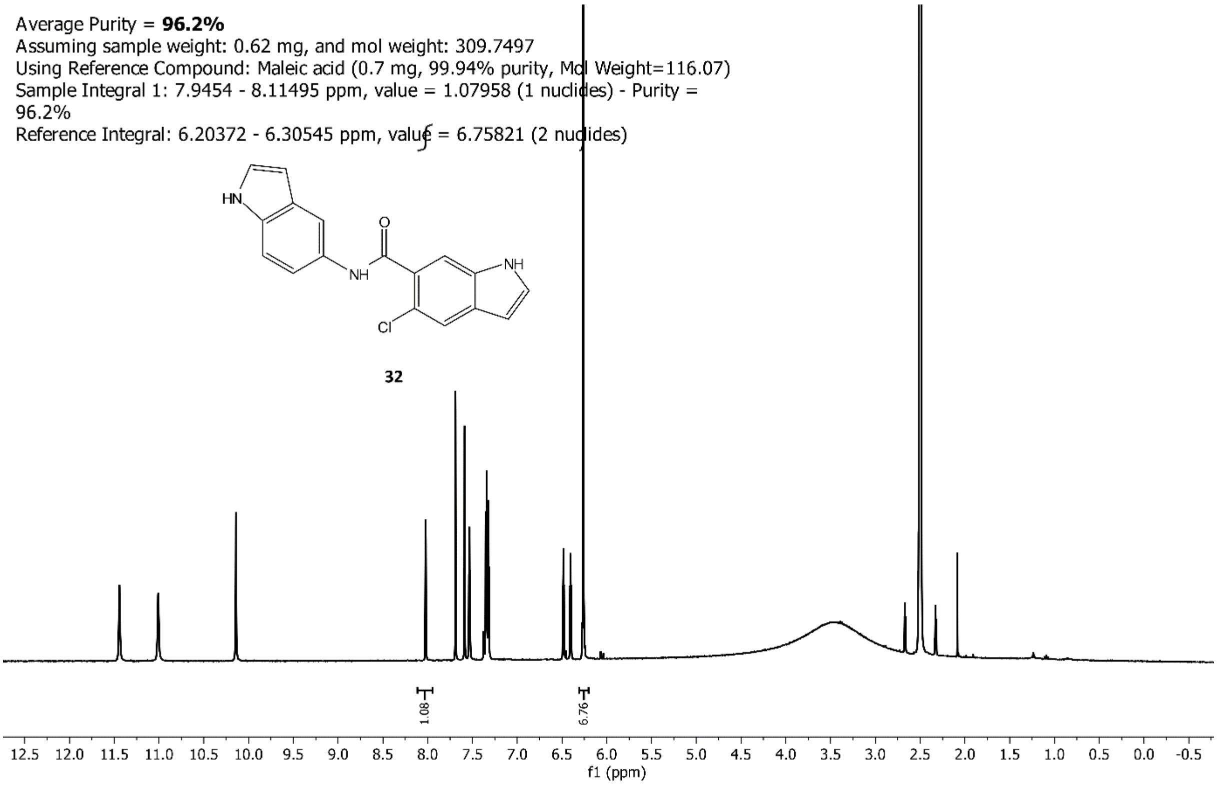

qHNMR (400 MHz, DMSO-d$_6$, maleic acid as reference) of compound **32**.

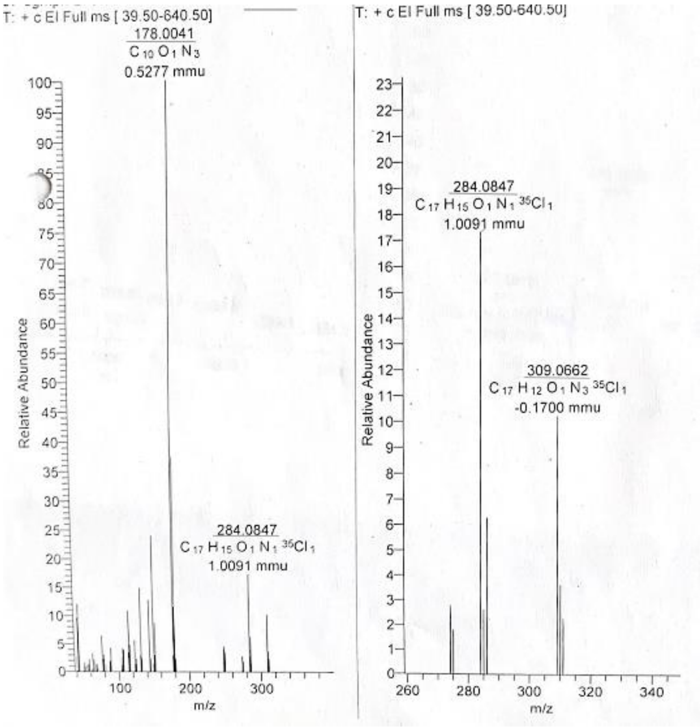

HRMS (EI+) of compound **32**. Left panel: Full spectrum. Right panel: Zoom in.

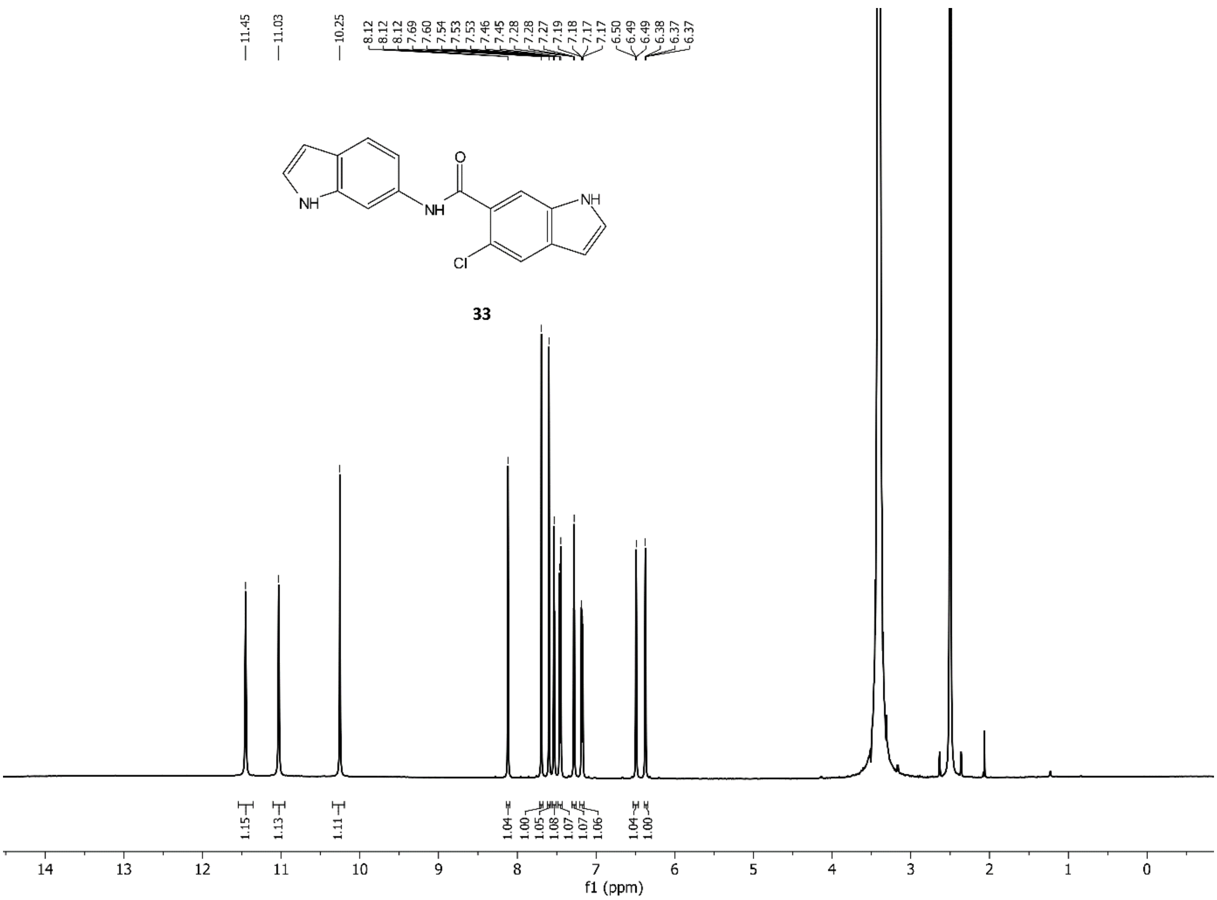

^{1}H-NMR (500 MHz, DMSO-d6) of compound **33**.

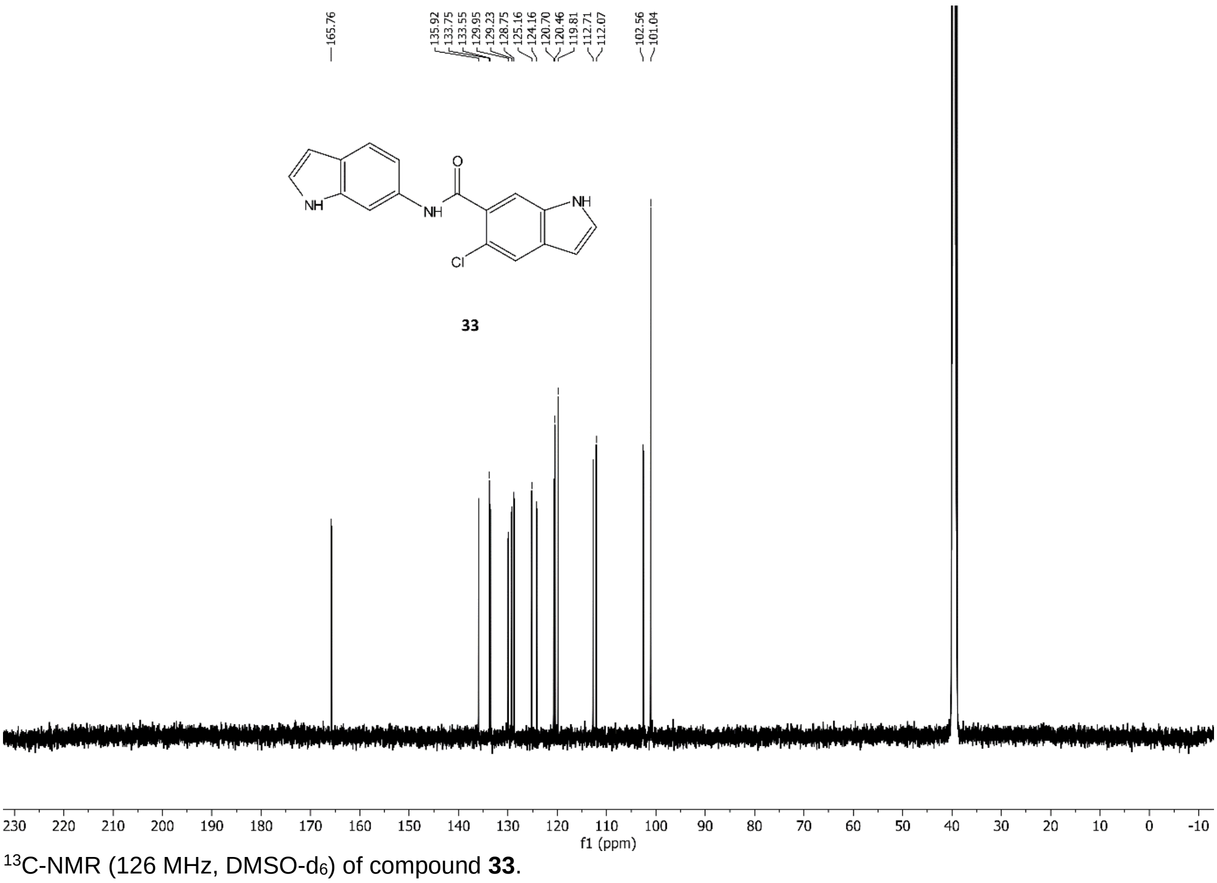

^{13}C-NMR (126 MHz, DMSO-d6) of compound **33**.

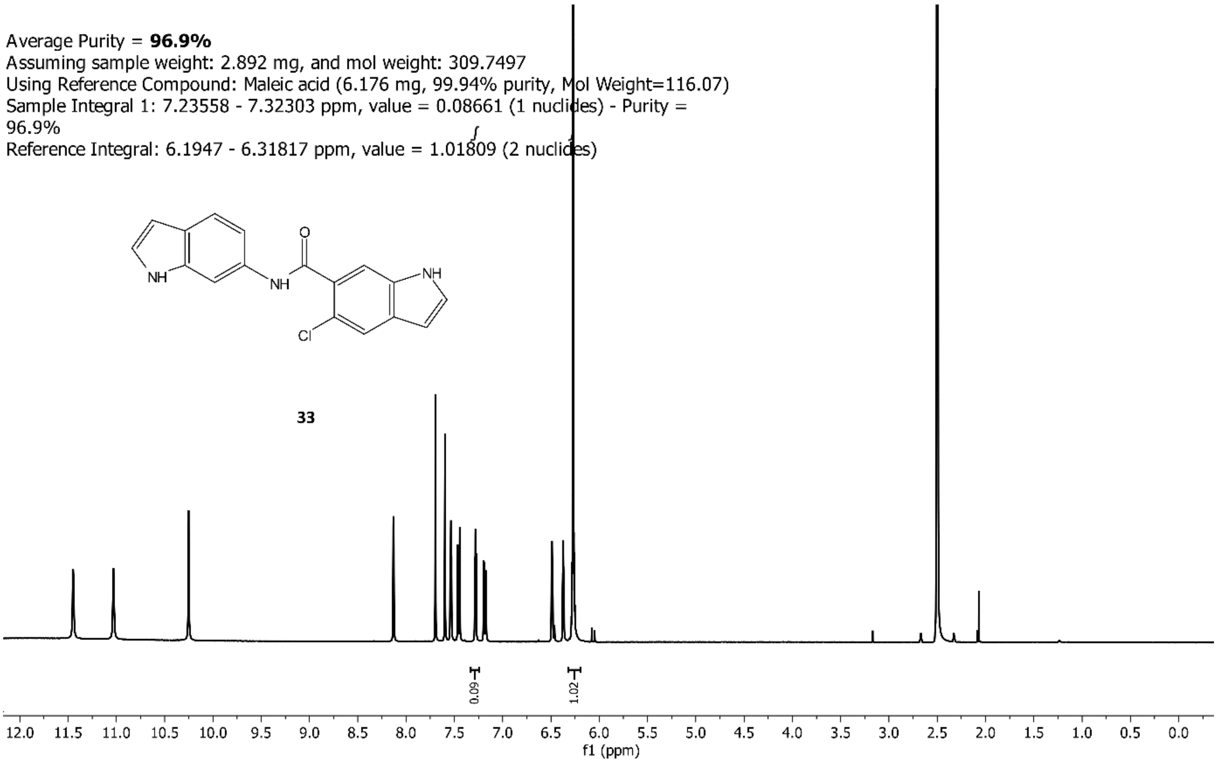

qHNMR (400 MHz, DMSO-d_6, maleic acid as reference) of compound **33.**

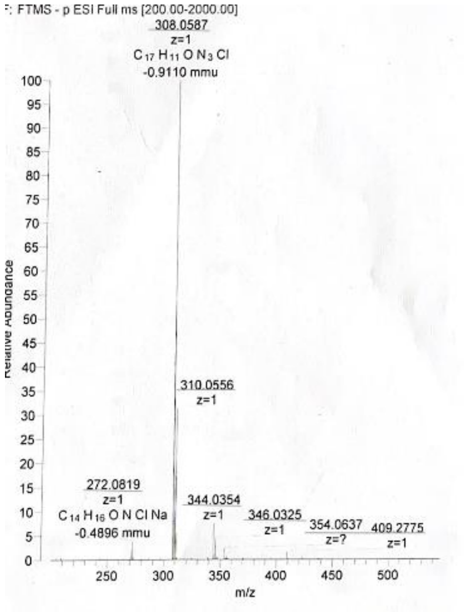

HRMS (ESI-) of compound **33**.

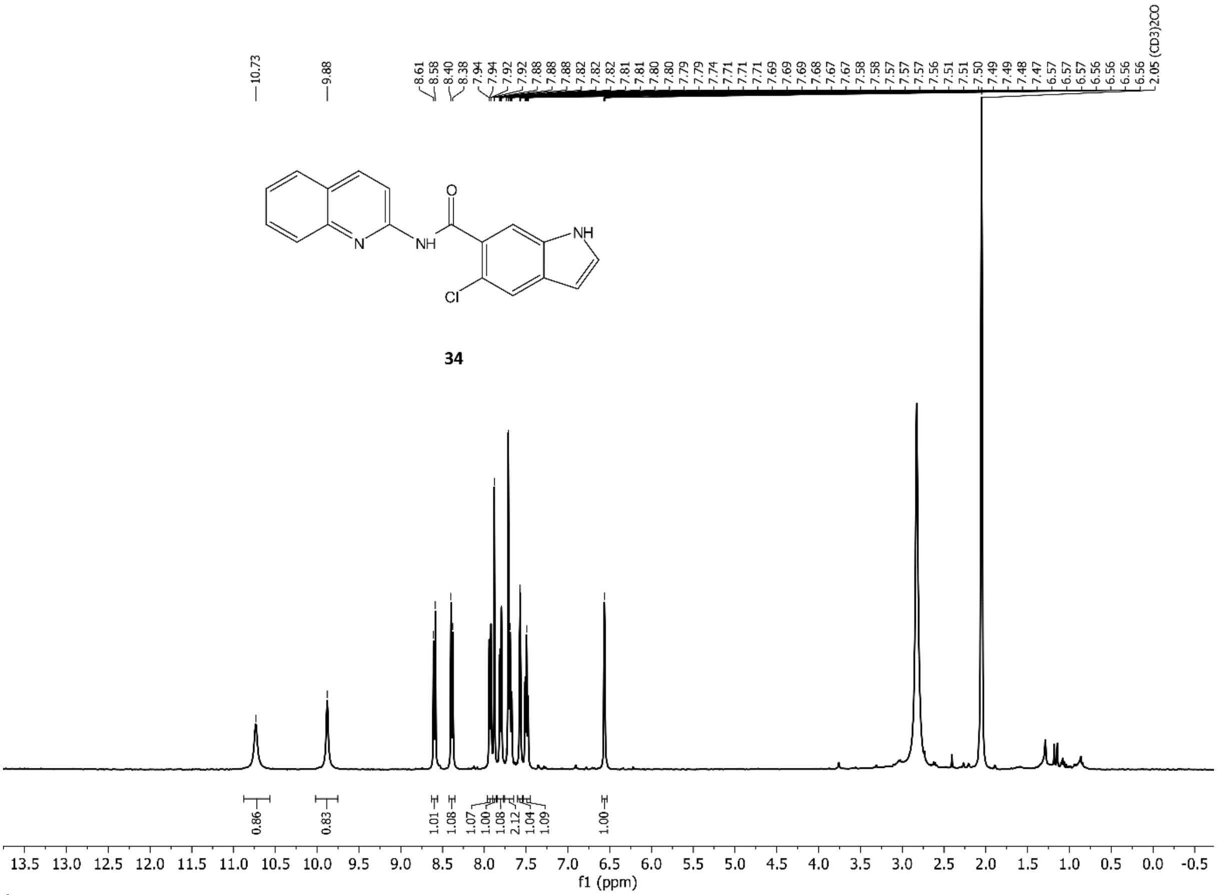

^{1}H-NMR (400 MHz, acetone-d$_6$) of compound **34.**

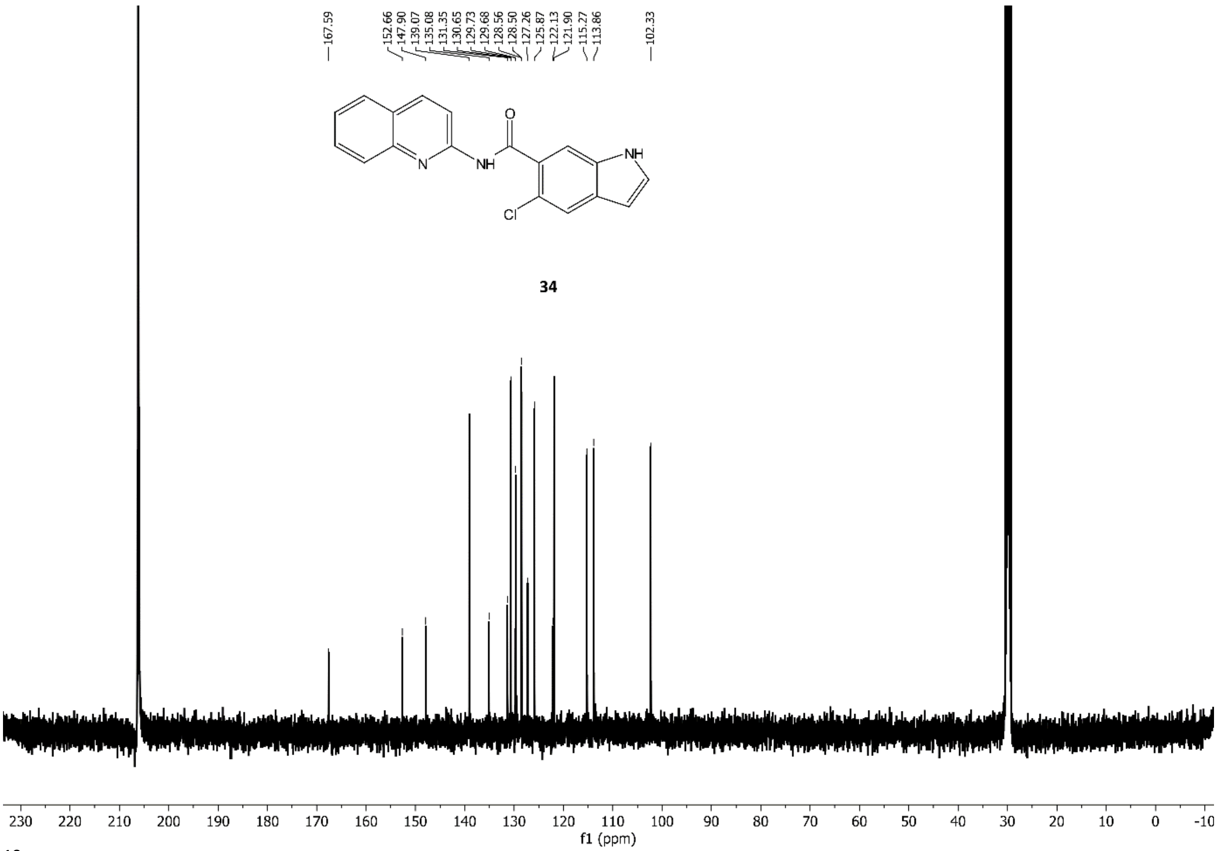

^{13}C-NMR (101 MHz, acetone-d$_6$) of compound **34.**

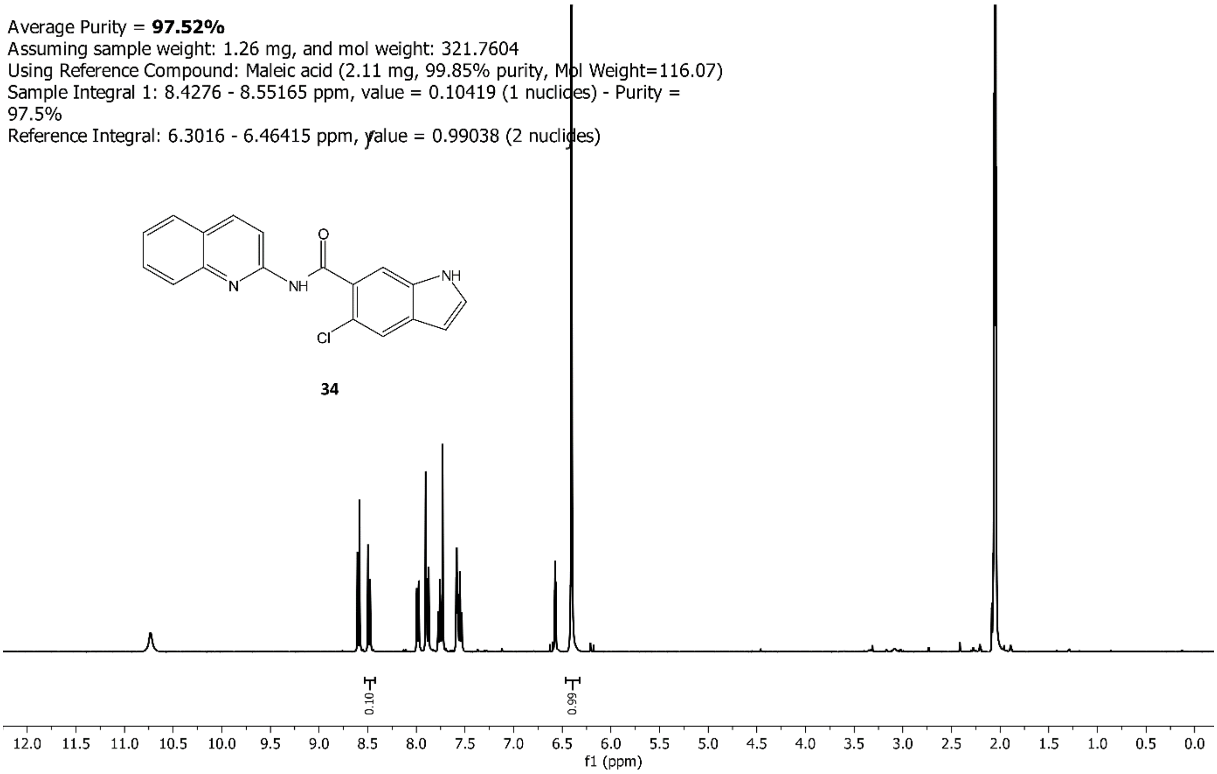

qHNMR (400 MHz, acetone-d$_6$, maleic acid as reference) of compound **34**.

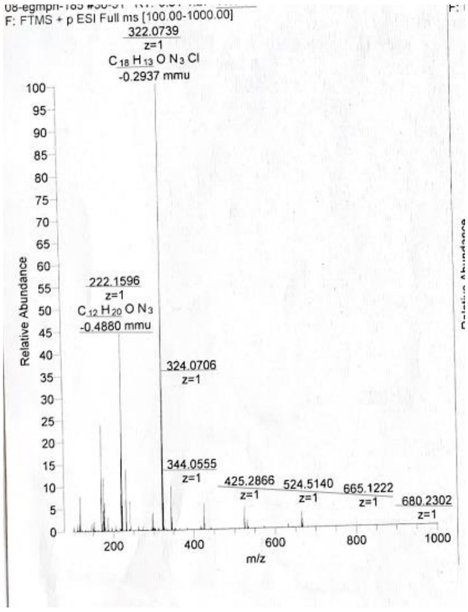

HRMS (ESI+) of compound **34**.

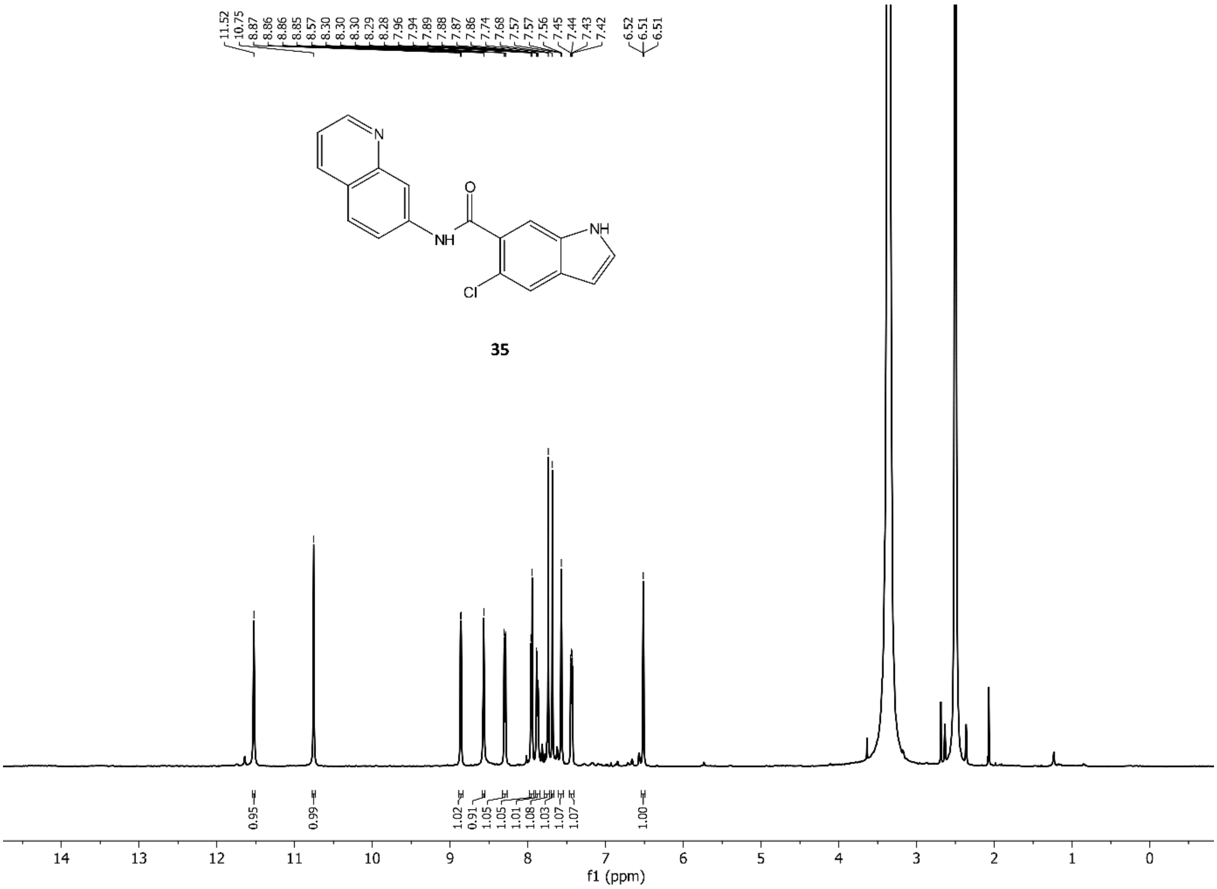

^{1}H-NMR (500 MHz, DMSO-d6) of compound **35**.

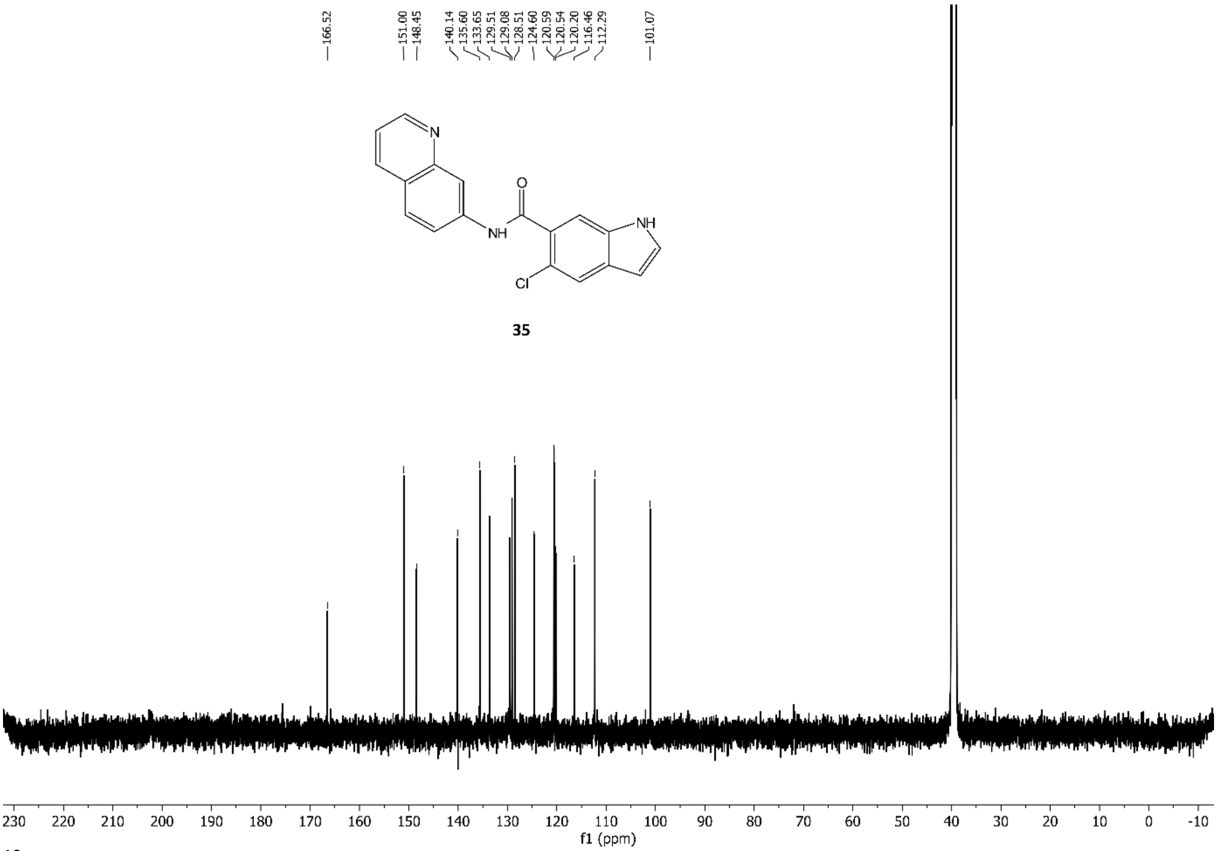

^{13}C-NMR (126 MHz, DMSO-d6) of compound **35**.

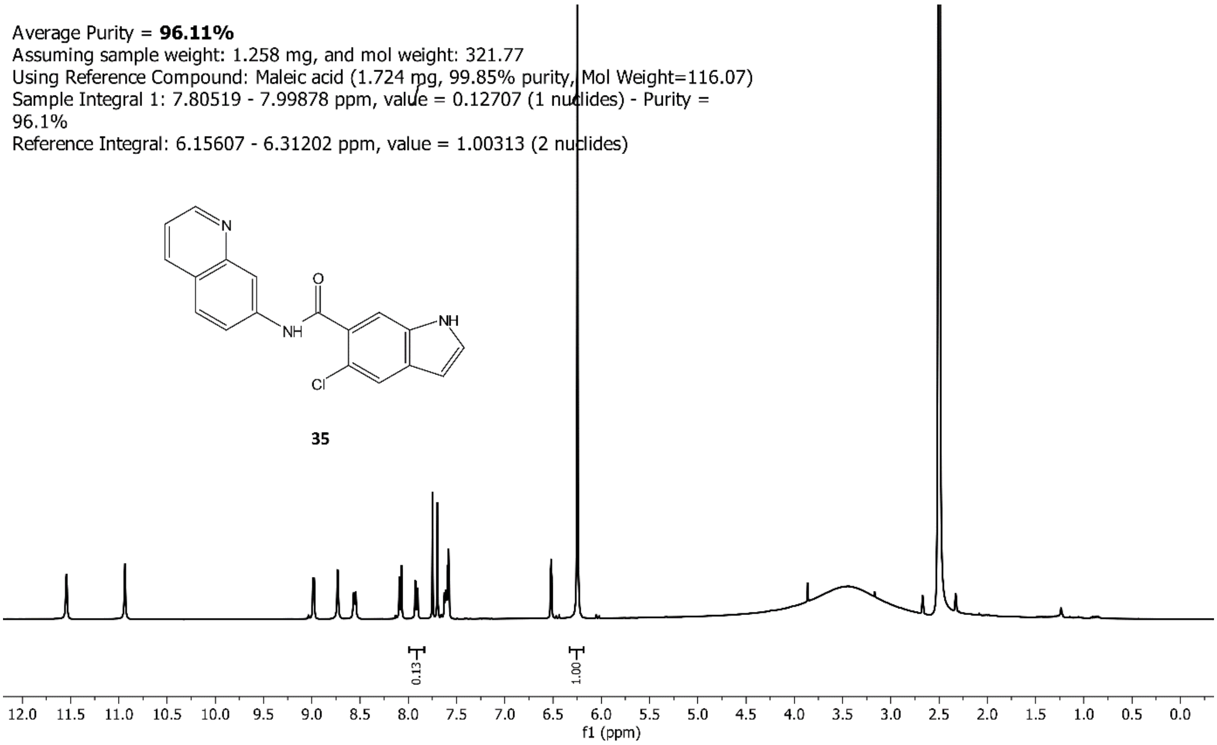

qHNMR (400 MHz, DMSO-d$_6$, maleic acid as reference) of compound **35**.

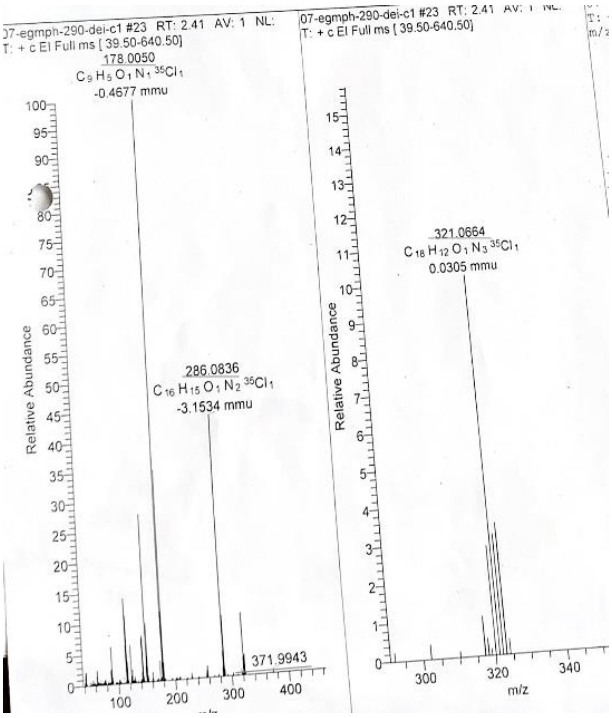

HRMS (EI+) of compound **35**. Left panel: Full spectrum. Right panel: Zoom in.

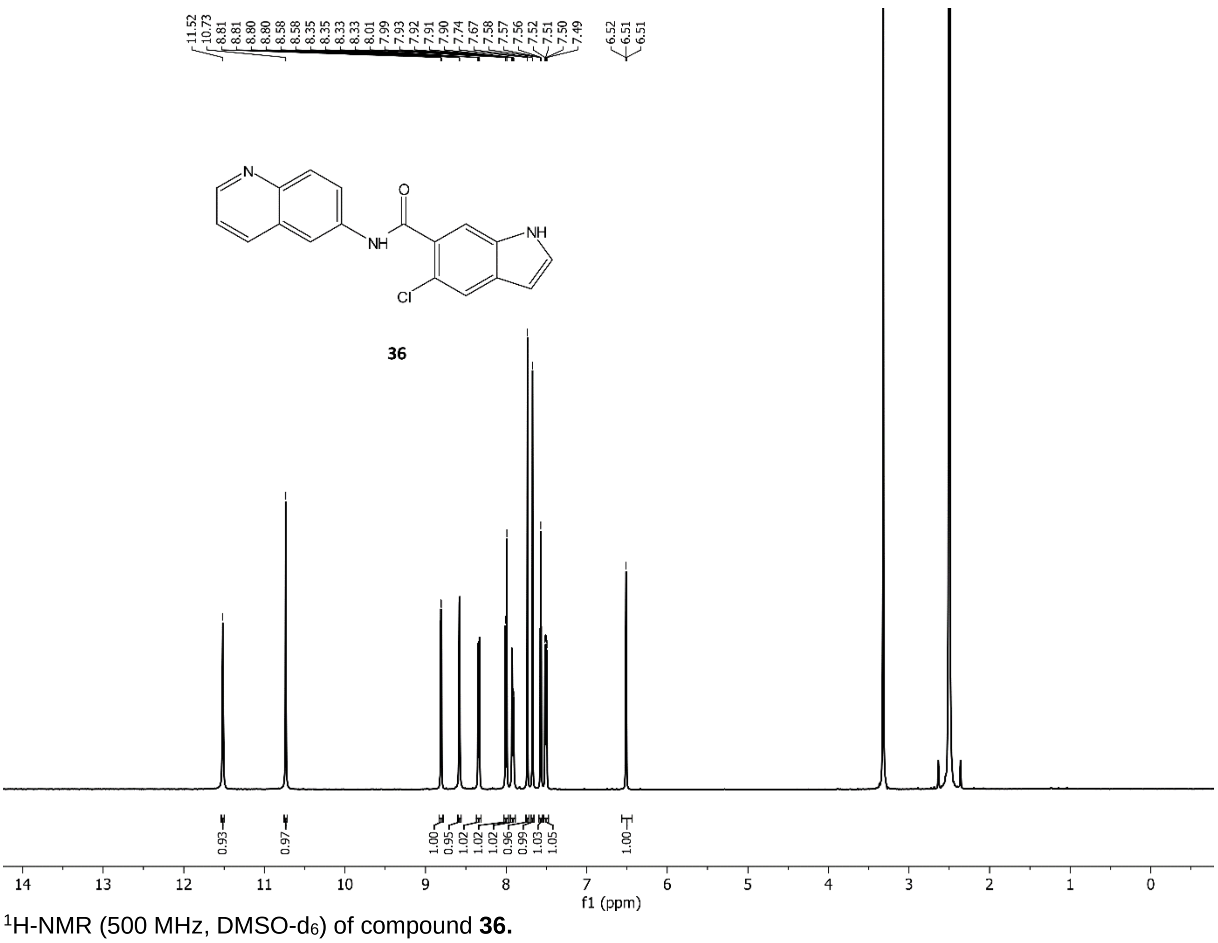

^{1}H-NMR (500 MHz, DMSO-d$_6$) of compound **36.**

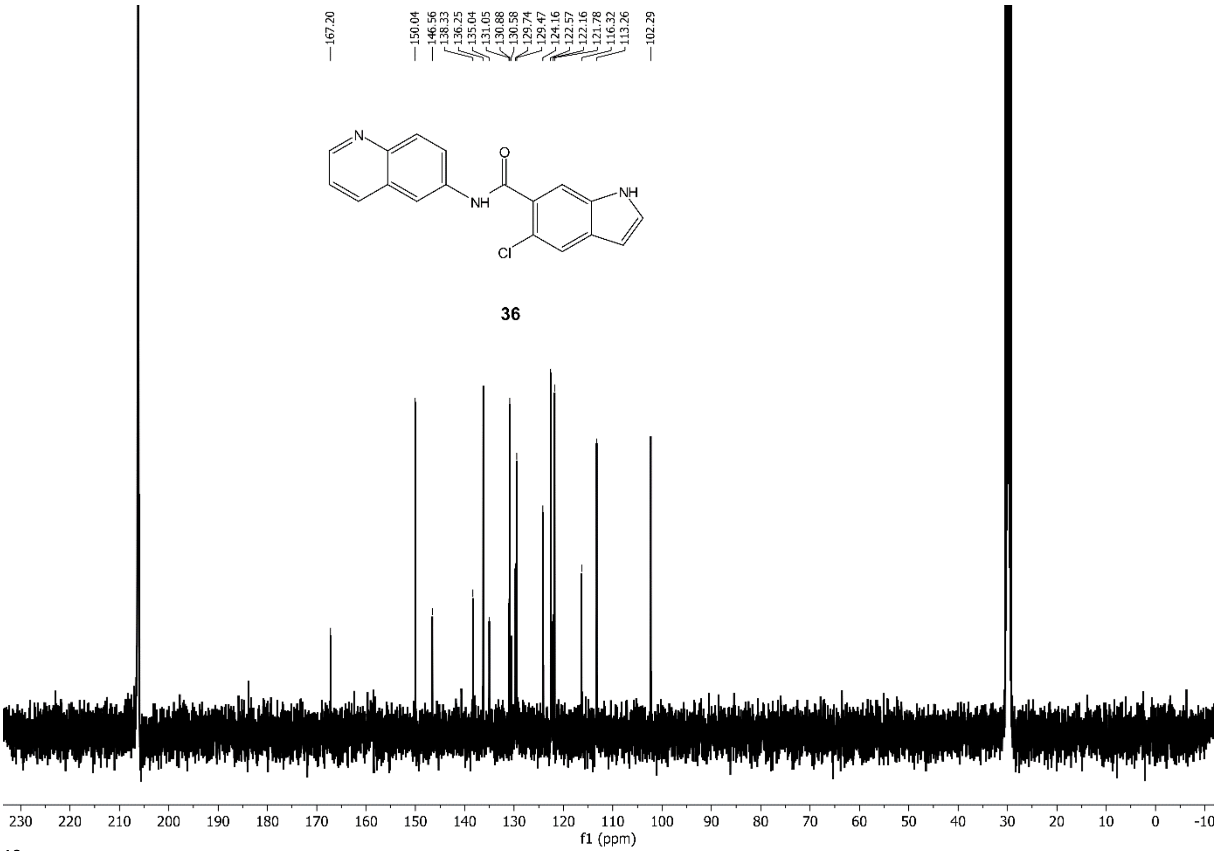

^{13}C-NMR (101 MHz, acetone-d$_6$) of compound **36**.

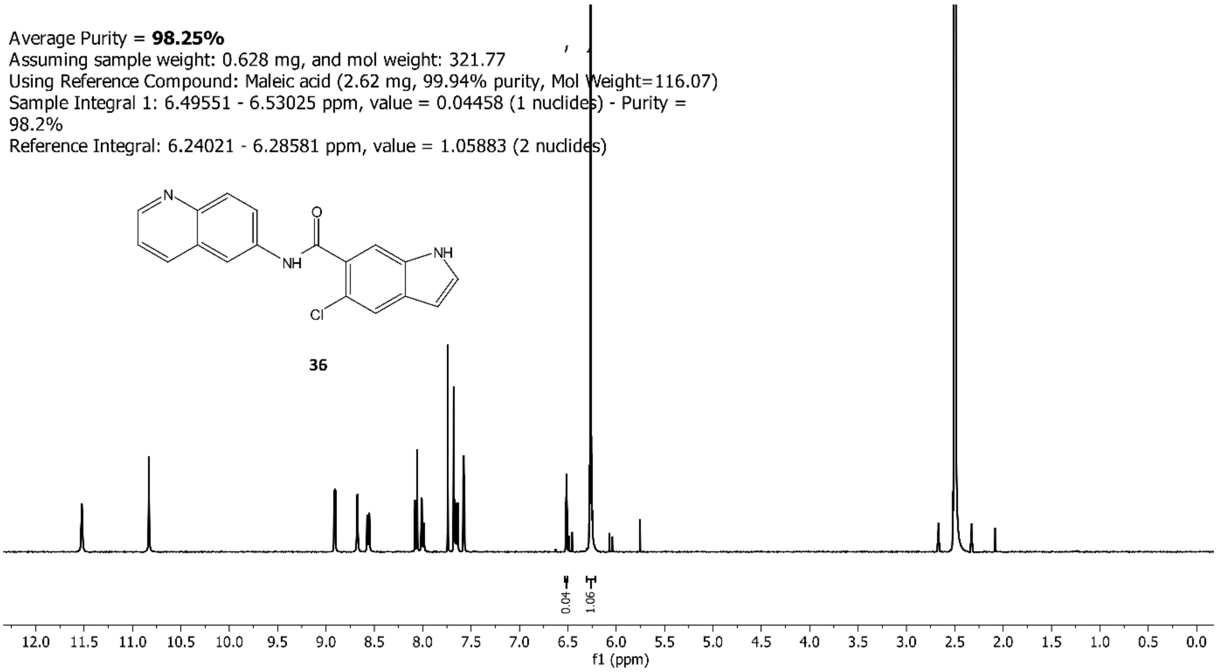

qHNMR (400 MHz, DMSO-d$_6$, maleic acid as reference) of compound **36**.

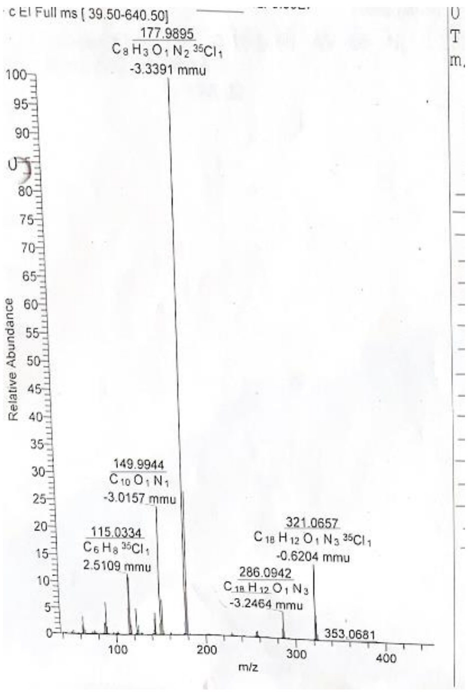

HRMS (EI+) of compound **36**.

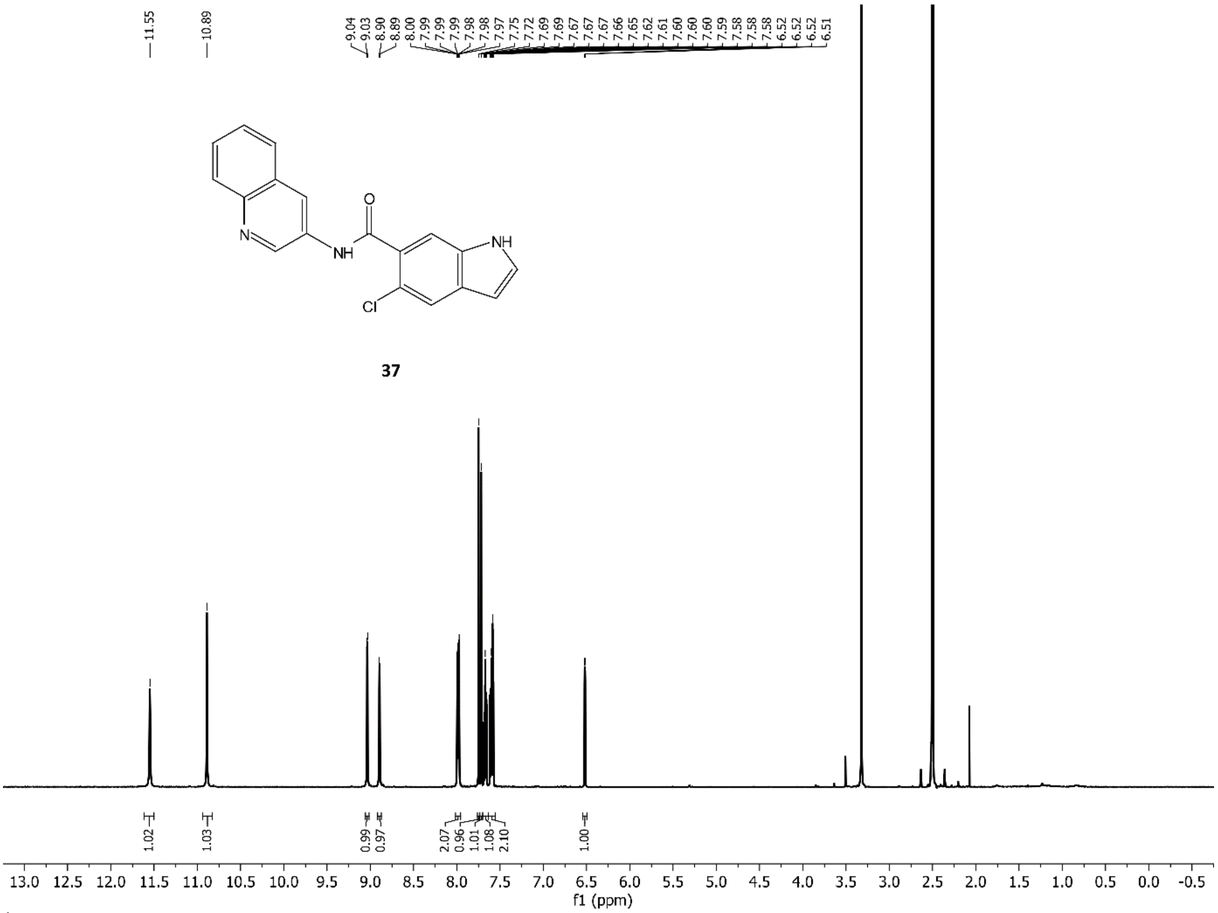

^{1}H-NMR (500 MHz, DMSO-d$_6$) of compound **37**.

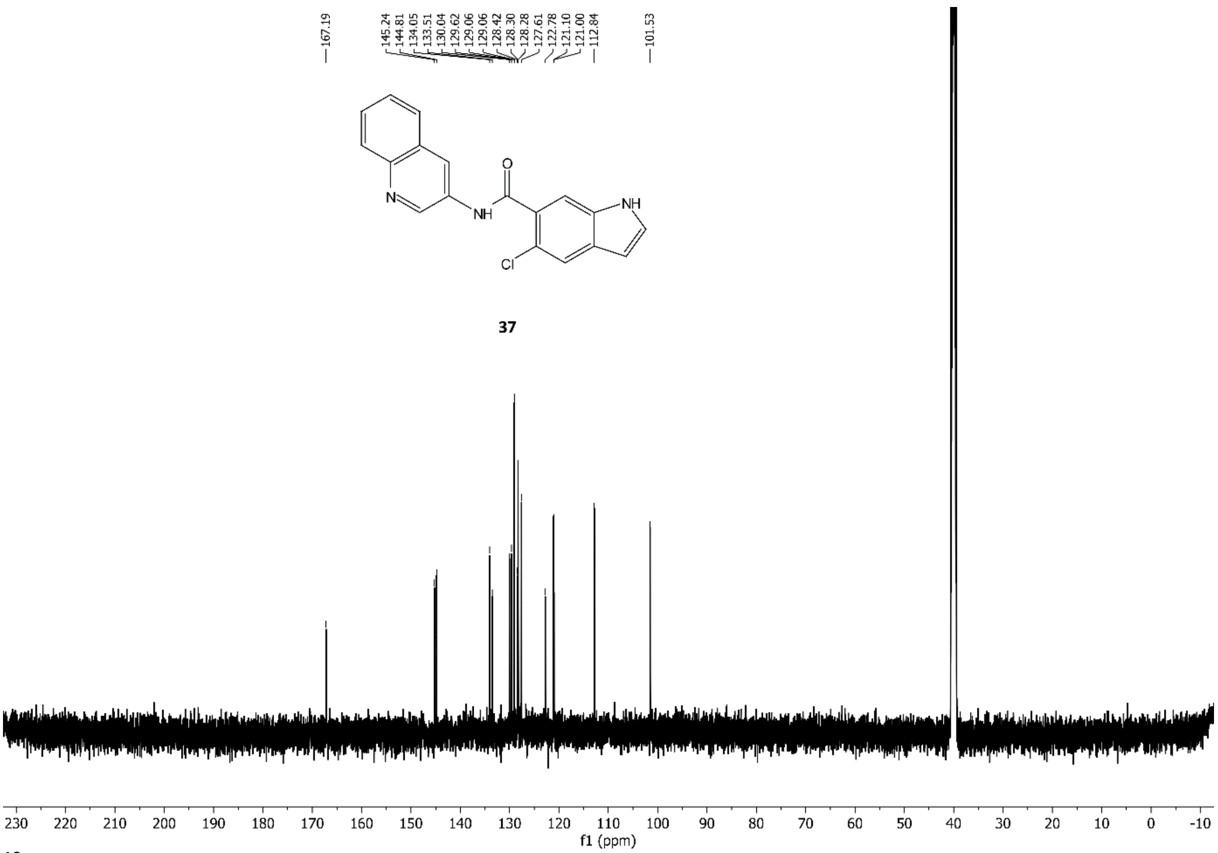

^{13}C-NMR (126 MHz, DMSO-d$_6$) of compound **37**.

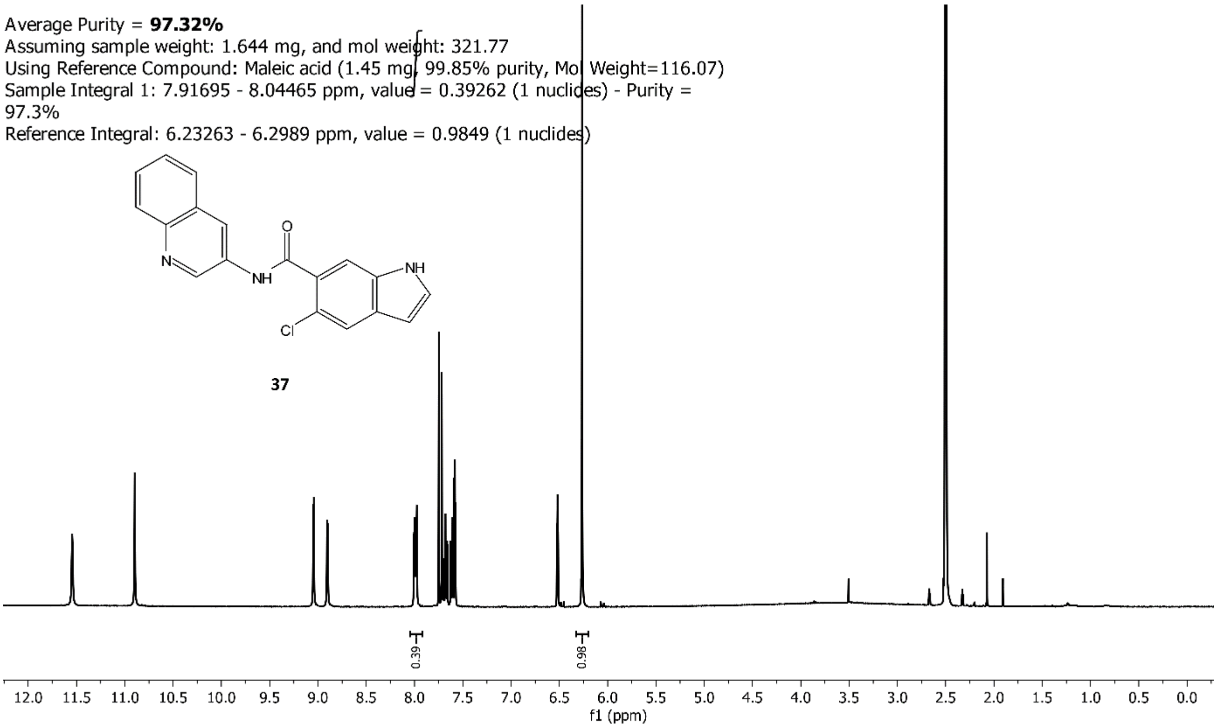

qHNMR (400 MHz, DMSO-d$_6$, maleic acid as reference) of compound **37**.

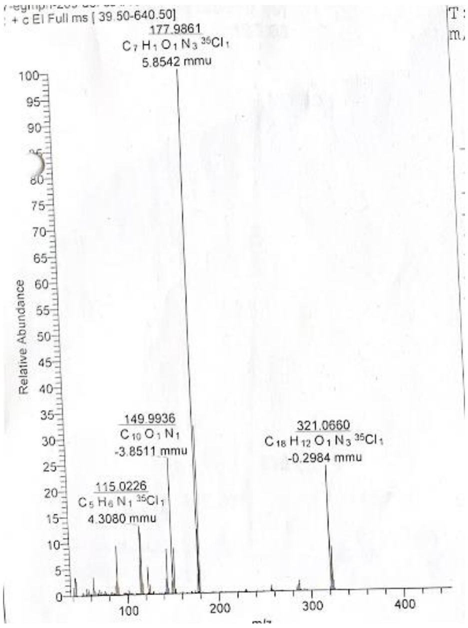

HRMS (EI+) of compound **37**.

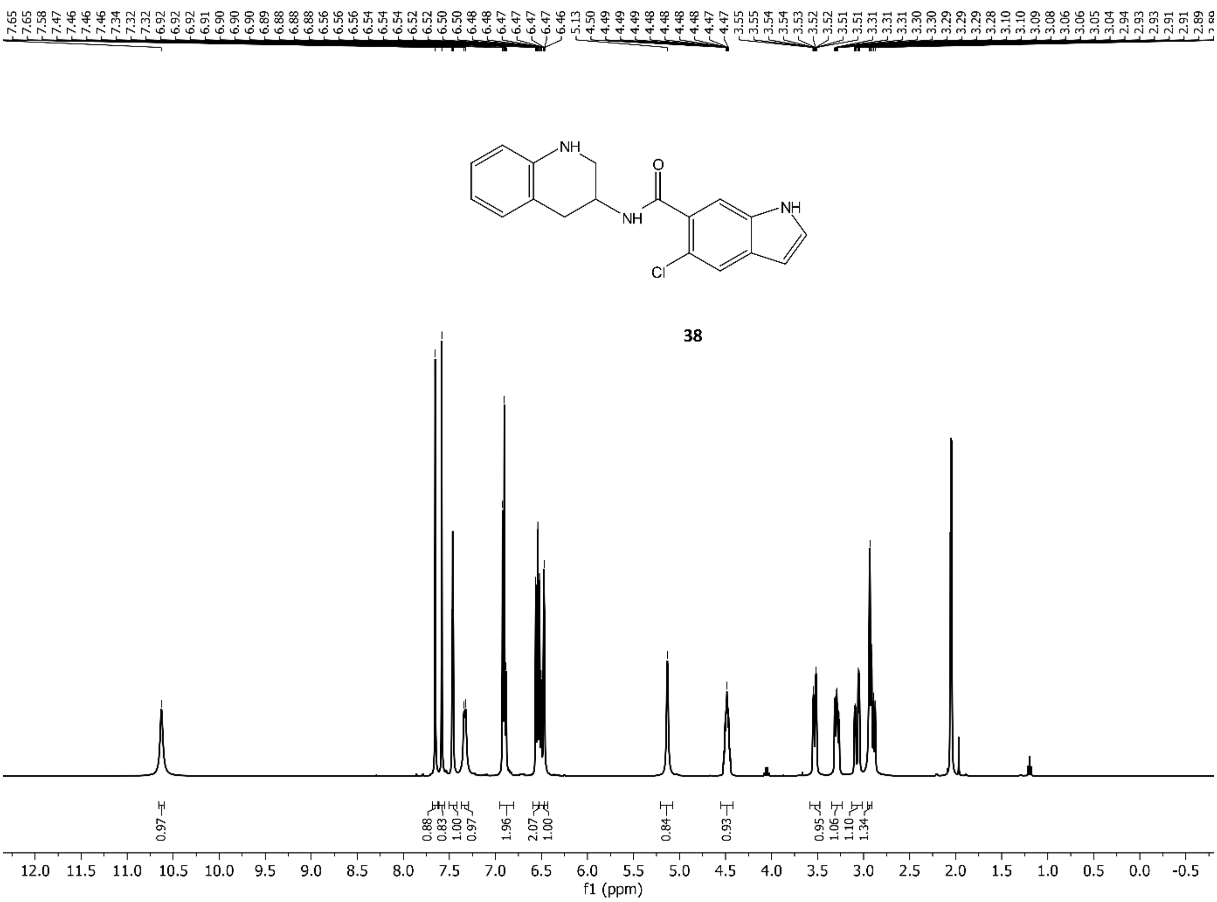

^{1}H-NMR (400 MHz, acetone-d_6) of compound **38**.

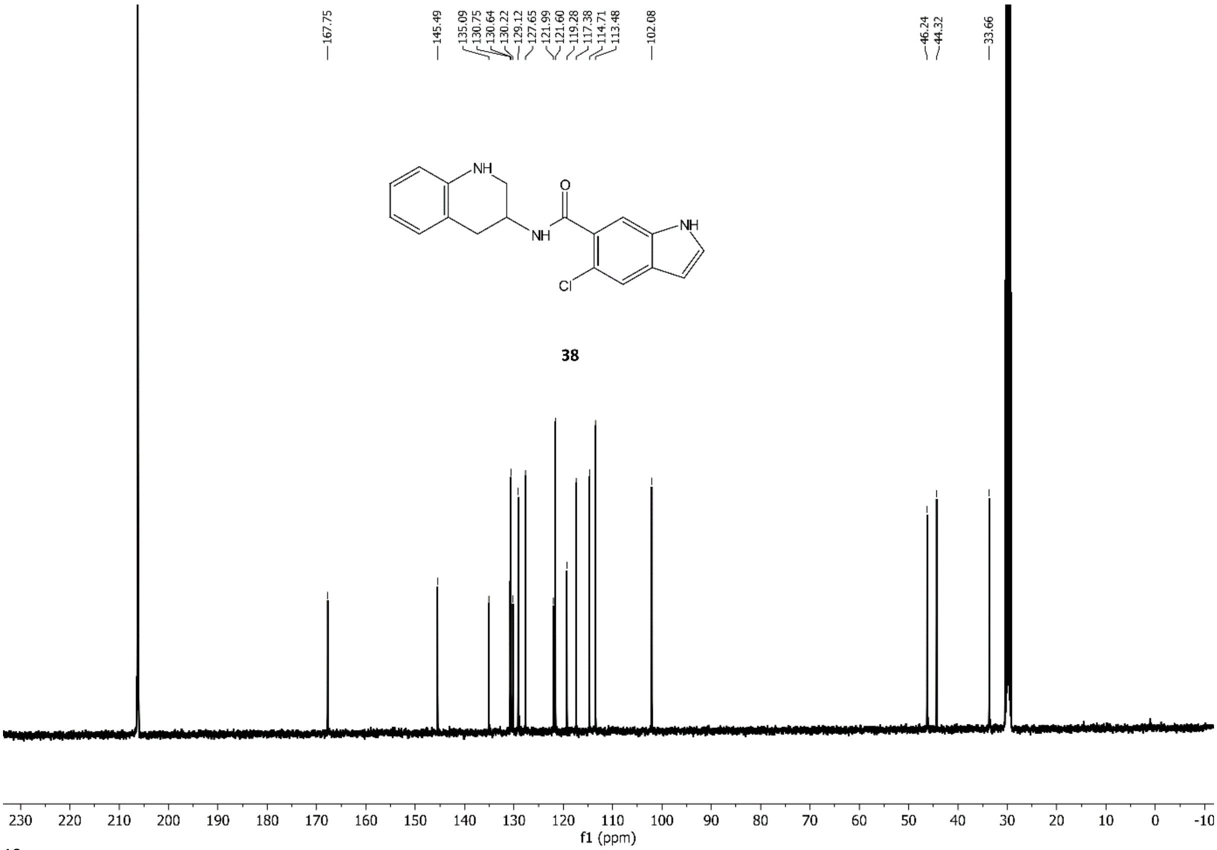

^{13}C-NMR (101 MHz, acetone-d_6) of compound **38**.

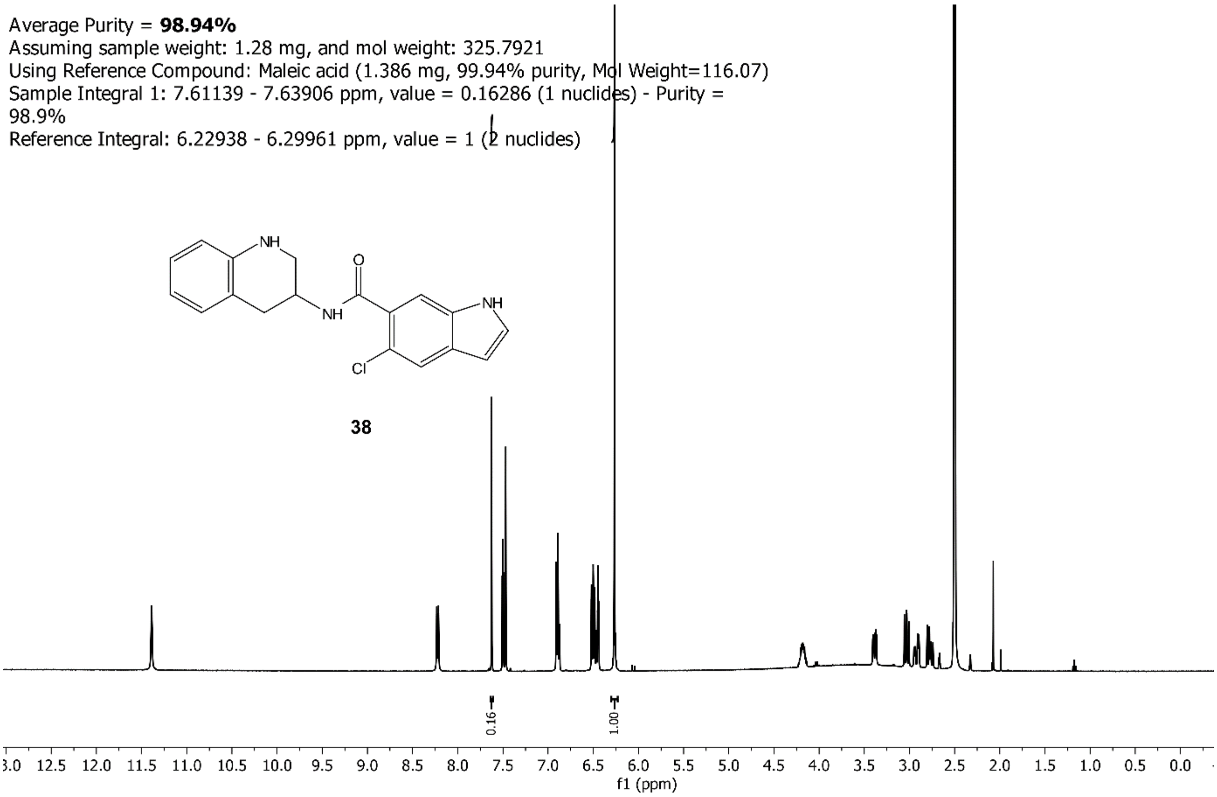

qHNMR (400 MHz, DMSO-d$_6$, maleic acid as reference) of compound **38**.

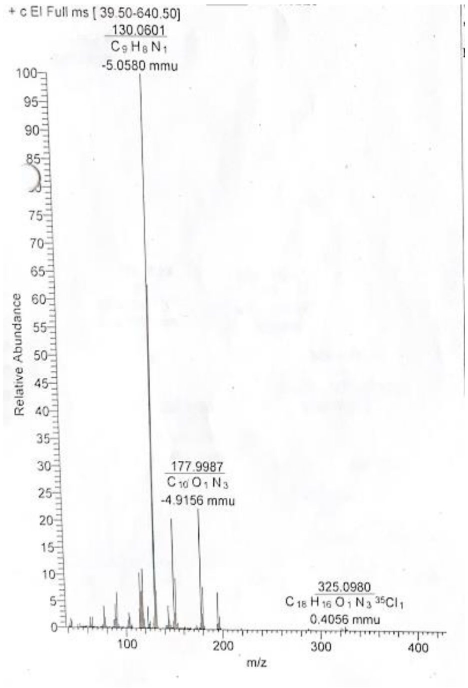

HRMS (EI+) of compound **38**.

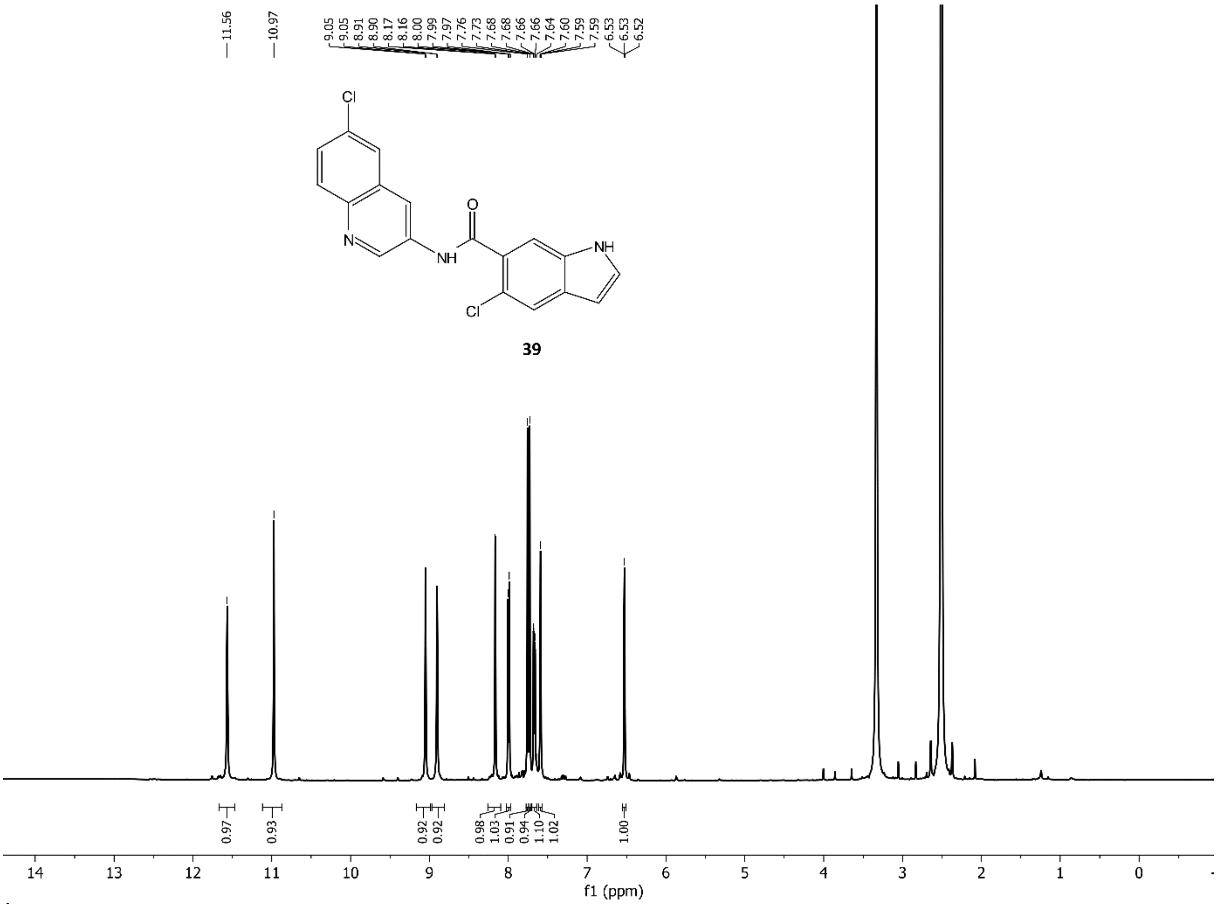

^{1}H-NMR (500 MHz, DMSO-d$_6$) of compound **39**.

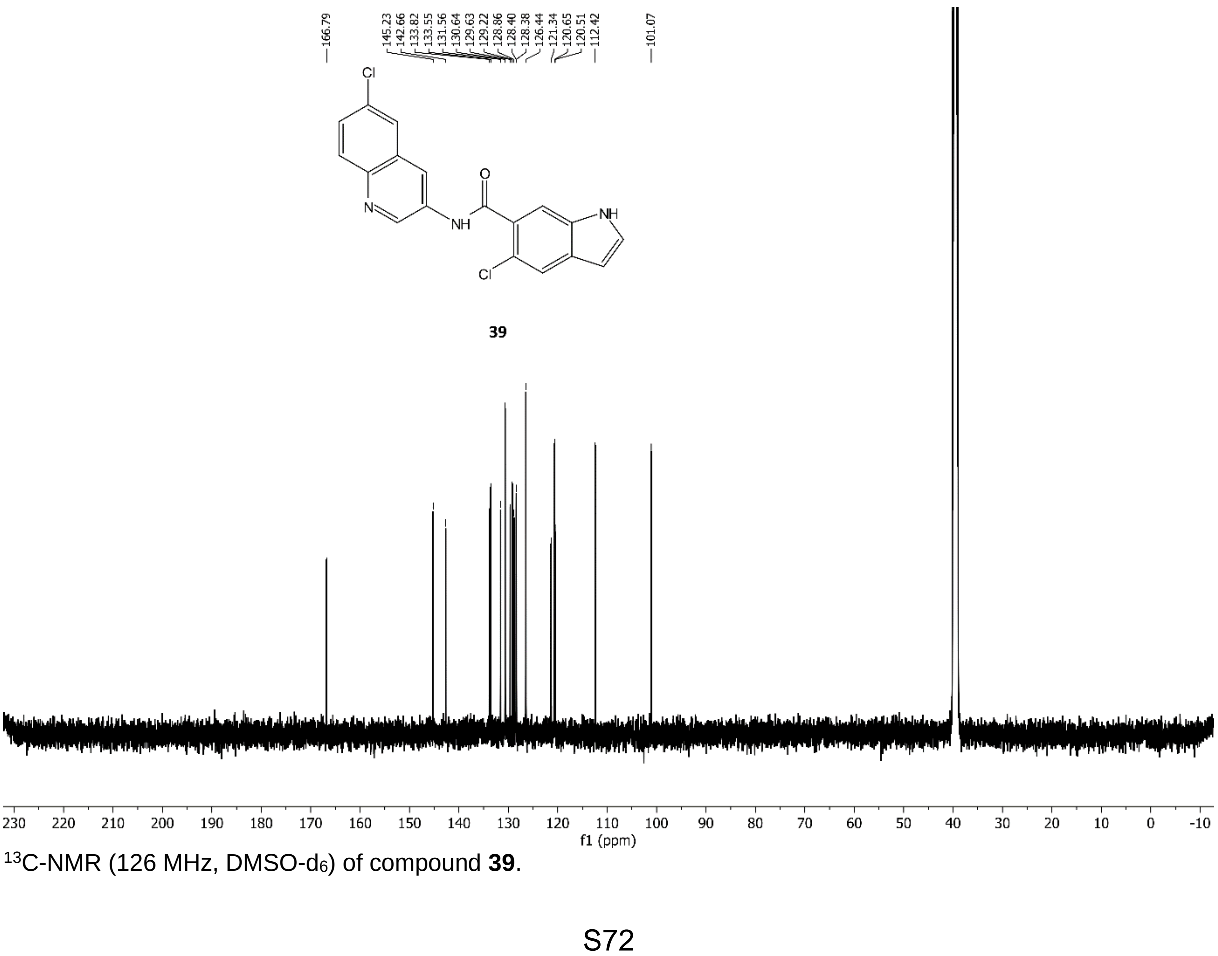

^{13}C-NMR (126 MHz, DMSO-d$_6$) of compound **39**.

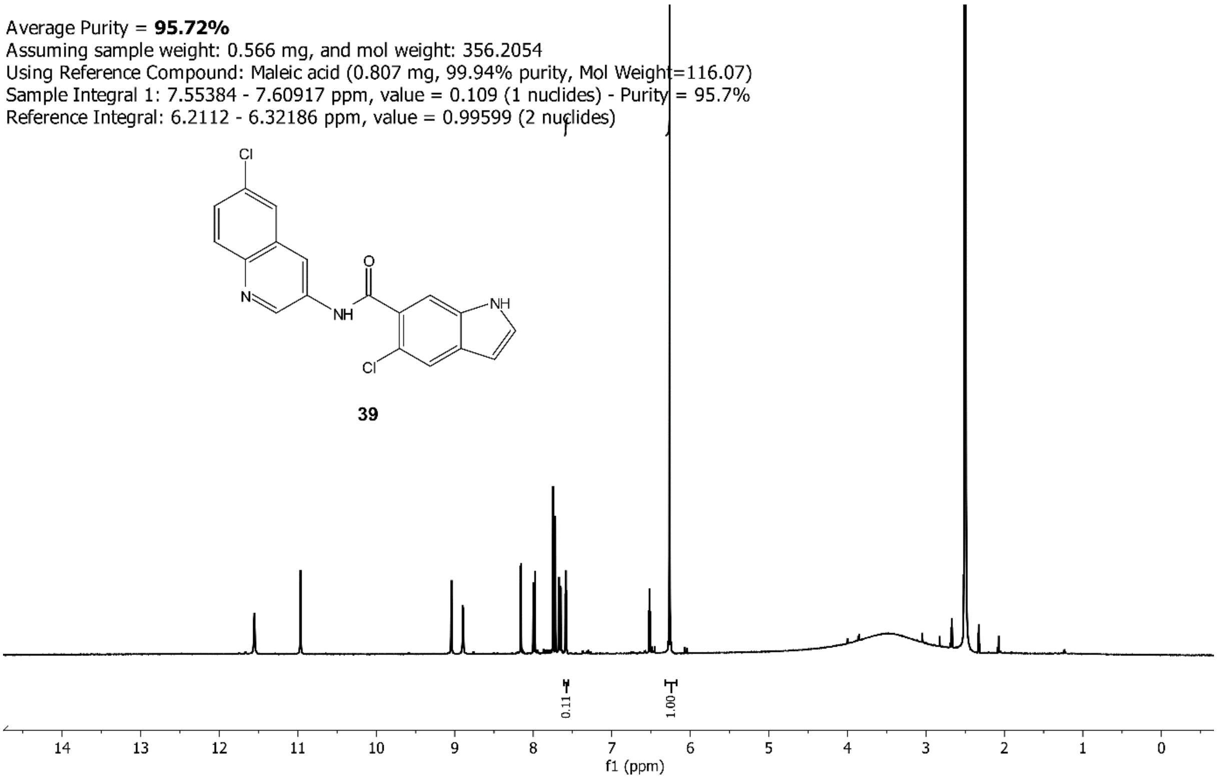

qHNMR (400 MHz, DMSO-d$_6$, maleic acid as reference) of compound **39**.

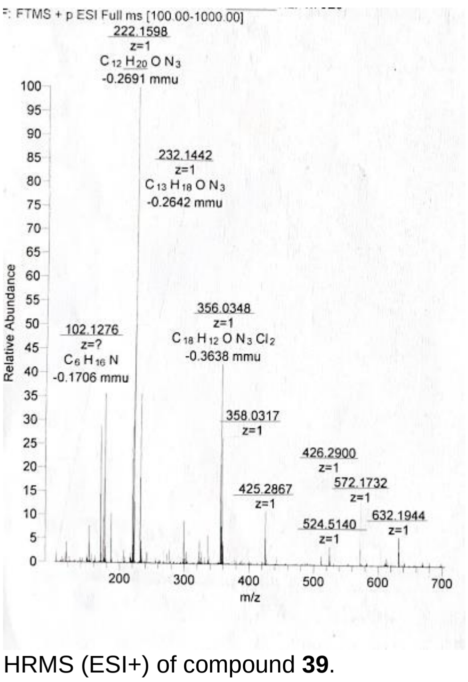

HRMS (ESI+) of compound **39**.

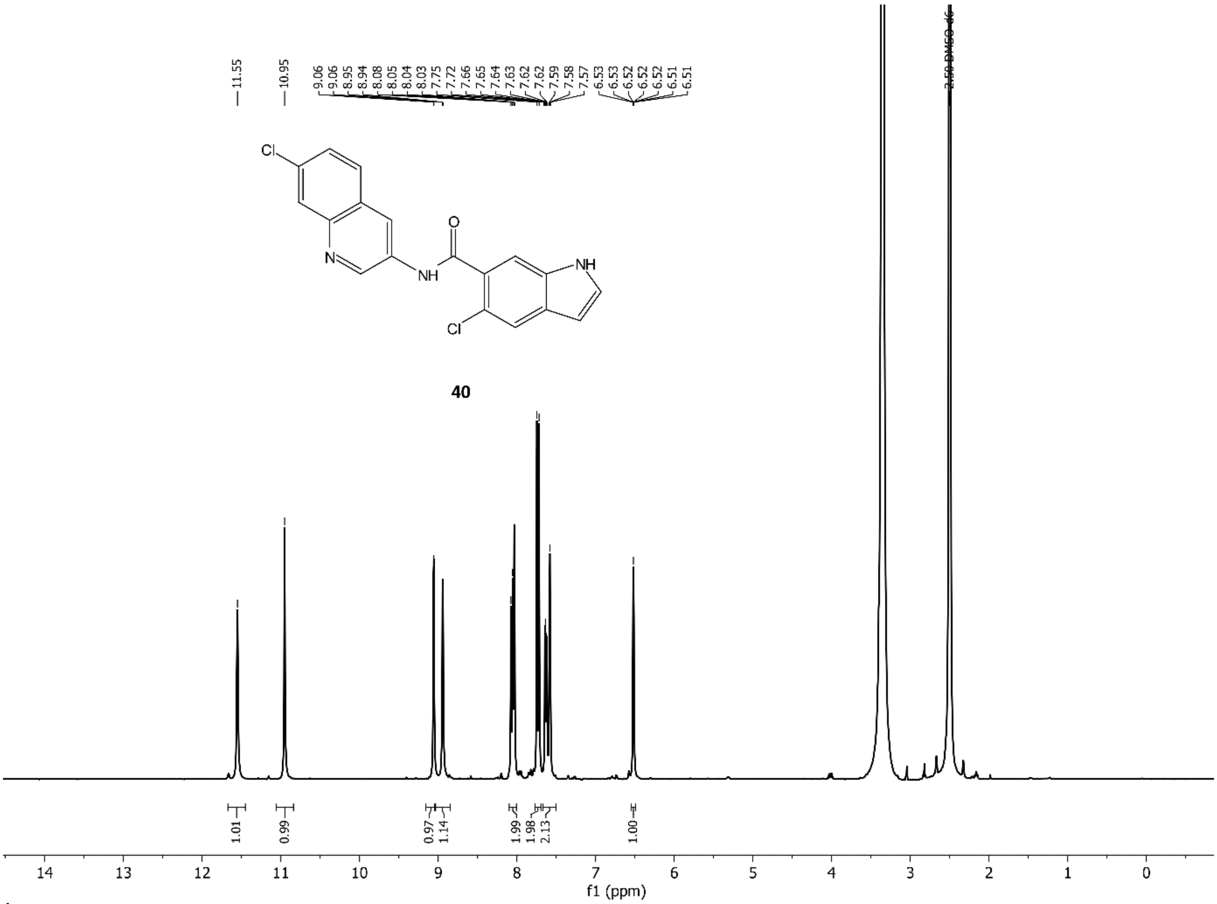

^{1}H-NMR (500 MHz, DMSO-d$_6$) of compound **40**.

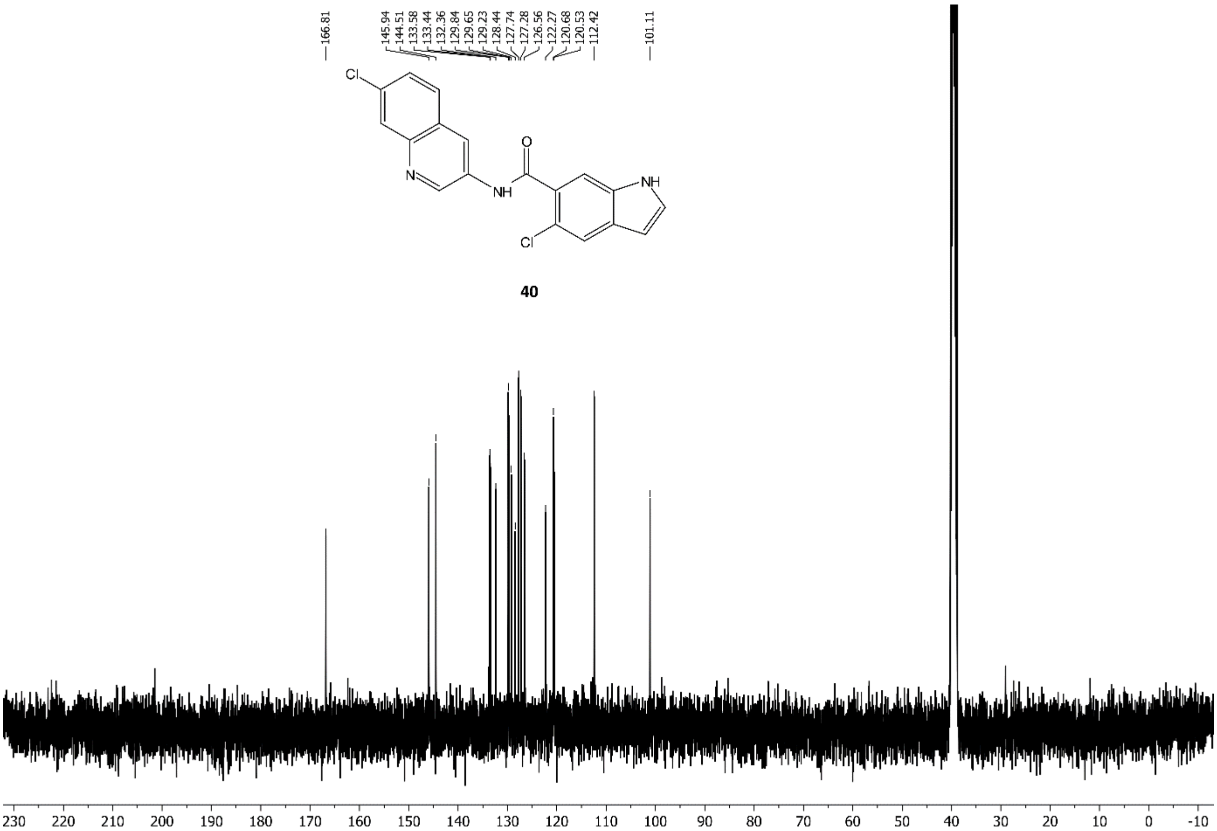

^{13}C-NMR (126 MHz, DMSO-d$_6$) of compound **40**.

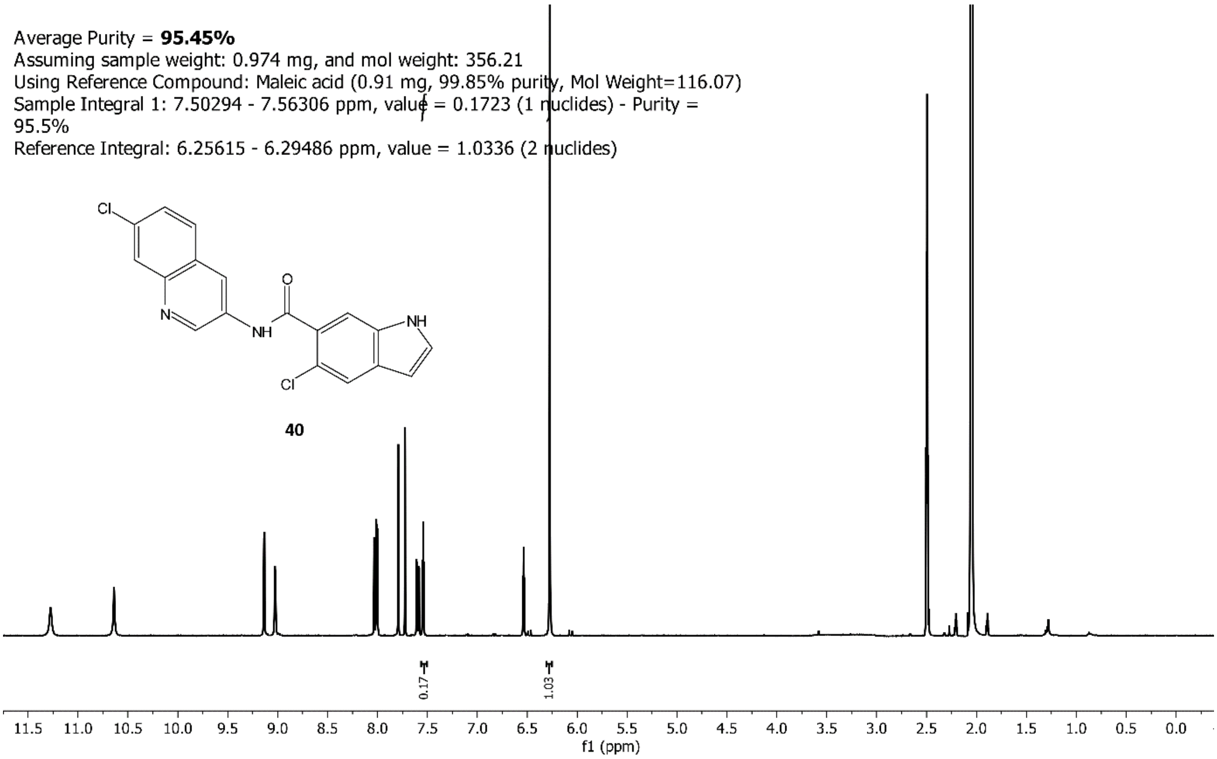

qHNMR (400 MHz, acetone-d_6 + 2 drops DMSO-d_6, maleic acid as reference) of compound **40**.

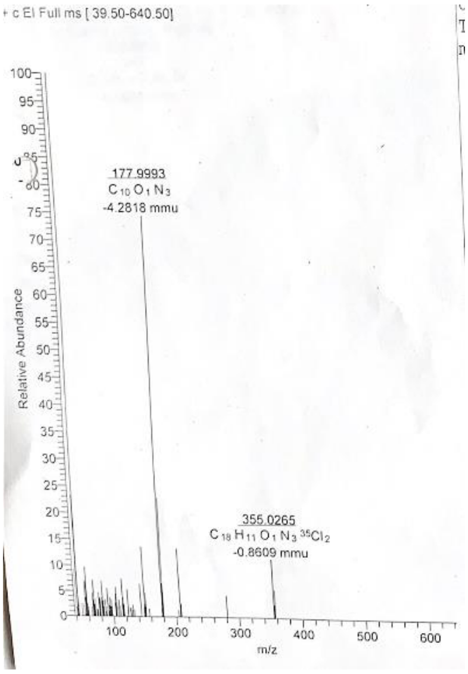

HRMS (EI+) of compound **40**.

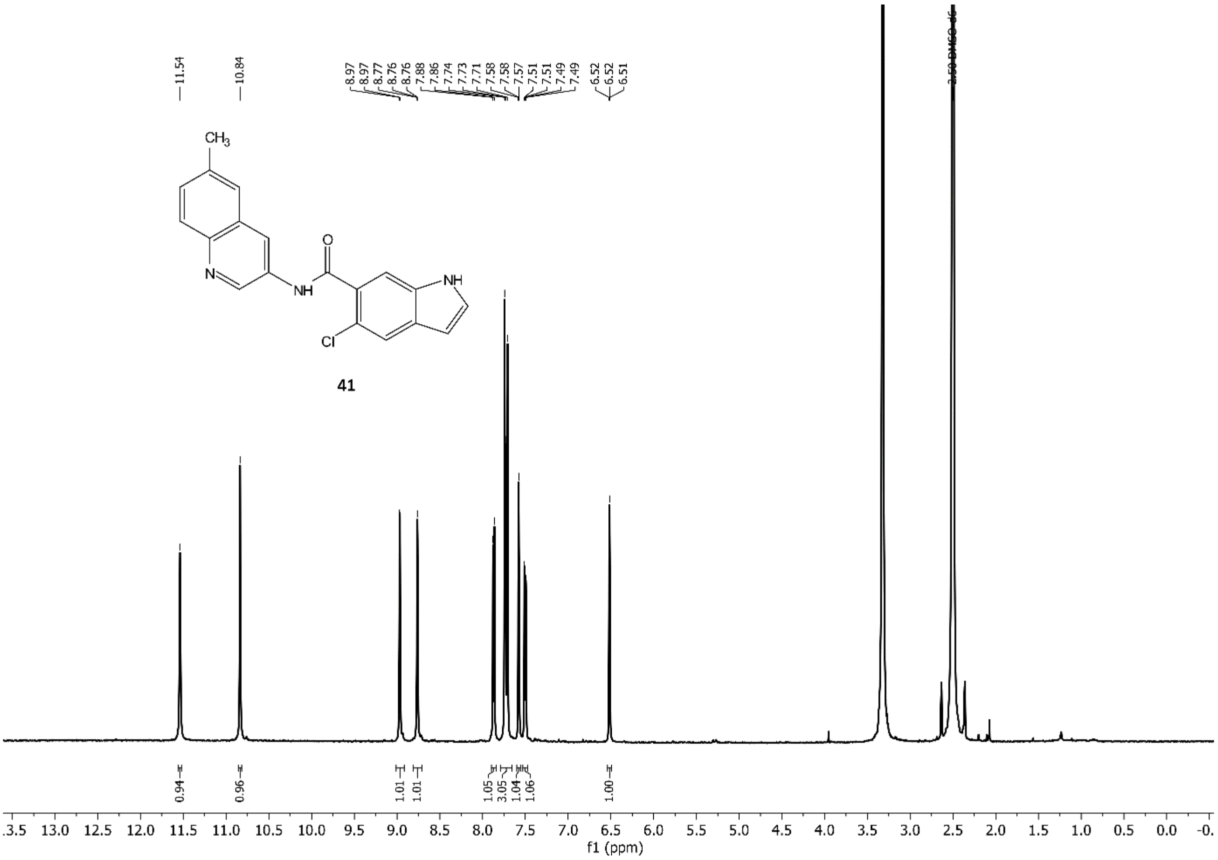

^{1}H-NMR (500 MHz, DMSO-d_6) of compound **41**.

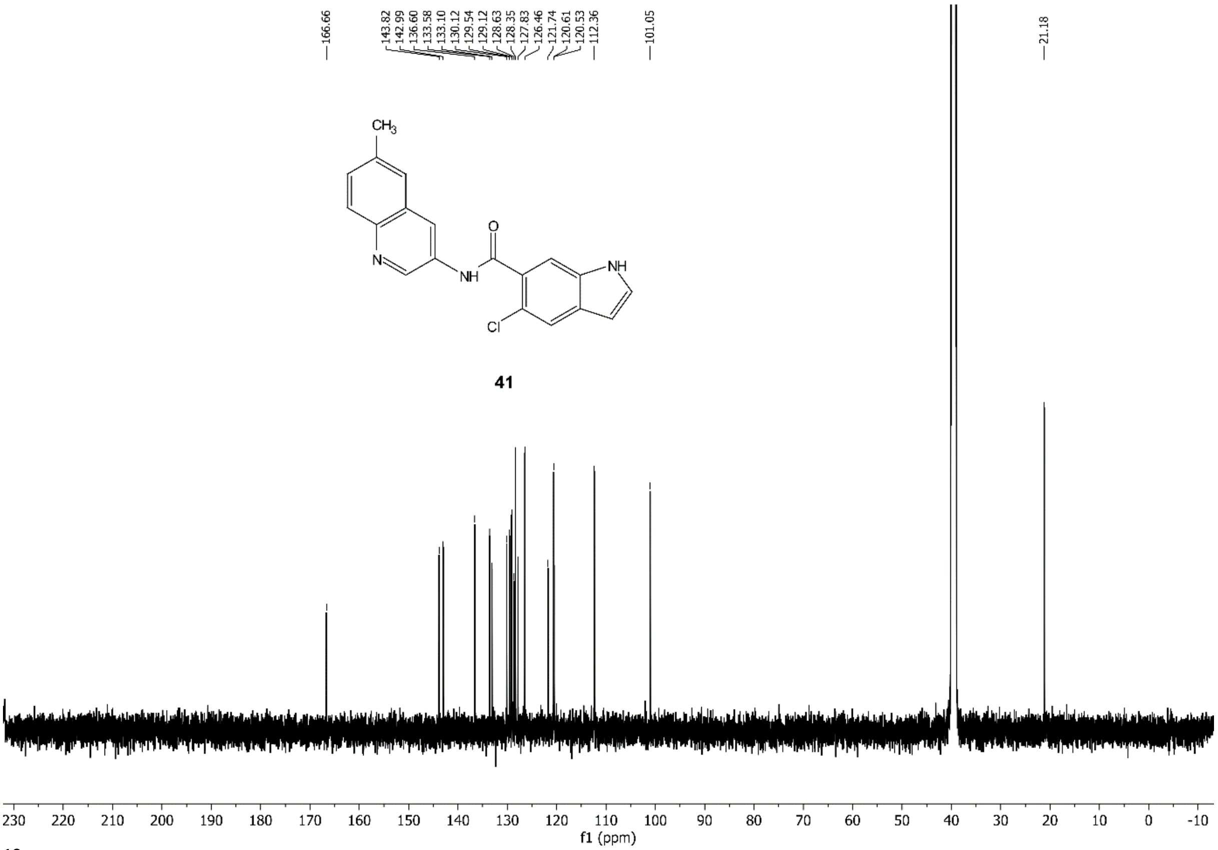

^{13}C-NMR (126 MHz, DMSO-d_6) of compound **41**.

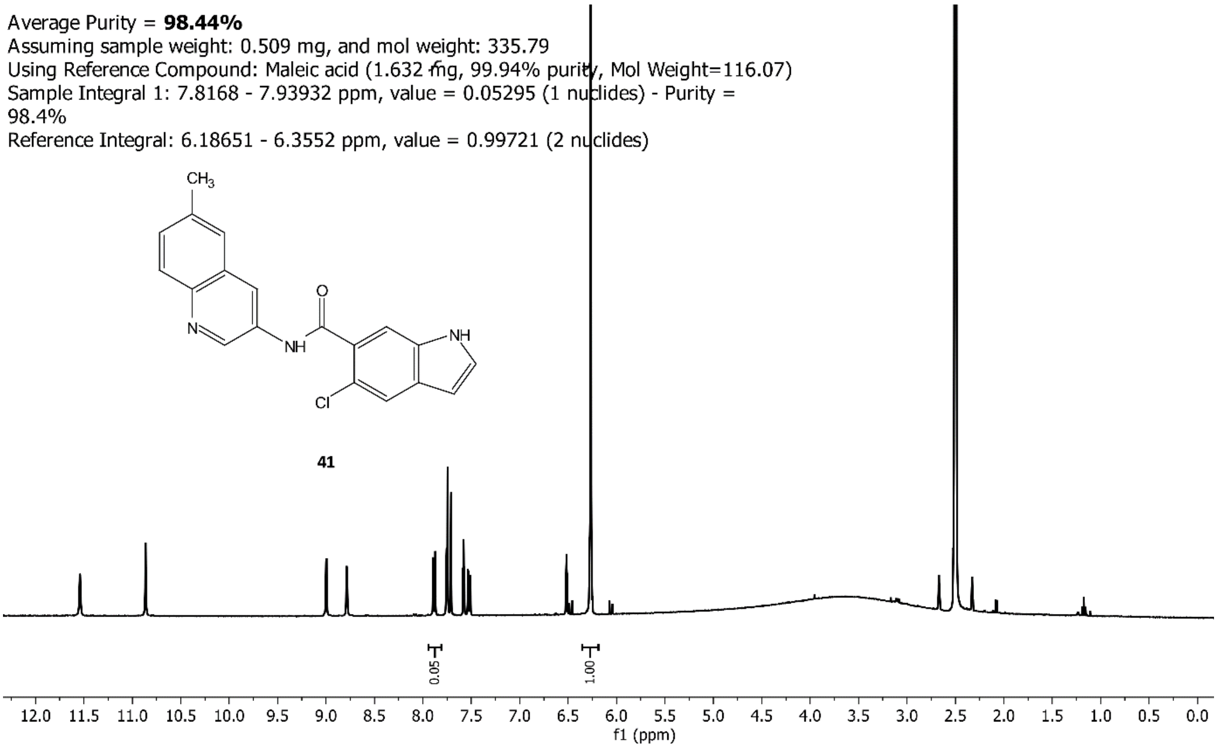

qHNMR (400 MHz, DMSO-d_6, maleic acid as reference) of compound **41**.

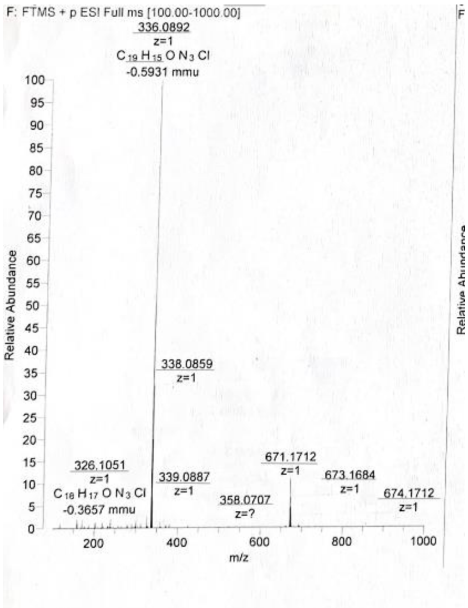

HRMS (ESI+) of compound **41**.

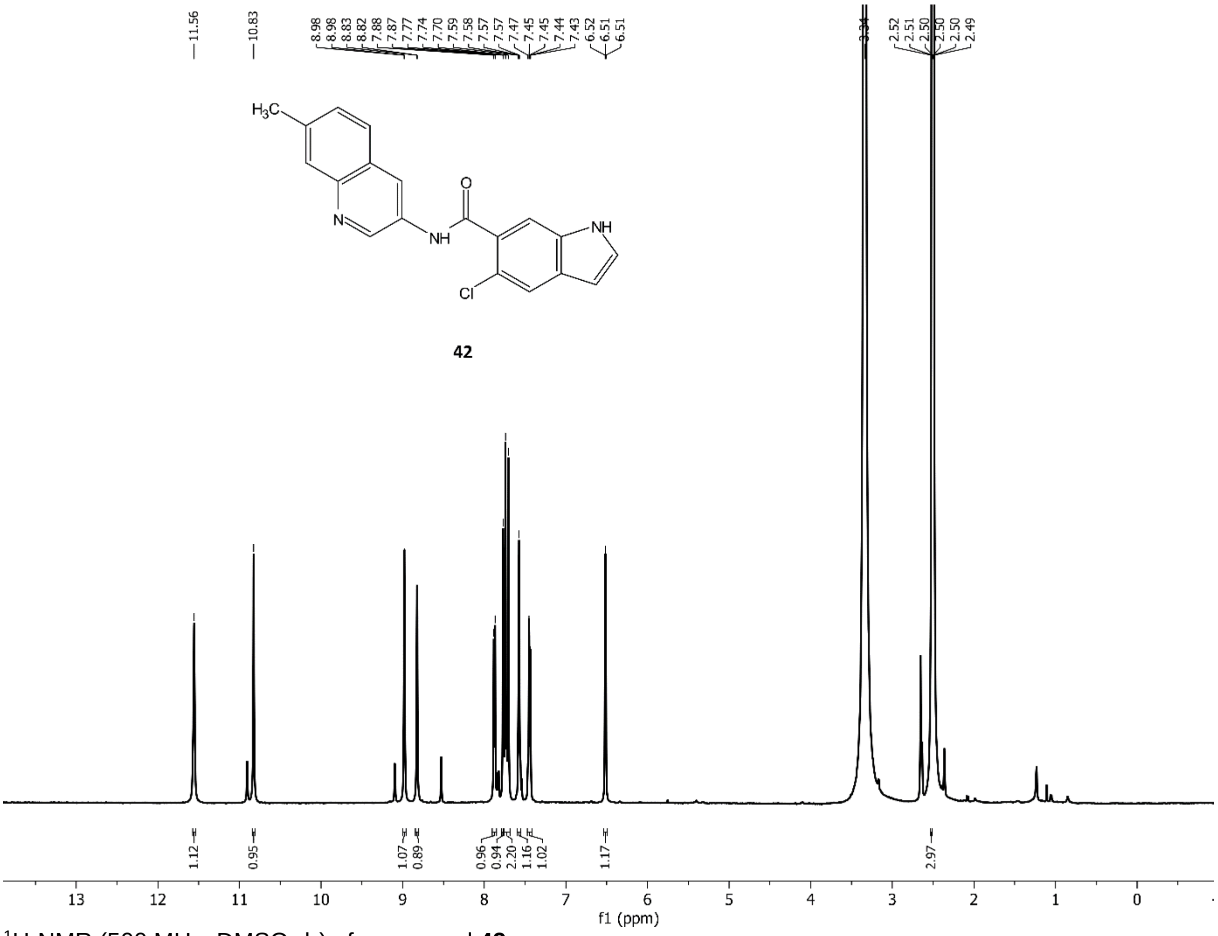

^{1}H-NMR (500 MHz, DMSO-d_6) of compound **42**.

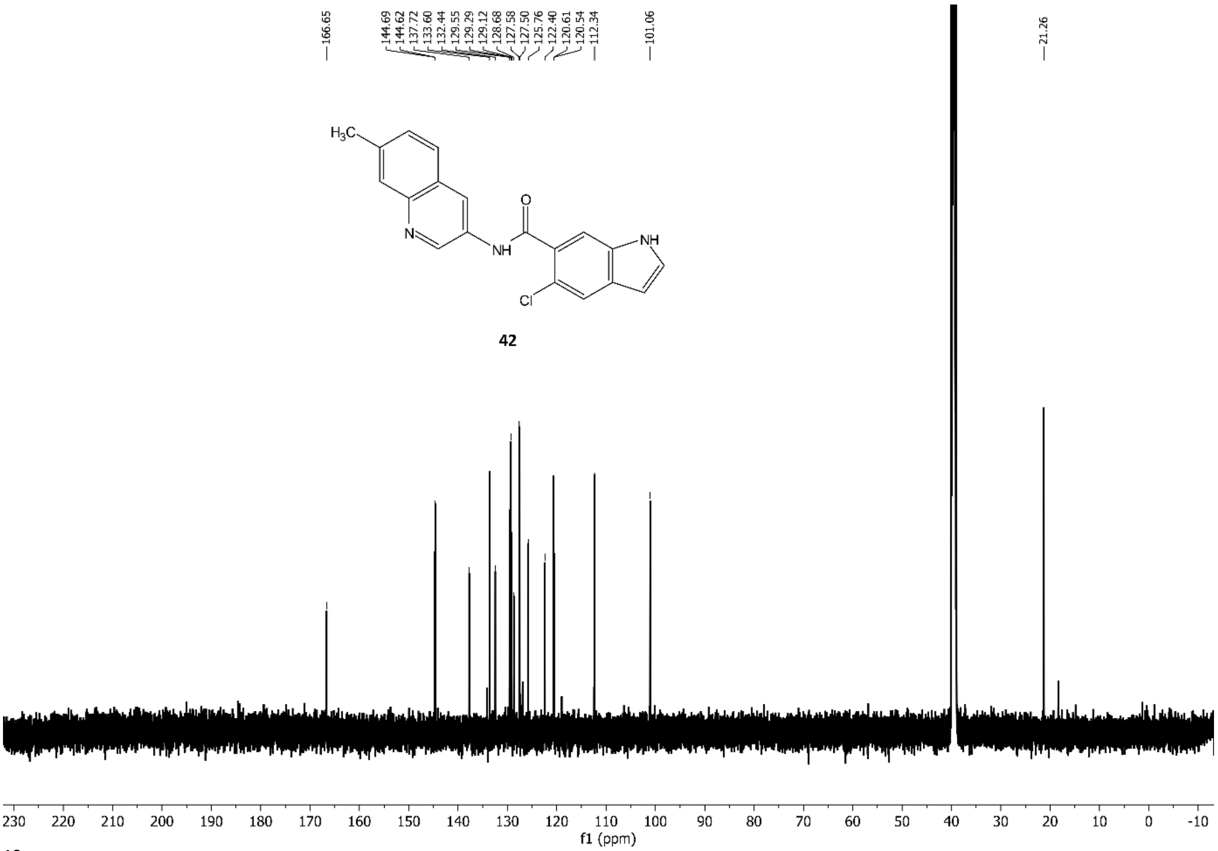

^{13}C-NMR (126 MHz, DMSO-d_6) of compound **42.**

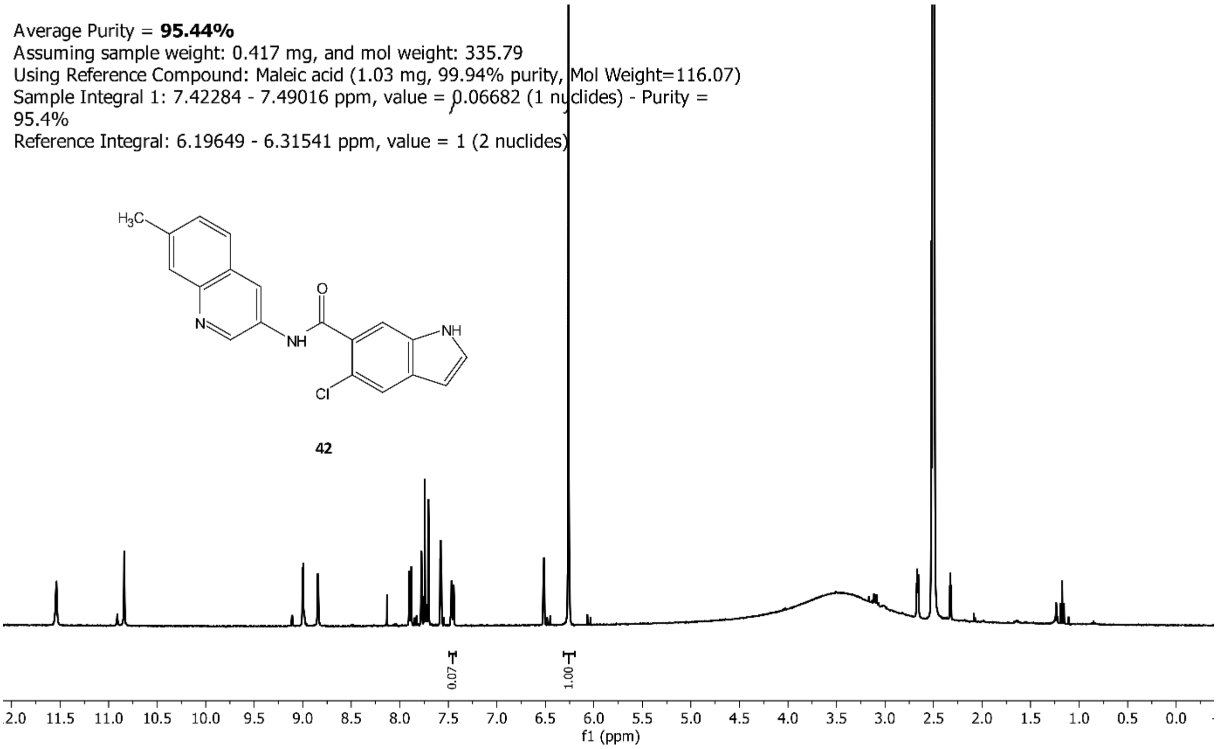

qHNMR (400 MHz, DMSO-d$_6$, maleic acid as reference) of compound **42**.

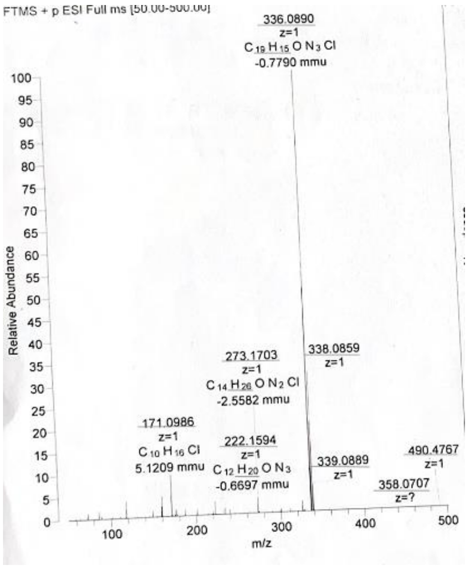

HRMS (ESI+) of compound **42**.

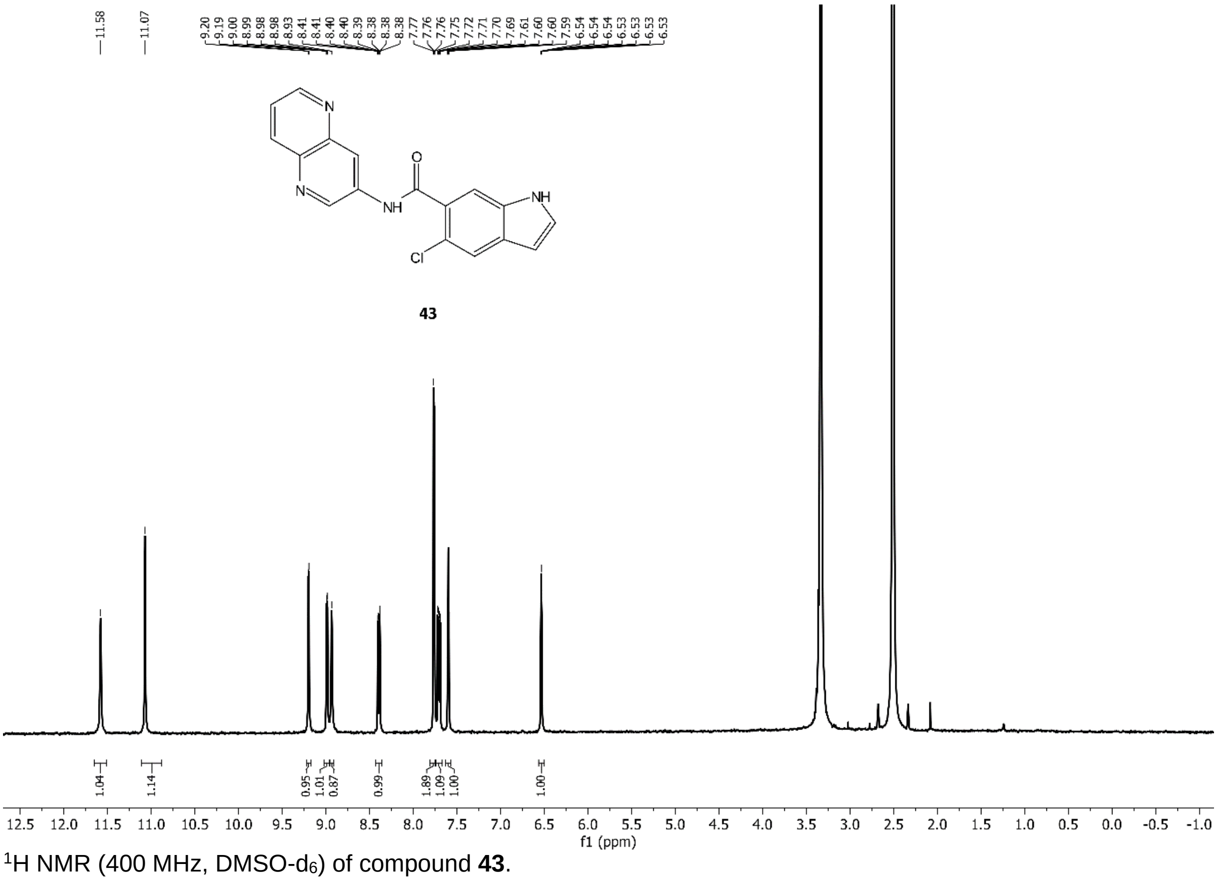

^{1}H NMR (400 MHz, DMSO-d6) of compound **43**.

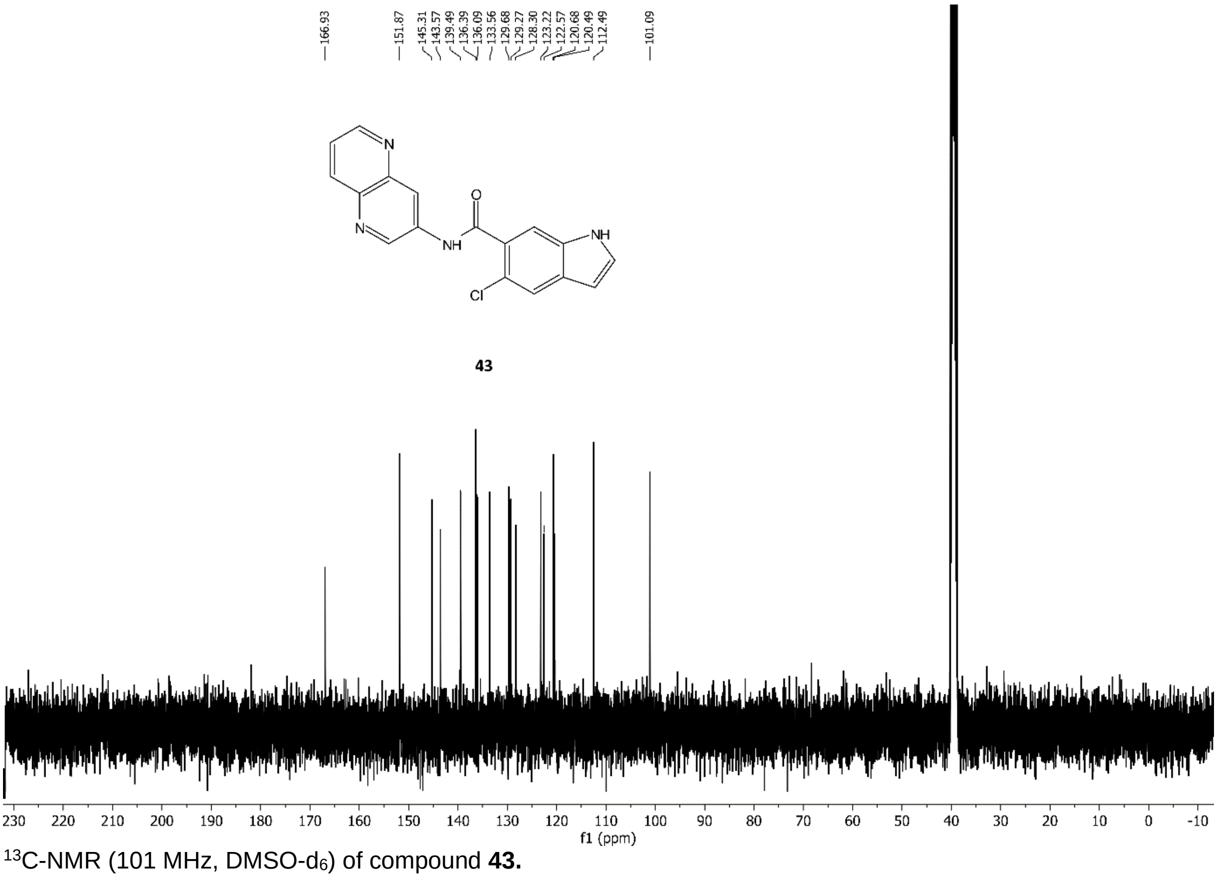

^{13}C-NMR (101 MHz, DMSO-d6) of compound **43.**

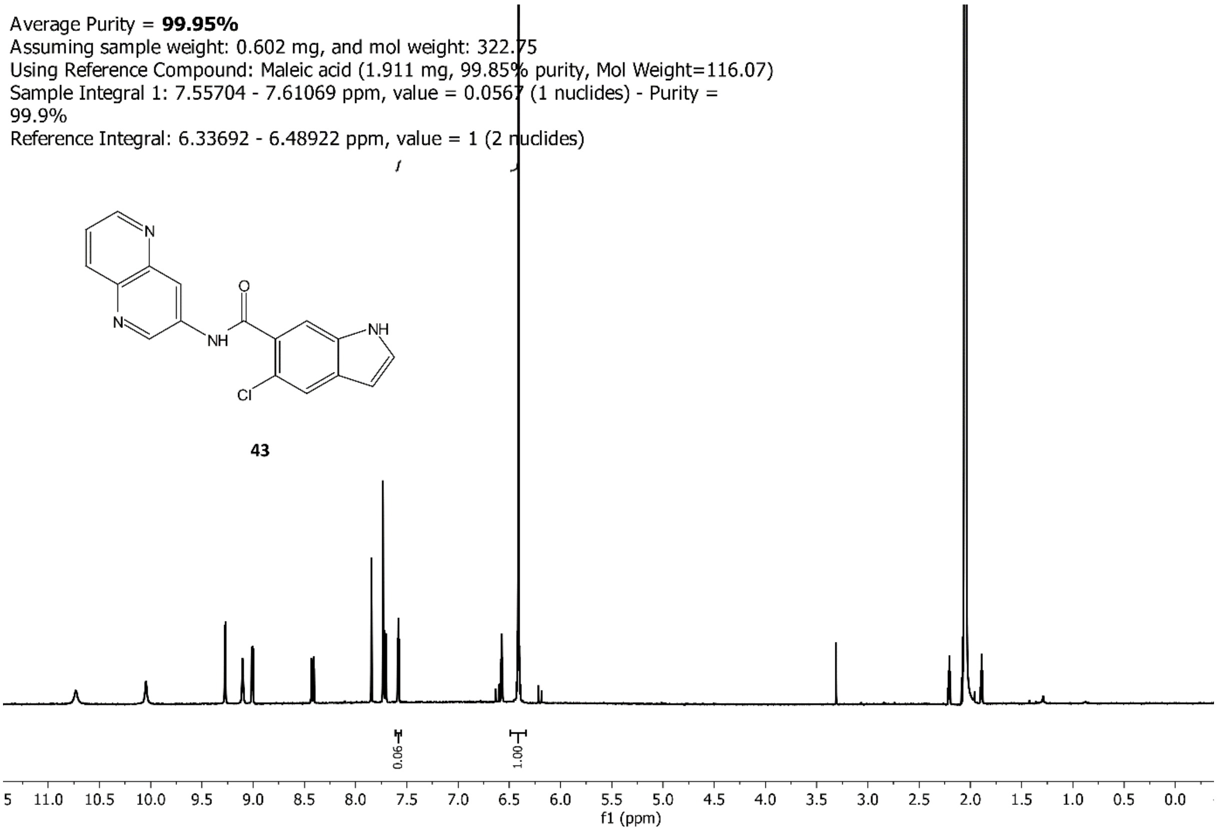

qHNMR (400 MHz, acetone-d$_6$, maleic acid as reference) of compound **43**.

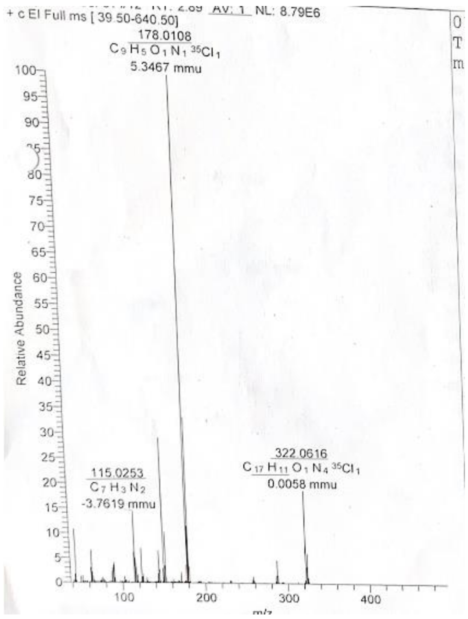

HRMS (EI+) of compound **43**.

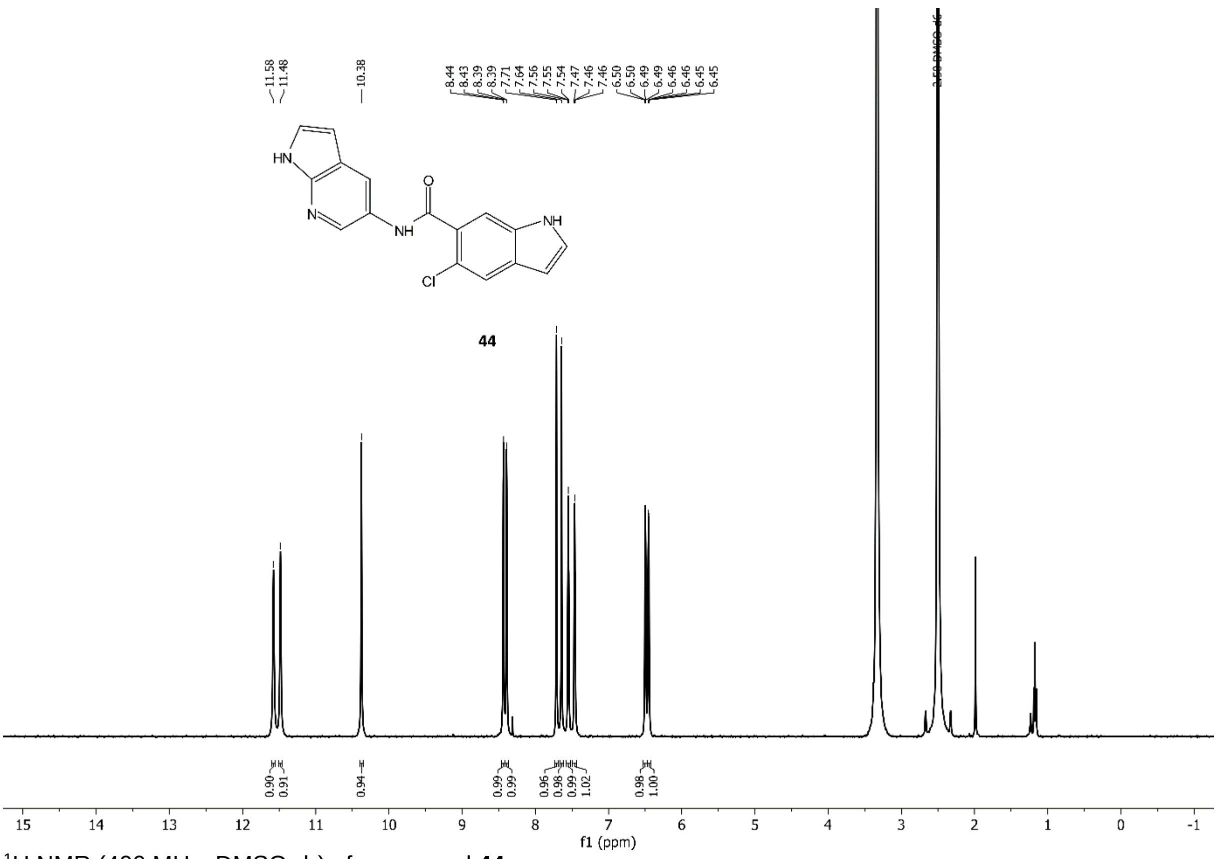

^{1}H NMR (400 MHz, DMSO-d$_6$) of compound **44**.

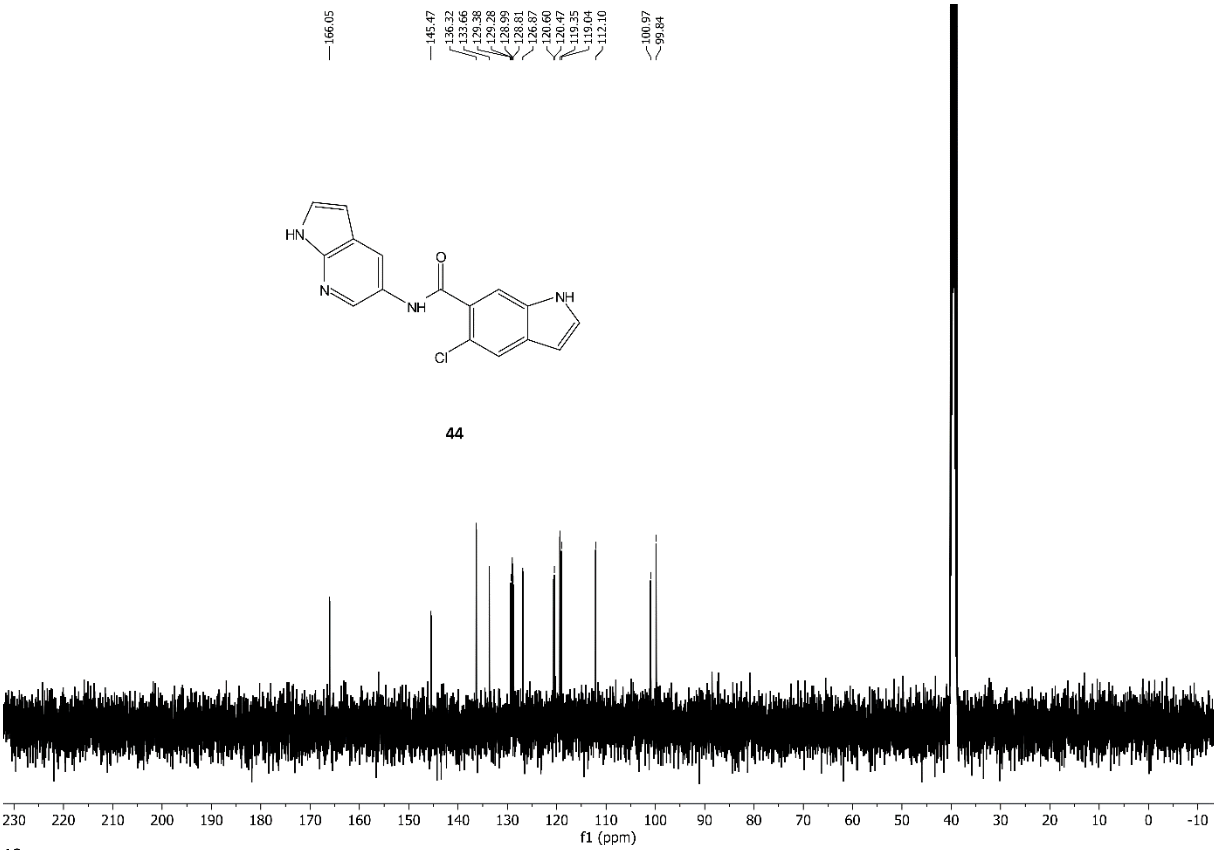

^{13}C-NMR (101 MHz, DMSO-d$_6$) of compound **44.**

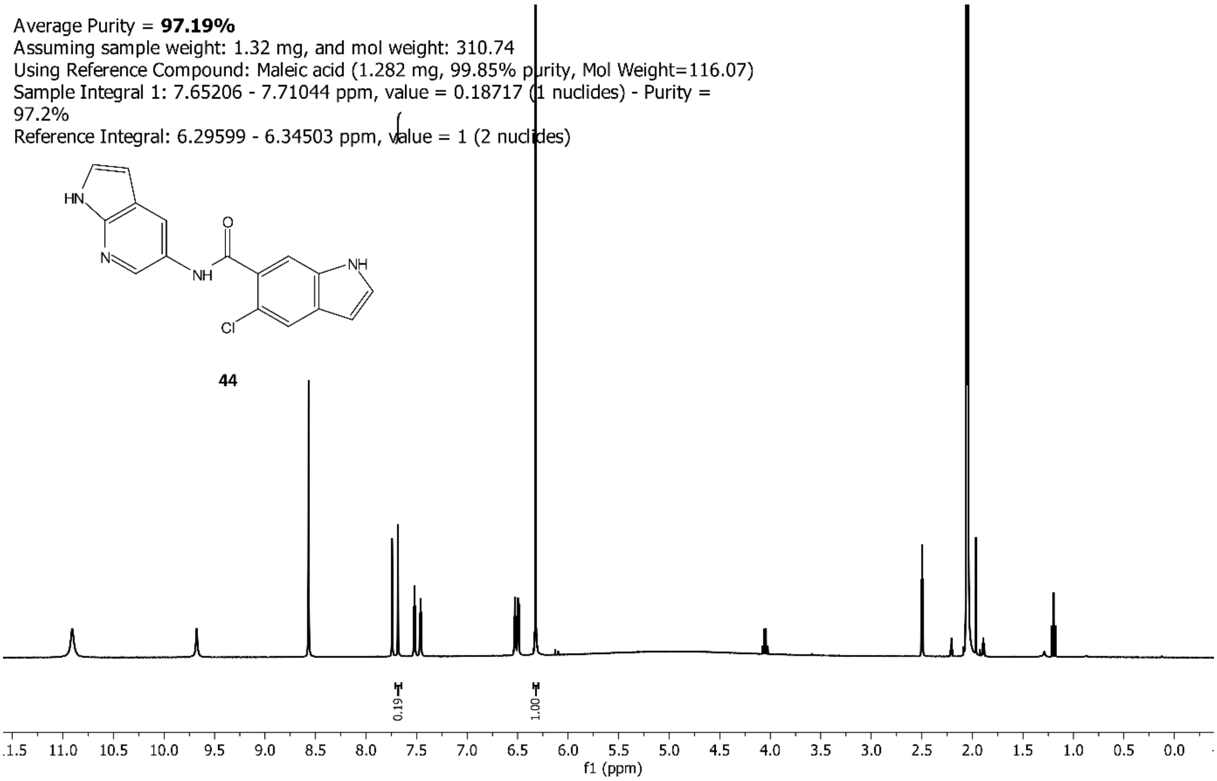

qHNMR (400 MHz, acetone-d_6 + 4 drops DMSO-d_6, maleic acid as reference) of compound **44**.

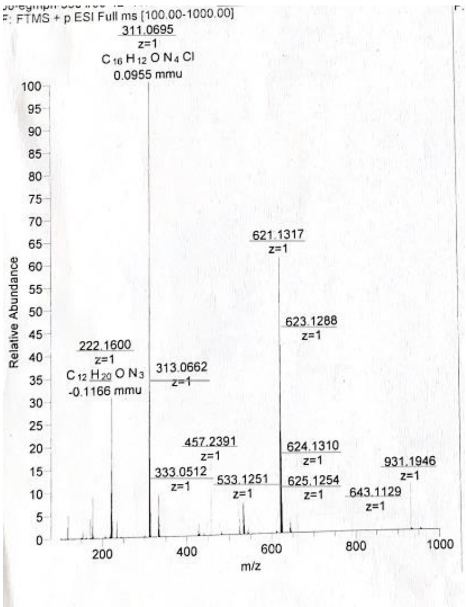

HRMS (ESI+) of compound **44**.

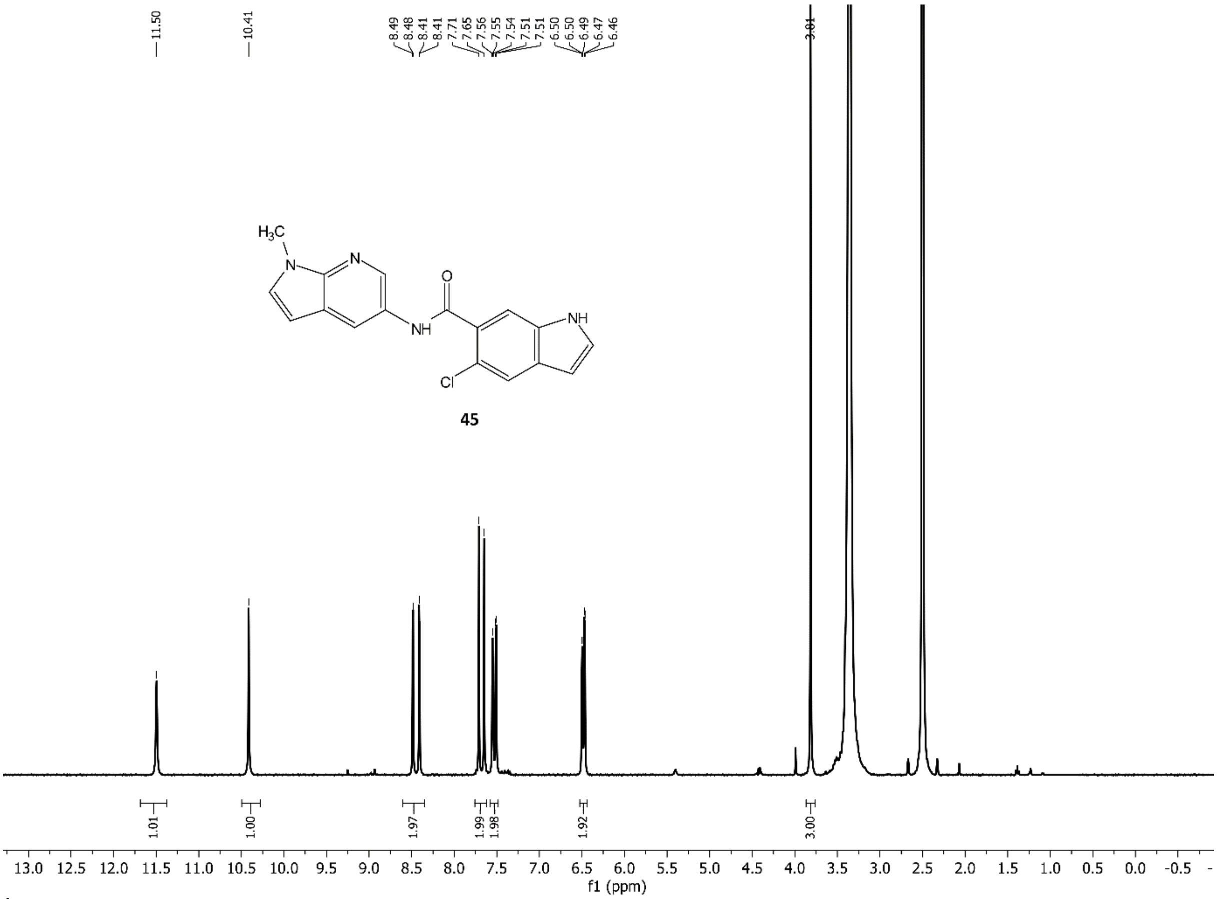

^1H NMR (400 MHz, DMSO-d_6) of compound **45**.

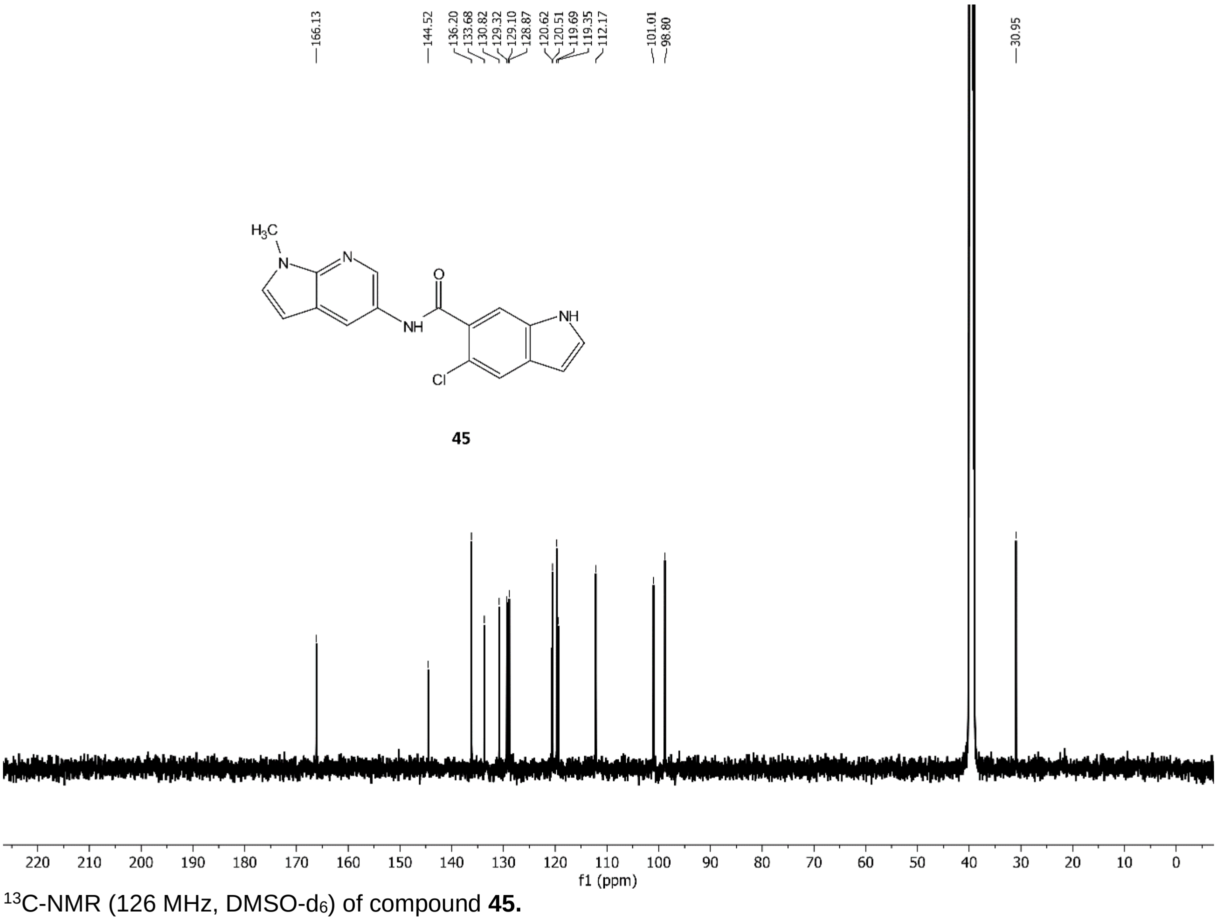

^{13}C-NMR (126 MHz, DMSO-d_6) of compound **45.**

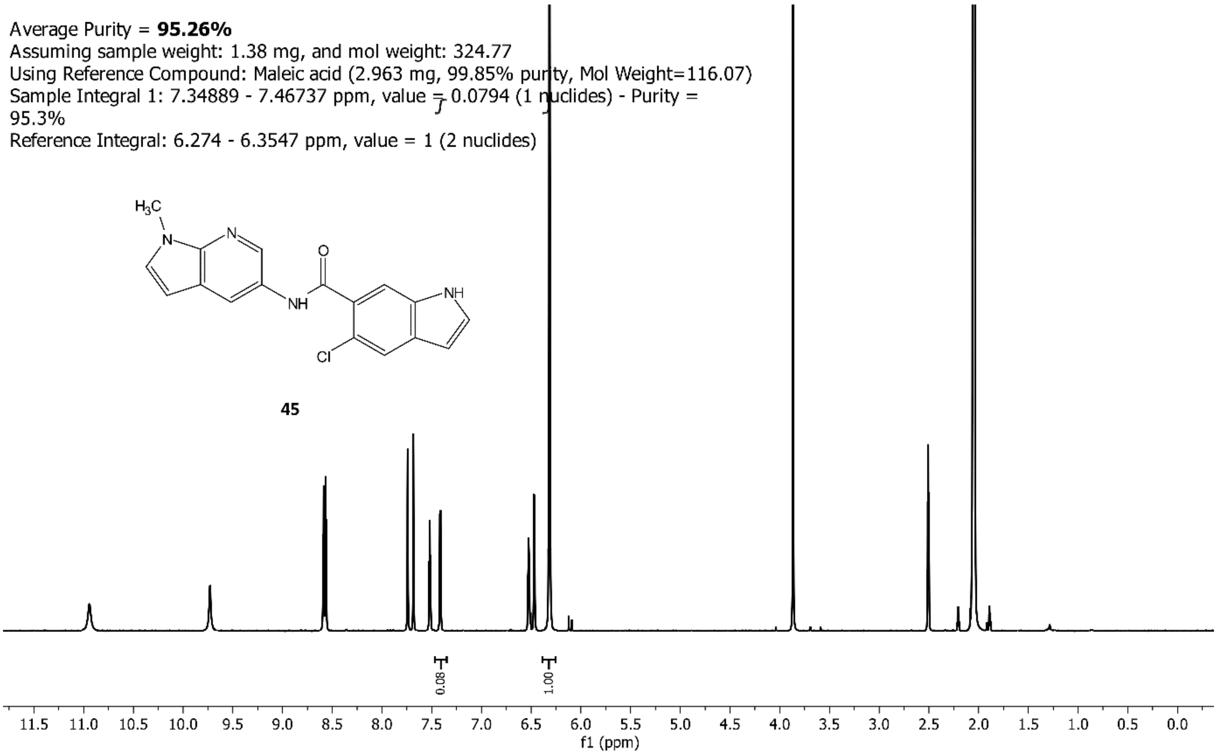

qHNMR (400 MHz, acetone-d_6 + 1 drop DMSO-d_6, maleic acid as reference) of compound **45**.

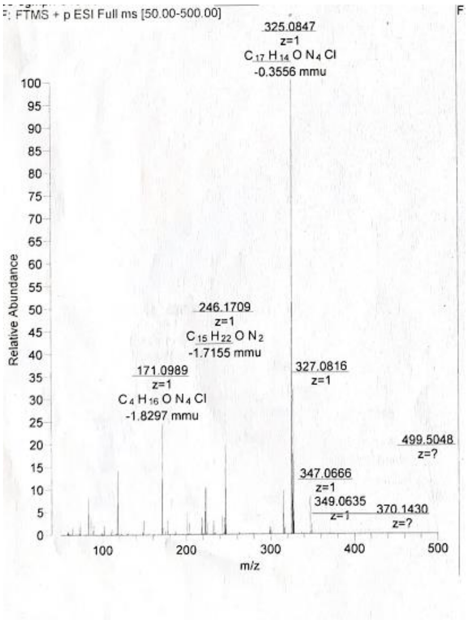

HRMS (ESI+) of compound **45**.

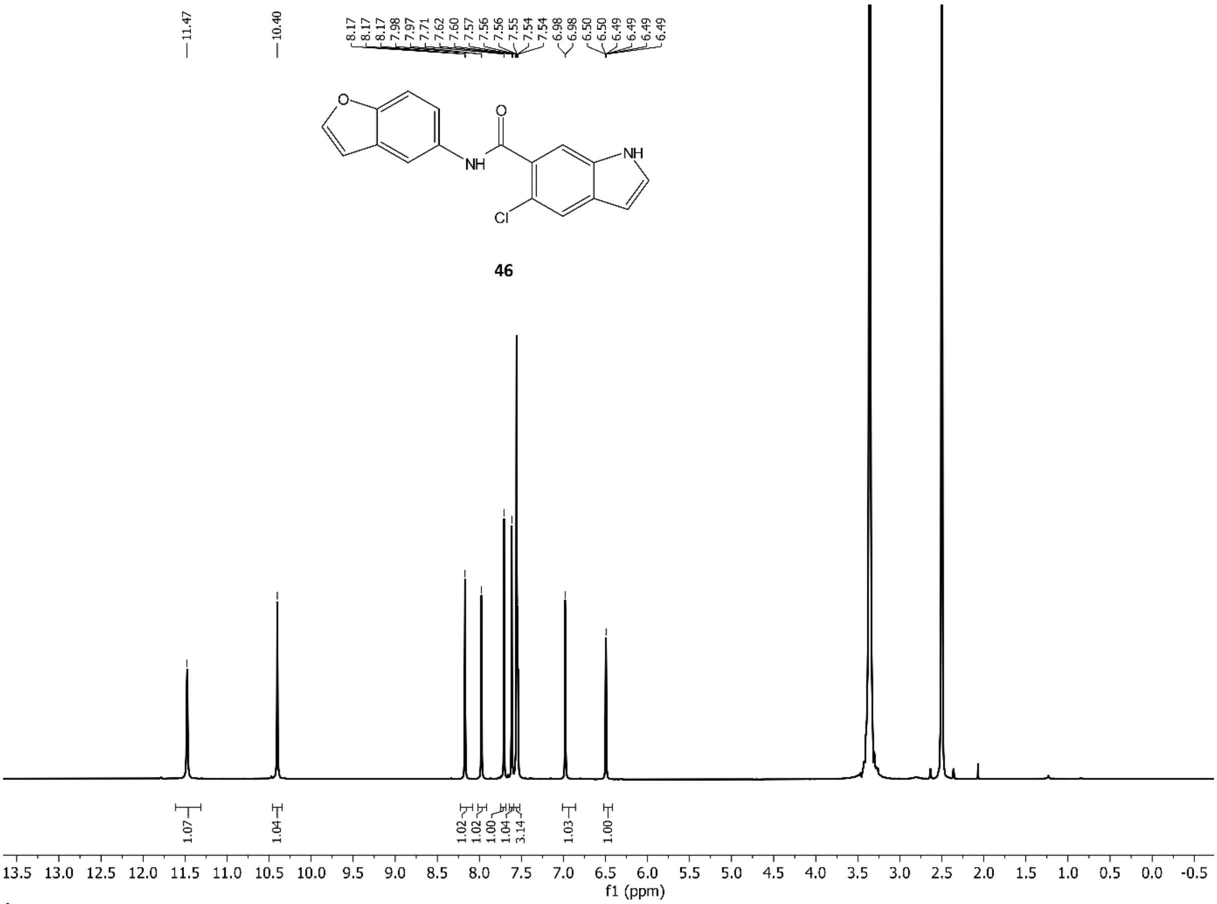

^{1}H NMR (500 MHz, DMSO-d_6) of compound **46**.

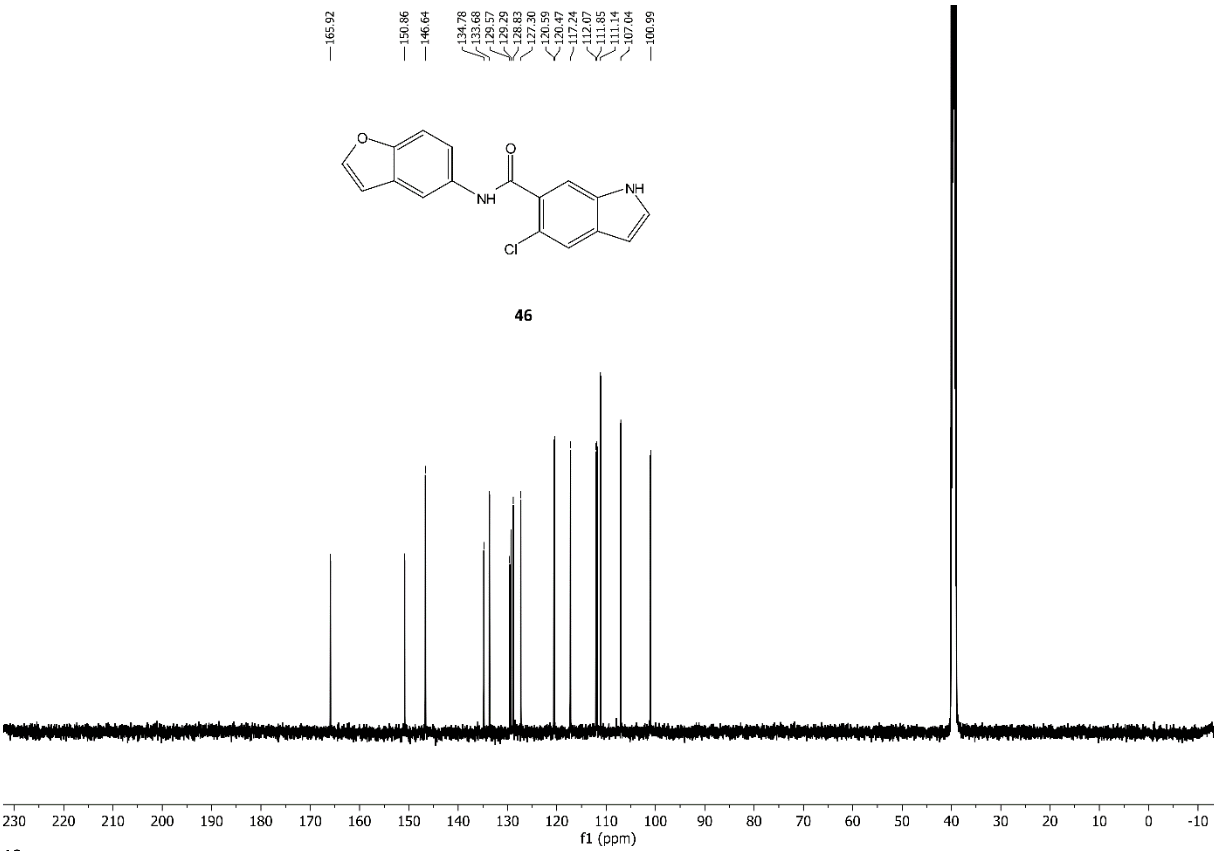

^{13}C-NMR (126 MHz, DMSO-d_6) of compound **46.**

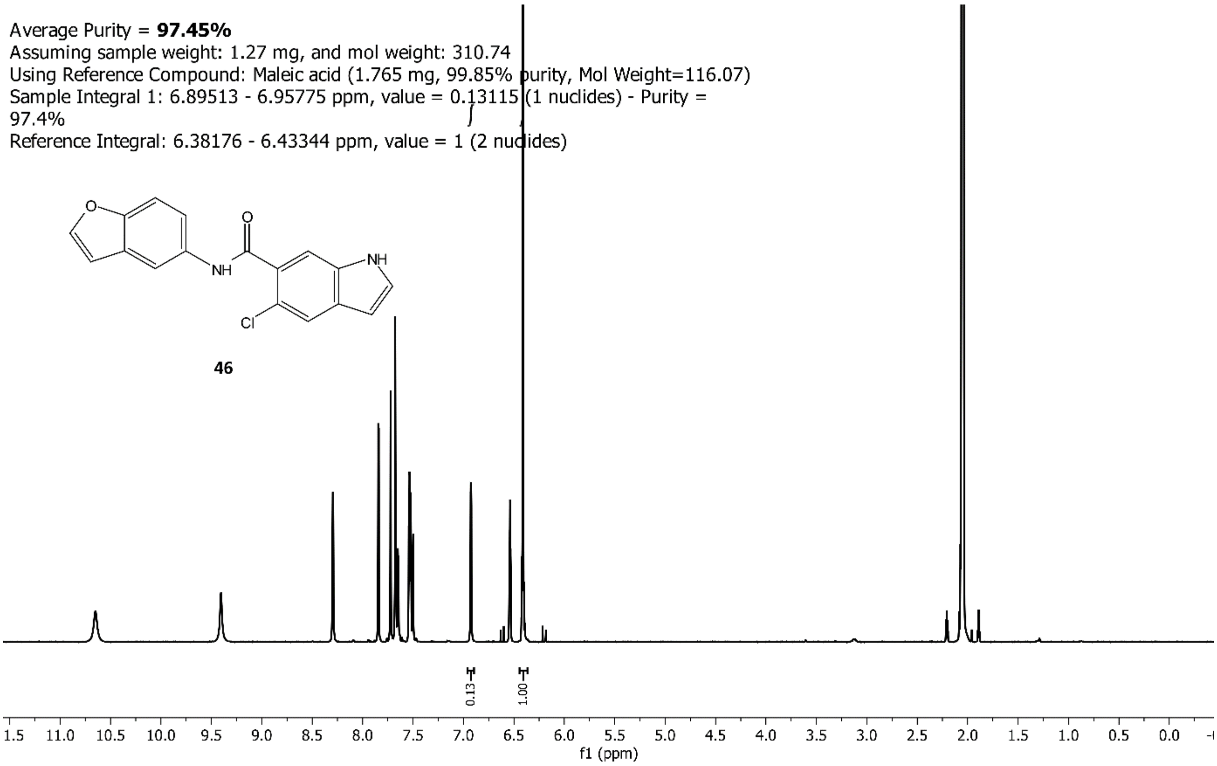

qHNMR (400 MHz, acetone-d$_6$, maleic acid as reference) of compound **46**.

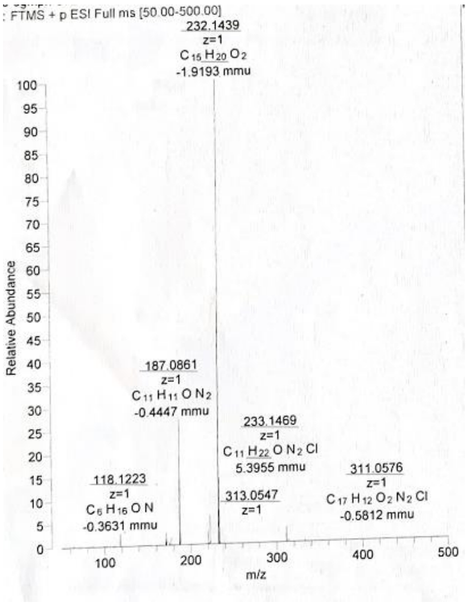

HRMS (ESI+) of compound **46**.

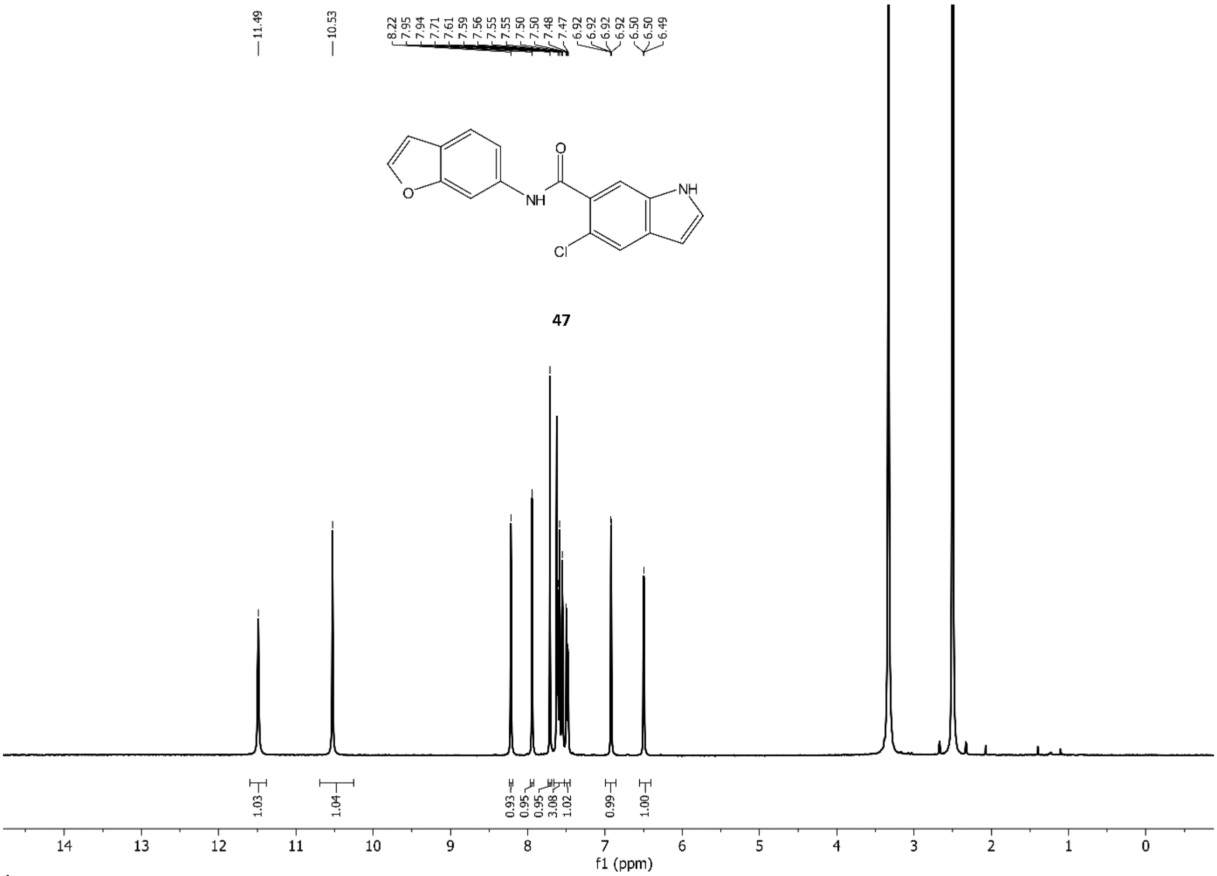

^{1}H-NMR (400 MHz, DMSO-d_6) of compound **47**.

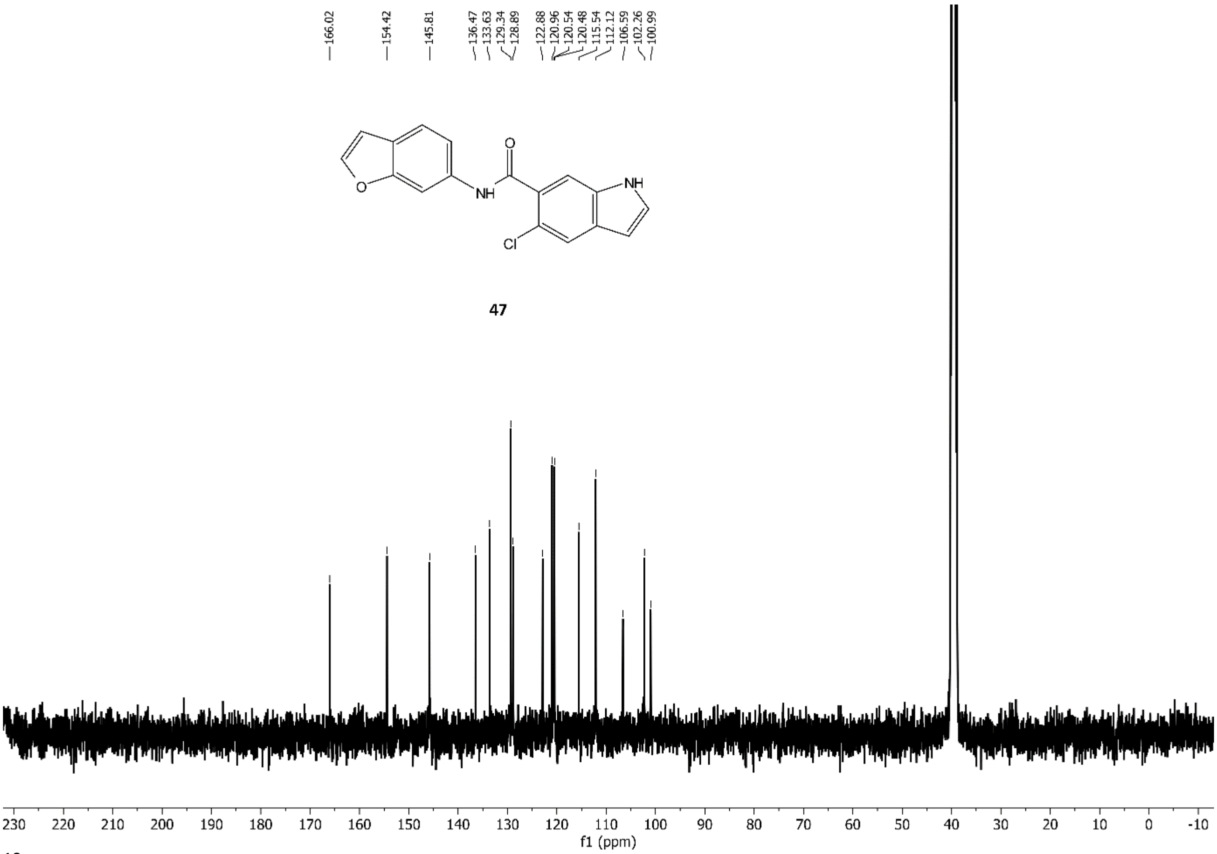

^{13}C-NMR (101 MHz, DMSO-d_6) of compound **47.**

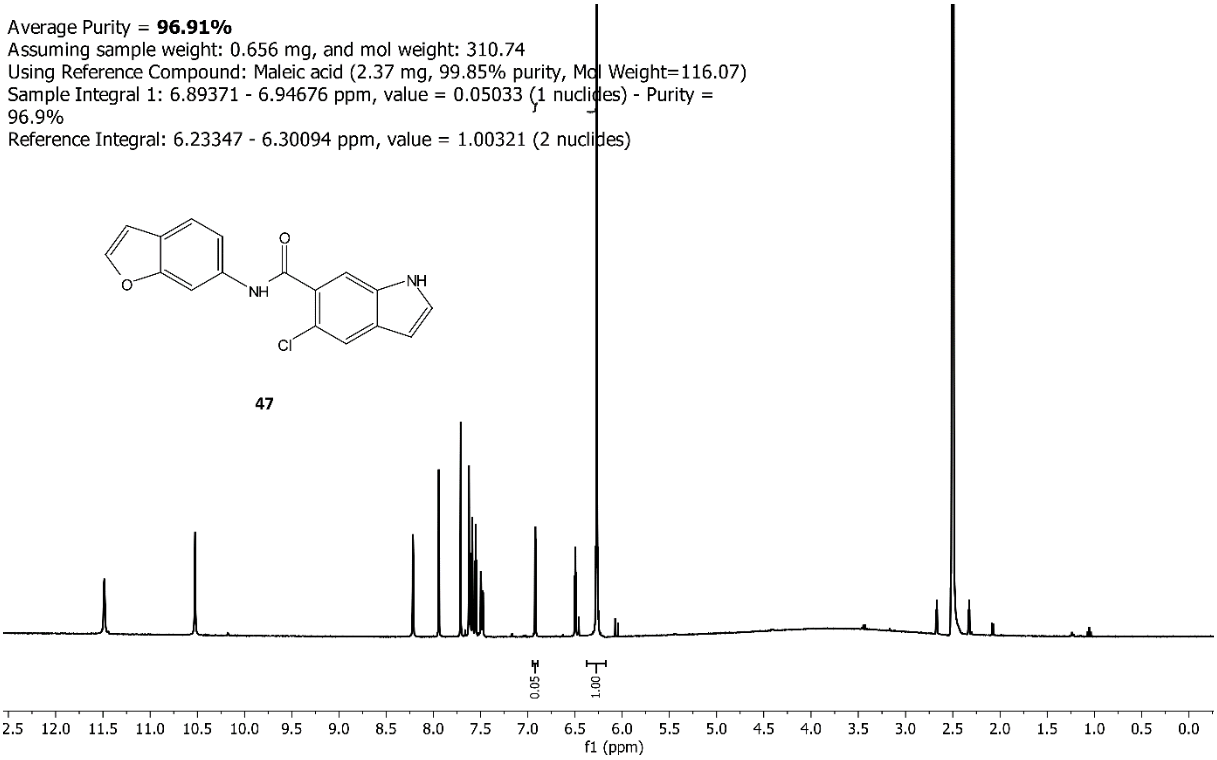

qHNMR (400 MHz, DMSO-d$_6$, maleic acid as reference) of compound **47**.

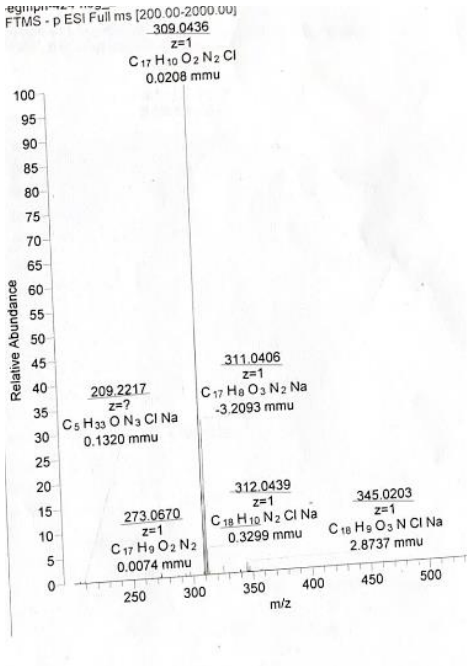

HRMS (ESI-) of compound **47**.

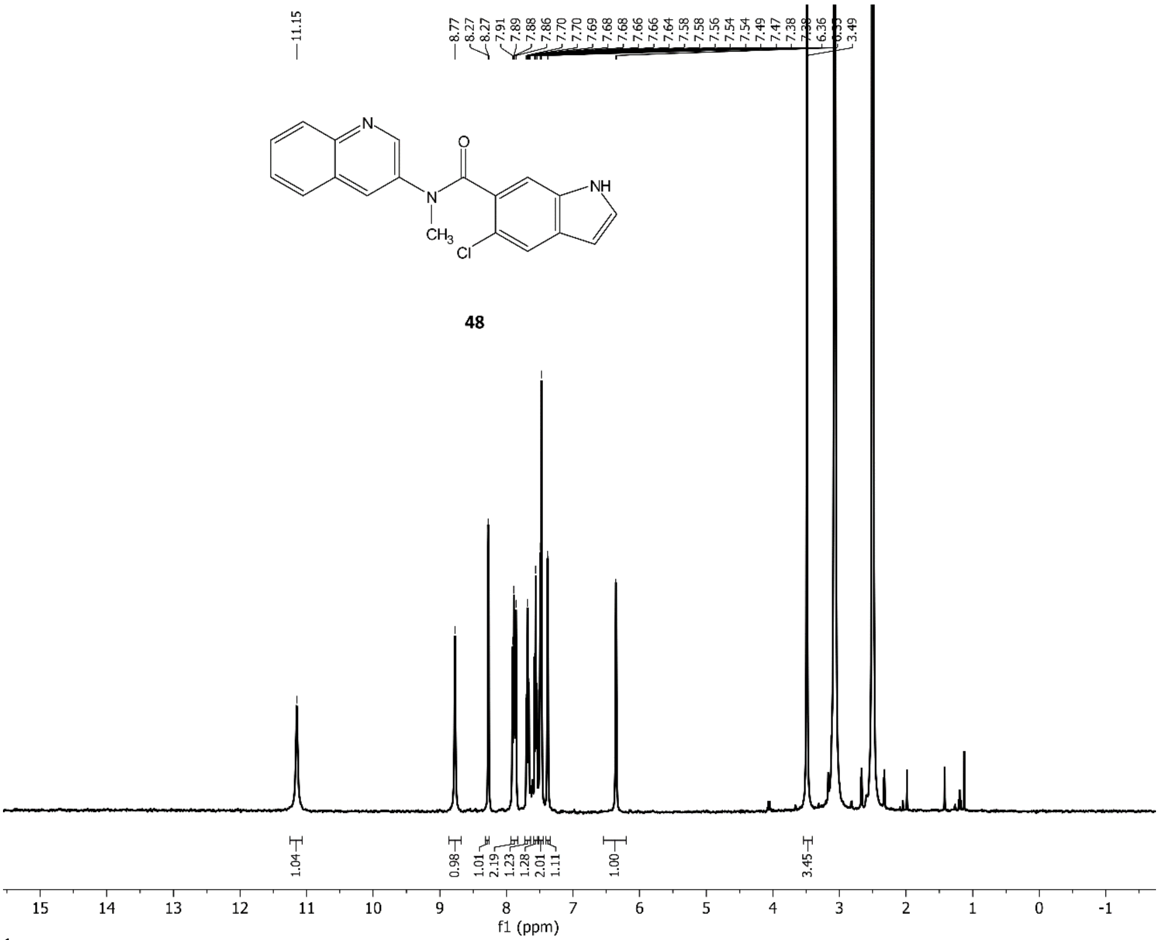

^{1}H-NMR (400 MHz, DMSO-d6, 80°C) of compound **48**.

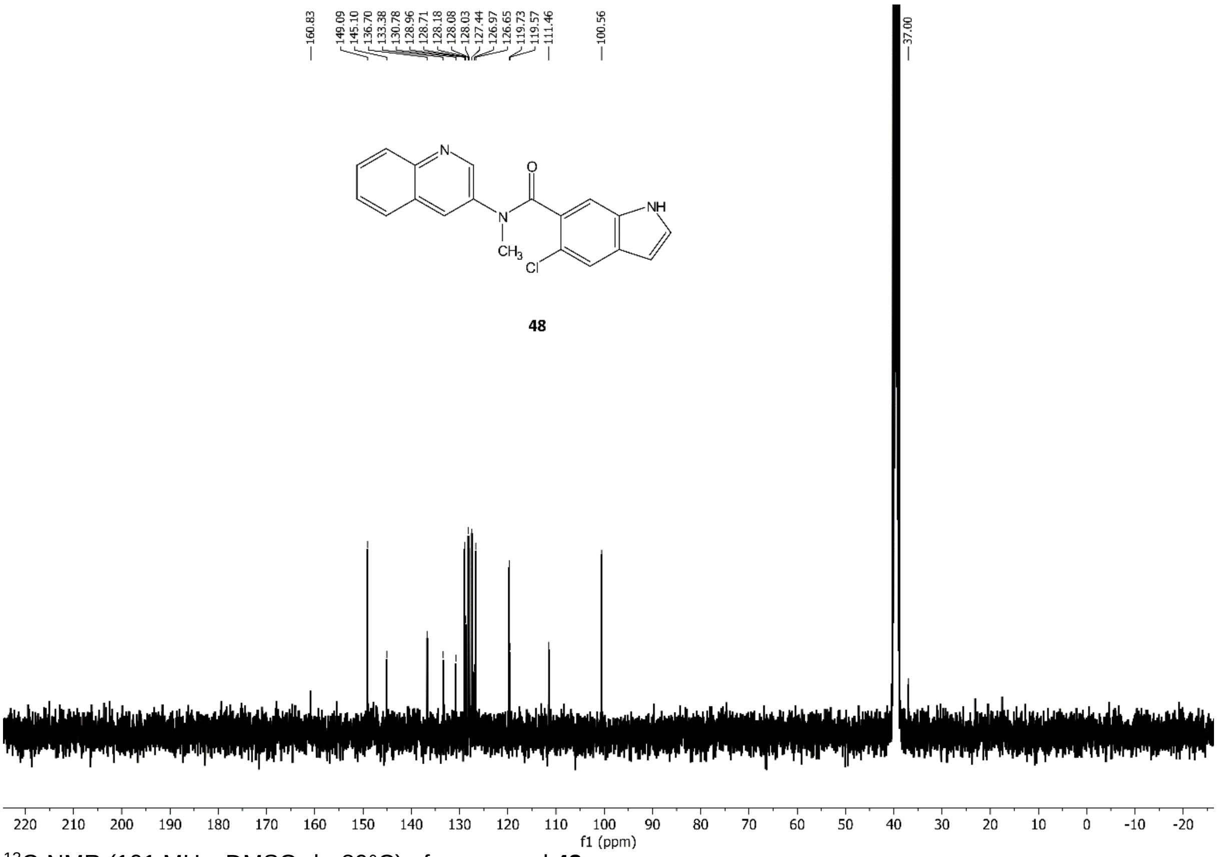

^{13}C-NMR (101 MHz, DMSO-d6, 80°C) of compound **48.**

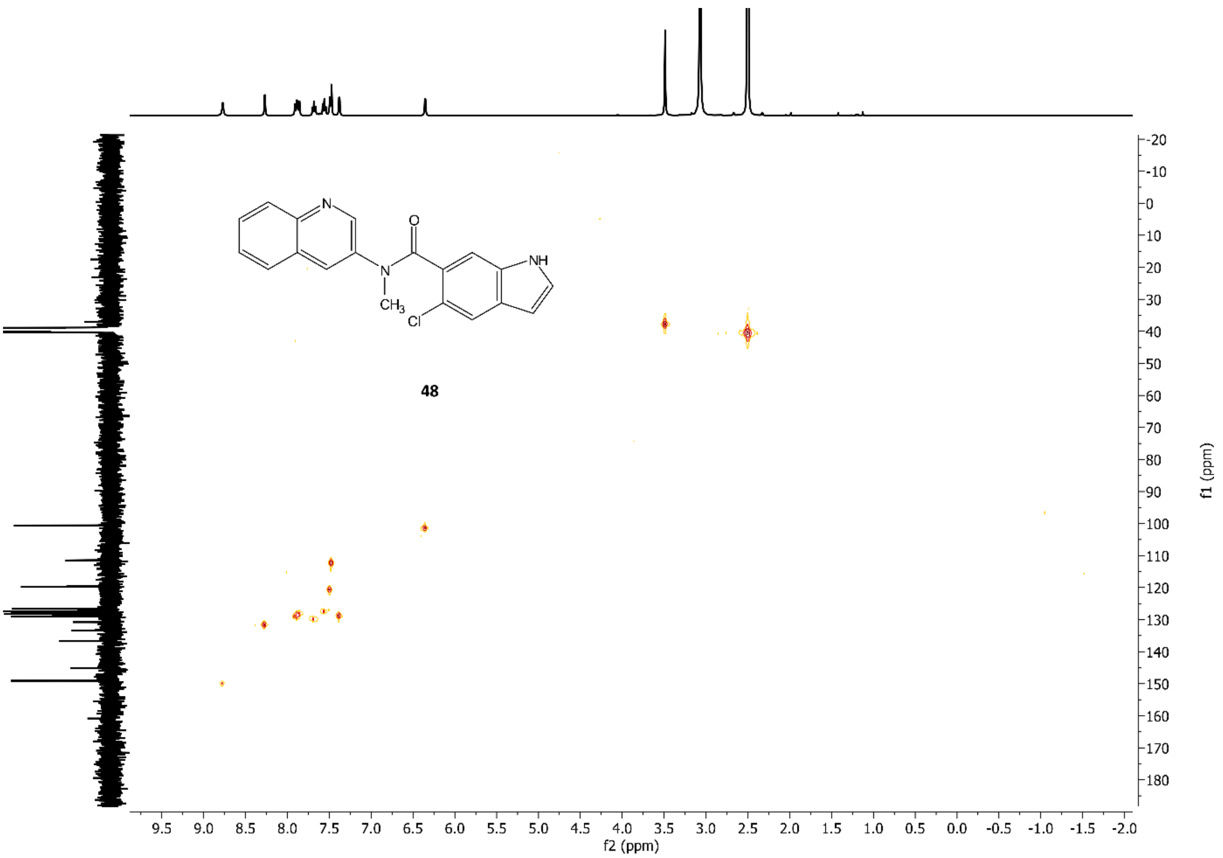

HSQC (DMSO-d6, 80°C) of compound **48**.

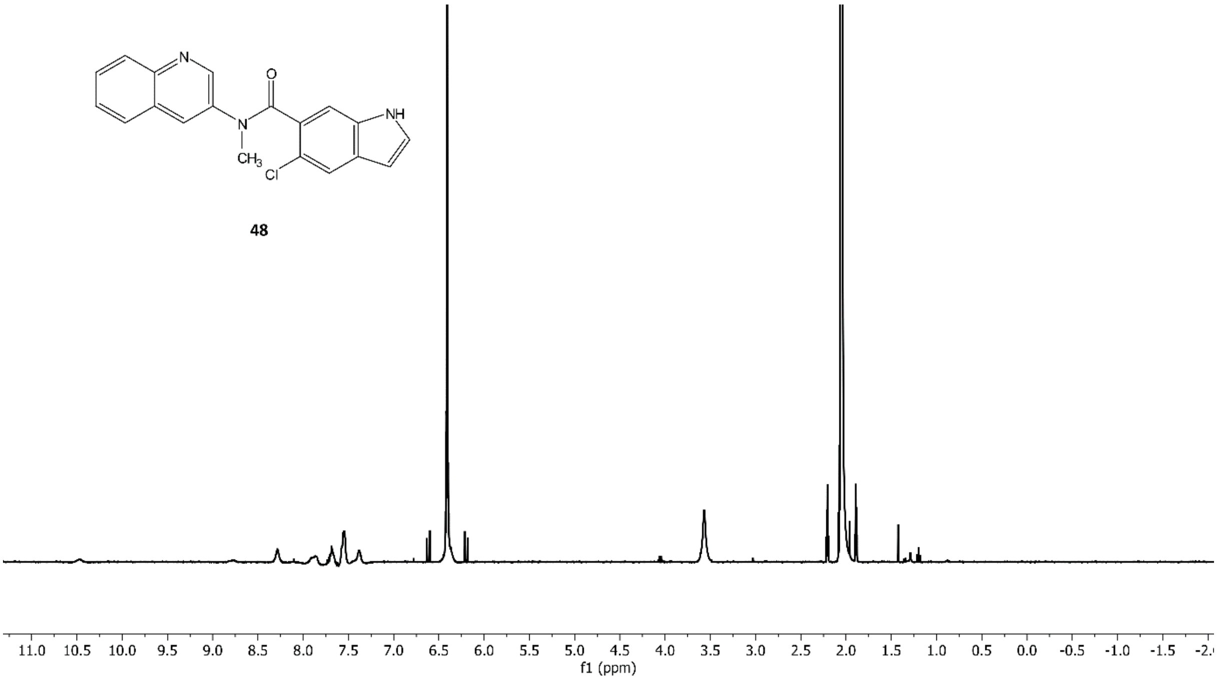

qHNMR (400 MHz, acetone-d6, rt, maleic acid as reference) of compound **48**. Signal broadening. No integration possible.

PDA chromatogram of compound **48**

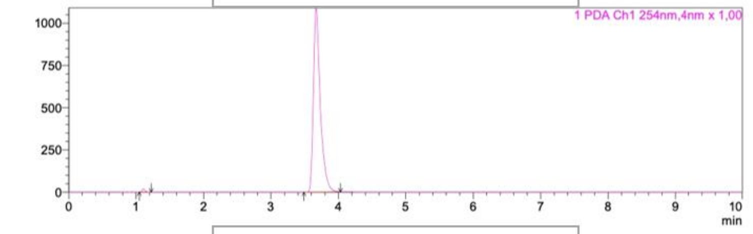

PDA peak table of compound **48**

PDA Ch1 254nm

Peak#	Ret. Time	Area	Height	Area%
1	1,106	56398	18169	0,724
2	3,670	7732240	1088614	99,276
Total		7788638	1106784	100,000

MS chromatogram of compound **48**

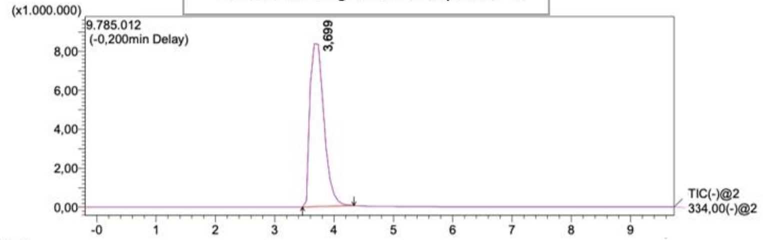

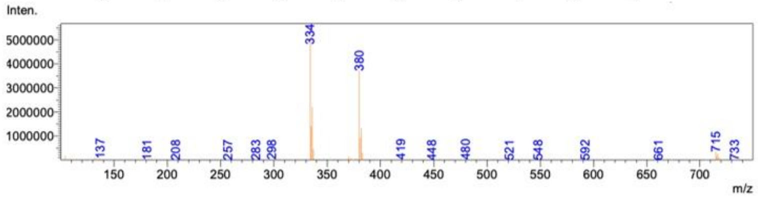

LC-MS purity analysis of compound **48**.

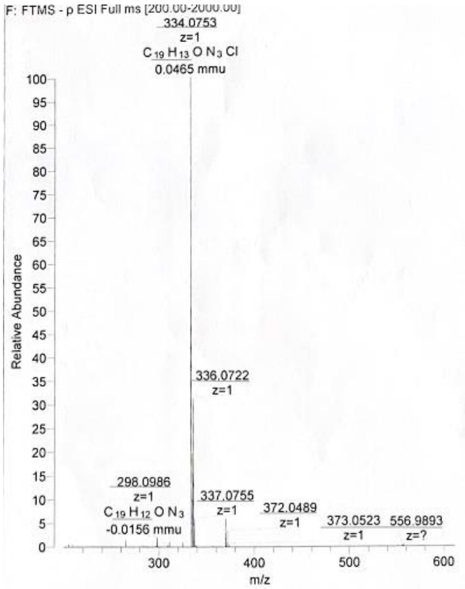

HRMS (ESI-) of compound **48**.

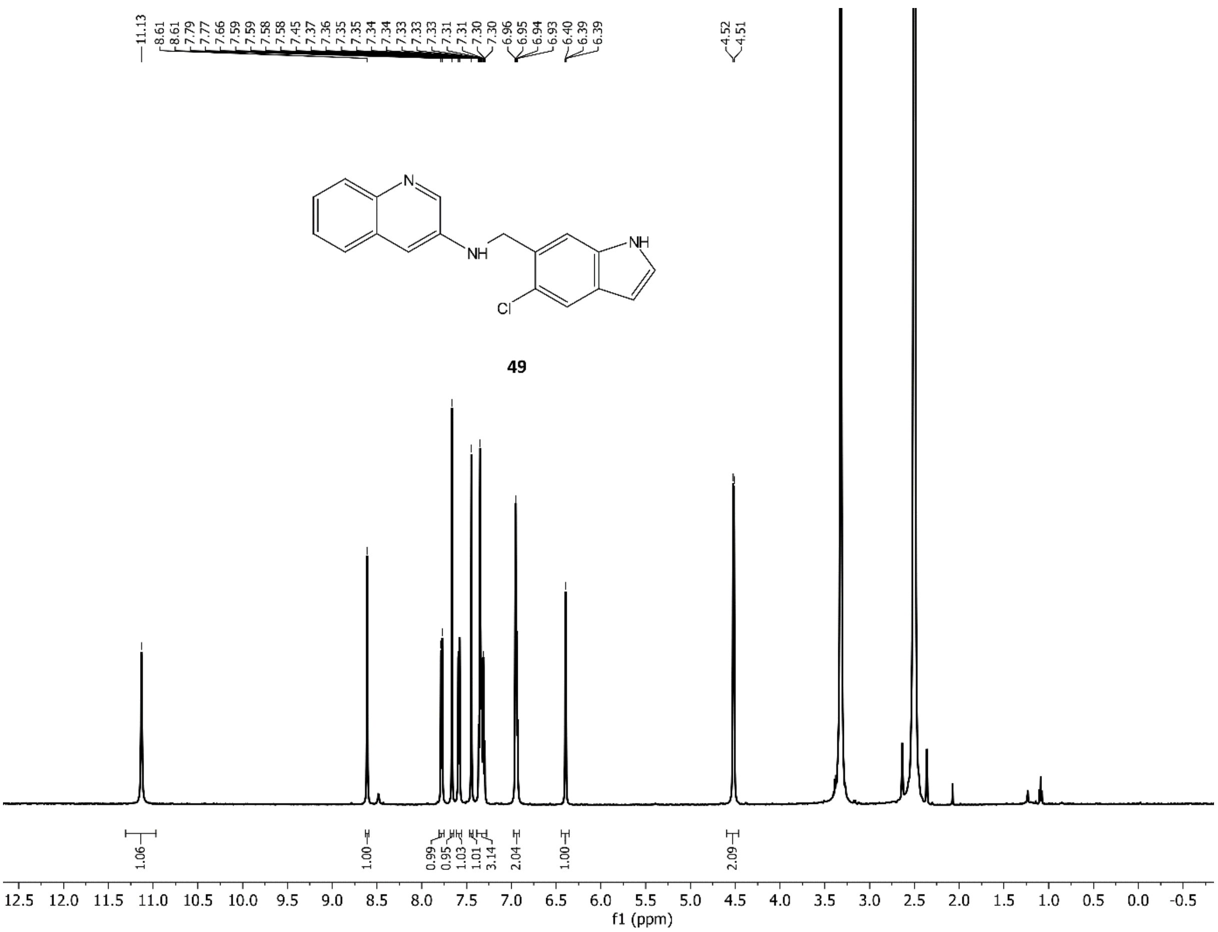

^{1}H-NMR (500 MHz, DMSO-d$_6$) of compound **49**.

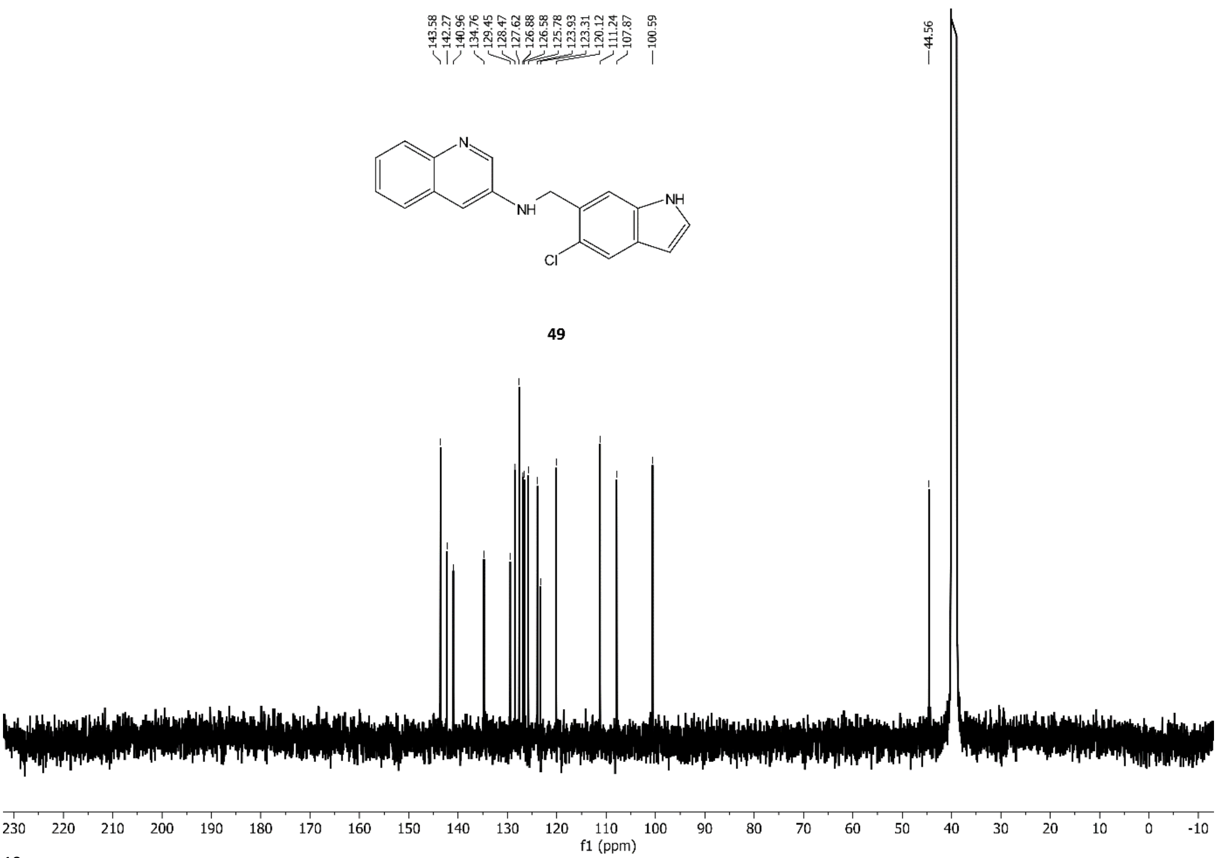

^{13}C-NMR (126 MHz, DMSO-d$_6$) of compound **49**.

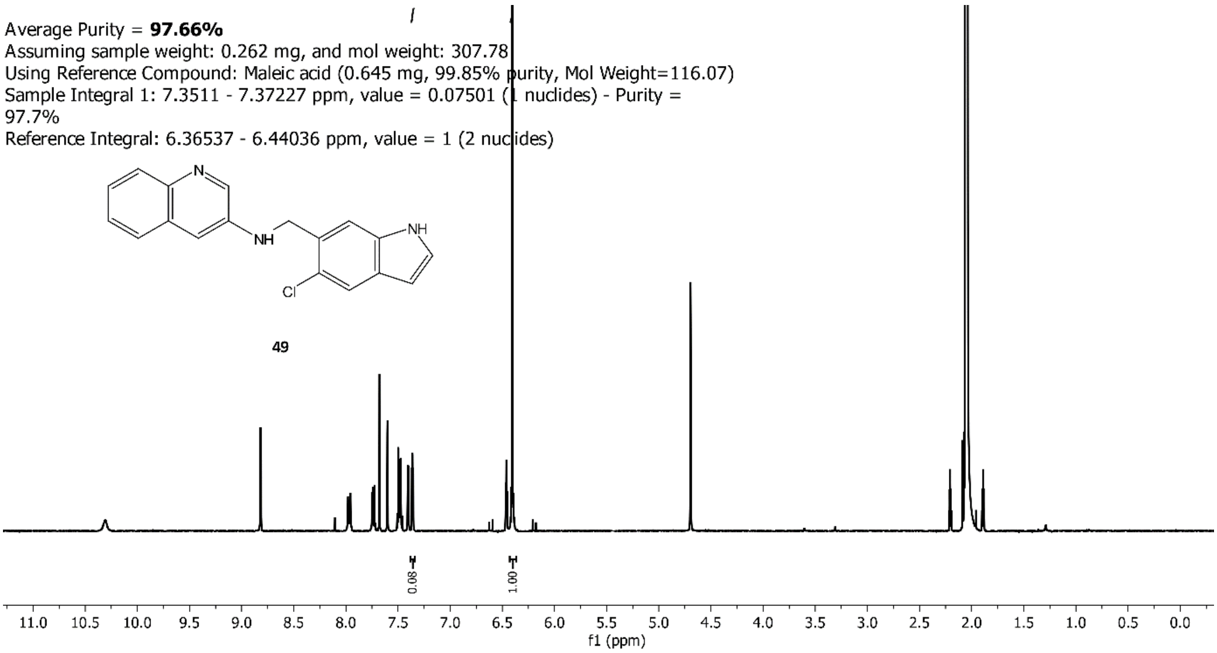

qHNMR (400 MHz, acetone-d$_6$, maleic acid as reference) of compound **49**.

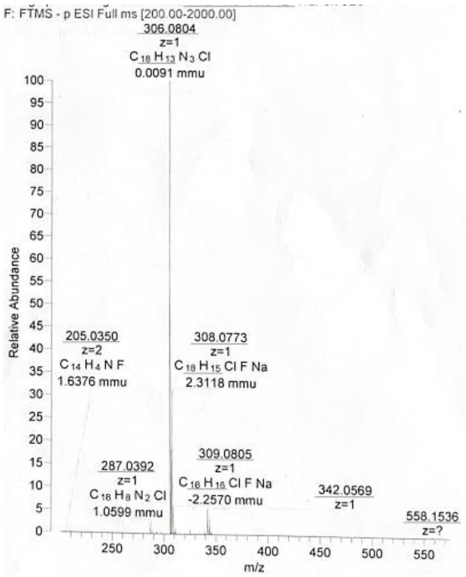

HRMS (ESI-) of compound **49**.

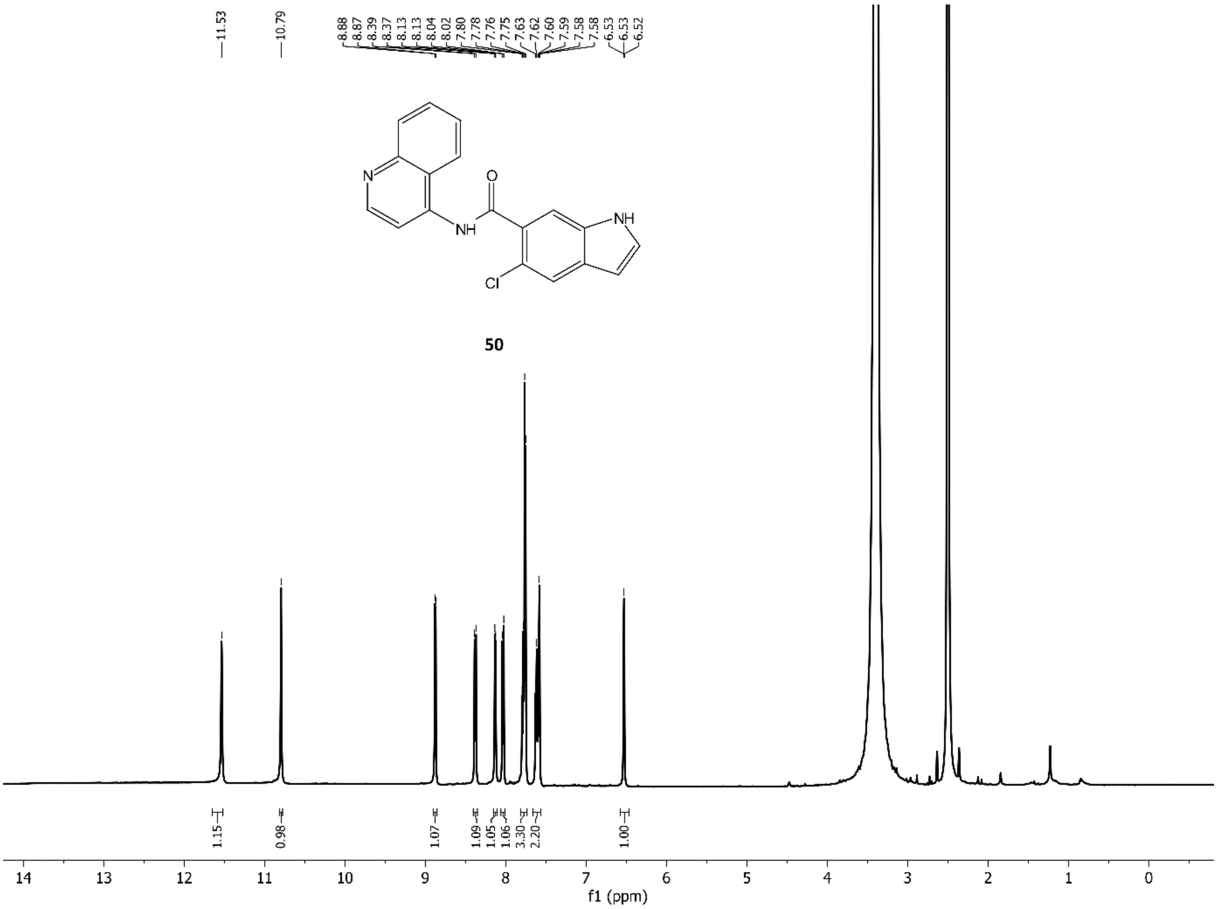

^{1}H-NMR (500 MHz, DMSO-d_6) of compound **50**.

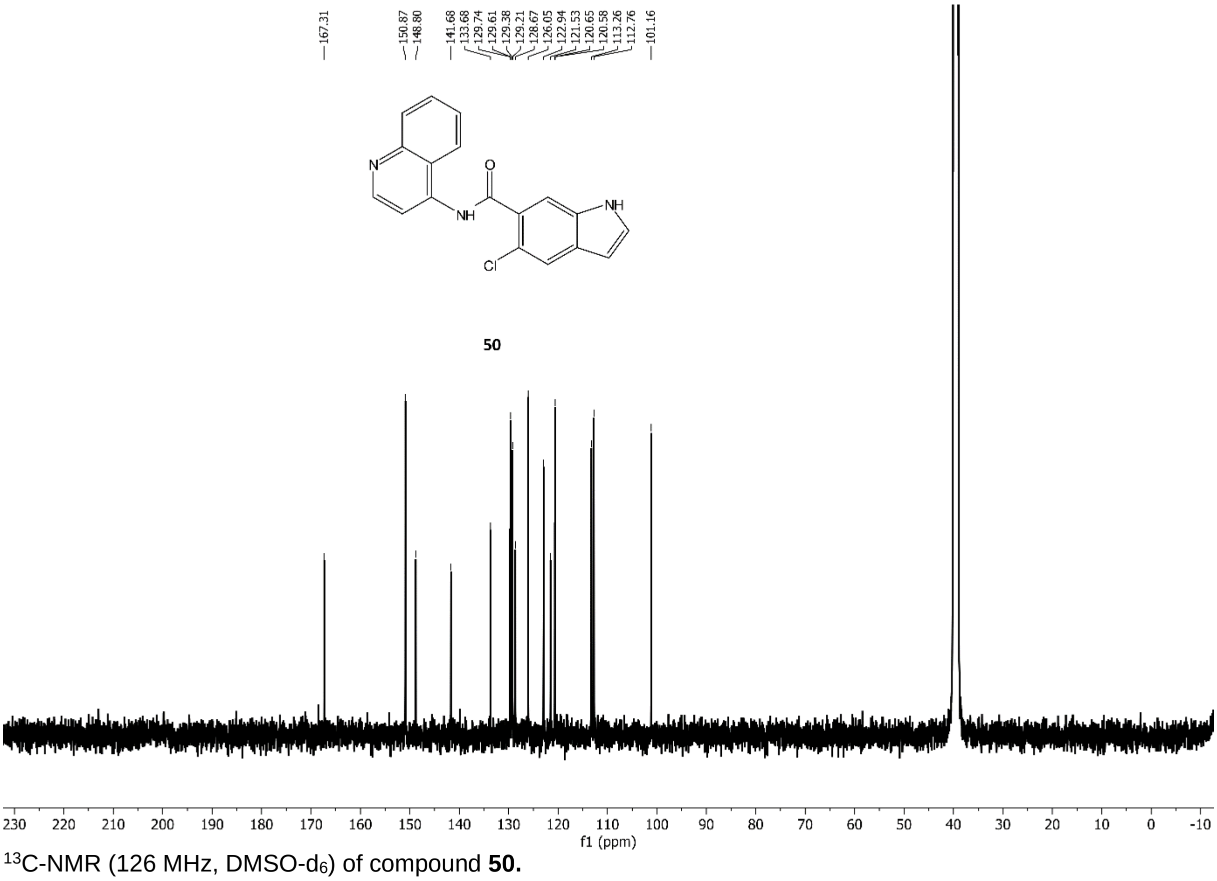

^{13}C-NMR (126 MHz, DMSO-d_6) of compound **50.**

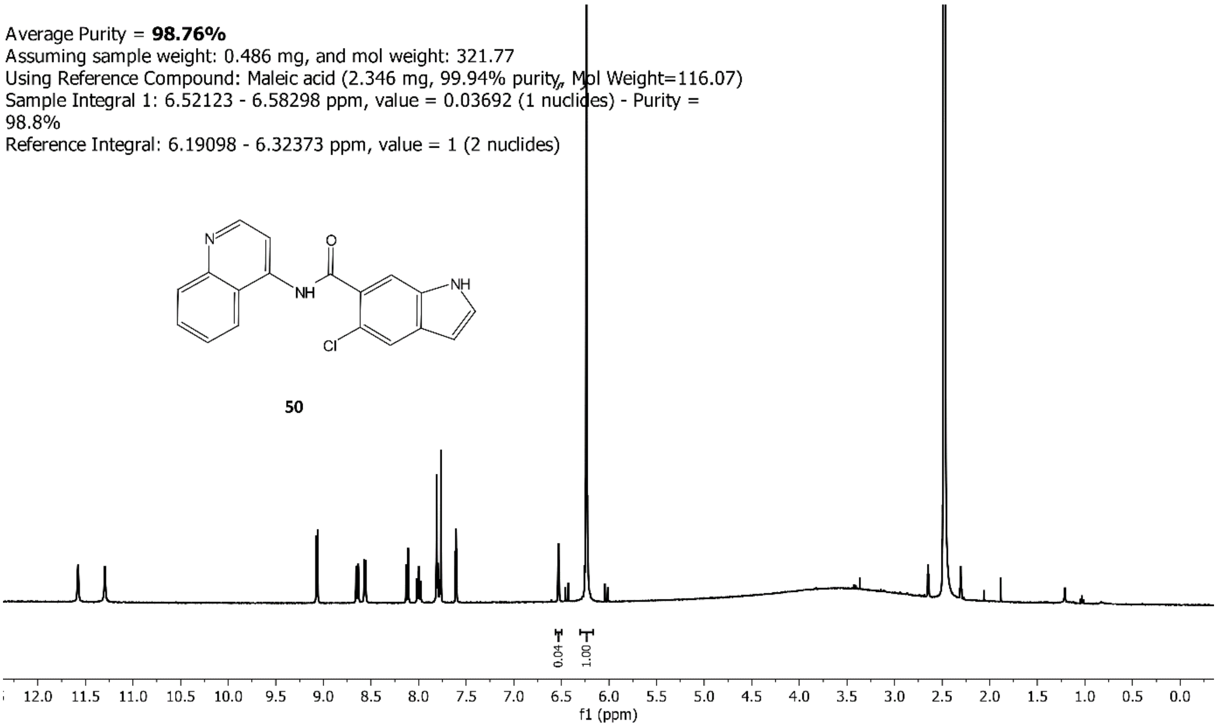

qHNMR (400 MHz, DMSO-d$_6$, maleic acid as reference) of compound **50**.

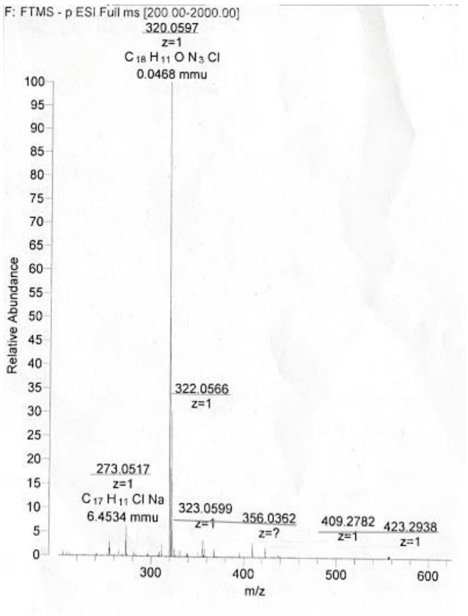

HRMS (ESI-) of compound **50**.

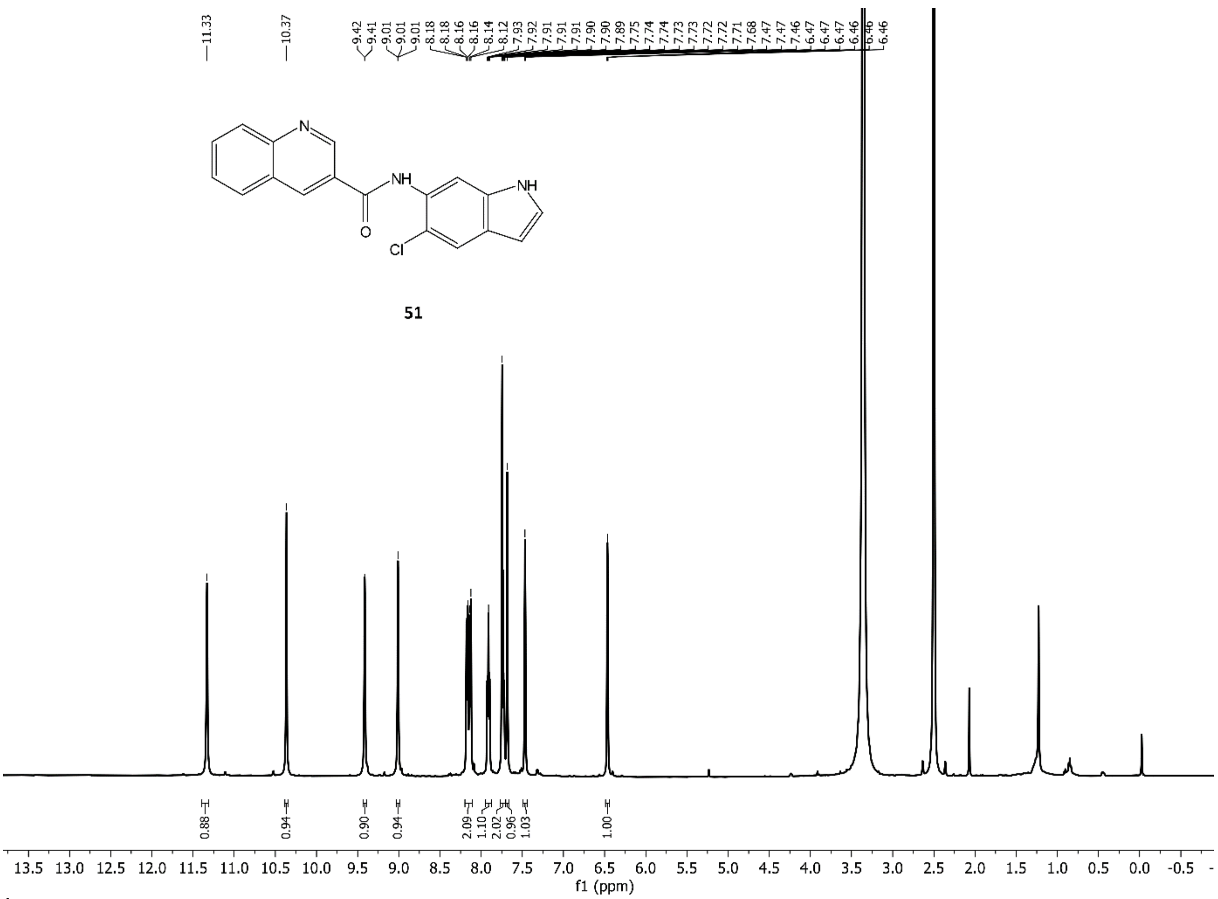

^{1}H-NMR (500 MHz, DMSO-d6) of compound **51**.

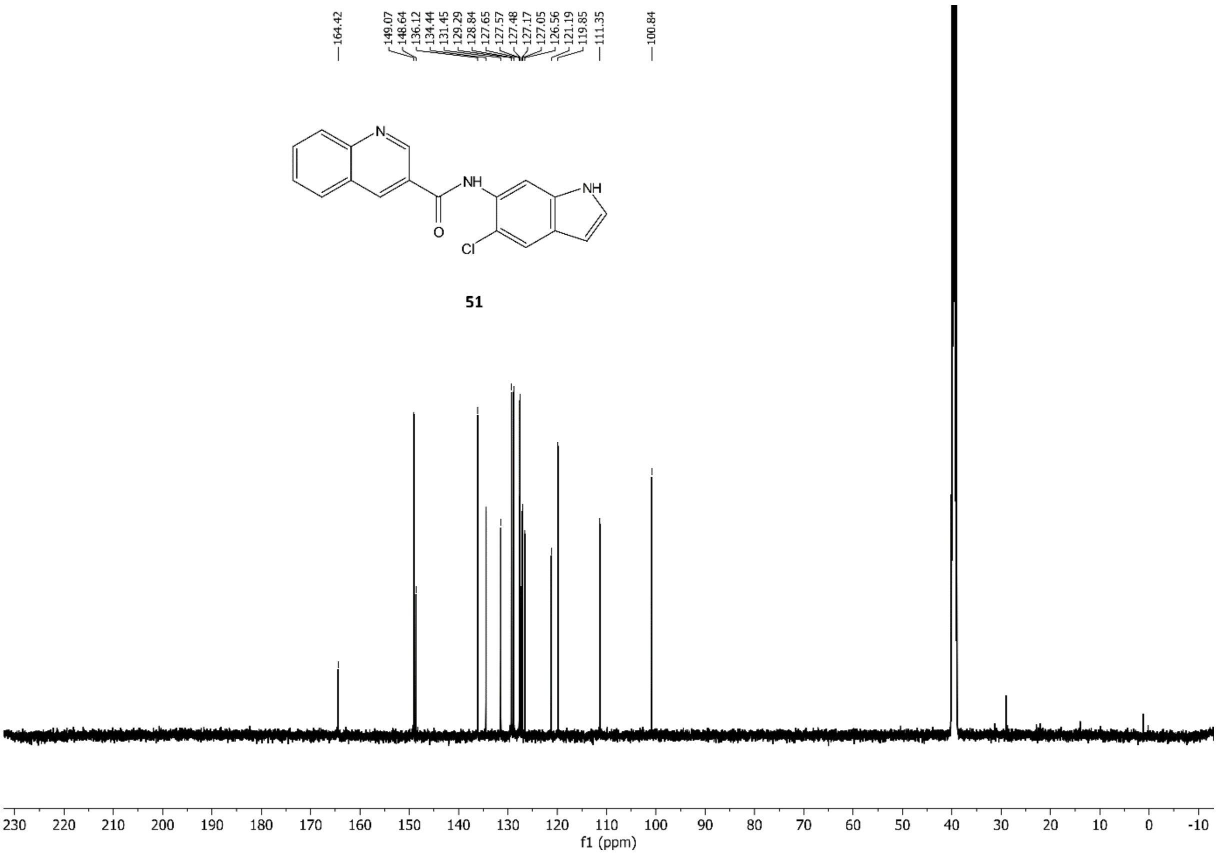

^{13}C-NMR (126 MHz, DMSO-d6) of compound **51**.

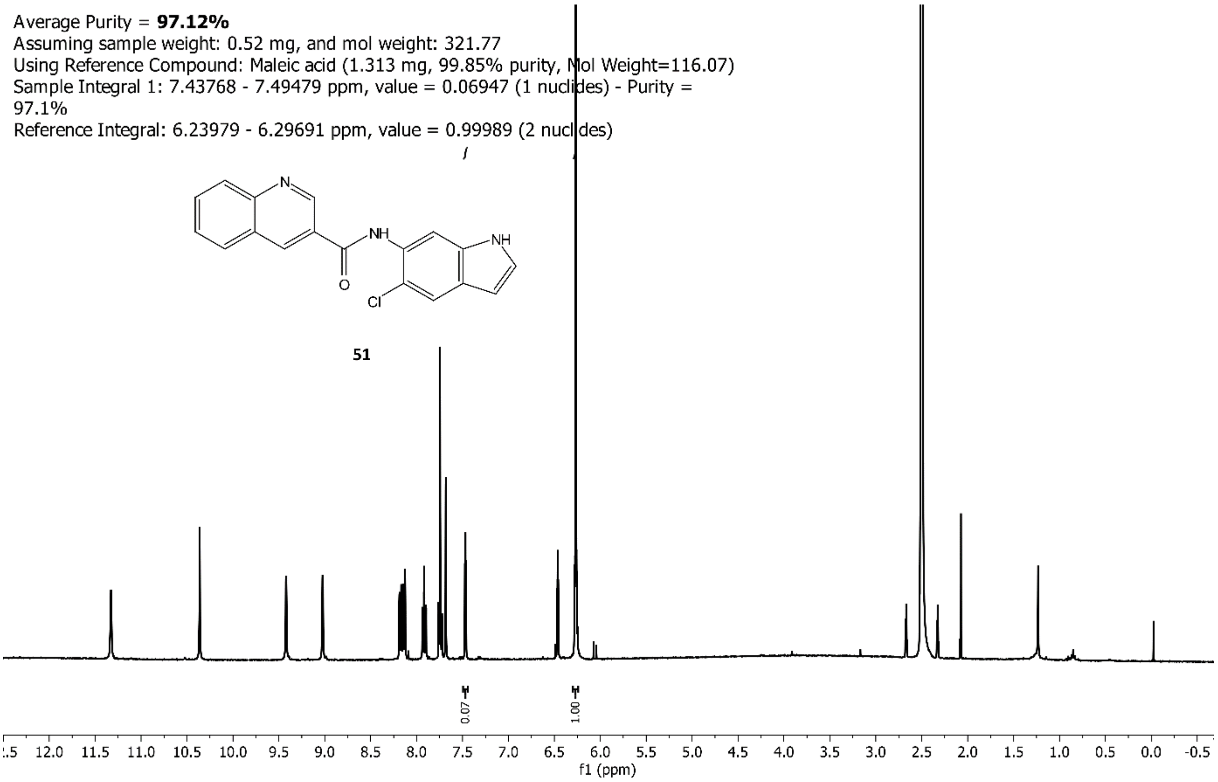

qHNMR (400 MHz, DMSO-d_6, maleic acid as reference) of compound **51**.

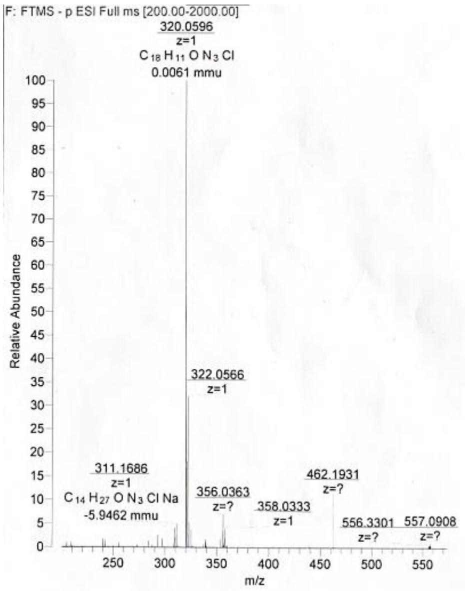

HRMS (ESI-) of compound **51**.
